# An explainable dual-modal diagnostic model for coronary artery disease: a feature-gated approach using tongue and facial image features

**DOI:** 10.3389/frai.2025.1662577

**Published:** 2025-11-17

**Authors:** Mei Zhao, Manrui Wang, Linmiao Fan, Mai Liu, Dongsheng Wei, Dong Dong, Xiaoqing Zhang

**Affiliations:** 1 Department of Life Sciences, Beijing University of Chinese Medicine, Beijing, China; 2 Department of Computer Science and Technology, Tsinghua University, Beijing, China

**Keywords:** coronary artery disease, diagnostic model, tongue feature, facial feature, feature-wise gating mechanism, explainable artificial intelligence (XAI)

## Abstract

**Background and objective:**

Coronary artery disease (CAD) is a major threat to human health, and early non-invasive identification is crucial for its prevention and management. However, current diagnostic methods still face limitations in terms of non-invasiveness, cost, and accessibility. Tongue and facial features have been recognized as closely associated with CAD. To address these challenges, this study proposes a dual-modal diagnostic model incorporating a feature-wise gating mechanism to enable intelligent, non-invasive CAD detection based on tongue and facial images.

**Methods:**

A total of 936 participants were enrolled in this study, and standardized tongue and facial images were collected from each subject. Image segmentation was performed using MedSAM, followed by deep semantic feature extraction using the MDFA-Swin network. Traditional color and texture features were also incorporated. A feature-guided gating mechanism was developed to enable personalized multimodal fusion of tongue and facial features. The diagnostic performance of the proposed model was evaluated on an independent external test set. In addition, SHAP (SHapley Additive Explanations) analysis were conducted to enhance model interpretability.

**Results:**

The proposed CAD diagnostic model based on fused multidimensional tongue and facial features (TF_FGC) demonstrated excellent performance in internal validation (AUC = 0.945, Accuracy = 0.872) and maintained good generalizability on the external test set (AUC = 0.896, Accuracy = 0.825). The SHAP analysis identified T_contrast, T_RGB_R, T_homogeneity, F_homogeneity, F_RGB_B, F_RGB_G, F_RGB_R, and F_contrast as the most influential features driving model predictions.

**Conclusion:**

The proposed dual-branch fusion model demonstrates high diagnostic accuracy, strong interpretability, and good generalizability. By integrating traditional color and texture features with deep semantic representations, this approach offers a promising solution for non-invasive and intelligent screening of CAD, providing a novel perspective and practical support for clinical decision-making.

## 1 Introduction

For Coronary artery disease (CAD), as one of the major cardiovascular diseases posing a significant threat to human health (Ainiwaer et al., 2024), has become a leading cause of mortality worldwide (Benjamin et al., 2017; Benjamin et al., 2018). Unfortunately, delayed diagnosis often places CAD patients at risk of myocardial infarction and even sudden cardiac death (Lee et al., 2019). Therefore, achieving rapid and accurate screening and diagnosis of CAD is crucial for its early detection, effective treatment, and disease management. Although coronary angiography is currently regarded as the gold standard for CAD diagnosis, its invasive nature and high cost limit its widespread use in large-scale screening and early diagnosis (Wood et al., 2024). Other diagnostic methods often rely heavily on advanced equipment and the interpretative expertise of specialized physicians, making them difficult to implement in resource-limited settings. Therefore, developing a more rapid, non-invasive, accessible, and cost-effective diagnostic method for CAD is not only critical for clinical management but also an urgent necessity for effective large-scale population screening.

In traditional Chinese medicine (TCM), it is believed that internal pathological changes can be inferred through external bodily manifestations—a concept known as ‘observing the exterior to understand the interior’ (司外揣内) (Liang and Gu, 2021). Tongue diagnosis and facial diagnosis, as integral components of TCM diagnostics, assess changes in tongue appearance and facial features to reflect the circulation of qi and blood as well as the functional state of internal organs, thereby supporting disease diagnosis (Xie et al., 2021; Duan et al., 2024b). Modern studies have shown that tongue and facial features may serve as effective biomarkers for the diagnosis of CAD. Anatomically, the blood supply to the tongue is closely related to the coronary circulation (Bavitz et al., 1994; Wu et al., 2007). Coronary atherosclerosis can lead to myocardial ischemia and impair peripheral microcirculation, particularly the microcirculation of the tongue, resulting in abnormal changes such as a dark red or bluish-purple tongue body or the presence of petechiae and stasis spots (Kagami et al., 2012; Wang et al., 2022). Clinical observations have shown a correlation between tongue color features and the degree of coronary artery stenosis in patients with CAD (Xia et al., 2023; Li et al., 2024). In addition, facial features such as complexion and skin texture are also associated with CAD risk; patients often exhibit signs such as a dull facial appearance and dark red lips (Christoffersen et al., 2014; Christoffersen and Tybjærg-Hansen, 2016). Lin et al. (2020) through a multicenter prospective study, demonstrated the clinical feasibility of a CAD diagnostic model based on facial images, achieving an AUC of 0.730, further supporting the application value of facial features in CAD screening. In summary, tongue and facial characteristics are closely related to CAD, suggesting that these non-invasive physiological indicators have the potential to serve as novel biomarkers for CAD diagnosis and risk assessment.

**Figure 1:**
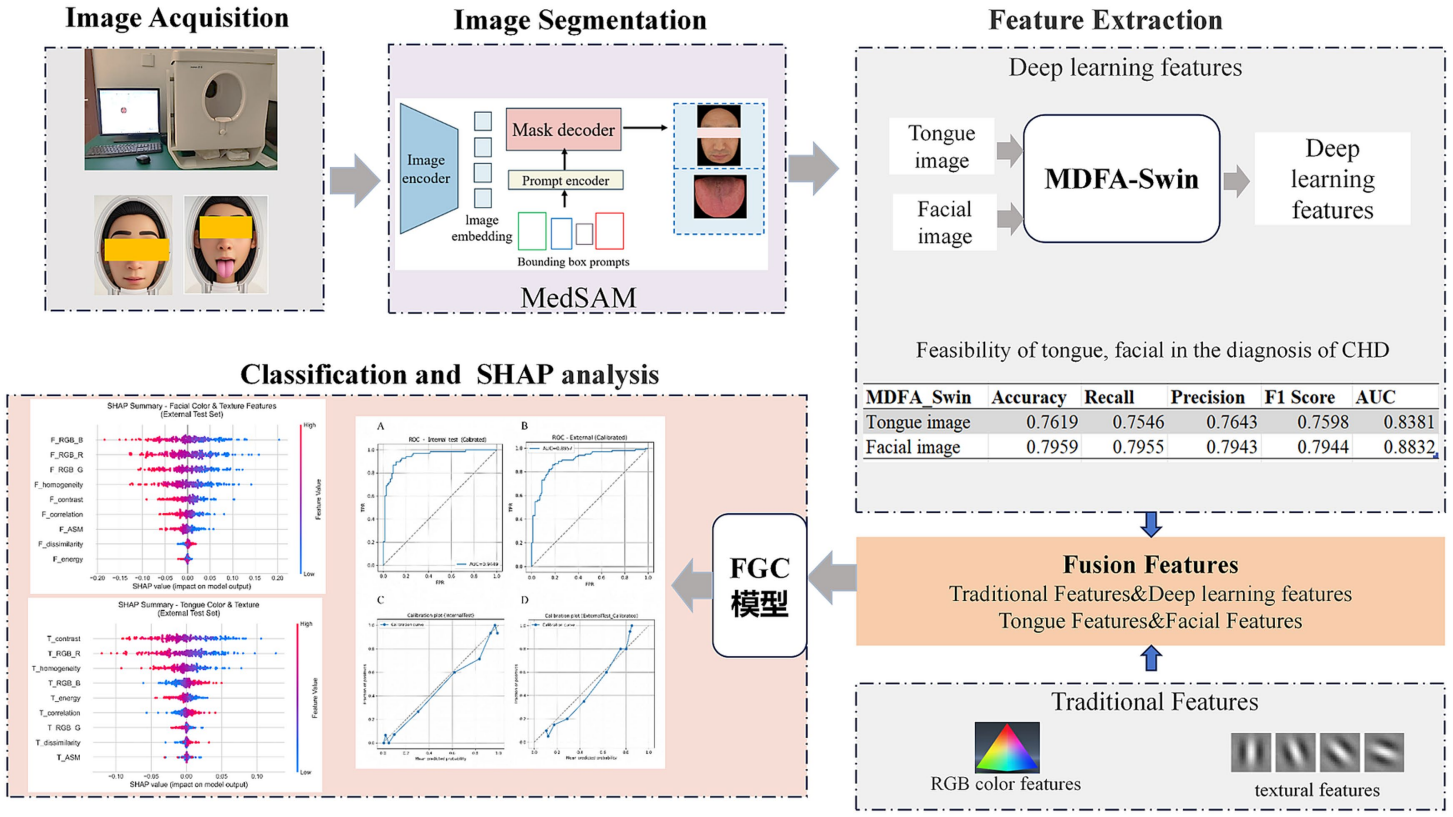
Architecture of the feature-gated classifier (FGC) model.

In recent years, numerous researchers have leveraged artificial intelligence technologies to develop intelligent diagnostic models for CAD by integrating multi-source data such as medical imaging, blood biomarkers, and electronic medical records (Coenen et al., 2018; Vallée et al., 2019; Shen et al., 2024; Addisu et al., 2025). These models have introduced new approaches for early disease detection and risk assessment. However, their effectiveness largely depends on access to high-quality clinical data, the acquisition of which often involves complex procedures and high costs, thereby limiting their practical implementation in primary healthcare settings and large-scale population screening. In contrast, tongue and facial image data offer advantages such as ease of acquisition, non-invasiveness, and low cost. Existing studies have demonstrated the potential clinical value of these features in supporting CAD diagnosis, yet their broader application in real-world medical scenarios remains underexplored and lacks systematic integration. Currently, most studies on CAD diagnosis rely solely on either tongue features or facial features, and few have explored models that integrate multi-dimensional features from both modalities. To address the challenges faced in CAD diagnosis, this study proposes a non-invasive diagnostic model based on the integration of tongue and facial features. The model introduces a feature-wise gating mechanism to enable adaptive weighted fusion of tongue and facial features under multimodal input, thereby improving both individualized diagnostic accuracy and the model’s discriminative capability. In addition, the model incorporates the SHapley Additive exPlanations (SHAP) method to analyze the contribution of each feature, enhancing interpretability and providing more reliable decision support for clinical practice (Pearson, 1901; Yu et al., 2025). Notably, the model is well-suited for a wide range of application scenarios, including community health centers, physical examination institutions, and daily home health monitoring. It does not rely on advanced medical equipment or specialized personnel, allowing for efficient diagnosis and timely medical guidance for patients. The workflow of proposed methodology is shown in Figure 1.

## 2 Materials and methods

### 2.1 Data source

The data for this study were collected from Dongcheng Hospital and Tongzhou Hospital of Dongzhimen Hospital, Beijing University of Chinese Medicine, between July 2023 and July 2024. A total of 737 participants were enrolled at Tongzhou Hospital, including 337 patients with CAD and 400 non-CAD controls, which were used for model training and internal validation. An independent external validation cohort consisted of 200 participants (100 CAD patients and 100 non-CAD controls) recruited from Dongcheng Hospital during the same period. Detailed baseline characteristics of all cohorts are presented in Table 1.

**Table 1:** Basic information of participants.

Item	Train and internal validation dataset		
	CAD (*n* = 337)	NCAD (*n* = 400)	*P* value
Age (years)	65.17 ± 10.086	63.9 ± 10.046	0.06
Sex (male/female)	195/142	228/172	0.81
Hypertension	219	148	<0.0001
Diabetes	118	80	<0.0001

	Independent external validation dataset		
	CAD (*n* = 100)	NCAD (*n* = 100)	*P* value
Age (Years)	65.41 ± 10.179	64.96 ± 10.315	0.78
Sex (male/female)	56/44	54/46	0.78
Hypertension	63	31	<0.0001
Diabetes	34	18	0.012

To control for major demographic confounders, CAD and non-CAD participants were matched on age (±5 years) and sex. Other cardiovascular risk factors such as hypertension and diabetes were not used as matching criteria, because these conditions are established risk factors and potential mediators of CAD, and their associated facial and lingual phenotypes may contribute to the diagnostic signal captured by our image-based model. This design preserves real-world differences that the model is intended to recognize and avoids over-adjustment of true disease characteristics. Consequently, the prevalence of CAD in the study cohort was approximately 50%, which is higher than that in typical clinical populations; therefore, the primary goal of this study was to evaluate discriminative ability rather than to estimate absolute risk in the general population.

All CAD patients included in the study were diagnosed based on coronary angiography (CAG). Obstructive CAD was defined as a stenosis of ≥50% in at least one major coronary artery (left anterior descending artery, left circumflex artery, right coronary artery, or left main stem), consistent with diagnostic thresholds recommended in major international guidelines and clinical practice (Knuuti et al., 2020). Patients meeting this criterion were classified as CAD-positive, whereas those with <50% stenosis were classified as CAD-negative. To ensure consistency between image acquisition and reference diagnostic testing, all participants completed facial and tongue imaging within 2 weeks of their CAG examination during the same hospitalization. All CAD patients were symptomatic cases who underwent angiography due to suspected ischemic manifestations (e.g., chest pain, exertional angina, or positive stress test), rather than asymptomatic incidental findings. Although a stenosis ≥70% is often considered hemodynamically significant (“severe stenosis”) in clinical settings, we adopted the 50% threshold because it represents the internationally accepted definition of obstructive CAD and has been widely used in clinical trials and diagnostic model development.

Non-CAD controls were carefully selected to match the CAD group in age and sex distribution (no significant differences observed, see Table 1) and met the following criteria: (i) aged 18–85 years, conscious, without psychiatric disorders, and able to complete the full image acquisition process; (ii) no visible facial, oral, or tongue deformities; (iii) no history of CAD or other severe cardiovascular diseases (e.g., heart failure, cardiomyopathy, congenital heart disease); (iv) no major chronic illnesses such as renal insufficiency, cirrhosis, or malignancy; (v) not on long-term immunosuppressants, glucocorticoids, or other medications that may significantly affect cardiovascular metabolism.

This study was approved by the Medical Ethics Committee of Dongzhimen Hospital, Beijing University of Chinese Medicine (approval number: 2023DZMEC-228-03), and written informed consent was obtained from all participants.

### 2.2 Data acquisition and cross-center colorimetry consistency

**Figure 2:**
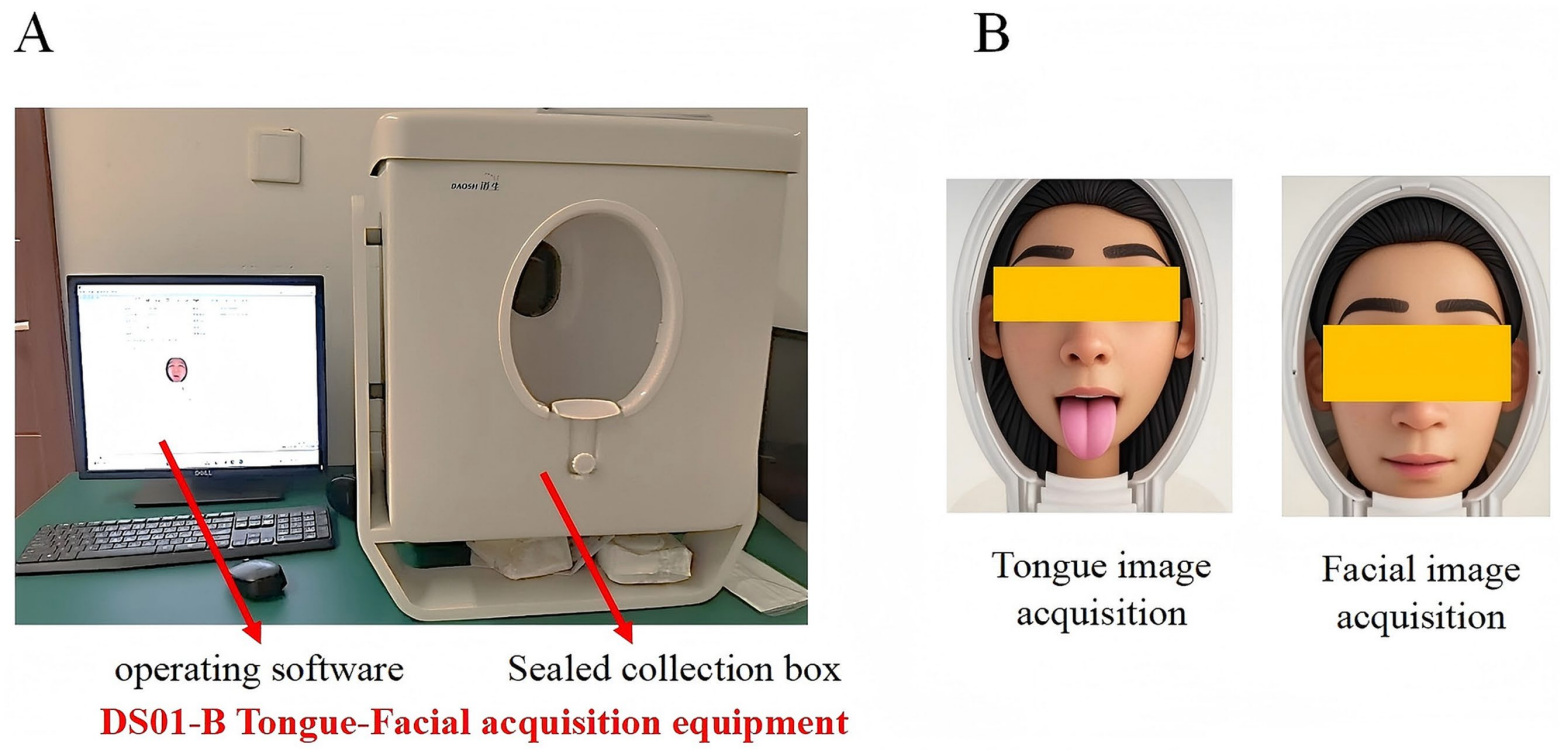
**(A)** Appearance and operation interface of the DS01-B tongue and facial image acquisition device. **(B)** Schematic illustration of tongue and facial image acquisition.

Tongue and facial images were acquired using the same model of imaging device, the DS01-B Tongue Diagnosis Instrument (Model: DS01-B, Product No.: YM0100520, Registration No.: Shanghai Medical Device Registration 20,202,200,062; Shanghai Daosheng Medical Technology Co., Ltd., China). The system is equipped with an independent power supply and a sealed acquisition chamber, incorporating a Canon EOS 1200D DSLR camera (18 megapixels) and an LED cold light source that simulates natural daylight (color temperature: 4,500–6,500 K; color rendering index, CRI ≥ 90). All devices were spectrally calibrated before deployment using a 1.5 m integrating sphere to ensure long-term imaging stability (see Figure 2).

Imaging parameters were strictly standardized: shutter speed 1/200 s, aperture f/5.6, ISO 200, illumination intensity maintained at 3,000 ± 10% LUX, and relative distortion ≤ ± 5%. Color fidelity was verified using a standard 24-color calibration chart, requiring the color deviation between measured and reference CIE LAB values (ΔE*ab) to be ≤10. All images were acquired indoors under controlled lighting conditions by uniformly trained personnel. Participants were photographed approximately 2 h after a meal, seated upright with the chin stabilized on a support. During tongue imaging, subjects extended the tongue naturally and slightly downward, followed by facial image collection. Image quality was monitored in real time, and resampling was performed if occlusion, blur, or distortion was detected until high-quality images were obtained.

To verify cross-center imaging consistency, the external validation set was collected using the same DS01-B system under an identical acquisition protocol. A colorimetry consistency analysis was further conducted across the two centers by computing CIE ΔE*ab color differences and performing Bland–Altman analyses for the L*, a*, and b* channels. Results showed that the median cross-center ΔE*ab was 4.48 (IQR: 3.75–6.91) for facial images and 5.42 (IQR: 4.11–8.39) for tongue images, both below the generally accepted threshold for medical imaging tasks (ΔEab < 10). No systematic bias was observed between centers; only moderate random variations were found, primarily along the lightness (L) and red–green (a)* dimensions (see Supplementary Figure S2). These findings confirm excellent cross-center color consistency, ensuring the reliability of subsequent model performance evaluation.

### 2.3 Segmentation of tongue and facial image regions

**Figure 3:**
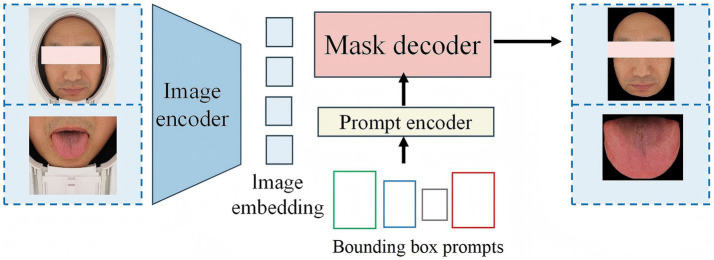
Overview of the architecture of MedSAM.

Currently, there are relatively few segmentation algorithms specifically designed for TCM data. In the past, training a high-quality medical image segmentation model required extensive manual annotation, which is labor-intensive. This is particularly challenging in the field of TCM due to the lack of public data and the specialized nature of data collection and annotation. MedSAM (Ma J. et al., 2024) is an optimized model based on SAM (Mazurowski et al., 2023), designed to accommodate various medical image segmentation tasks (Figure 3). MedSAM significantly improves the recognition and segmentation of targets in medical images through fine tuning on large medical image datasets. It demonstrates strong zero-shot generalization across various medical tasks, significantly reducing the burden of manual annotation (Zhang et al., 2024). However, to date, no studies have applied MedSAM to the segmentation of tongue and facial images. In this study, we used MedSAM to segment the tongue and facial regions. Segmentation was performed using automatic point prompting with default parameters, without fine-tuning or threshold post-processing on our dataset. To ensure segmentation quality, images were strictly controlled during acquisition, with blurred, occluded, or low-quality images excluded. To evaluate segmentation performance, we randomly selected 200 images from the dataset (100 tongue, 100 face), and two annotators with experience in TCM imaging performed pixel-level annotations using LabelMe. The results showed that MedSAM achieved high segmentation performance for both tongue and facial images, effectively excluding irrelevant background regions (Tongue image: IoU = 0.9393, Dice = 0.9687; Facial image: IoU = 0.9676, Dice = 0.9515).

### 2.4 Deep feature extraction based on MDFA-Swin

**Figure 4:**
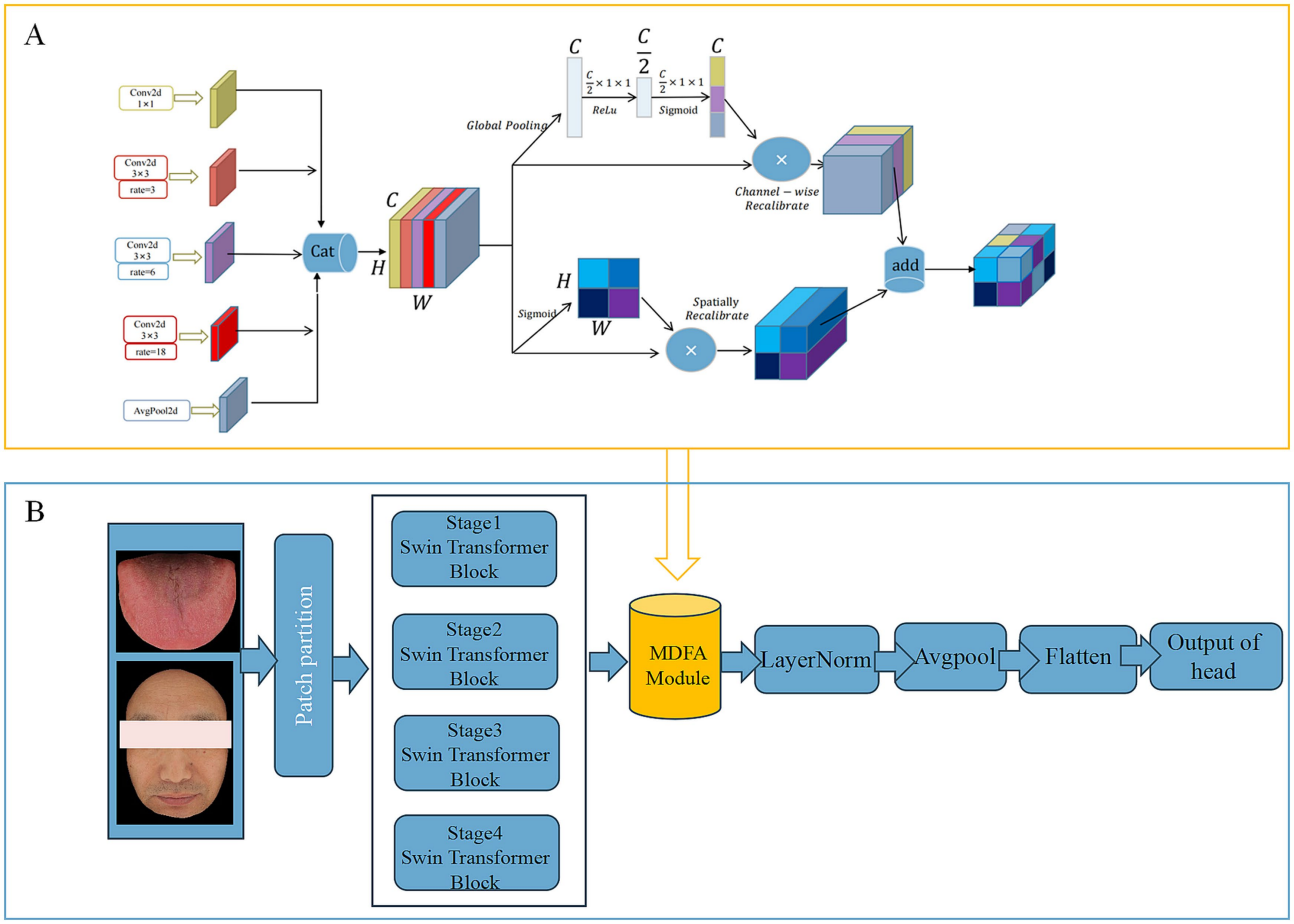
**(A)** Network structure of MDFA module. **(B)** MDFA-Swin-Transformer.

To effectively capture high-level semantic features (Deep Feature) embedded in tongue and facial images, this study adopts the MDFA-Swin network (Figure 4) as the backbone model for deep feature extraction. The Swin Transformer (Liu et al., 2021) adopts a hierarchical structure combined with a shifted window-based multi-head self-attention mechanism, which offers strong local perception and global modeling capabilities. This makes it particularly well-suited for analyzing medical images of the tongue and face, which often exhibit unstructured morphology, fine-grained variations, and blurred boundaries. To enhance the model’s ability to capture local details, a Multi-scale Dilated Fusion Attention (MDFA) module—integrating multi-scale dilated convolutions and attention mechanisms—is incorporated. This component strengthens the model’s capacity to perceive and represent key diagnostic features. During the feature extraction process, both tongue and facial images first undergo initial transformation through a patch partitioning and linear embedding module. These representations are then processed through multiple Transformer blocks to progressively integrate cross-scale contextual information. For high-level feature representation, we retain the global feature output preceding the classification head—specifically, the feature vector obtained after the ‘avgpool’ operation but before the ‘flatten’ operation. This semantic-level abstraction provides discriminative power for subsequent feature fusion and disease prediction tasks. The final deep feature is flattened into a one-dimensional vector of size 768 using ‘torch.flatten (x, 1)’. We further evaluated the discriminative power of deep features extracted by MDFA-Swin from tongue and facial images, and conducted systematic comparisons with several mainstream baseline models, including ViT-B/16, Swin-Small, Swin-Tiny, ResNet18, ResNet34, and ResNet50. Experimental results demonstrated that MDFA-Swin outperforms these baselines across multiple performance metrics, confirming its effectiveness and suitability for tongue and facial image analysis tasks.

### 2.5 MDFA-Swin model training

The diagnostic model in this study was developed using the Python programming language and implemented with the PyTorch deep learning framework. All experiments were conducted in a high-performance computing environment equipped with dual NVIDIA A100 GPUs. Considering the inherent differences in feature distribution between tongue and facial images, distinct optimizers and hyperparameter configurations were applied to optimize model performance. For the tongue image dataset, we adopted the Stochastic Gradient Descent (SGD) optimizer with a momentum of 0.9, a weight decay of 5 × 10^−4^, and an initial learning rate of 5 × 10^−4^. In contrast, facial image training employed the Adam optimizer with an initial learning rate of 5 × 10^−5^ and a momentum parameter of 0.9 to accommodate its higher complexity in feature distribution. To enhance model stability and convergence, a cosine annealing learning rate scheduler was introduced, decaying the learning rate to one-tenth of its initial value every 50 training epochs. Training was conducted for a total of 200 epochs with a consistent batch size of 32. A fixed random seed of 42 was used to ensure reproducibility of the experimental results. The model with the best validation accuracy was preserved during training based on dynamic monitoring of validation performance, and the corresponding weights were retained for subsequent testing and analysis (Song et al., 2024).

The high-level semantic features of tongue and facial images extracted by the MDFA-Swin model heavily rely on the black-box mechanisms of deep neural networks, resulting in limited clinical interpretability. This limitation poses challenges in meeting the requirements for diagnostic transparency and reliability in real-world applications. To enhance the interpretability and generalizability of the model, this study further incorporates traditional color and texture features of tongue and facial images, which are inherently more explainable. For color characterization, the average R, G, and B values in the RGB color space are employed as quantitative indicators of overall image color (Xie et al., 2021), aligning with TCM theories of color-based diagnosis. Regarding texture, gray-level co-occurrence matrix (GLCM) are used to extract spatial gray-level distribution and directional texture responses. These features effectively capture surface roughness, textural regularity, and spatial frequency characteristics of the tongue tissue, thereby complementing the high-level semantic features with more interpretable and detailed information.

### 2.6 Construction of a diagnostic model for coronary artery disease

#### 2.6.1 Model architecture design

**Figure 5:**
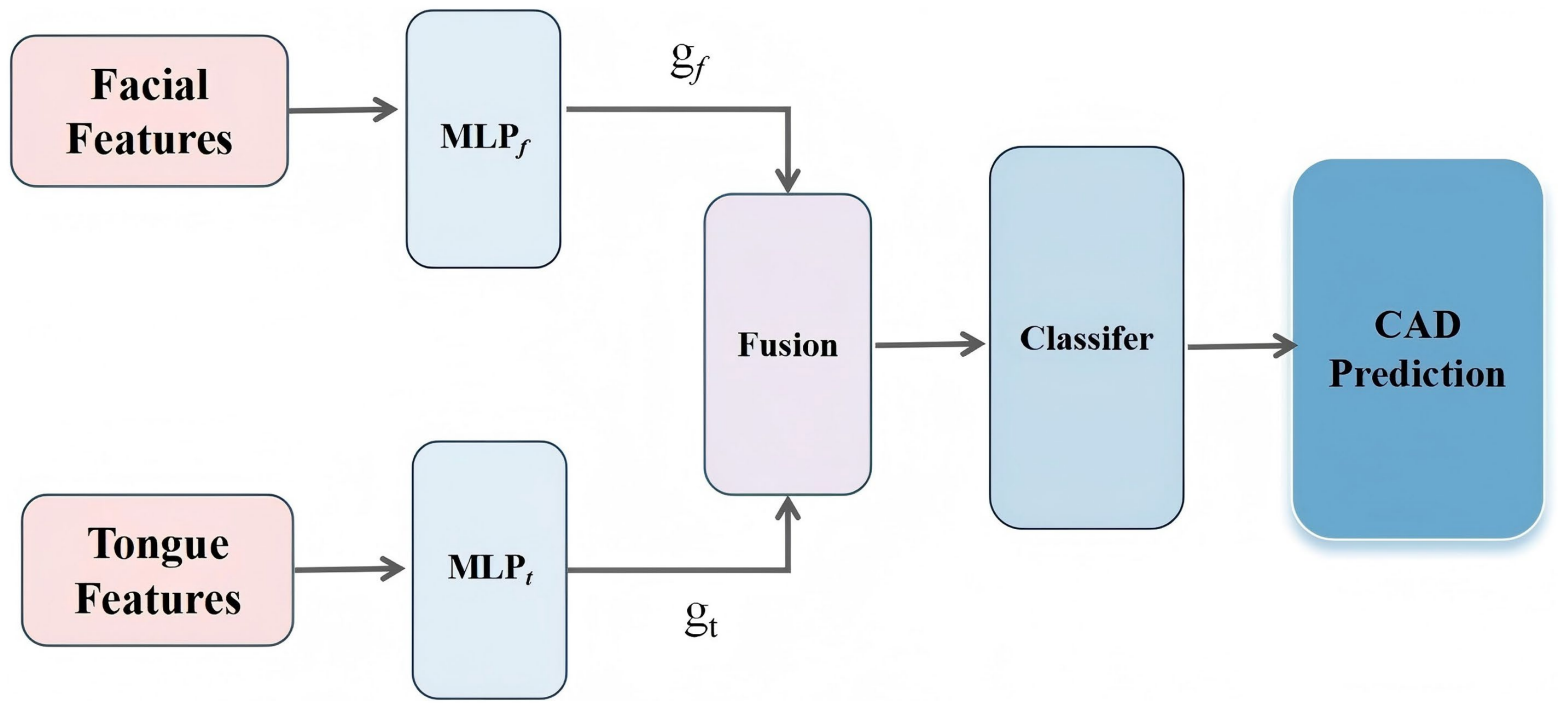
The workflow of the proposed methodology.

To effectively integrate and dynamically balance the contributions of facial and tongue features in the diagnosis of CAD, thereby improving both the predictive performance and interpretability of the model, we designed a dual-branch gated neural network, referred to as the Feature-Gated Classifier (FGC), as illustrated in Figure 5. The model comprises four main components: (1) input branches that receive two separate sets of features corresponding to facial and tongue modalities; (2) a gating module in which the concatenated facial and tongue features are passed through a fully connected layer followed by a Softmax activation function to generate two gating weights representing the attention assigned to each modality; (3) a feature fusion module that applies the gating weights to the respective features and concatenates them into a unified feature vector; and (4) a classifier module that feeds the fused vector into a feedforward neural network with 64 hidden units (using ReLU activation and a dropout rate of 0.35), followed by a Softmax output layer for binary CAD classification. This architecture enables the model to adaptively adjust the relative importance of facial and tongue features based on the feature distribution of each individual sample, thereby enhancing its discriminative power and generalization ability.

In this architecture, the gating module incorporates an attention-based Feature Gate Layer as a regulatory mechanism prior to deep feature fusion. This module is implemented using a lightweight fully connected neural network, which ensures both good trainability and computational efficiency. Specifically, let the facial feature vector be denoted as $F\in {\mathbb{R}}^{d}$ , and the tongue feature vector as $T\in {\mathbb{R}}^{d}$ . Each vector is first passed through a nonlinear transformation to generate modality-specific attention weights (Equation 1):


$$g_{f}=\sigma ({\mathrm{MLP}}_{f}(F)),g_{t}=\sigma ({\mathrm{MLP}}_{t}(T))$$ (1)

where σ(.) denotes the Sigmoid activation function, and ${\mathrm{MLP}}_{-}f$ and ${\mathrm{MLP}}_{\_}t$ represent two-layer multilayer perceptrons applied to the facial and tongue features, respectively. The outputs $g_{\_}f$ and $g_{\_}t$ correspond to the attention weights assigned to the facial and tongue modalities.

The fused feature vector H is then defined as (Equation 2):


$$H=g_{f}\cdot F+g_{t}\cdot T$$ (2)

This fused vector 𝐻 is subsequently fed into the downstream classifier to predict CAD risk.

Through this mechanism, the model achieves dynamic weighted fusion of multimodal features from the tongue and face, enabling adaptive modeling of inter-individual variability. Moreover, the gating structure provides explicit and quantifiable attention weights, which can be leveraged for downstream interpretability analyses—such as examining gating weight distributions or evaluating feature importance—thus enhancing the model’s practical utility and reliability in clinical settings.

#### 2.6.2 Feature set construction and preprocessing

In this study, a dataset comprising tongue and facial image features was used for modeling and analysis. Each sample was labeled to indicate whether the subject was diagnosed with CAD. After loading the dataset, the label column (“Type”) was separated from the feature data. Based on clinical expertise and image processing techniques, all variables were categorized into four subsets according to their source and type: (1) facial color and texture features (e.g., F_RGB, F_contrast), (2) deep facial features (F_PC), (3) tongue color and texture features (e.g., T_RGB, T_energy), and (4) deep tongue features (T_PC). This grouping strategy was designed to represent both low-level and high-level information extracted from the images.

#### 2.6.3 Data partitioning and model evaluation strategy

To ensure rigorous and unbiased model evaluation, we employed a nested cross-validation framework with three distinct stages: an outer split for model selection and internal validation, and an independent external test set for final generalization assessment.

The primary dataset (n = 737), collected from Dongzhimen Hospital Tongzhou Branch (337 CAD, 400 non-CAD), was first divided into two parts via stratified sampling at an 80:20 ratio using random_state = 121: ① Trainval set (*n* = 590): used for model training and hyperparameter optimization; ② Internal test set (*n* = 147): reserved exclusively for internal performance evaluation and kept completely unseen during the training process. ③ Within the Trainval set, a second stratified split (85:15) was performed to form: Training subset (*n* = 501): used to train the FeatureGatedClassifier; ④Validation subset (*n* = 89): used for early stopping, hyperparameter tuning, and calibration of prediction probabilities (via Platt scaling). All preprocessing steps—including feature grouping, standardization (StandardScaler), and dimensionality reduction (PCA)—were fitted only on the training subset and then applied consistently to both the validation and internal test sets to prevent data leakage.

After model training and selection, the best-performing model and its associated calibrator were saved. The final model was evaluated on the internal test dataset. This partitioning strategy ensured strict separation between training, validation, and testing phases, enabling reliable estimation of model performance and robust assessment of generalizability across institutions.

#### 2.6.4 Model training and performance evaluation

The CAD diagnostic model in this study was implemented using the PyTorch deep learning framework. The architecture comprises a dual-branch gated fusion network followed by a classification head. Specifically, the gating module takes facial and tongue feature vectors as parallel inputs and generates modality-specific attention weights through lightweight multilayer perceptrons with Sigmoid activation. These weights are normalized via Softmax to enable adaptive, sample-wise fusion of the two modalities. The subsequent classifier consists of two fully connected hidden layers (each with 256 units), with each layer followed sequentially by batch normalization (BatchNorm), ReLU activation, and Dropout regularization (dropout rate = 0.35). The final output layer employs a Softmax function to produce probabilistic predictions for the binary classification task (CAD vs. non-CAD). To address the mild class imbalance in the development cohort (337 CAD cases vs. 400 non-CAD controls), class weights were automatically computed using scikit-learn’s compute_class_weight (‘balanced’) and incorporated into the cross-entropy loss function, thereby enhancing the model’s sensitivity to the minority class.

During training, mini-batch stochastic gradient descent was performed with a batch size of 32, and the model was trained for up to 200 epochs. For each experimental run, the training set was partitioned into training and validation subsets at an 85:15 ratio (val_ratio = 0.15), with the validation subset used to monitor generalization performance. The Adam optimizer was employed with an initial learning rate of 1 × 10^−3^ and a weight decay of 1 × 10^−5^ to further mitigate overfitting. A dynamic learning rate scheduling strategy, ReduceLROnPlateau (factor = 0.5, patience = 6), was applied to automatically reduce the learning rate based on the validation AUC. Additionally, an early stopping mechanism was implemented: training was terminated if the validation loss failed to improve for 25 consecutive epochs (patience = 25), thus preventing overfitting and improving model robustness. All model parameters were updated automatically via backpropagation.

#### 2.6.5 Performance evaluation metrics

During the testing phase, the model’s performance was comprehensively assessed using multiple metrics (Gorur et al., 2018; Gorur et al., 2019), including accuracy, area under the receiver operating characteristic curve (AUC), F1 score, recall (sensitivity), and precision (positive predictive value, PPV) (Equations 3–6). In this context, true positives (TP) correspond to correctly identified CAD cases, true negatives (TN) to correctly classified non-CAD controls, false positives (FP) to non-CAD cases erroneously classified as CAD, and false negatives (FN) to CAD cases incorrectly labeled as non-CAD. This multi-dimensional evaluation framework enables balanced assessment of both overall discriminative ability and class-specific performance, ensuring the model’s reliability and practical applicability in clinical diagnostic settings.


$$\text{Accuracy}=\frac{\mathrm{TP}+\mathrm{TN}}{\mathrm{TP}+\mathrm{TN}+\mathrm{FP}+\mathrm{FN}}$$ (3)

$$\text{Recall}=\frac{\mathrm{TP}}{\mathrm{TP}+\mathrm{FN}}$$ (4)

$$\text{Precision}=\frac{\mathrm{TP}}{\mathrm{TP}+\mathrm{FP}}$$ (5)

$$F1\;\text{Score}=\frac{2\mathrm{TP}}{2\mathrm{TP}+\mathrm{FP}+\mathrm{FN}}$$ (6)

### 2.7 Validation on new testing dataset

To further evaluate the generalization capability of the proposed TF_FGC model, we established an independent external validation cohort by collecting tongue and facial images from 100 patients with CAD and 100 age- and sex-matched non-CAD controls at Dongzhimen Hospital (Dongcheng Branch). All participants underwent standardized acquisition of high-resolution facial and tongue images under controlled illumination and fixed posture conditions. This external test set was entirely independent of the model development dataset and was used to assess the model’s robustness and transferability in real-world clinical settings.

The TF_FGC model trained on the development set is directly applied to the external test set without any retraining or fine-tuning. The model outputs are used to calculate predicted probabilities for CAD, and the model’s generalization ability is evaluated using standard classification performance metrics (accuracy, recall, precision, F1 score, AUC, etc.). Calibration performance was further evaluated using the Brier score, calibration intercept, and slope. To improve the reliability of predicted probabilities, Platt scaling was applied prior to external testing.

To better reflect real-world clinical application scenarios, positive and negative predictive values (PPV and NPV) were reweighted using Bayes’ theorem under three realistic disease prevalence settings (5, 10, and 15%), corresponding to community screening, general outpatient, and cardiology referral populations, respectively. Finally, decision curve analysis (DCA) was performed to quantify the net clinical benefit across a range of probability thresholds (0.1–0.6), and the results were compared with those of the CAD Consortium Basic model as well as the “treat-all” and “treat-none” strategies.

### 2.8 Clinical baseline model construction

To provide clinical benchmark models, two types of baseline models were established on the external test set (n = 200, 100 CAD patients and 100 non-CAD controls): a physician-based visual baseline (Visual baseline) and a demographic-based logistic regression model (Age + Gender).

#### 2.8.1 Visual baseline based on facial and tongue features

The visual baseline was constructed as follows: three board-certified clinicians independently reviewed standardized facial and tongue photographs of all participants. Based on literature evidence, clinical experience, and preliminary model results, five key visual features associated with CAD were selected for binary annotation: tongue color, tongue texture, tongue contrast, facial color, and facial texture. Each feature was labeled as abnormal (=1) or normal (=0). A consensus label for each feature was determined using the majority rule (≥2 clinicians rated as abnormal). The total number of abnormal features (0–5) was then calculated as a risk score for CAD. Participants with ≥3 abnormal features were classified as CAD-positive (label = 1), and those with fewer than 3 abnormal features were classified as CAD-negative (label = 0).

#### 2.8.2 Demographic-based logistic regression model

A logistic regression model was constructed using age (continuous variable, in years) and gender (female = 0, male = 1) as independent variables, with CAD diagnosis as the dependent variable. The model outputs predicted probabilities for CAD, which can be thresholded to simulate different clinical scenarios. For community screening (prevalence ~5%, rule-out), a high-sensitivity threshold was selected to minimize missed diagnoses. For cardiology triage (prevalence ~30%, rule-in), a high-specificity threshold was chosen to reduce false-positive referrals.

### 2.9 Model interpretability analysis

To improve the transparency and clinical interpretability of the proposed TF_FGC model, we performed a three-level interpretability analysis using the external test set: global feature importance, individual decision path, and local feature dependence.

#### 2.9.1 Global feature importance (SHAP)

We applied the SHAP method to quantify each feature’s contribution to model prediction (Lundberg and Lee, 2017; Khorram et al., 2021). Using the TreeExplainer algorithm, SHAp values were computed for all samples in the external test set. Four feature groups were analyzed separately: facial color–texture, tongue color–texture, facial depth, and tongue depth features. Mean absolute SHAP values were used to rank feature importance, and SHAP summary plots were generated to visualize both contribution magnitude and direction.

#### 2.9.2 Individual decision path analysis

To visualize the model’s reasoning at the individual level, we generated SHAP decision plots for representative true-positive and true-negative cases. Only clinically interpretable facial and tongue color–texture features were included. The plots illustrated how each feature cumulatively influenced the prediction probability toward or away from CAD, revealing dominant contributors in each case (Nazim et al., 2025).

#### 2.9.3 Local feature dependence

To examine the nonlinear effects of key features on CAD risk, we plotted Partial Dependence Plots (PDPs) and Individual Conditional Expectation (ICE) curves. Six representative features (F_RGB_B, F_RGB_R, F_homogeneity, F_contrast, T_homogeneity, T_RGB_R) were analyzed. PDPs described the overall marginal effect, while ICE curves showed individual variations, allowing assessment of feature consistency and model generalizability.

## 3 Results

### 3.1 Performance evaluation of deep feature extraction based on MDFA-Swin and comparison with benchmark vision models

**Figure 6:**
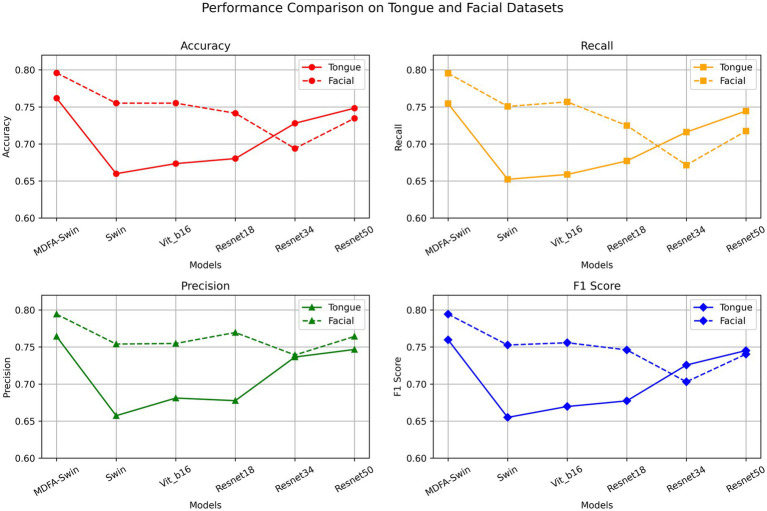
Validation performance of the MDFA-Swin model and baseline models on the tongue and facial image datasets.

**Figure 7:**
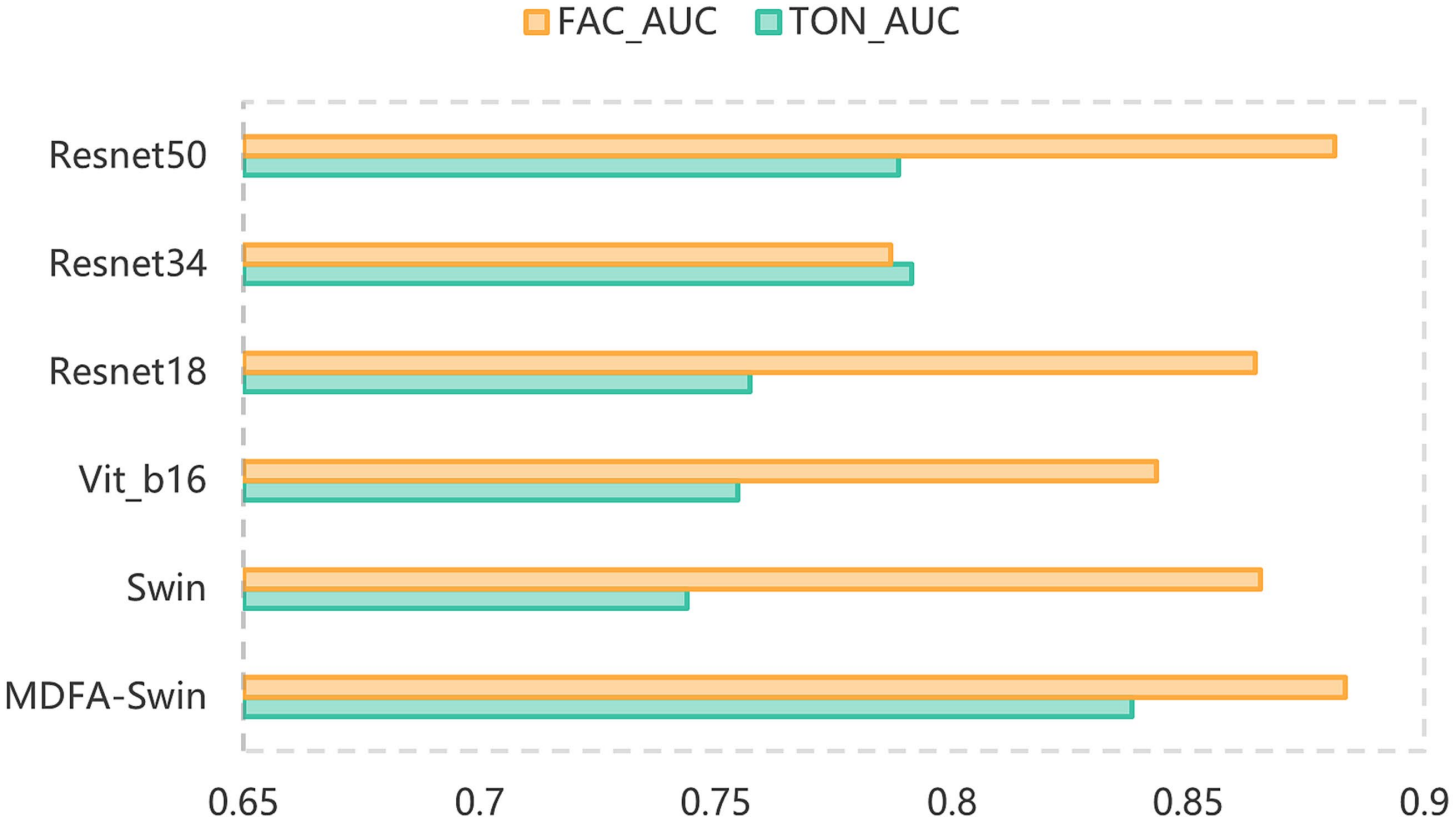
AUC comparison of the MDFA-Swin model and baseline models on the tongue and facial image datasets.

To verify the effectiveness of the proposed MDFA-Swin model in extracting deep features from tongue and facial images, we conducted a systematic comparison with several mainstream visual models, including ViT-B/16, Swin-Tiny, Swin-Small, ResNet18, ResNet34, and ResNet50. All models were trained and validated using the same dataset and training protocols, and their classification performance was evaluated separately on unimodal tasks involving tongue and facial images. As shown in Figures 6, 7, MDFA-Swin achieved the best performance across multiple evaluation metrics—including Accuracy, Recall, Precision, F1 Score, and AUC—significantly outperforming all baseline models. This demonstrates the model’s strong feature representation and discriminative capability in medical image analysis tasks. Specifically, on the tongue image validation set, the MDFA-Swin model achieved an Accuracy of 0.7619, Recall of 0.7546, Precision of 0.7643, F1 Score of 0.7598, and an AUC of 0.8381. On the facial image validation set, the model exhibited even stronger performance, with an Accuracy of 0.7959, Recall of 0.7955, Precision of 0.7943, F1 Score of 0.7944, and an AUC as high as 0.8832. These results clearly indicate that the improved MDFA-Swin model possesses superior deep feature modeling capabilities for both tongue and facial images. The introduced MDFA mechanism effectively enhances the model’s ability to perceive fine-grained structural variations and blurred boundary regions, thereby improving its capacity to express key pathological features in tongue and facial regions for more accurate CAD identification. Compared with traditional convolutional networks such as the ResNet series, MDFA-Swin leverages the cross-scale global modeling capability of the Transformer architecture to better integrate unstructured image information, exhibiting enhanced robustness and generalization ability. Collectively, these findings validate the applicability and advancement of MDFA-Swin as a backbone for deep feature extraction in the field of medical image analysis.

### 3.2 Visualization and dimensionality reduction of deep features

**Figure 8:**
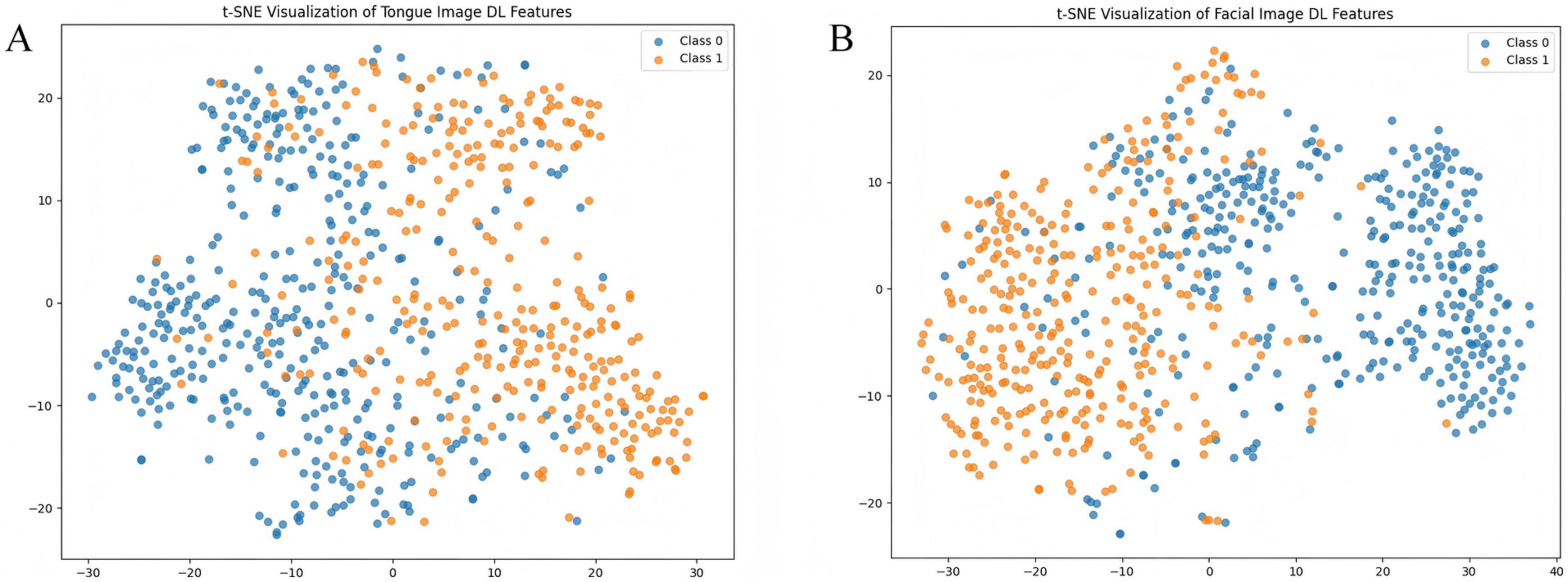
Visualization of tongue and facial deep features. **(A)** t-SNE plot of tongue image deep features. **(B)** t-SNE plot of facial image deep features.

Based on the optimal weights obtained from the trained MDFA-Swin model, high-dimensional semantic feature vectors (with a dimensionality of D = 768) were extracted from both tongue and facial images to represent the underlying semantic information of each individual in the deep feature space. To intuitively demonstrate the class separability of the extracted deep features, the t-distributed Stochastic Neighbor Embedding (t-SNE) algorithm (der Maaten and Hinton, 2008) was applied to perform nonlinear dimensionality reduction and map the features into a two-dimensional space. As shown in Figures 8A,B, the t-SNE visualizations reveal a clear separation between CAD and non-CAD samples for both tongue and facial features, indicating that the semantic features extracted by the MDFA-Swin model exhibit strong discriminative capability and class separability. Additionally, to reduce feature redundancy in subsequent fusion and classification modeling and to enhance computational efficiency, this study applies PCA (Aït-Sahalia and Xiu, 2019) for the linear dimensionality reduction of deep features in the following steps.

### 3.3 Validation results of five classifiers based on tongue and facial features

To The extraction of high-level semantic features from tongue and facial images using the MDFA-Swin model relies heavily on the black-box nature of deep neural networks, which limits clinical interpretability and fails to fully meet the requirements for diagnostic transparency and reliability in real-world applications. To address this issue, this study further incorporates interpretable traditional color and texture features derived from tongue and facial images as a complement to the deep semantic features. By enhancing the interpretability of the deep learning model and exploring the auxiliary diagnostic value of traditional image features in CAD identification, we conducted classification performance evaluations using these handcrafted features. Specifically, the traditional color and texture features were input into five widely used machine learning classifiers—Support Vector Machine (SVM), K-Nearest Neighbor (KNN), Multilayer Perceptron (MLP), Random Forest (RF), and eXtreme Gradient Boosting (XGB)—for CAD classification. The experimental results are summarized in Tables 2, 3. As shown in Table 2, for the classification task based on traditional tongue image features, the MLP model achieved the best overall performance across all metrics, with an Accuracy of 66.09%, Recall of 59.95%, Precision of 63.64%, F1 Score of 67.10%, and an AUC of 0.7038. While SVM and Logistic Regression (LR) achieved slightly higher Precision, their lower Recall led to inferior F1 scores and AUC compared to MLP. These findings suggest that the MLP exhibits a stronger capacity for capturing nonlinear color and texture patterns in tongue images, and that traditional features can support preliminary CAD risk screening to a certain extent, though their discriminative power remains limited.

**Table 2:** Performance comparison of traditional machine learning classifiers based on tongue color and texture feature set.

Models	Accuracy	Recall	Precision	F1 Score	AUC
SVM	0.6405	0.4365	0.6618	0.5249	0.6911
RF	0.6011	0.4718	0.5788	0.5197	0.6209
MLP	0.6609	0.5995	0.6364	0.6710	0.7038

**Table 3:** Performance comparison of traditional machine learning classifiers based on facial color and texture feature set.

Models	Accuracy	Recall	Precision	F1 Score	AUC
SVM	0.722	0.647	0.715	0.677	0.792
RF	0.716	0.647	0.705	0.673	0.777
MLP	0.727	0.658	0.723	0.686	0.798
LR	0.730	0.679	0.717	0.695	0.802
ET	0.716	0.667	0.695	0.680	0.769

In contrast, classification based on traditional facial image features yielded significantly better performance. As shown in Table 3, the Logistic Regression model outperformed all others across the five evaluation metrics, achieving an Accuracy of 72.98%, Recall of 67.92%, Precision of 71.73%, F1 Score of 69.54%, and an AUC of 0.8022—the highest among all models tested. MLP also demonstrated relatively stable and competitive performance. Overall, these results indicate that facial image features provide greater diagnostic value in CAD identification, likely due to more pronounced structural differences in skin color variation and texture distribution between CAD and non-CAD individuals.

### 3.4 Performance of the FGC model on the test set

To systematically evaluate the performance of the proposed FGC model in the intelligent diagnosis of CAD, and to verify the effectiveness of multimodal fusion between tongue and facial features, three models were constructed for comparison: (1) an FGC model based on fused tongue and facial features (TF_FGC); (2) an FGC model using only tongue features as input (T_FGC); and (3) an FGC model using only facial features as input (F_FGC). As shown in Table 4, the fusion model TF_FGC achieved the best performance across all evaluation metrics, with an accuracy of 0.872, recall of 0.897, precision of 0.836, F1-score of 0.865, and an AUC of 0.945. These results demonstrate that the proposed gating mechanism can automatically learn and adjust the relative importance of tongue and facial features, enabling effective information fusion and substantially improving the model’s discriminative ability for CAD.

**Table 4:** Comparison of classification performance of FGC model on verification set under different input data sets.

Models	Accuracy	Recall	Precision	F1 Score	AUC
TF _FGC	0.872	0.897	0.836	0.865	0.945
T_FGC	0.831	0.824	0.812	0.818	0.902
F_FGC	0.858	0.882	0.823	0.8148	0.935

In contrast, the unimodal models T_FGC and F_FGC performed less favorably, with accuracies of 0.831 and 0.858, and AUCs of 0.902 and 0.935, respectively. Although both unimodal models showed reasonable diagnostic capability, their accuracy and robustness were inferior to the fusion model due to limited information utilization. This finding indicates that tongue and facial features provide complementary diagnostic cues in CAD classification tasks. In summary, the experimental results confirm that: (1) the FGC model possesses strong multimodal feature fusion capabilities; and (2) both tongue and facial features contribute valuable and distinct information in CAD diagnosis, and their integration significantly enhances model accuracy and stability. Therefore, the FGC model incorporating a multidimensional feature fusion strategy and a gating mechanism represents the optimal CAD recognition solution in this study.

### 3.5 Performance of the TF_FGC model in external validation

#### 3.5.1 Overall performance

**Figure 9:**
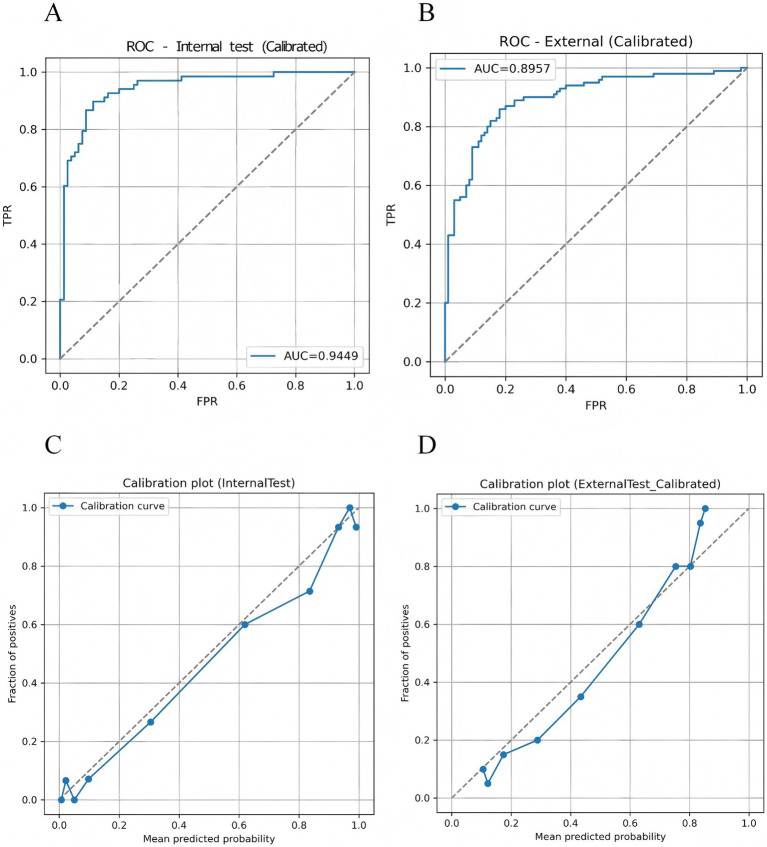
Discriminative performance and probability calibration of the TF_FGC model. **(A)** Receiver operating characteristic (ROC) curve for the internal validation set. **(B)** ROC curve for the independent external validation set. **(C)** Calibration plot for the internal validation set. **(D)** Calibration plot for the external validation set.

The proposed TF_FGC model demonstrated excellent discriminative performance, good calibration, and strong clinical applicability in both internal and external validation. As shown in Table 5 and Figure 9A, in the internal validation cohort, the model achieved an accuracy of 0.872, F1-score of 0.865 (95% CI: 0.800–0.920), recall of 0.897 (95% CI: 0.823–0.960), and precision of 0.836 (95% CI: 0.747–0.917). The AUC reached 0.945 (95% CI, DeLong: 0.905–0.985; Bootstrap: 0.904–0.977), indicating outstanding discriminative ability. The Brier score was 0.091, with a calibration intercept of −0.259 and a slope of 0.927, suggesting good overall model fit after Platt scaling calibration (Figure 9C).

**Table 5:** Model performance on internal and external test sets.

Metric	Internal test	External test
AUC (95% CI: DeLong; Bootstrap)	0.945 (0.905–0.985; 0.904–0.977)	0.896 (0.850–0.941; 0.848–0.939)
F1 (95% CI, bootstrap)	0.865 (0.800–0.920)	0.843 (0.788–0.894)
Recall (95% CI, bootstrap)	0.897 (0.823–0.960)	0.860 (0.789–0.929)
Precision (95% CI, bootstrap)	0.836 (0.747–0.917)	0.827 (0.750–0.894)
ACC	0.872	0.825
Brier score	0.091	0.130
Calibration intercept	−0.259	−0.035
Calibration slope	0.927	1.350

To further evaluate the model’s generalizability, an external validation was performed on an independent test cohort from another clinical center, comprising 200 participants (100 CAD patients and 100 non-CAD controls). As shown in Table 5 and Figure 9B, the model correctly identified 86 CAD and 79 non-CAD cases, achieving an accuracy of 0.825, recall of 0.860, precision of 0.827, F1-score of 0.843, and an AUC of 0.896 (95% CI: 0.850–0.941). The Brier score was 0.130, with a calibration intercept of −0.035 and a slope of 1.350, indicating acceptable calibration consistency across centers and good robustness and transferability (Figure 9D).

To explicitly examine site-level stability, a per-center performance analysis was conducted. The TF_FGC model achieved consistent discrimination across both acquisition sites: in Site A (internal validation), the AUC was 0.945 (95% CI: 0.905–0.985) and AUPRC 0.934; in Site B (external validation), the AUC was 0.896 (95% CI: 0.850–0.941) and AUPRC 0.902. Calibration slopes (0.927 vs. 1.350) and intercepts (−0.259 vs. –0.035) showed no substantial drift, and Brier scores (0.091 vs. 0.130) remained within an acceptable range. These findings indicate that the model’s predictive performance was not affected by site-specific factors, supporting its robustness to center heterogeneity (see Supplementary Table S1).

**Figure 10:**
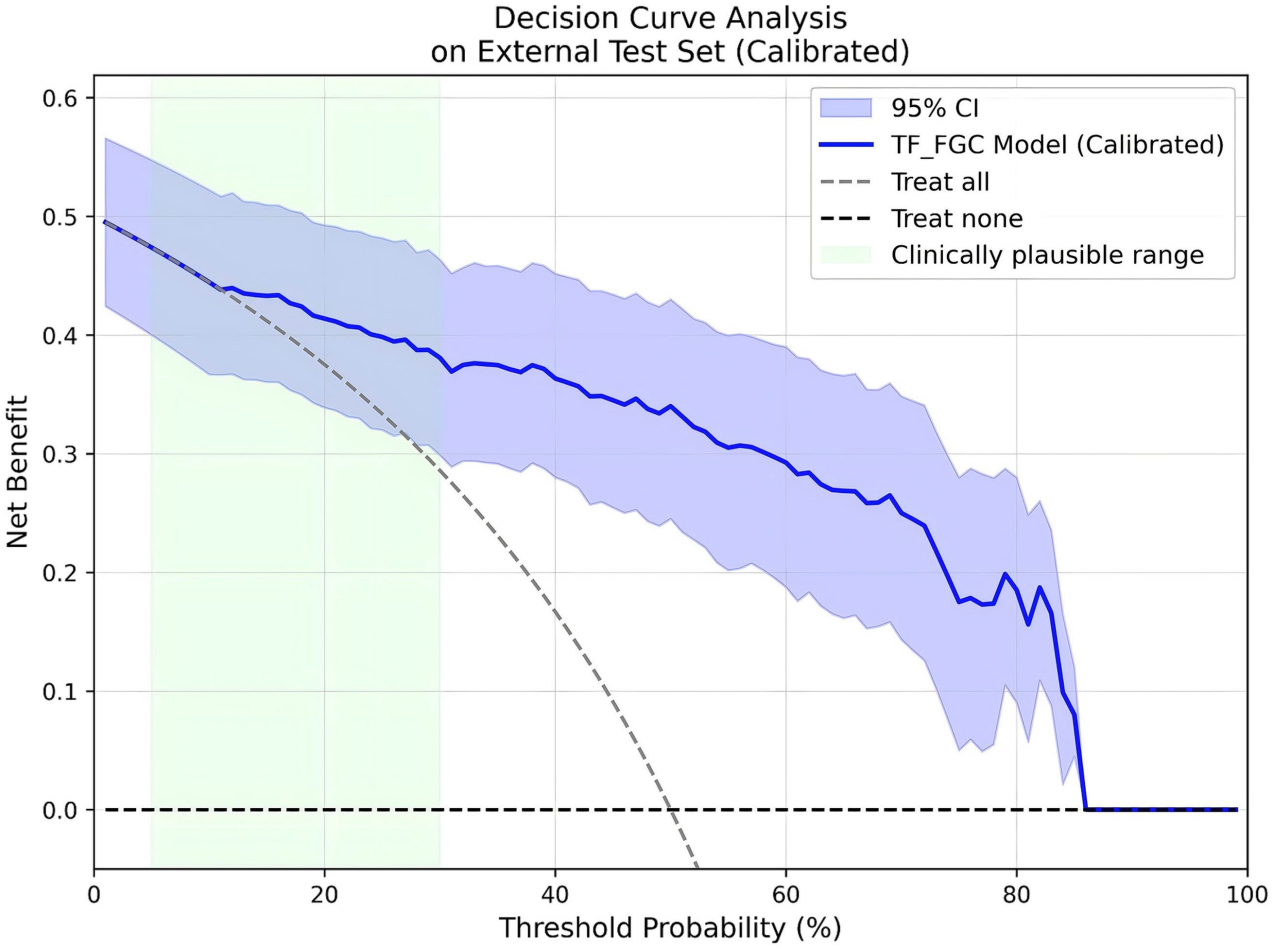
Decision curve analysis (DCA) results of the TF_FGC model.

Considering the model’s potential value in real-world screening scenarios, prevalence-weighted PPV and NPV were further estimated under hypothetical disease prevalences of 5, 10, and 15%. As summarized in Table 6, the PPVs (95% CI) were 0.201 (0.146–0.301), 0.347 (0.265–0.476), and 0.457 (0.364–0.591), respectively, while the NPVs (95% CI) remained consistently high at 0.991 (0.986–0.995), 0.981 (0.972–0.990), and 0.971 (0.956–0.984). These findings indicate that the TF_FGC model maintains excellent exclusion capability in low-prevalence populations, supporting its potential use as an auxiliary tool for preliminary screening of obstructive CAD risk. Furthermore, DCA (Figure 10) demonstrated that, within the clinically relevant probability threshold range of 0.1–0.6, the TF_FGC model provided consistently higher net benefit than the “treat-all” or “treat-none” strategies, confirming its practical utility in clinical decision-making.

**Table 6:** Estimated PPV and NPV under assumed disease prevalences of 5, 10, and 15%.

Prevalence	PPV (95% CI)	NPV (95% CI)
5%	0.201 (0.146–0.301)	0.991 (0.986–0.995)
10%	0.347 (0.265–0.476)	0.981 (0.972–0.990)
15%	0.457 (0.364–0.591)	0.971 (0.956–0.984)

#### 3.5.2 Robustness to common corruptions

To evaluate the robustness of the TF_FGC model under varying imaging conditions, we introduced three types of controlled perturbations to the external test set: brightness jitter (±20%), contrast jitter (±20%), and Gaussian noise (*σ* = 0.02). These perturbations were designed to simulate potential variations in illumination, device characteristics, and environmental interference commonly encountered in clinical image acquisition.

As summarized in Table 7, under the original (unperturbed) external test set, the model achieved an accuracy of 0.825, F1-score of 0.843, AUC of 0.896, AUPRC of 0.902, recall of 0.860, and precision of 0.827. After applying brightness, contrast, and Gaussian noise perturbations, only minor performance fluctuations were observed. The AUC values remained within 0.893–0.900, while accuracy, F1-score, and recall were largely unchanged. Notably, the 95% bootstrap confidence intervals of the AUCs substantially overlapped with those of the unperturbed results. These findings indicate that the TF_FGC model exhibits strong robustness to common image perturbations. Its discriminative performance was not significantly affected by variations in lighting, contrast, or noise levels, underscoring the model’s stability and generalizability across diverse imaging conditions in multi-center real-world applications.

**Table 7:** Model robustness under common corruptions.

Perturbation	Accuracy	F1-score	AUC	AUPRC	ΔAUC (vs. Clean)	Recall	Precision
Clean	0.825	0.843	0.896	0.902	—	0.860	0.827
Brightness (±20%)	0.830	0.835	0.893	0.899	−0.003	0.860	0.811
Contrast (±20%)	0.825	0.833	0.900	0.906	+0.004	0.870	0.798
Gaussian noise (σ = 0.02)	0.830	0.830	0.830	0.830	0.830	0.830	0.830

#### 3.5.3 Subgroup-specific threshold optimization

To further evaluate potential performance differences of the model across key clinical subgroups and explore simple mitigation strategies, we conducted subgroup-specific threshold tuning on the external validation cohort. The subgroup variables included hypertension, diabetes, and gender, resulting in six primary subgroups (Table 8). Among hypertensive patients (n = 106, AUC = 0.873), the model achieved both sensitivity and specificity of 82.1% at the default threshold of 0.5. Raising the threshold to the Youden optimal value of 0.57 increased specificity to 87.2% while only slightly reducing sensitivity to 79.1%, effectively lowering the false-positive rate. In diabetic patients (n = 52, AUC = 0.891), sensitivity and specificity were 89.7 and 91.3% at the default threshold, with minimal change after Youden threshold adjustment, indicating robust model performance despite potential interference from diabetes-related tongue coating. Gender subgroup analysis showed AUCs of 0.883 for males and 0.916 for females, with sensitivity and specificity close to overall levels and no clinically significant differences. To provide a more comprehensive evaluation of subgroup performance, we report PPV, NPV, and calibration slope/intercept for each subgroup in the Supplementary Table S2.

**Table 8:** Subgroup performance based on Youden-optimal decision thresholds.

Subgroup	N	AUC	Youden threshold	Sensitivity	Specificity
Hypertension	106	0.873	0.57	0.791	0.872
Non-hypertensive	94	0.934	0.509	0.939	0.820
Diabetes	52	0.891	0.509	0.897	0.913
Non-diabetes	148	0.901	0.392	0.887	0.753
Male	110	0.883	0.477	0.821	0.815
Female	90	0.916	0.517	0.932	0.826

Overall, the subgroup-specific threshold tuning demonstrates that, even without retraining the model, simple adjustment of decision thresholds for different clinical subgroups can effectively optimize performance, reduce false positives, or increase sensitivity, supporting safe and interpretable application of the model across diverse patient populations.

### 3.6 Performance of clinical baseline models

The results of the visual baseline are presented in Table 9. The model achieved an overall accuracy of 67.0%, sensitivity of 68.0%, specificity of 66.0%, PPV of 66.7%, NPV of 67.4%, F1 score of 0.673, and an approximate AUC of 0.670. As the visual baseline outputs are fixed binary classifications, its performance cannot be adjusted via threshold modification, and it therefore serves as a single reference point. The demographic logistic regression model was constructed with CAD diagnosis as the dependent variable and age and sex as independent variables. As shown in Table 10, using the default threshold of 0.5, the model achieved a sensitivity of 0.51 and a specificity of 0.53, indicating limited predictive performance. To simulate typical clinical application scenarios, two operational thresholds were further defined: Community screening (rule-out): a high-sensitivity threshold (≥90%) was selected to minimize missed diagnoses, corresponding to a threshold of 0.4584, sensitivity of 0.92, specificity of 0.12, PPV of 0.51, and NPV of 0.60; Cardiology triage (rule-in): a high-specificity threshold (≥85%) was selected to reduce false-positive referrals, corresponding to a threshold of 0.5349, sensitivity of 0.19, specificity of 0.85, PPV of 0.56, and NPV of 0.51.

**Table 9:** Diagnostic performance of the physician visual baseline on the external test set.

Metric	Value
Accuracy	0.670
Sensitivity	0.680
Specificity	0.660
PPV	0.667
NPV	0.674
F1 Score	0.673

**Table 10:** Diagnostic performance of the age-sex logistic model across clinical scenarios.

Scenario	Threshold	Sensitivity	Specificity	PPV	NPV
Community screening (rule-out, high sensitivity)	≥0.4584	0.92	0.12	0.511	0.600
Cardiology triage (rule-in, high specificity)	≥0.5349	0.19	0.85	0.559	0.512

Overall, the physician visual baseline slightly outperformed the demographic model in terms of accuracy, PPV, and NPV, but it lacked the flexibility of threshold adjustment. Conversely, the demographic model could adapt to different clinical scenarios through threshold setting, yet its overall discriminative ability remained limited. These baseline results provide reference points for performance comparison and threshold selection in subsequent multimodal imaging models. In contrast, the proposed TF_FGC model demonstrated markedly superior performance across all key metrics compared with both clinical baselines, while retaining the flexibility to adjust thresholds for different clinical scenarios. For example, in the external test set, the TF_FGC model achieved an accuracy of 0.825, sensitivity of 0.86, specificity of 0.79, PPV of 0.827, NPV of 0.843, and an AUC of 0.896. These results indicate that the TF_FGC model not only exhibits stronger overall discriminative ability but also provides reliable and adjustable predictions across various clinical contexts, highlighting its clinical utility as a non-invasive, interpretable, and practically applicable tool for auxiliary CAD screening and triage.

### 3.7 Model interpretability analysis based on the external test set

#### 3.7.1 Global feature importance (SHAP analysis)

**Figure 11:**
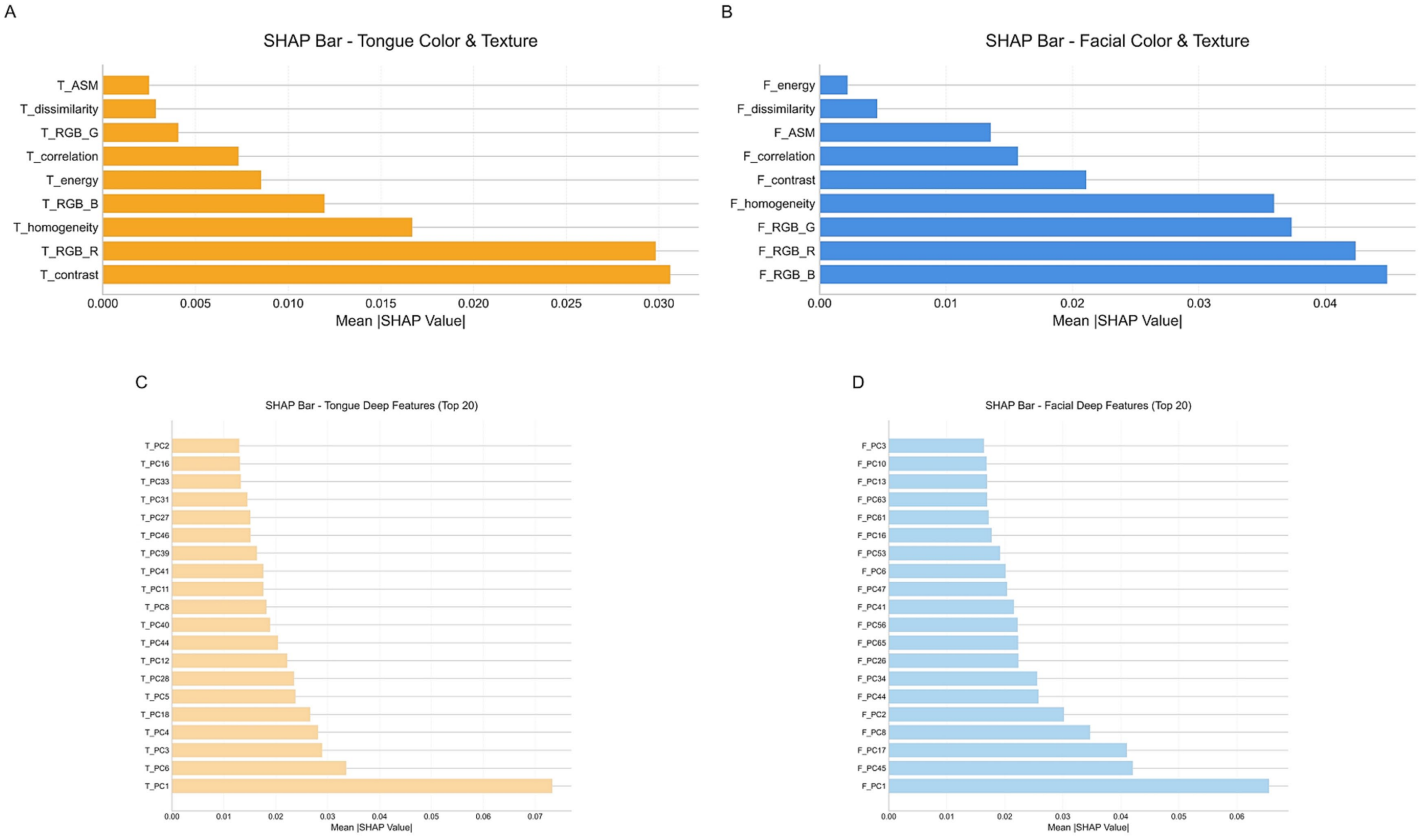
SHA*p* value distribution for four feature groups. **(A)** Tongue color and texture features. **(B)** Facial color and texture features. **(C)** Tongue deep features. **(D)** Facial deep features.

To enhance the overall interpretability of the model, we employed the SHAP method to analyze the final multimodal fusion model. Figure 11 presents bar plots of SHAP value distributions for four feature groups—facial/tongue color–texture features and facial/tongue depth features—in the external test set. As shown, among tongue color–texture features, T_contrast and T_RGB_R exhibited the highest mean SHAP values, serving as the core contributors, followed by T_homogeneity and T_RGB_B. Within tongue depth features, T_PC1 showed the largest mean SHAP value, indicating the strongest influence on model output. For facial color–texture features, F_RGB_B, F_RGB_R, F_RGB_G, and F_homogeneity contributed most significantly, while in facial depth features, F_PC1 exerted the greatest control over model predictions.

**Figure 12:**
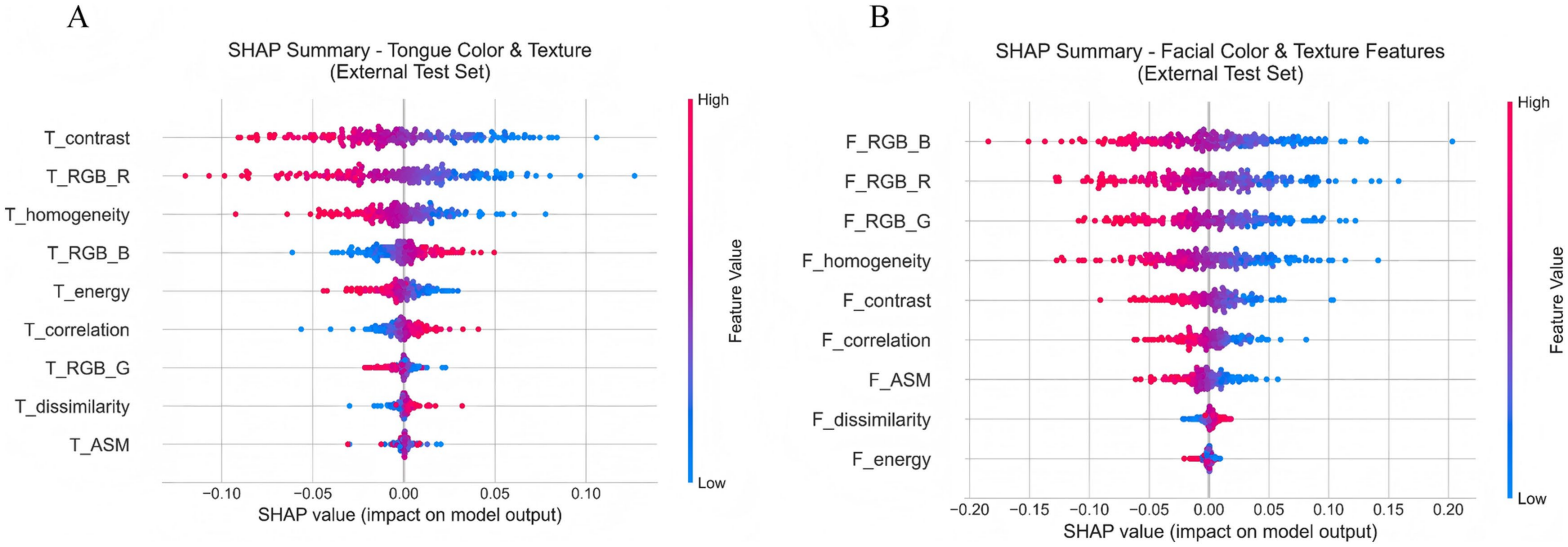
**(A)** SHAP scatter plot of tongue color and texture features. **(B)** SHAP scatter plot of facial color and texture features.

Further analysis of Figure 12A shows the SHAP scatter distributions of tongue color–texture features. T_contrast, T_RGB_R, and T_homogeneity displayed a clear bimodal trend: low feature values corresponded to negative SHAP values (blue), whereas high feature values corresponded to positive SHAP values (red). This pattern indicates that lower values of these features were associated with a higher likelihood of CAD prediction. Similarly, Figure 12B illustrates that facial color–texture features (F_RGB_B, F_RGB_R, F_RGB_G, and F_homogeneity) exhibited comparable negative trends. When these feature values were low—such as bluish complexion or coarse skin texture—the corresponding SHAP values were strongly negative, suggesting that the model identified these traits as high-risk indicators for CAD. In contrast, F_energy and F_dissimilarity displayed more concentrated and lower-magnitude SHAP distributions, indicating relatively limited influence on model decisions. Overall, features related to pale, dark, or uneven visual characteristics in both the tongue and facial images tended to show negative SHAP values, implying that these visual cues play an important role in the model’s identification of CAD risk.

#### 3.7.2 SHAP decision path analysis of typical cases

**Figure 13:**
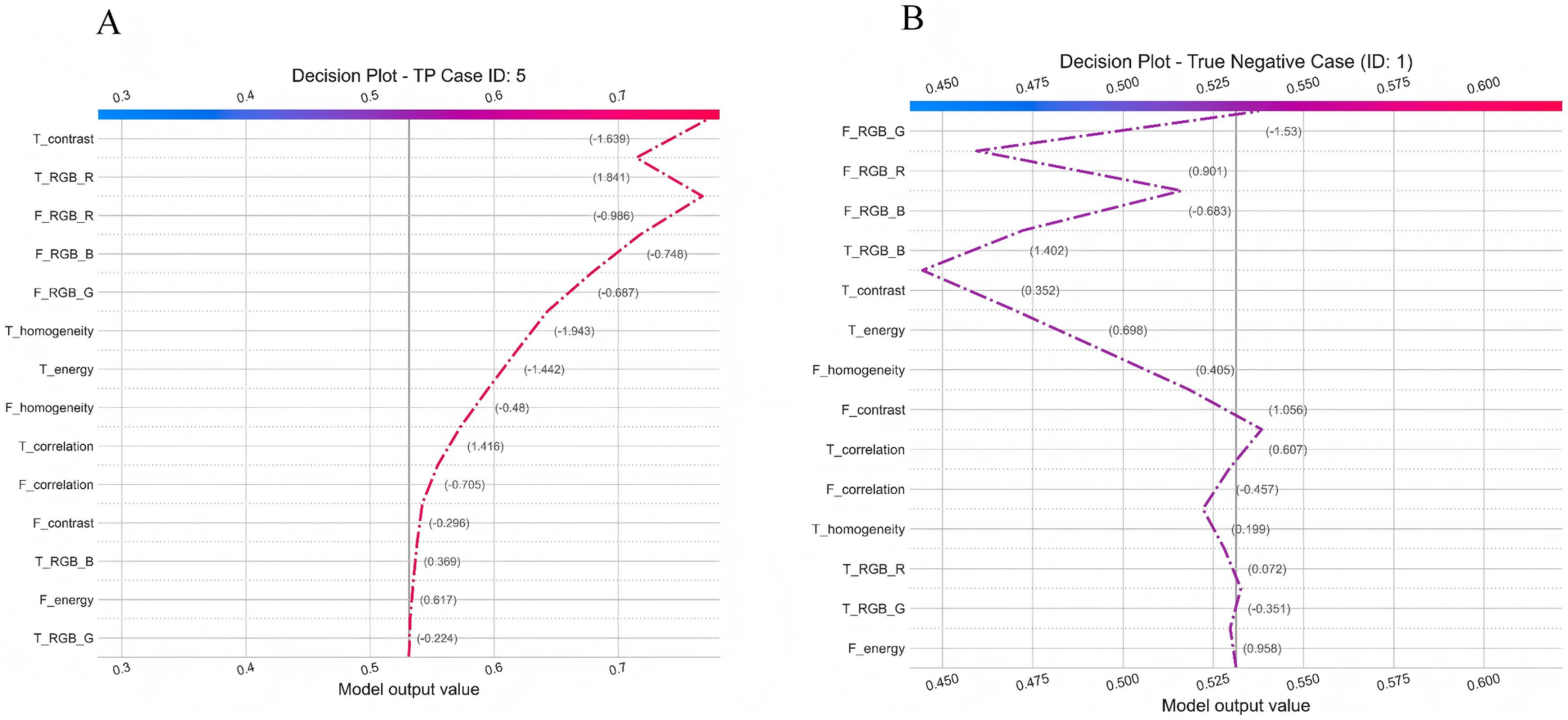
Individual decision Pathway Analysis of the TF_FGC Model. **(A)** Decision plot for a true positive case (ID: 5). **(B)** Decision plot for a true negative case (ID: 1).

To move beyond global feature importance and enhance the clinical interpretability of the model, we further performed explainability analysis using the SHAP method to investigate the model’s decision-making mechanisms in depth. Figure 13 illustrates the SHAP decision paths of two representative individuals, where only clinically interpretable facial and tongue color and texture features were retained for analysis. As shown in Figure 13A, for a true-positive patient (ID: 5), the model’s initial prediction value was 0.50, which ultimately increased to approximately 0.72, indicating a strong tendency toward a positive (CAD) classification. This decision was mainly driven by tongue features, among which tongue redness (T_RGB_R) contributed the most (+1.841), suggesting a pronounced “heat pattern” according to traditional diagnostic interpretation. Meanwhile, the decreased homogeneity (T_homogeneity: −1.943) and lower contrast (T_contrast: −1.639) indicated disturbed surface texture on the tongue. Although facial color features (e.g., F_RGB_R: −0.986) had a negative contribution, implying a pale or dull facial tone, they were insufficient to counterbalance the dominant influence of tongue features. In contrast, for the negative patient (ID: 1), the model’s initial value was 0.525, which decreased to about 0.45, leading to a negative prediction. Here, the decision was primarily driven by facial features, with strong negative contributions from the green (F_RGB_G: −1.53) and red (F_RGB_R: −0.901) channels, reflecting a healthy, rosy complexion and sufficient qi and blood circulation. In terms of tongue features, the patient exhibited a thin, evenly distributed coating and clear texture, consistent with a balanced physiological state. These comparisons demonstrate that the model effectively integrates multimodal visual information to differentiate between pathological and healthy conditions. Moreover, its reasoning process aligns with the traditional Chinese medicine principle of *“holistic inspection and integrated diagnosis,”* suggesting both biomedical validity and interpretive consistency with traditional diagnostic thinking.

#### 3.7.3 Local dependence and individual effects of key features

**Figure 14:**
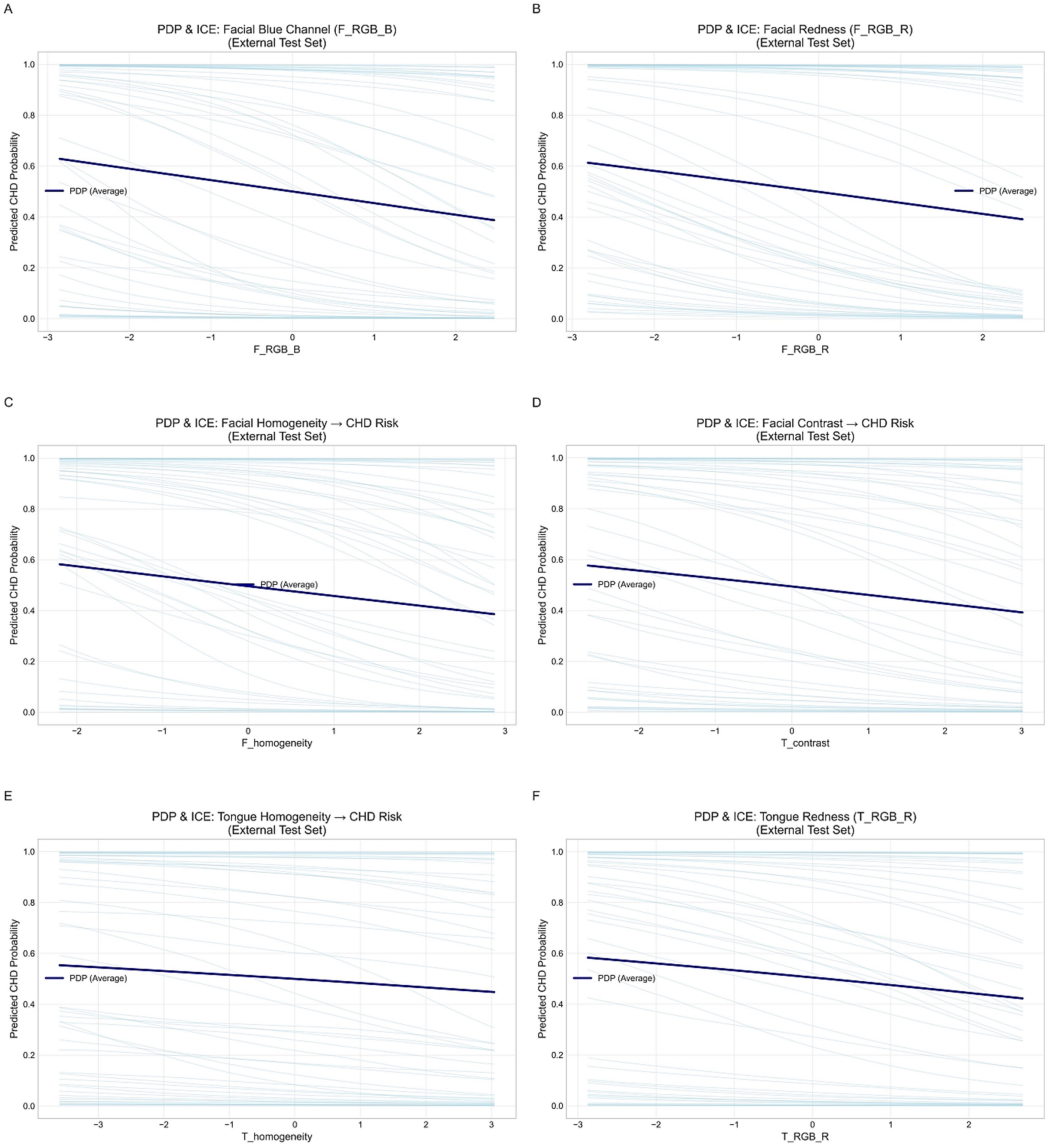
Partial dependence and individual conditional expectation (ICE) curves for key facial and tongue features in the external validation set. **(A)** Facial blue channel (F_RGB_B). **(B)** Facial red channel (F_RGB_R). **(C)** Facial homogeneity. **(D)** Tongue contrast. **(E)** Tongue homogeneity. **(F)** Tongue red channel (T_RGB_R).

To further elucidate the relationship between the imaging features learned by the model and the risk of CAD, we plotted PDPs and ICE curves for six key features based on the external validation set (Figure 14). The analyzed features included facial blue channel intensity (F_RGB_B), facial redness (F_RGB_R), facial homogeneity (F_homogeneity), facial contrast (F_contrast), tongue homogeneity (T_homogeneity), and tongue redness (T_RGB_R).

Overall, the PDP curves (dark blue lines) for all six features showed smooth and largely monotonic trends, while the ICE curves (light blue lines) demonstrated consistent individual-level behaviors. This consistency suggests that the model exhibits strong external generalizability and a low risk of overfitting. In particular, increases in facial and tongue redness (F_RGB_R and T_RGB_R) were associated with a decreased probability of CAD, implying that reduced redness may reflect microcirculatory insufficiency or hemodynamic abnormalities. Similarly, decreases in facial and tongue homogeneity were linked to elevated CAD risk, suggesting that uneven texture or color distribution may capture underlying circulatory disturbances. Additionally, facial blue channel intensity (F_RGB_B) and facial contrast (F_contrast) showed mild negative correlations with CAD risk, further supporting the stability and predictive relevance of facial color–texture features. In summary, the PDP and ICE analyses demonstrated physiologically plausible and directionally consistent associations between the identified features and CAD risk, thereby validating the interpretability and external reliability of the proposed model.

### 3.8 Comparison with existing studies

To comprehensively evaluate the advantages of the proposed model, we systematically compared its performance with recent studies on intelligent diagnosis of CAD based on tongue images, facial images, or clinical data (see Table 11).

**Table 11:** Comparison of CAD diagnostic models.

Study	Modality	Model (Backbone)	AUC	Ext. Test	Fusion	Interp.
Shen et al.	Facial images	CNN (Custom)	0.73	No	No	No
Khan et al.	Clinical data	1D-CNN (—)	0.769*	Yes	No	No
Cai-Yi Ma et al.	EMR data	ML (XGBoost)	0.701	Yes	No	Partial
Duan et al.	Tongue + Clinical	XGBoost (—)	0.786	No	Shallow (feature)	Partial
Zhang et al.	Facial + Clinical	DL/ML (ResNet-18)	0.852	Yes	Early fusion	No
Duan et al.	Tongue images	CNN/DL (ResNet-18/Swin-T)	0.83–0.86	No	No	No
Ours	Tongue + Facial	FGC (MDFA-Swin + Gate)	0.9134	Yes (0.9102)	Deep, adaptive (gate)	SHAP analysis

CNN, Convolutional Neural Network; EMR, Electronic Medical Records; ML, Machine Learning; XGBoost, Extreme Gradient Boosting; DL, Deep Learning; Swin-T, Swin Transformer (Tiny version); FGC, Feature Gated Combination; MDFA, Multi-scale Dilated Fusion Attention; AUC, Area Under the Receiver Operating Characteristic Curve; Ext. Test, External Test; Fusion, multimodal fusion strategy; Interp., Interpretability. *Accuracy for CAD class; AUC not reported.

Several studies have explored deep learning for CAD detection using facial or tongue images. Lin et al. (2020) developed a facial image-based model achieving a sensitivity of 0.80 and an AUC of 0.730. Khan Mamun and Elfouly (2023) used NHANES data with a 1D-CNN, reporting an accuracy of 76.9%. Ma C. Y. et al. (2024) predicted CAD risk using electronic medical records, achieving an AUC of 0.701. Duan et al. (2024b) combined tongue image features with clinical factors and XGBoost, reporting an accuracy of 0.760 and an AUC of 0.786. More recently, Zhang et al. (2025) proposed a multimodal model using facial images and clinical variables, achieving an AUC of 0.852. Duan et al. (2024a) and Duan et al. (2024b) applied deep learning (e.g., CNNs, lightweight networks) to tongue image analysis, reporting AUCs between 0.83 and 0.86.

Notably, most of these models rely on single-modality inputs or shallow fusion strategies, and few incorporate interpretable feature analyses. In contrast, our model achieves an AUC of 0.9134 on the validation set and 0.9102 on the independent test set, outperforming all cited studies. The advantages of this study are reflected in three aspects: (i) the MDFA-Swin backbone achieved a 2.1–3.5% improvement in AUC compared with standard Swin and ResNet models; (ii) the feature-gated classifier enabled adaptive fusion of tongue and facial features, outperforming unimodal models (T_FGC: 0.8943; F_FGC: 0.9057); and (iii) the integration of interpretable color/texture features, combined with SHAP analysis, identified key discriminative predictors (e.g., T_RGB_R, F_RGB_B), enhancing consistency with TCM clinical phenotypes.

## 4 Discussion

The development of artificial intelligence has opened new avenues for disease diagnosis, with deep learning demonstrating remarkable performance in medical imaging and computer-aided diagnosis. However, most current diagnostic models still rely on clinical data such as blood tests, imaging scans, and electrocardiograms, which are costly, complex to obtain, and thus unsuitable for primary healthcare settings and large-scale population screening—ultimately limiting their clinical applicability and widespread adoption. In contrast, tongue and facial features, as core components of “inspection” in TCM, offer a non-invasive, low-cost, and easily collectible source of diagnostic information. These characteristics make them well-suited for community-based screening, telemedicine, and home health monitoring. In this study, we integrated traditional TCM image features—such as color and texture from tongue and facial images—with high-level semantic representations extracted by the MDFA-Swin network to construct a multimodal, multi-scale fusion diagnostic framework. To enhance model interpretability, we conducted both internal attention distribution analysis and external explainability assessment.

Notably, MedSAM demonstrated outstanding performance in both tongue and facial image segmentation tasks. In particular, its zero-shot generalization capability significantly outperformed traditional methods, greatly reducing the need for manual annotation, alleviating labeling workload, and lowering associated costs. Furthermore, MedSAM substantially minimized human intervention, thereby exhibiting excellent segmentation performance and practical advantages in tongue and facial image analysis. To effectively capture the multi-scale and fine-grained pathological characteristics associated with CAD in tongue and facial images, this study introduced a MDFA module into the Swin Transformer architecture, specifically designed for the unique imaging properties of these modalities. Experimental results demonstrated that the MDFA-Swin model significantly outperformed ViT, ResNet, and the standard Swin Transformer in tongue and facial image classification tasks, exhibiting superior discriminative ability and heightened sensitivity to subtle TCM phenotypes—such as purplish tongue color and facial dullness. However, despite its strong performance in automatic feature learning and classification, the inherently “black-box” nature of deep neural networks limits their clinical interpretability. In contrast, TCM visual diagnosis emphasizes observable and quantifiable external manifestations—such as tongue color, texture, facial complexion, and skin texture distribution—which serve as key indicators in clinical decision-making. To bridge the gap between performance and interpretability, this study further integrated clinically interpretable features—including color and texture descriptors derived from classical TCM diagnostic theory—with the deep semantic representations learned by the model. These features provide explicit clinical semantics and complement the deep representations to enhance both transparency and reliability. Furthermore, through a gating mechanism, the proposed Tongue–Face Fusion Gated Classifier (TF_FGC) dynamically combined deep and interpretable features from tongue and facial modalities. The fusion model not only achieved superior diagnostic accuracy (AUC = 0.896 in external validation) but also, via SHAP analysis, revealed key decision-driving features such as T_contrast and F_homogeneity, aligning the predictive reasoning more closely with TCM diagnostic logic. Overall, the results demonstrate that the multimodal fusion of tongue and facial features markedly outperforms single-modality approaches, validating the effectiveness of the “Deep Learning + TCM Feature” paradigm. This framework achieves a balance between high diagnostic performance and clinical interpretability, offering a feasible pathway toward a noninvasive, low-cost, and trustworthy CAD diagnostic system suitable for community screening and home-based health monitoring.

In addition, through interpretability analysis of the model, we found that CAD patients showed overall lower values in facial features such as F_RGB_R, F_RGB_G, and F_RGB_B, suggesting a lack of redness and brightness in the skin. This aligns with the TCM concept of “facial dullness” (mian se hui an), which is thought to reflect impaired facial microcirculation due to weakened cardiac function or blood stasis in the coronary vessels. This phenomenon is supported by previous studies, which have demonstrated a correlation between facial color characteristics and heart disease (Ren et al., 2020). Similarly, significant decreases in tongue features such as T_RGB_R, T_contrast, and T_homogeneity indicated paler tongue color, rougher texture, and reduced homogeneity. These findings are consistent with the TCM theory that “the tongue is the sprout of the heart,” where insufficient heart blood or stasis leads to pathological changes in the tongue, such as dull coloration, ecchymoses, or coarse texture. Recent studies have also confirmed that tongue color parameters (e.g., RGB values and contrast) are significantly correlated with the degree of coronary artery stenosis in CAD patients (Li et al., 2024; Rismawan et al., 2024). Due to blood stasis and impaired circulation, such patients often present with a purplish tongue, petechiae, dull facial complexion, or cyanotic lips (Lin and Wang, 2020). Altogether, alterations in the color and texture features of both tongue and facial images provide valuable cues for intelligent CAD diagnosis. The explainability embedded in our model not only enhances its clinical applicability in real-world scenarios but also builds user trust among healthcare professionals. As current medical research increasingly explores non-traditional biomarkers for CAD, tongue and facial features have emerged as promising diagnostic modalities. Our study contributes to the growing body of work in this domain and offers a valuable reference for future investigations into tongue-facial biomarkers in CAD.

In comparison with classical clinical risk prediction models, the results of this study also demonstrated encouraging significance. According to the literature, the Framingham risk score typically achieves an AUC of 0.70–0.75 (Wilson et al., 1998; D’Agostino et al., 2008; Lin et al., 2020), while the ASCVD 10-year risk assessment reports an AUC of approximately 0.74–0.78 (Andrus and Lacaille, 2014). These models are primarily designed for long-term risk prediction in asymptomatic populations, whereas the present model focuses on cross-sectional, real-time screening and diagnosis. In the external validation cohort, our model achieved an AUC of 0.91, indicating superior discriminative ability. Although this comparison is indirect, it suggests that image-based artificial intelligence tools may provide complementary value to existing clinical risk prediction methods, warranting further validation in future studies that integrate clinical variables with image features.

In addition, through interpretability analysis of the model, we found that, CAD patients showed overall lower values in facial features such as F_RGB_R, F_RGB_G, and F_RGB_B, suggesting a lack of redness and brightness in the skin. This aligns with the TCM concept of “facial dullness” (mian se hui an), which is thought to reflect impaired facial microcirculation due to weakened cardiac function or blood stasis in the coronary vessels. This phenomenon is supported by previous studies, which have demonstrated a correlation between facial color characteristics and heart disease (Ren et al., 2020). Similarly, significant decreases in tongue features such as T_RGB_R, T_contrast, and T_homogeneity indicated paler tongue color, rougher texture, and reduced homogeneity. These findings are consistent with the TCM theory that “the tongue is the sprout of the heart,” where insufficient heart blood or stasis leads to pathological changes in the tongue, such as dull coloration, ecchymoses, or coarse texture. Recent studies have also confirmed that tongue color parameters (e.g., RGB values and contrast) are significantly correlated with the degree of coronary artery stenosis in CAD patients (Li et al., 2024; Rismawan et al., 2024). Due to blood stasis and impaired circulation, such patients often present with a purplish tongue, petechiae, dull facial complexion, or cyanotic lips (Lin and Wang, 2020). Altogether, alterations in the color and texture features of both tongue and facial images provide valuable cues for intelligent CAD diagnosis. The explainability embedded in our model not only enhances its clinical applicability in real-world scenarios but also builds user trust among healthcare professionals. As current medical research increasingly explores non-traditional biomarkers for CAD, tongue and facial features have emerged as promising diagnostic modalities. Our study contributes to the growing body of work in this domain and offers a valuable reference for future investigations into tongue-facial biomarkers in CAD.

The model developed in this study demonstrates significant potential for widespread application. By simply capturing tongue and facial images, it enables rapid screening of CAD patients in a non-invasive, convenient, and cost-effective manner. These advantages make it particularly suitable for large-scale community-based screening, carrying important public health implications. In the future, we plan to further optimize and validate this model in community populations to promote its broad application in extensive CAD screening programs. Additionally, the model holds promise as a user-friendly tool for home-based health monitoring. When integrated with mobile devices, this non-invasive approach allows individuals to conveniently monitor their cardiovascular health at home, facilitating early detection of potential issues and reducing the risk of delayed diagnosis. Although promising, this study has several limitations. The dataset is relatively small and limited to Chinese participants, whose skin types are predominantly Fitzpatrick III–IV, resulting in low pigment variation and minimal risk of skin tone–related bias. Nevertheless, the model requires validation in larger and more diverse populations with broader skin tone representation. Due to the lack of detailed clinical data, subgroup analyses were restricted to three major cardiovascular risk factors: hypertension, diabetes, and sex. Furthermore, the case–control design may overestimate real-world performance, necessitating prospective evaluation in low-prevalence settings. Because image acquisition requires active participant cooperation, severe cases such as acute myocardial infarction or coronary stenosis greater than or equal to 70 percent were underrepresented in the sample. Although the imaging device incorporates hardware-level color calibration, differences in lighting conditions and equipment configurations across sites may still affect image consistency. Future multicenter deployments will incorporate gray cards or standard color charts to further enhance imaging consistency.

## 5 Conclusion

This study proposes a dual-branch multimodal fusion model incorporating a feature-wise gating mechanism for non-invasive and intelligent diagnosis of CAD based on multidimensional tongue and facial image features. The approach integrates deep semantic features extracted by the MDFA-Swin network with interpretable traditional color and texture features, effectively capturing multi-scale information associated with CAD from tongue and facial images. The model demonstrated strong generalizability and practical applicability on an independent external test set. SHAP-based interpretability analysis further revealed the decision pathways through which tongue and facial features contributed to the model’s predictions. This study provides quantitative evidence supporting the diagnostic value of tongue and facial signs in CAD. Its non-invasive, low-cost, and efficient nature offers a novel approach for community-based screening and home-based health monitoring of CAD.

## Data Availability

The raw data supporting the conclusions of this article will be made available by the authors, without undue reservation.
