# Supplementary figures and images for: An explainable dual-modal diagnostic model for coronary artery disease: a feature-gated approach using tongue and facial image features (part 2 of 3)
Source: Front Artif Intell. 2025 Nov 17;8:1662577. doi: 10.3389/frai.2025.1662577 (PMC12665729; doi:10.3389/frai.2025.1662577)

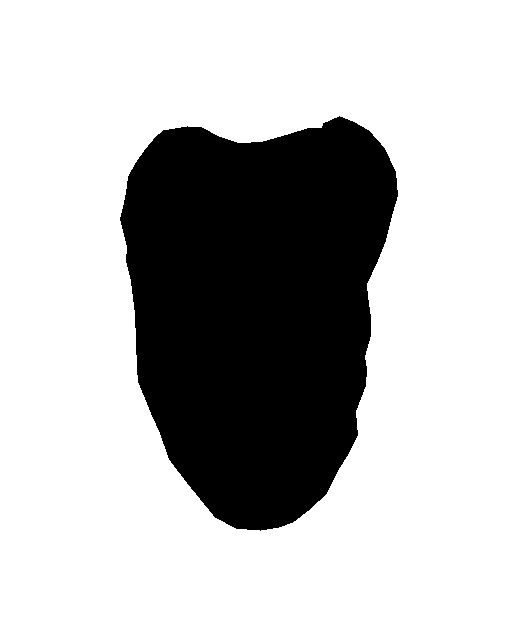

Supplement: Supplementary file 1 [file Data_Sheet_1.zip › tongue/001_tongue_mask.png]

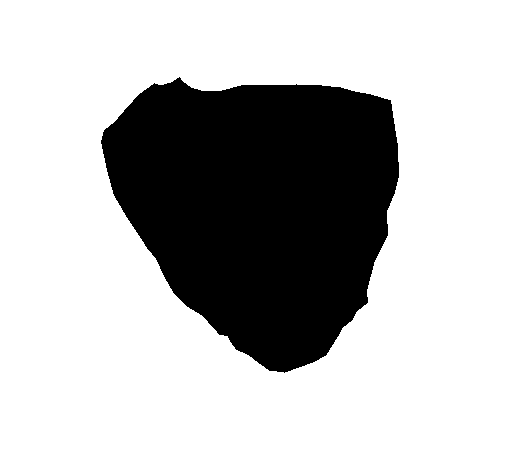

Supplement: Supplementary file 1 [file Data_Sheet_1.zip › tongue/002_tongue_mask.png]

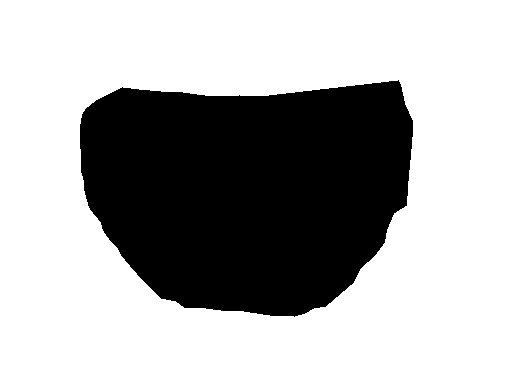

Supplement: Supplementary file 1 [file Data_Sheet_1.zip › tongue/003_tongue_mask.png]

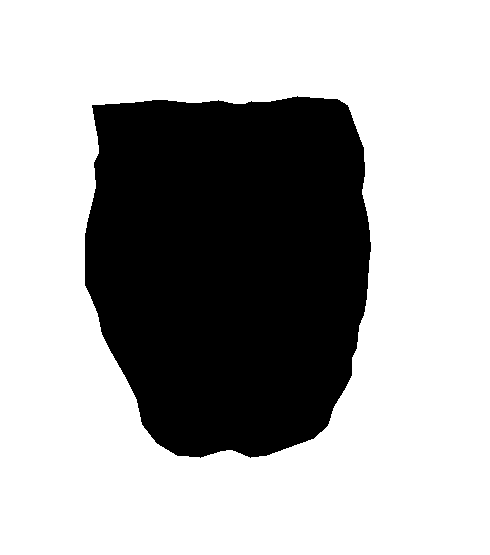

Supplement: Supplementary file 1 [file Data_Sheet_1.zip › tongue/004_tongue_mask.png]

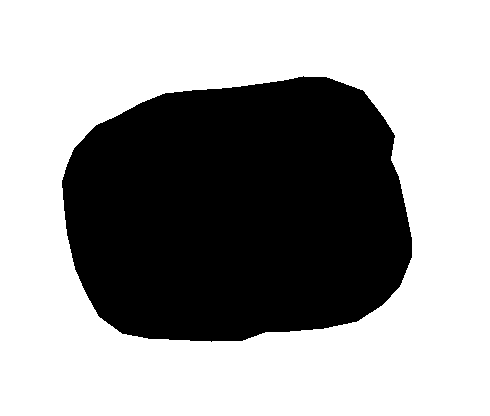

Supplement: Supplementary file 1 [file Data_Sheet_1.zip › tongue/005_tongue_mask.png]

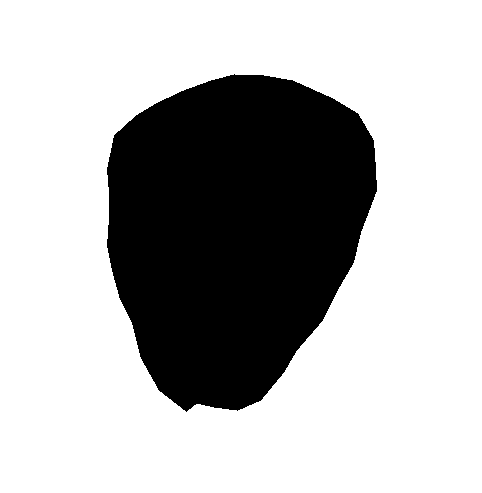

Supplement: Supplementary file 1 [file Data_Sheet_1.zip › tongue/006_tongue_mask.png]

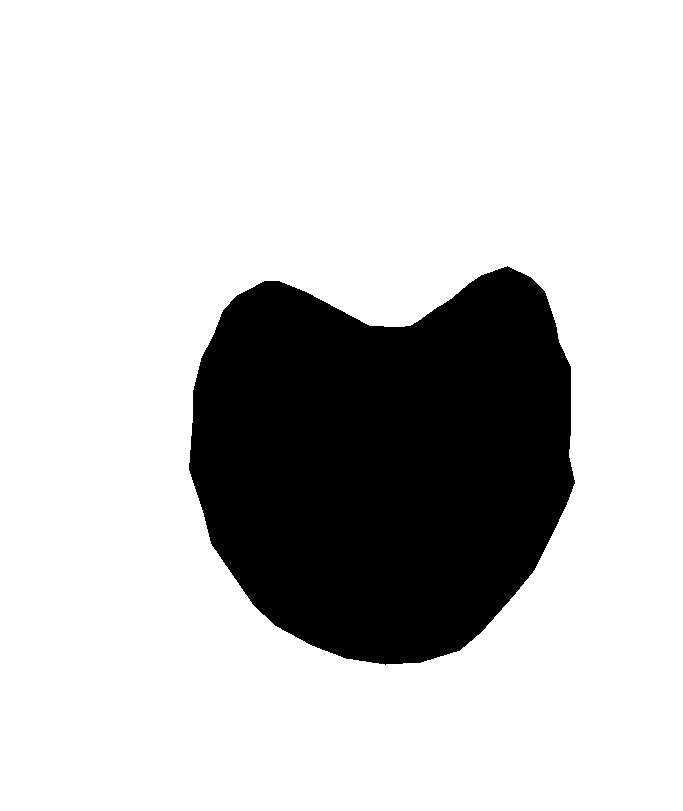

Supplement: Supplementary file 1 [file Data_Sheet_1.zip › tongue/007_tongue_mask.png]

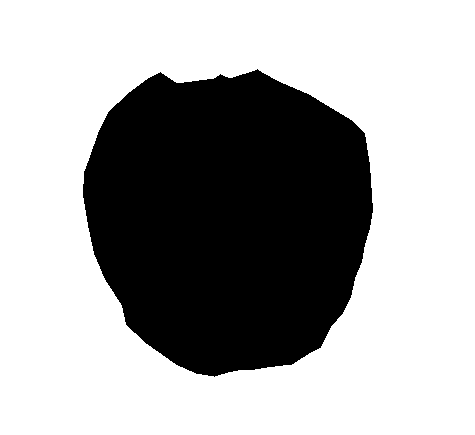

Supplement: Supplementary file 1 [file Data_Sheet_1.zip › tongue/008_tongue_mask.png]

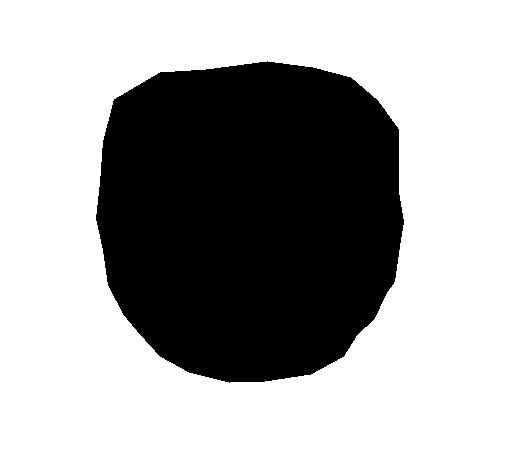

Supplement: Supplementary file 1 [file Data_Sheet_1.zip › tongue/009_tongue_mask.png]

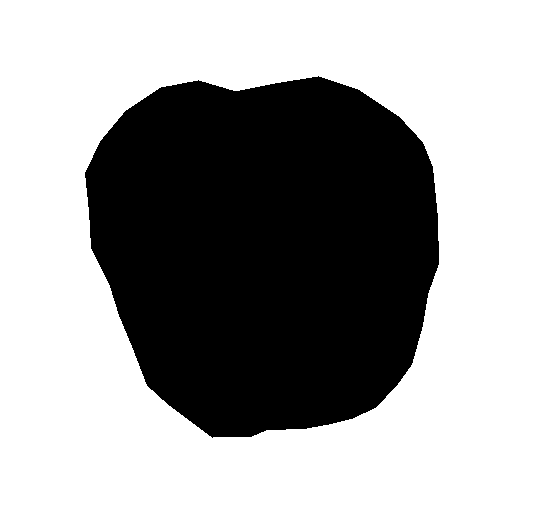

Supplement: Supplementary file 1 [file Data_Sheet_1.zip › tongue/010_tongue_mask.png]

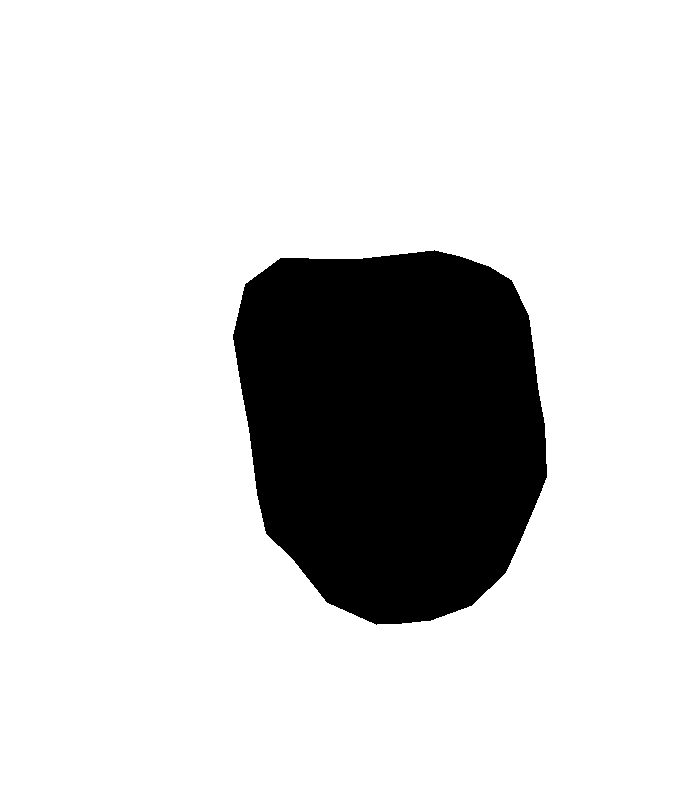

Supplement: Supplementary file 1 [file Data_Sheet_1.zip › tongue/011_tongue_mask.png]

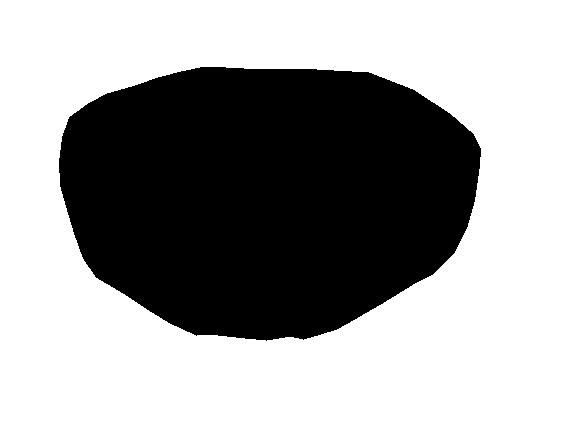

Supplement: Supplementary file 1 [file Data_Sheet_1.zip › tongue/012_tongue_mask.png]

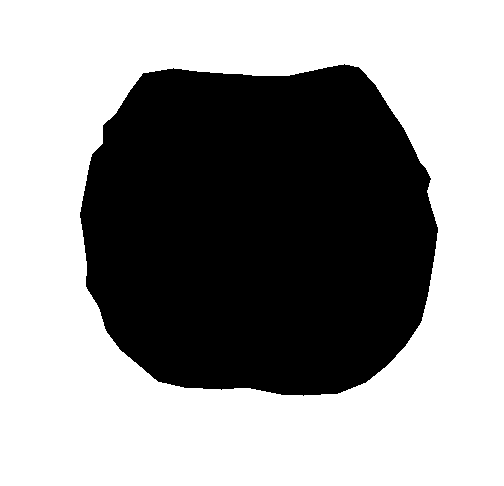

Supplement: Supplementary file 1 [file Data_Sheet_1.zip › tongue/013_tongue_mask.png]

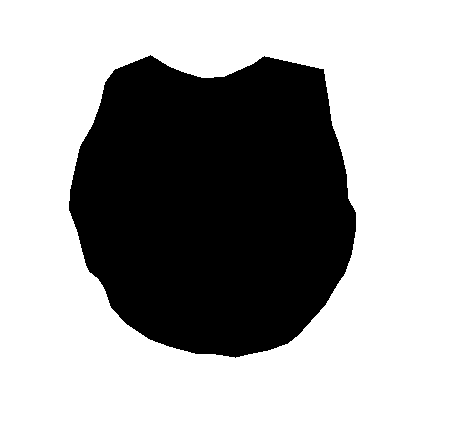

Supplement: Supplementary file 1 [file Data_Sheet_1.zip › tongue/014_tongue_mask.png]

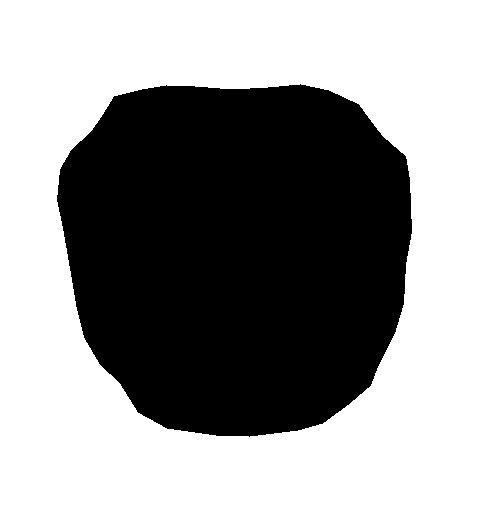

Supplement: Supplementary file 1 [file Data_Sheet_1.zip › tongue/015_tongue_mask.png]

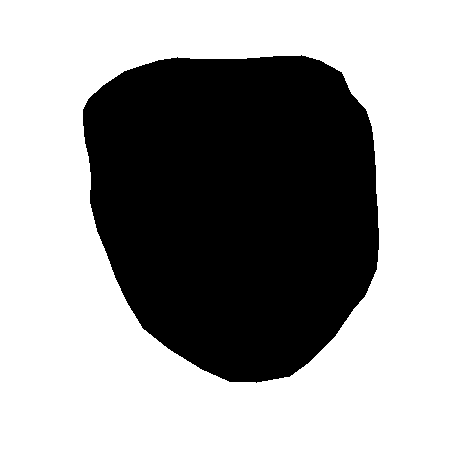

Supplement: Supplementary file 1 [file Data_Sheet_1.zip › tongue/016_tongue_mask.png]

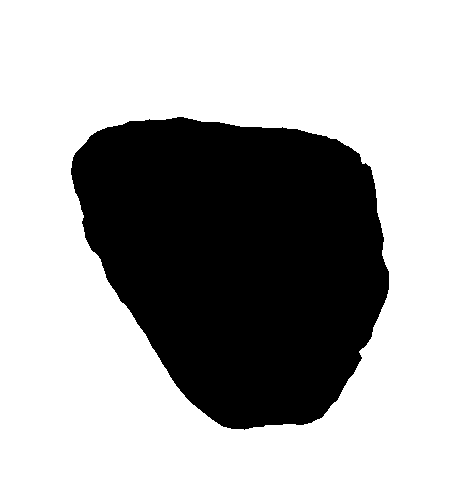

Supplement: Supplementary file 1 [file Data_Sheet_1.zip › tongue/017_tongue_mask.png]

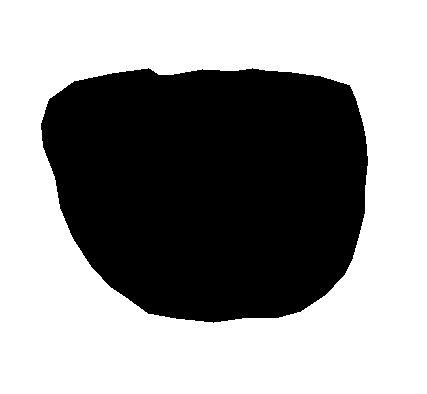

Supplement: Supplementary file 1 [file Data_Sheet_1.zip › tongue/018_tongue_mask.png]

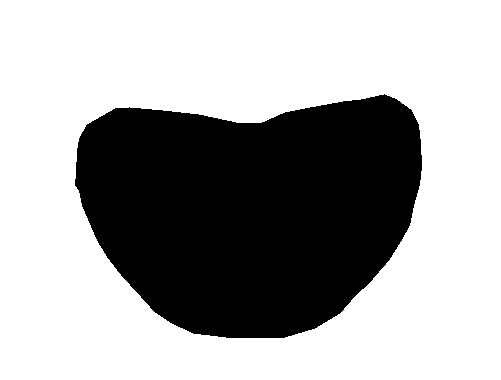

Supplement: Supplementary file 1 [file Data_Sheet_1.zip › tongue/019_tongue_mask.png]

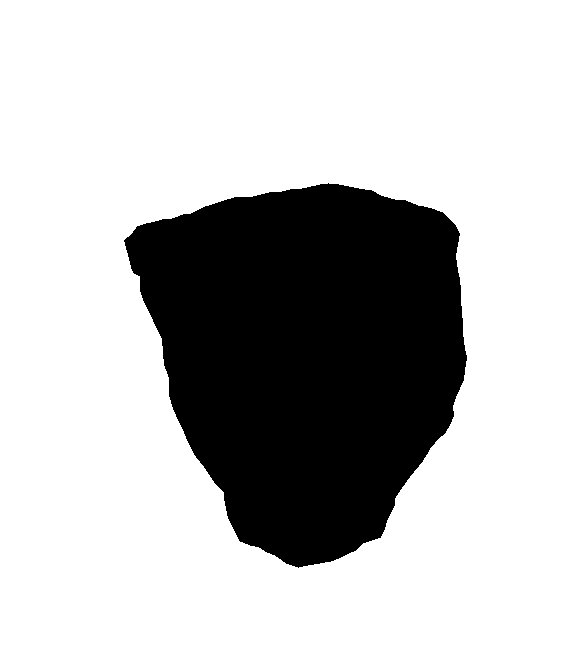

Supplement: Supplementary file 1 [file Data_Sheet_1.zip › tongue/020_tongue_mask.png]

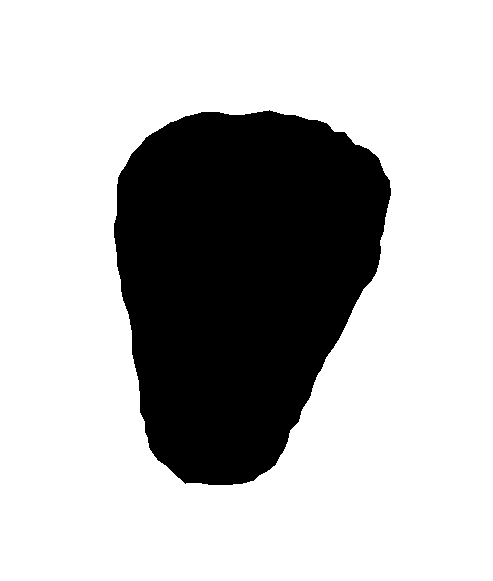

Supplement: Supplementary file 1 [file Data_Sheet_1.zip › tongue/021_tongue_mask.png]

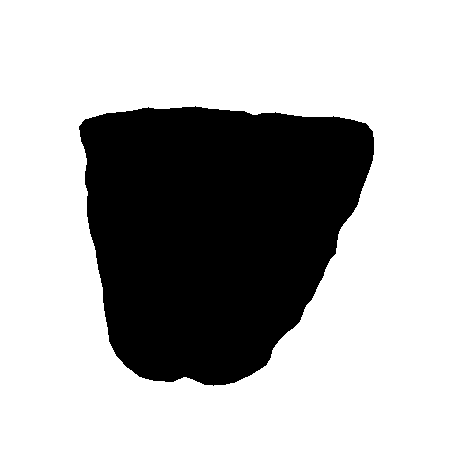

Supplement: Supplementary file 1 [file Data_Sheet_1.zip › tongue/022_tongue_mask.png]

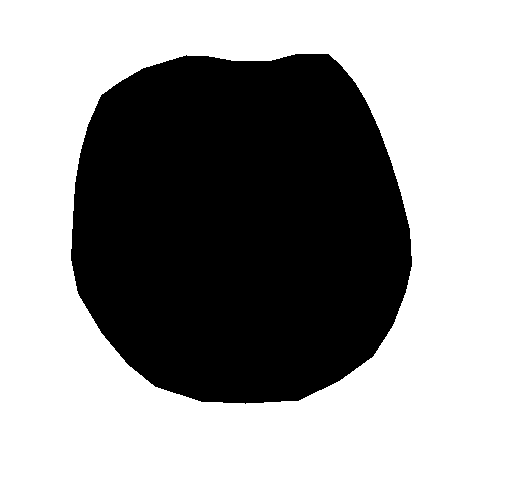

Supplement: Supplementary file 1 [file Data_Sheet_1.zip › tongue/023_tongue_mask.png]

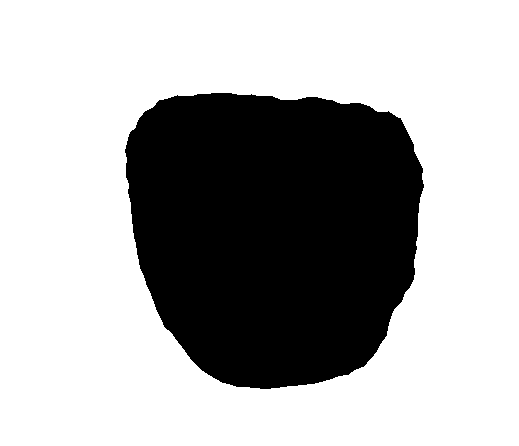

Supplement: Supplementary file 1 [file Data_Sheet_1.zip › tongue/024_tongue_mask.png]

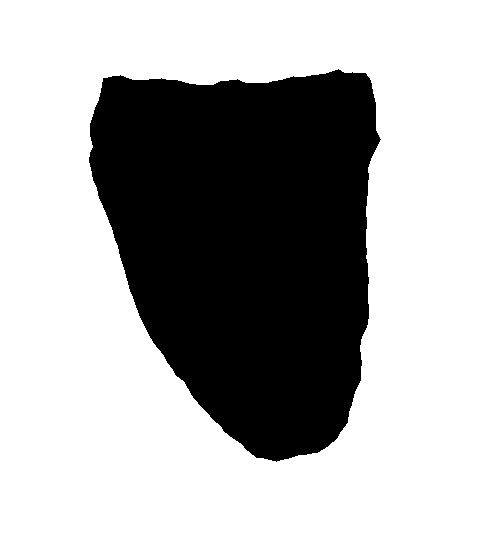

Supplement: Supplementary file 1 [file Data_Sheet_1.zip › tongue/025_tongue_mask.png]

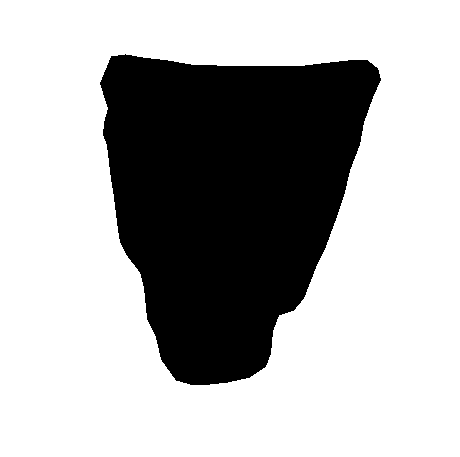

Supplement: Supplementary file 1 [file Data_Sheet_1.zip › tongue/026_tongue_mask.png]

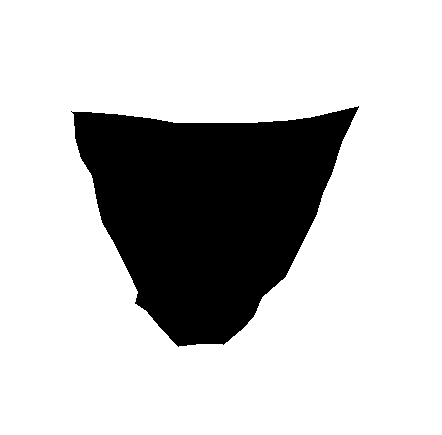

Supplement: Supplementary file 1 [file Data_Sheet_1.zip › tongue/027_tongue_mask.png]

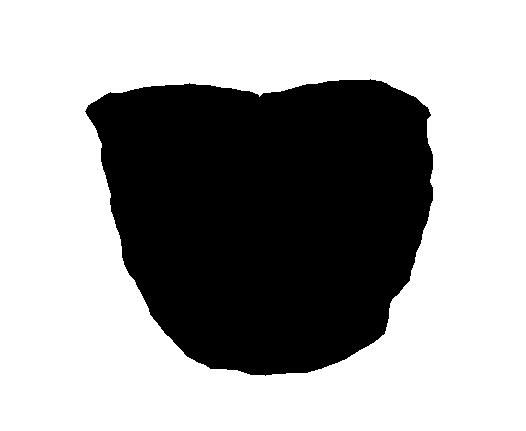

Supplement: Supplementary file 1 [file Data_Sheet_1.zip › tongue/028_tongue_mask.png]

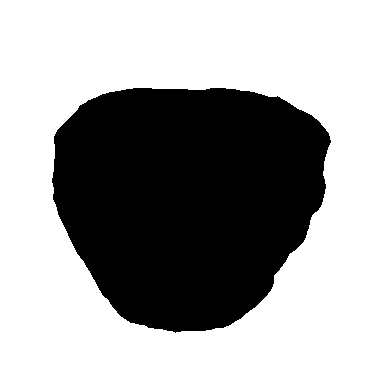

Supplement: Supplementary file 1 [file Data_Sheet_1.zip › tongue/029_tongue_mask.png]

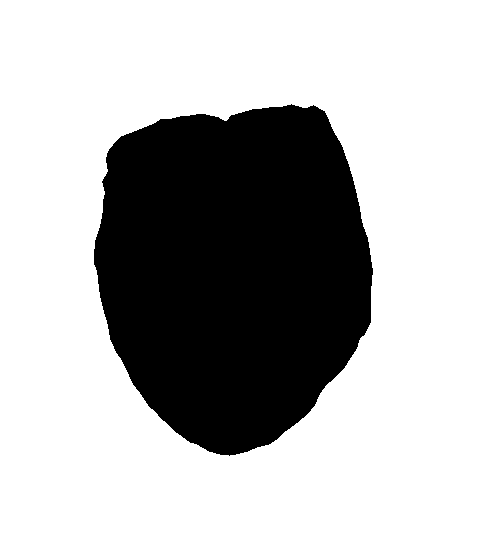

Supplement: Supplementary file 1 [file Data_Sheet_1.zip › tongue/030_tongue_mask.png]

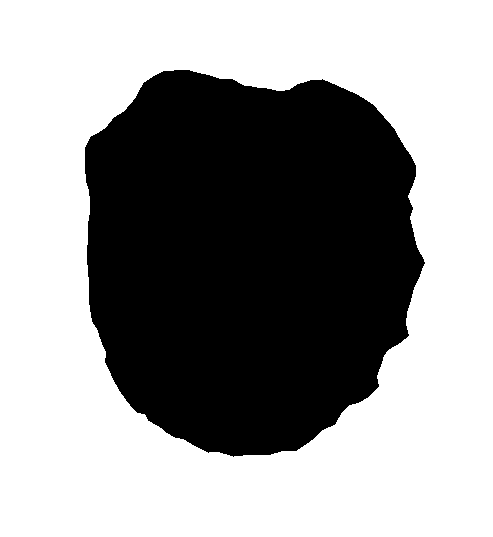

Supplement: Supplementary file 1 [file Data_Sheet_1.zip › tongue/031_tongue_mask.png]

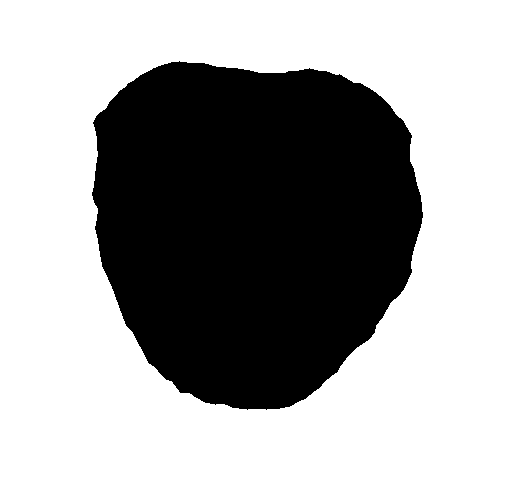

Supplement: Supplementary file 1 [file Data_Sheet_1.zip › tongue/032_tongue_mask.png]

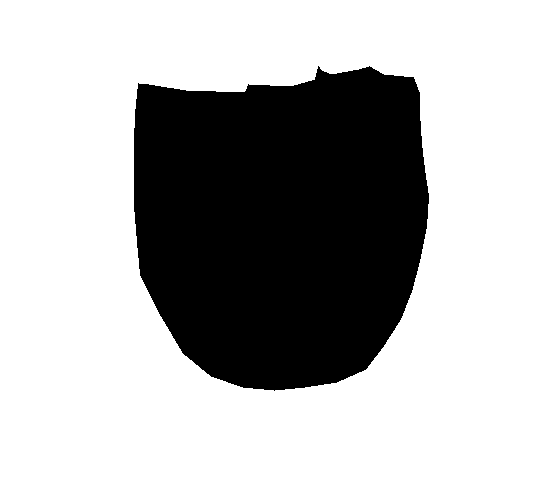

Supplement: Supplementary file 1 [file Data_Sheet_1.zip › tongue/033_tongue_mask.png]

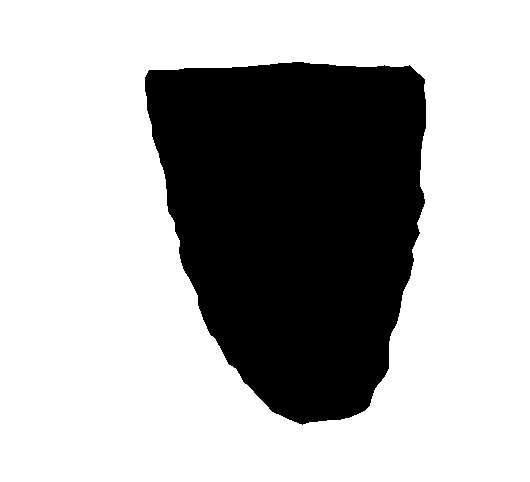

Supplement: Supplementary file 1 [file Data_Sheet_1.zip › tongue/034_tongue_mask.png]

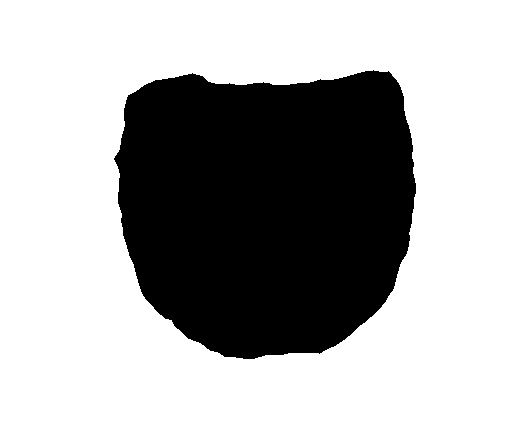

Supplement: Supplementary file 1 [file Data_Sheet_1.zip › tongue/035_tongue_mask.png]

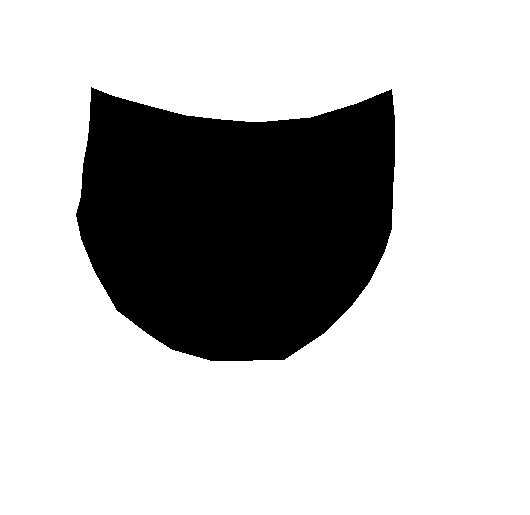

Supplement: Supplementary file 1 [file Data_Sheet_1.zip › tongue/036_tongue_mask.png]

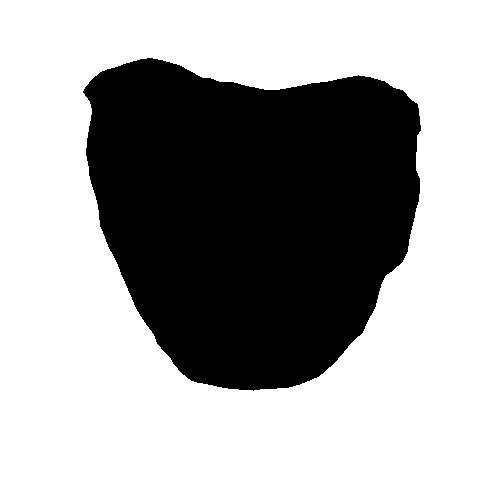

Supplement: Supplementary file 1 [file Data_Sheet_1.zip › tongue/037_tongue_mask.png]

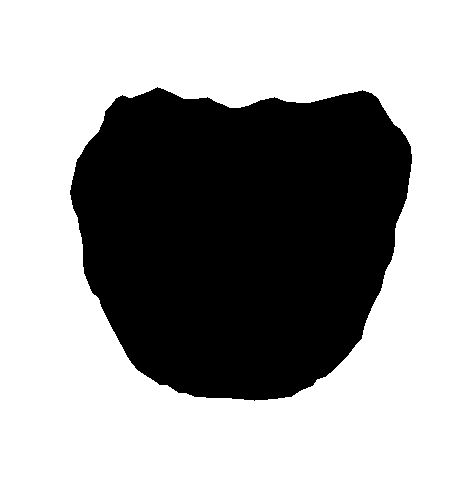

Supplement: Supplementary file 1 [file Data_Sheet_1.zip › tongue/038_tongue_mask.png]

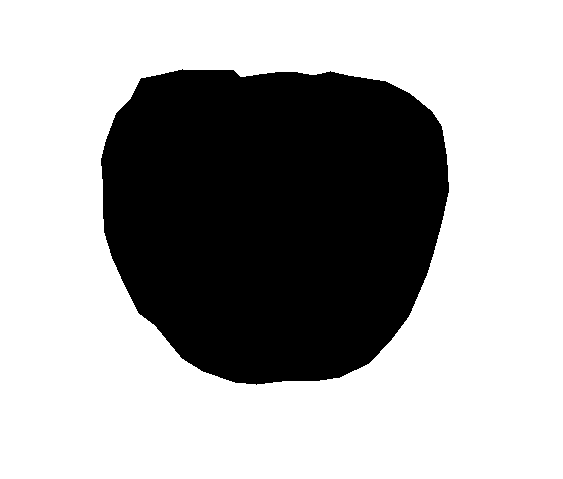

Supplement: Supplementary file 1 [file Data_Sheet_1.zip › tongue/039_tongue_mask.png]

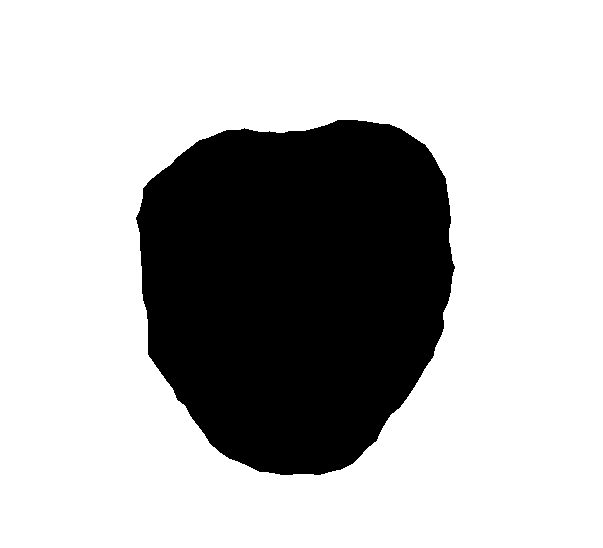

Supplement: Supplementary file 1 [file Data_Sheet_1.zip › tongue/040_tongue_mask.png]

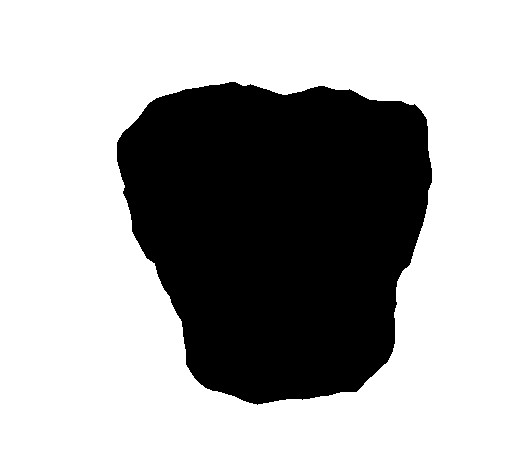

Supplement: Supplementary file 1 [file Data_Sheet_1.zip › tongue/041_tongue_mask.png]

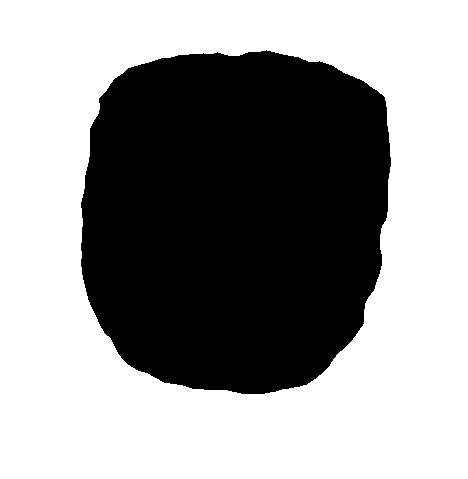

Supplement: Supplementary file 1 [file Data_Sheet_1.zip › tongue/042_tongue_mask.png]

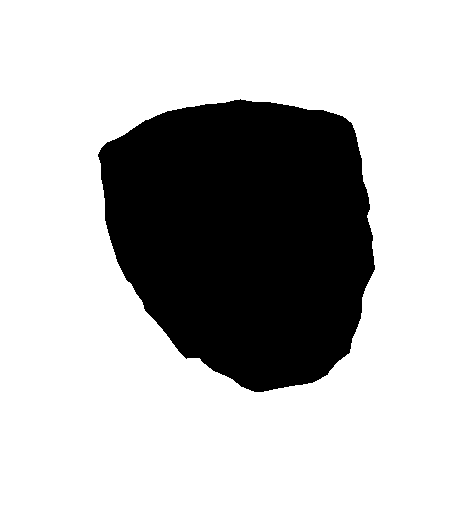

Supplement: Supplementary file 1 [file Data_Sheet_1.zip › tongue/043_tongue_mask.png]

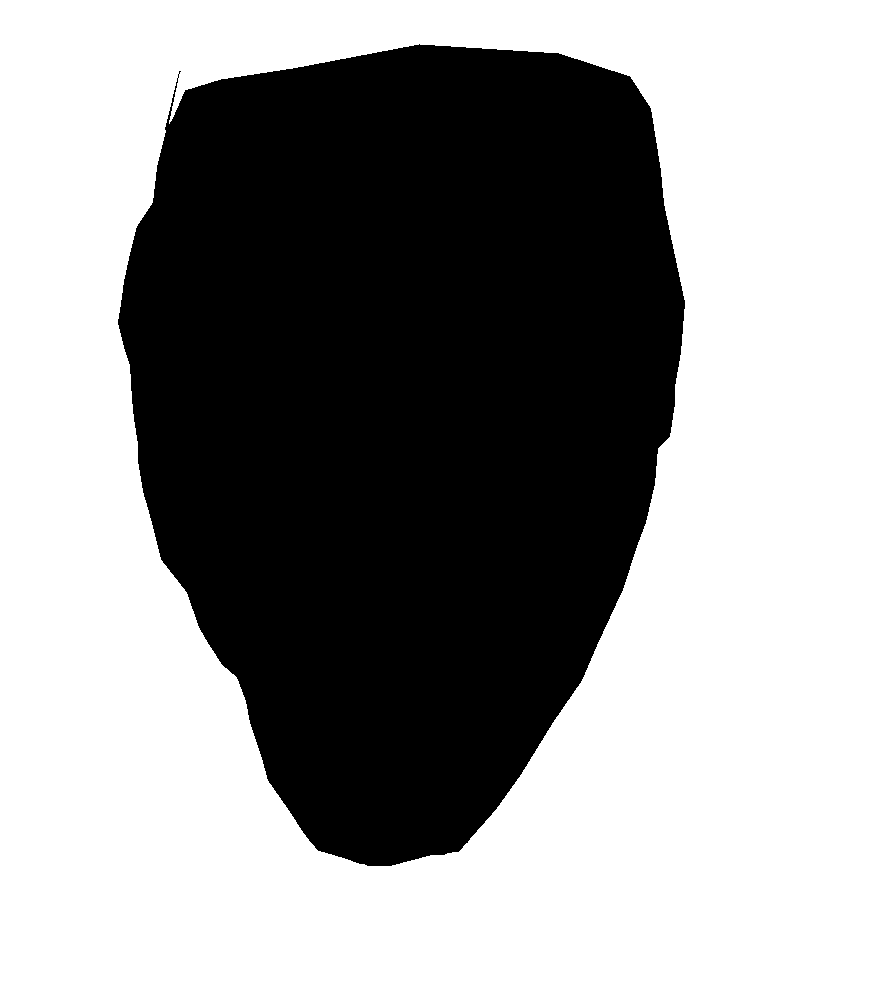

Supplement: Supplementary file 1 [file Data_Sheet_1.zip › tongue/044_tongue_mask.png]

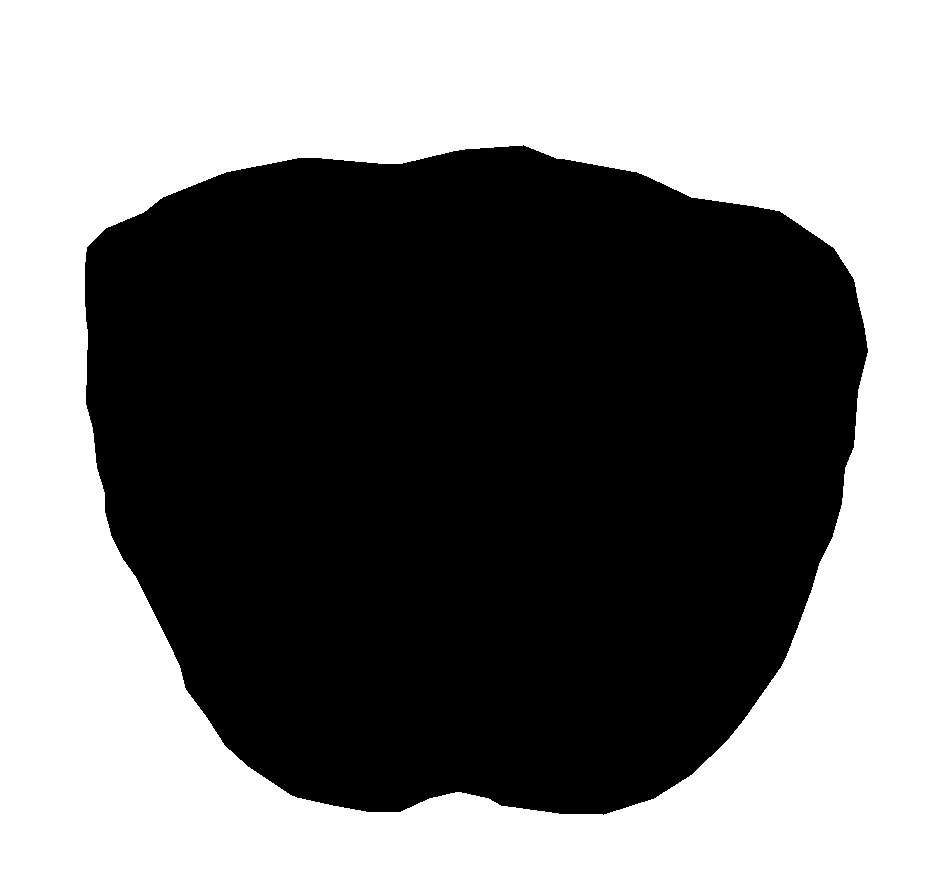

Supplement: Supplementary file 1 [file Data_Sheet_1.zip › tongue/045_tongue_mask.png]

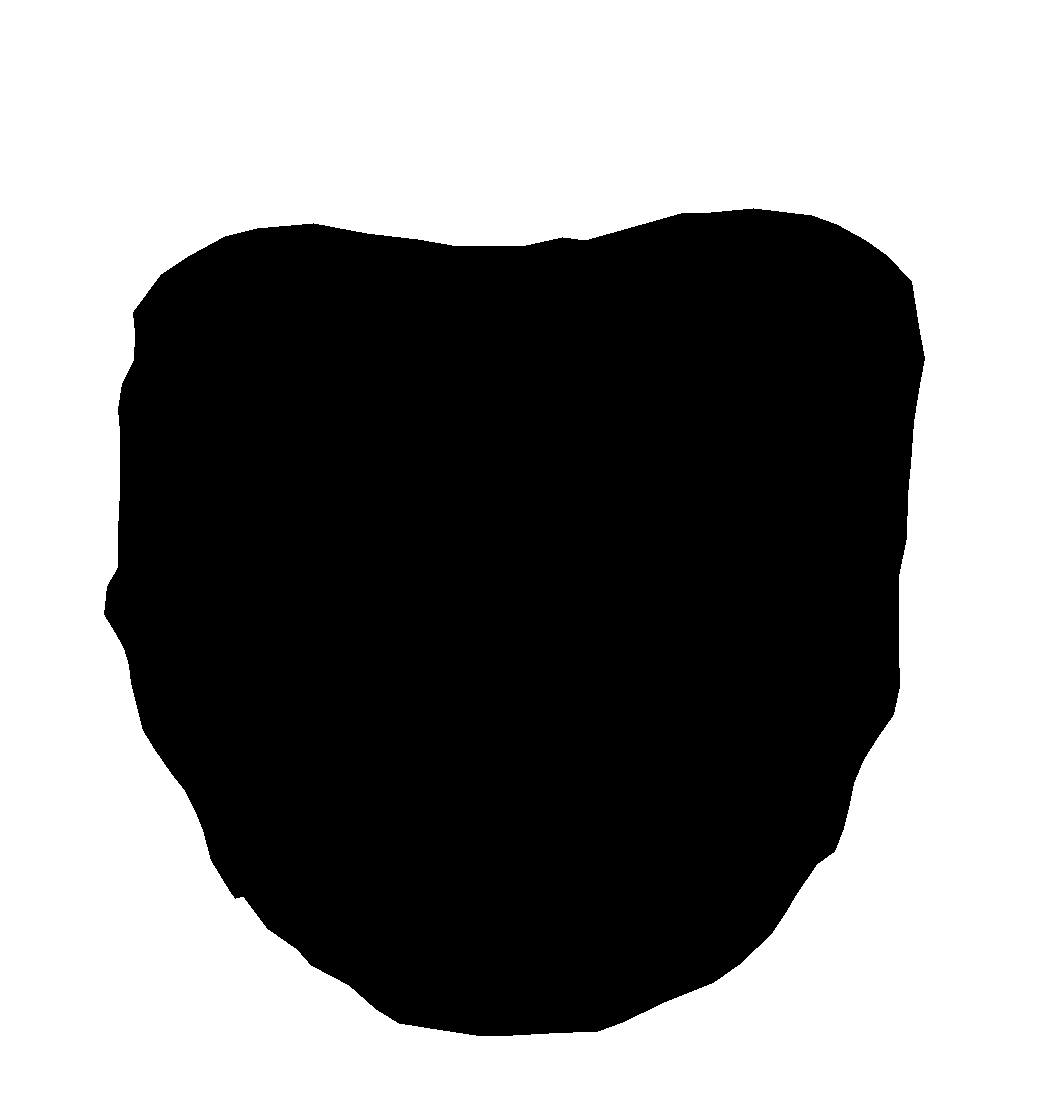

Supplement: Supplementary file 1 [file Data_Sheet_1.zip › tongue/046_tongue_mask.png]

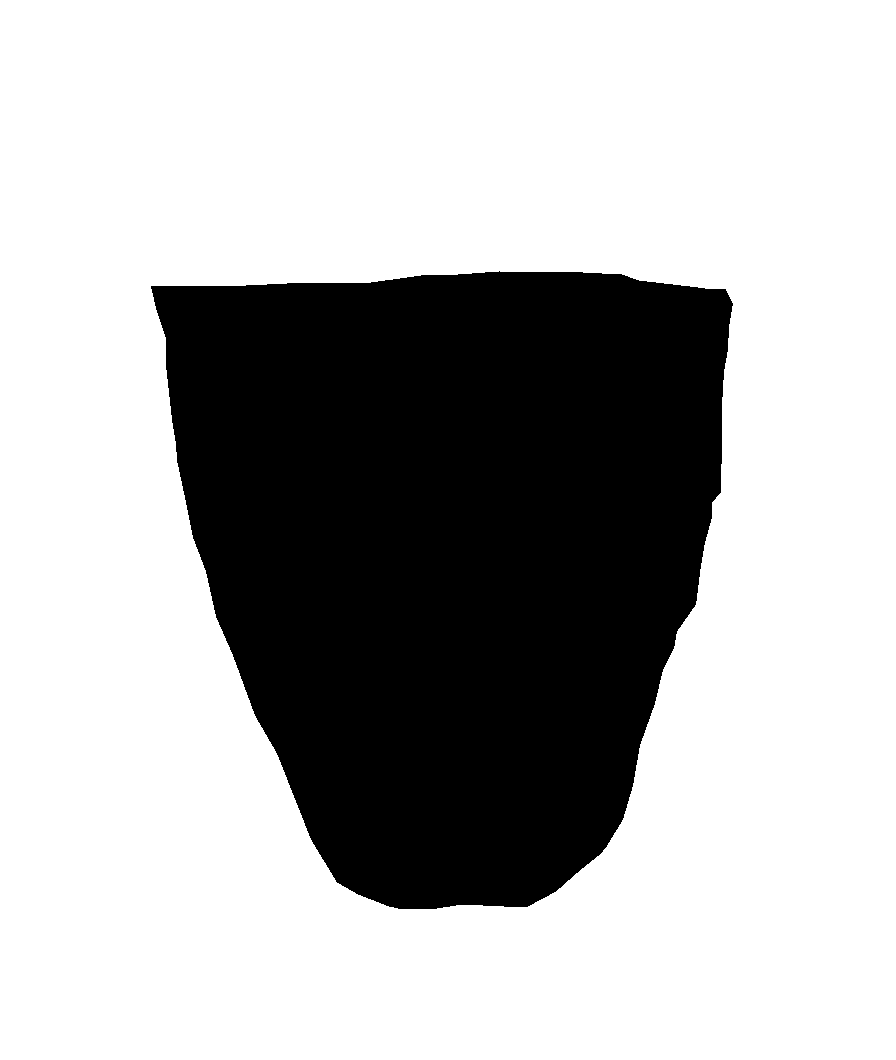

Supplement: Supplementary file 1 [file Data_Sheet_1.zip › tongue/047_tongue_mask.png]

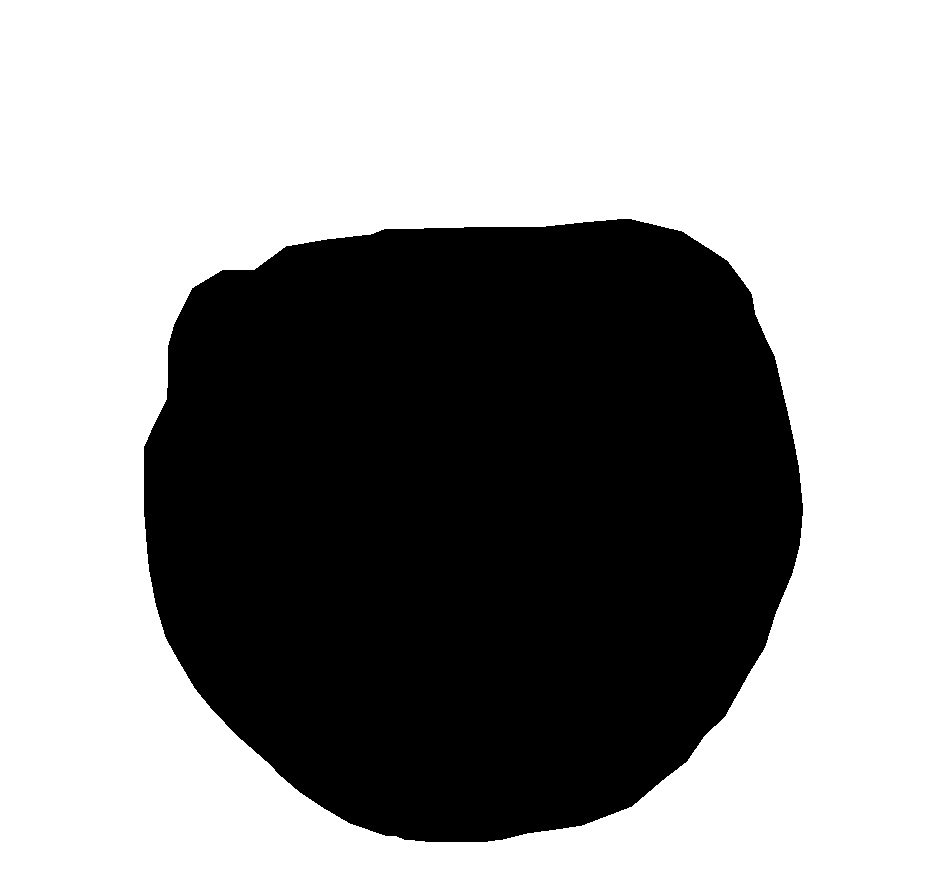

Supplement: Supplementary file 1 [file Data_Sheet_1.zip › tongue/048_tongue_mask.png]

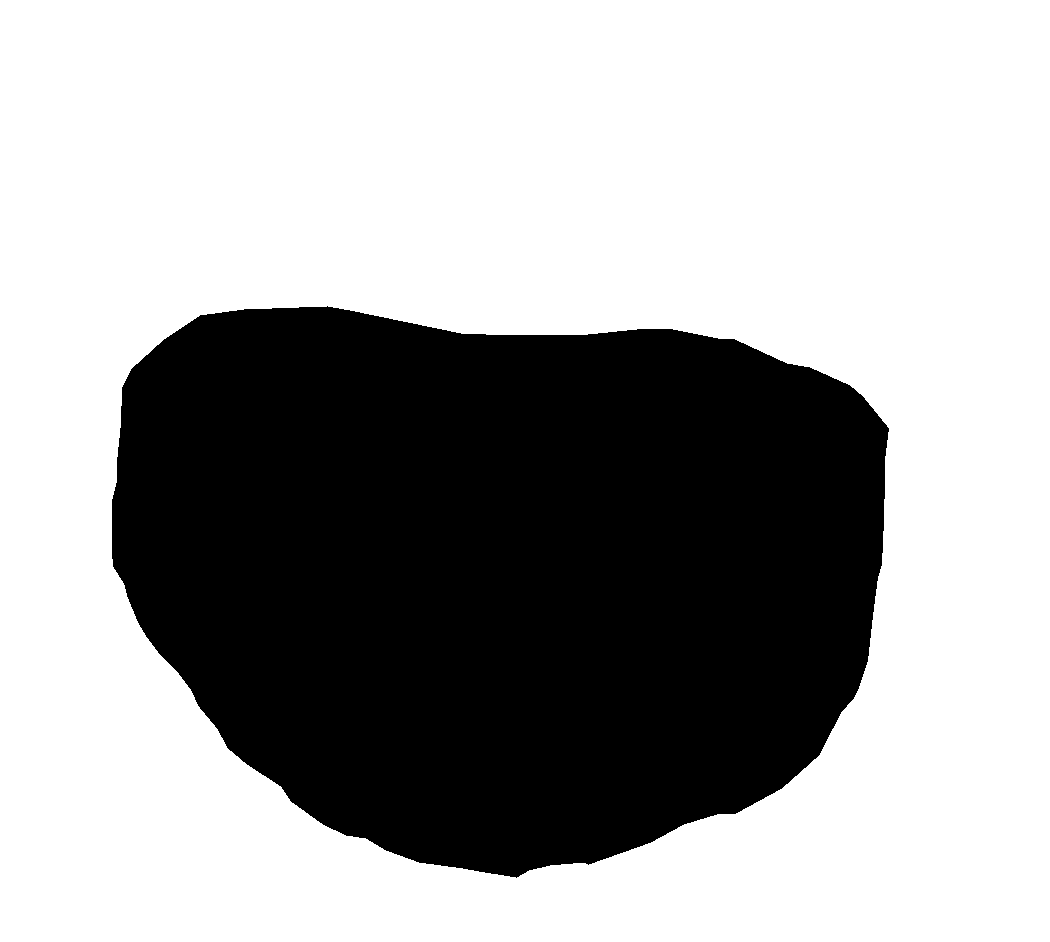

Supplement: Supplementary file 1 [file Data_Sheet_1.zip › tongue/049_tongue_mask.png]

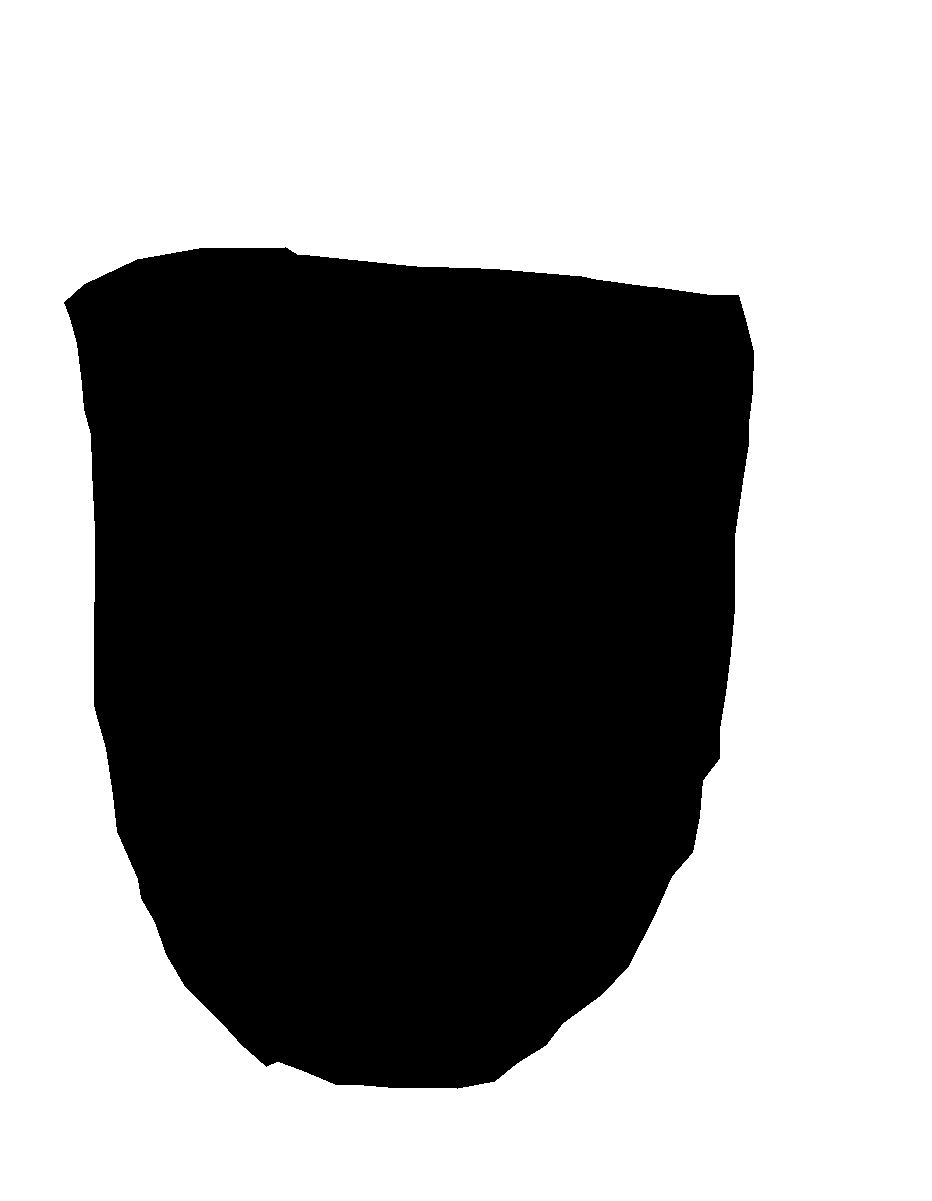

Supplement: Supplementary file 1 [file Data_Sheet_1.zip › tongue/050_tongue_mask.png]

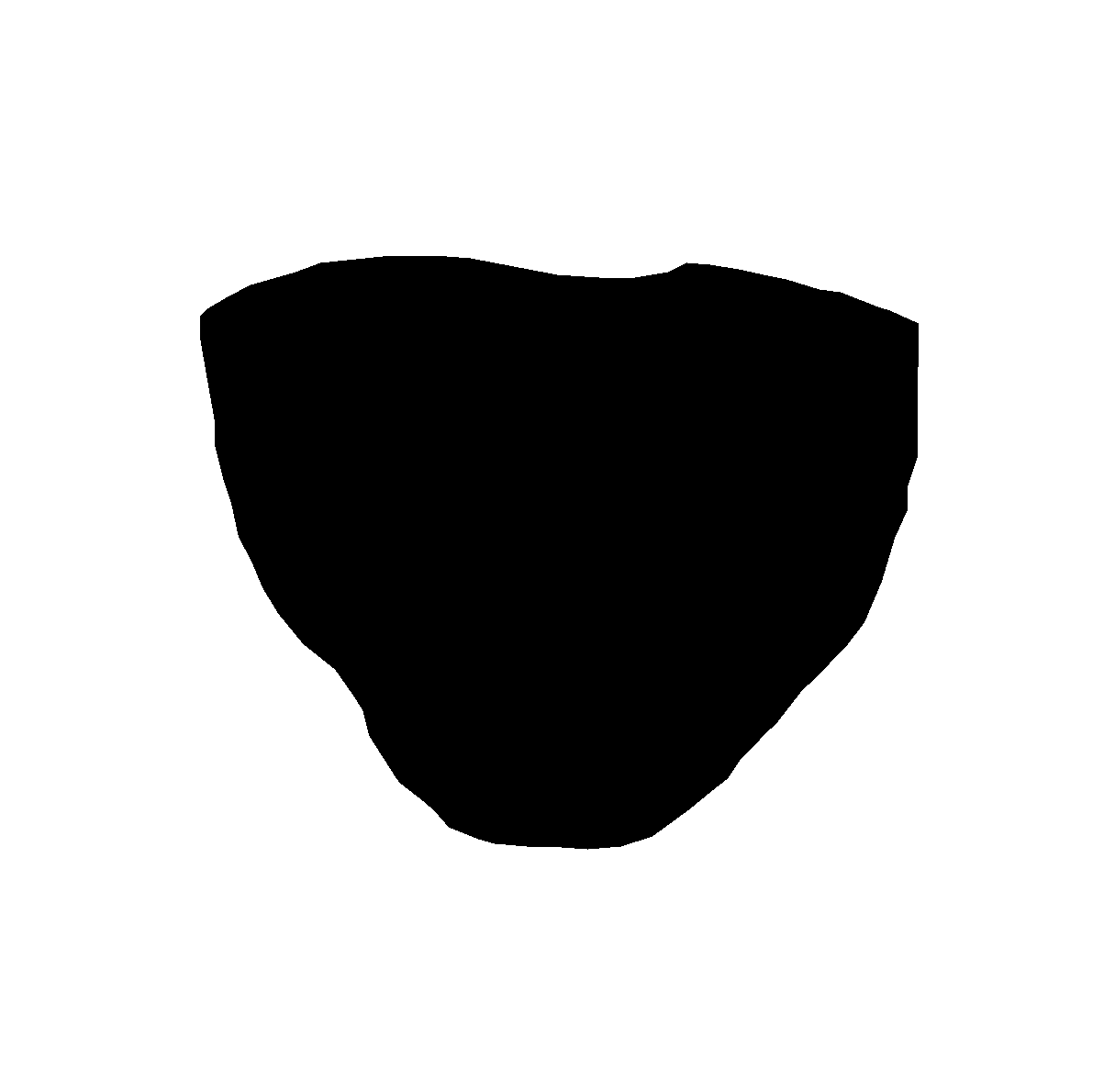

Supplement: Supplementary file 1 [file Data_Sheet_1.zip › tongue/051_tongue_mask.png]

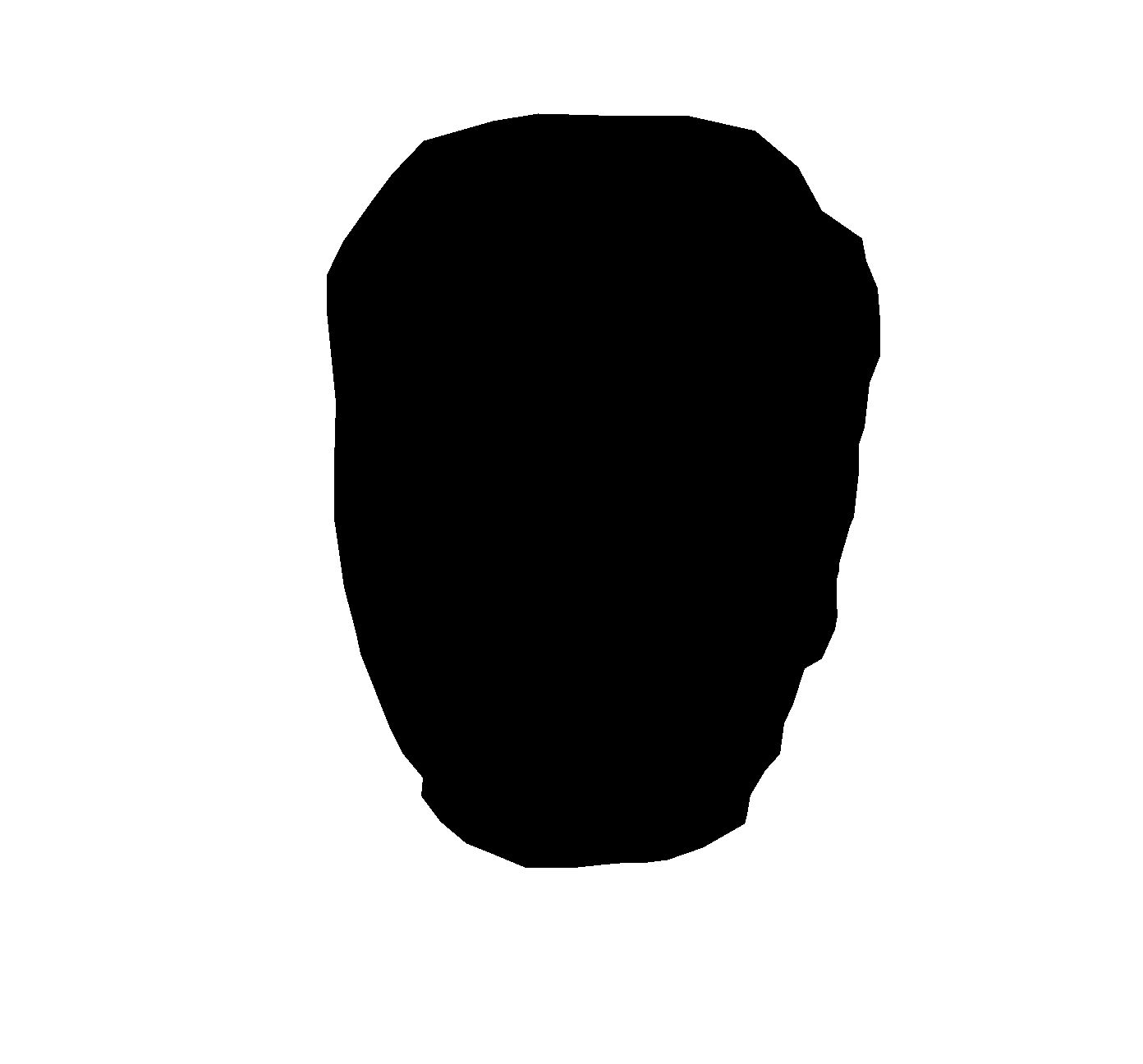

Supplement: Supplementary file 1 [file Data_Sheet_1.zip › tongue/052_tongue_mask.png]

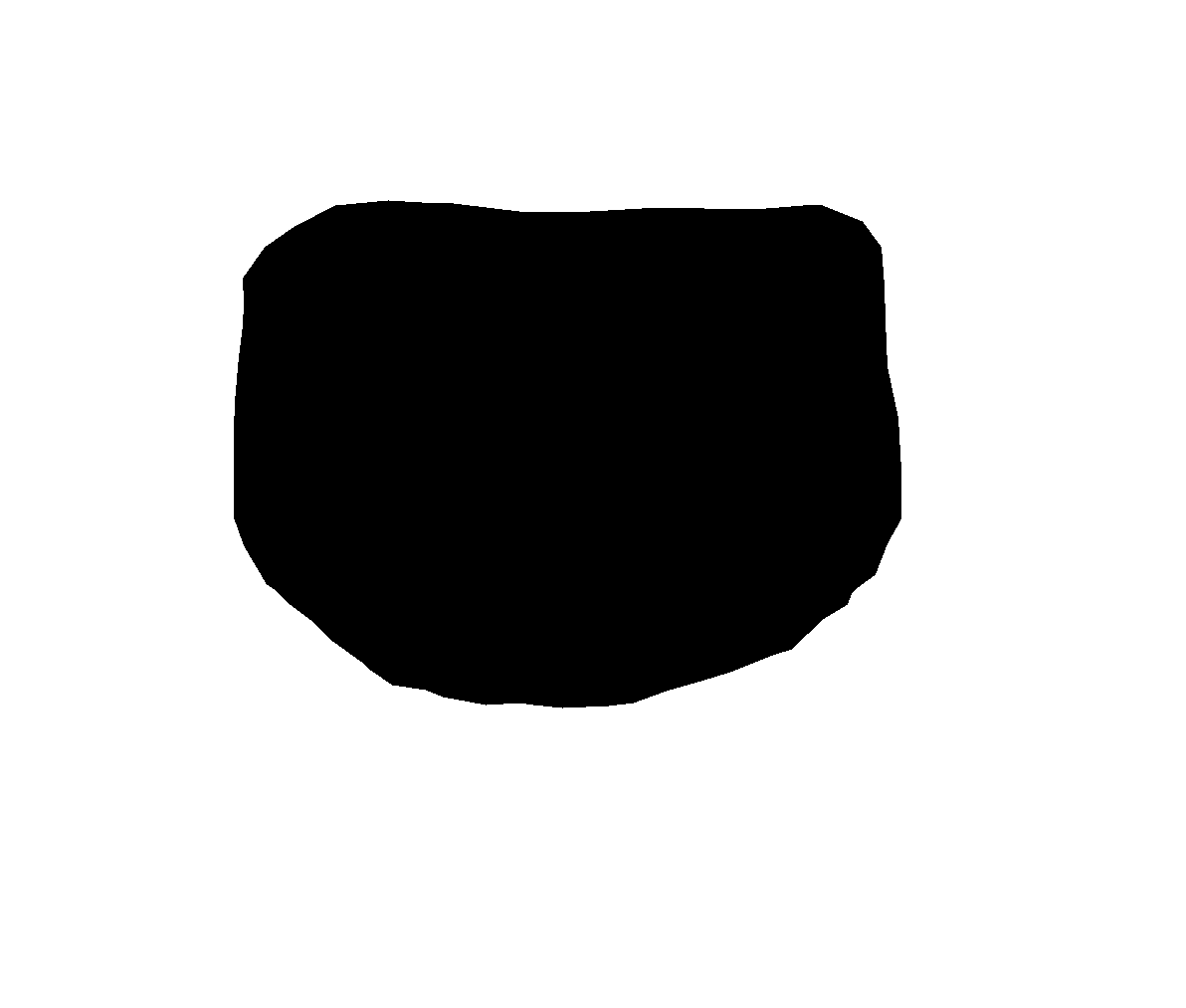

Supplement: Supplementary file 1 [file Data_Sheet_1.zip › tongue/053_tongue_mask.png]

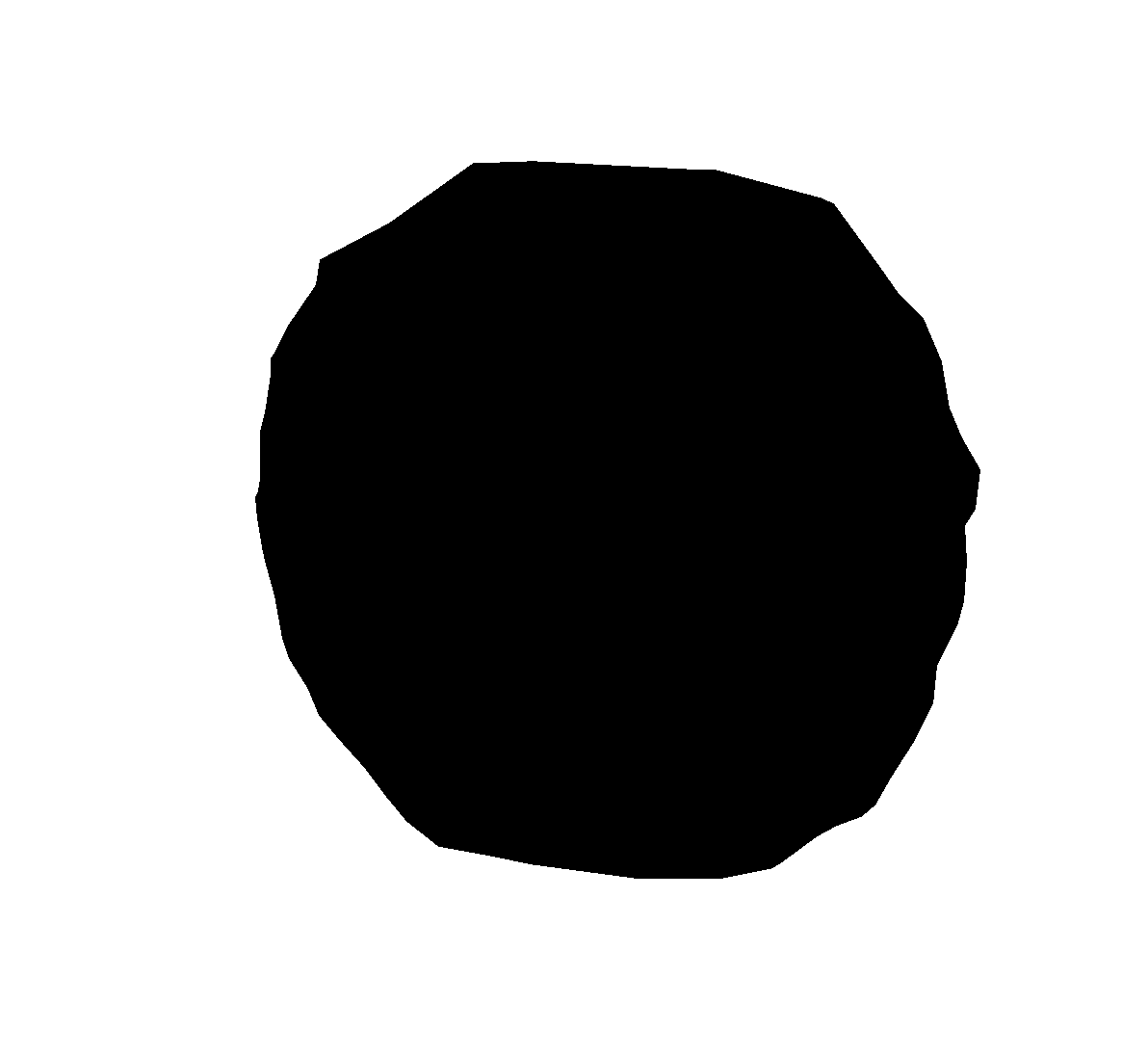

Supplement: Supplementary file 1 [file Data_Sheet_1.zip › tongue/054_tongue_mask.png]

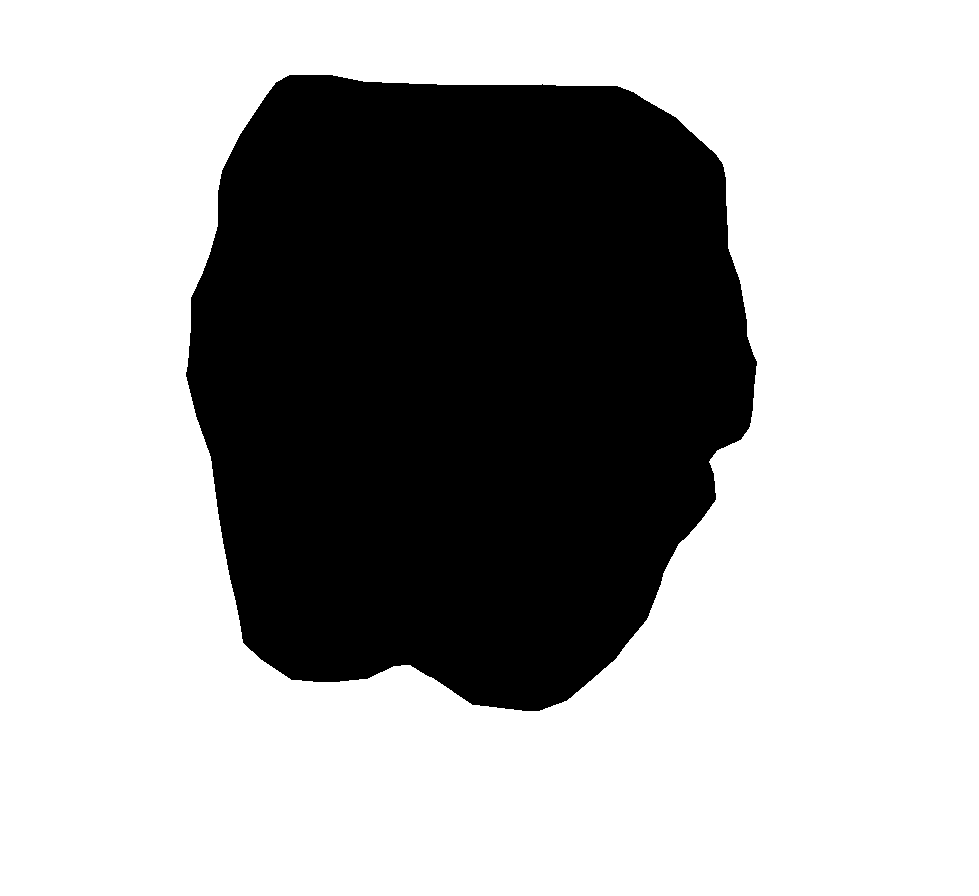

Supplement: Supplementary file 1 [file Data_Sheet_1.zip › tongue/055_tongue_mask.png]

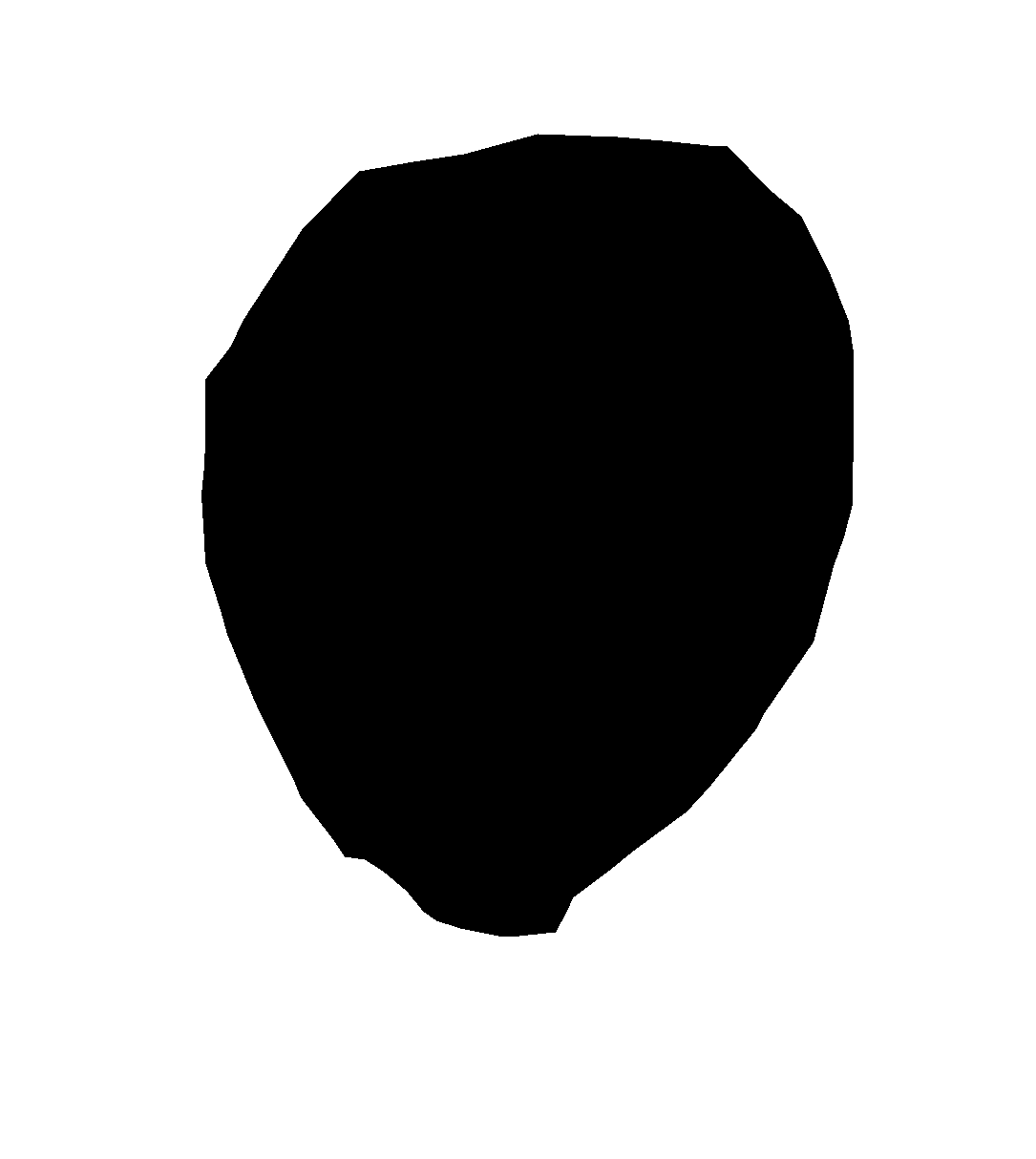

Supplement: Supplementary file 1 [file Data_Sheet_1.zip › tongue/056_tongue_mask.png]

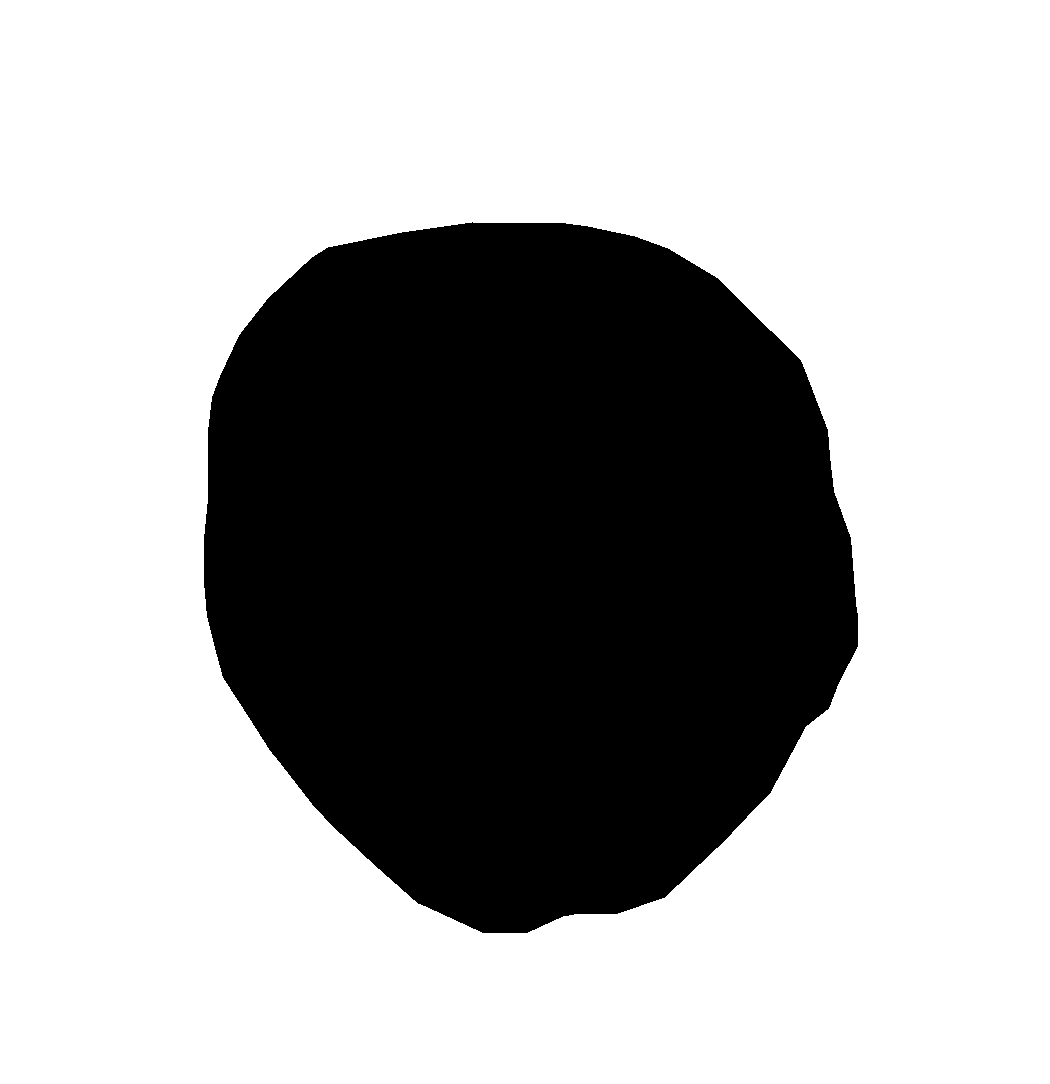

Supplement: Supplementary file 1 [file Data_Sheet_1.zip › tongue/057_tongue_mask.png]

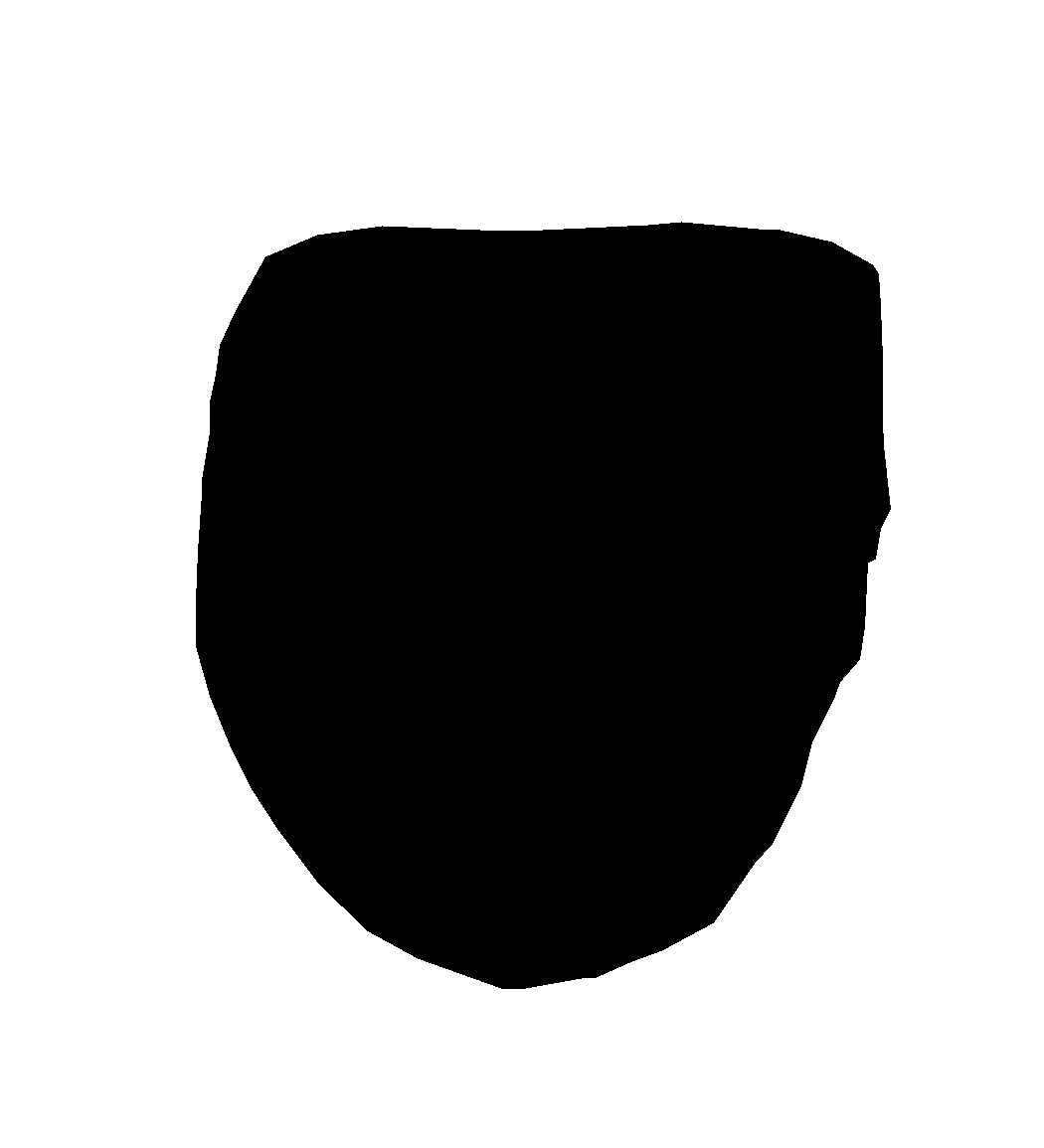

Supplement: Supplementary file 1 [file Data_Sheet_1.zip › tongue/058_tongue_mask.png]

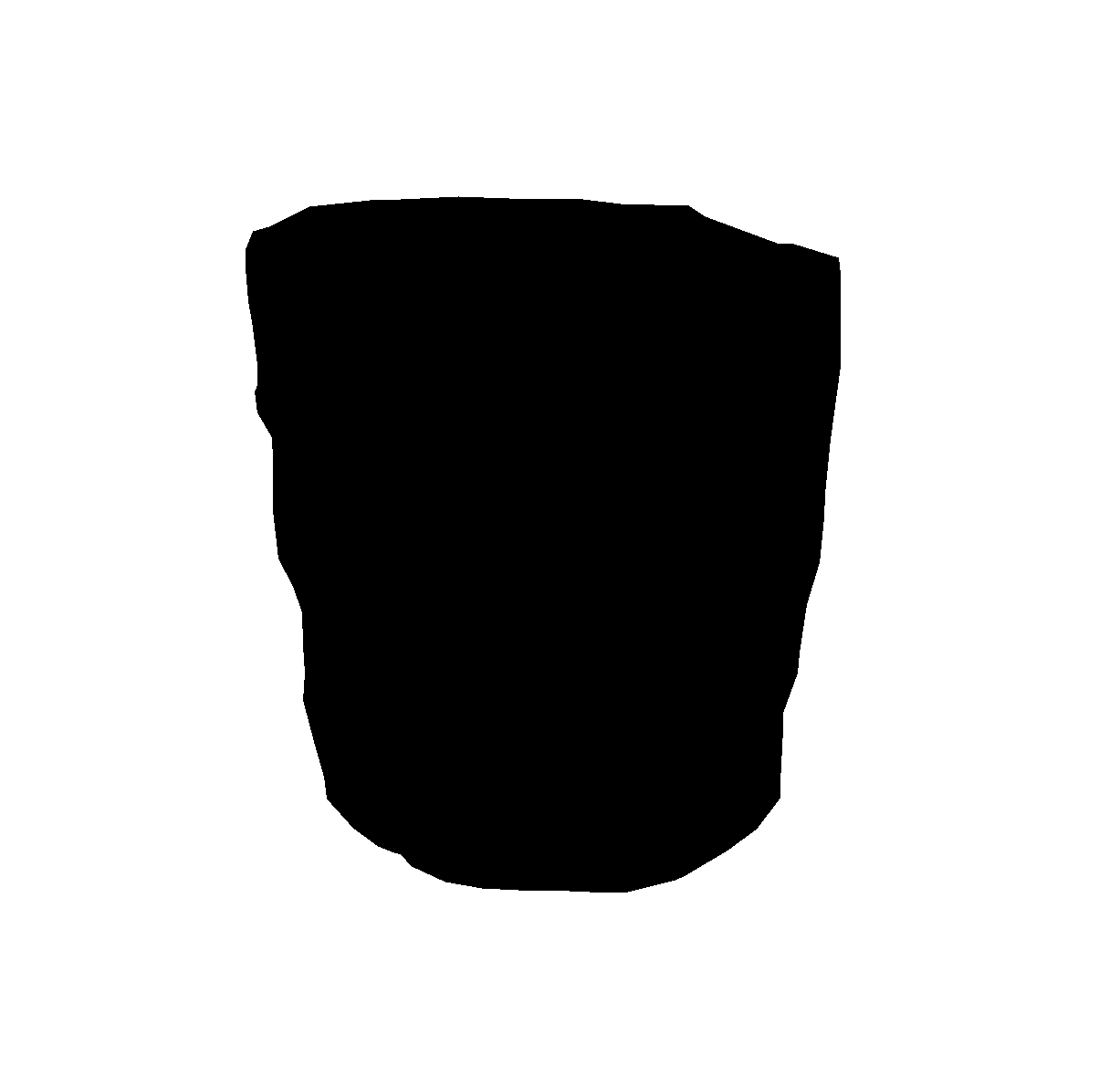

Supplement: Supplementary file 1 [file Data_Sheet_1.zip › tongue/059_tongue_mask.png]

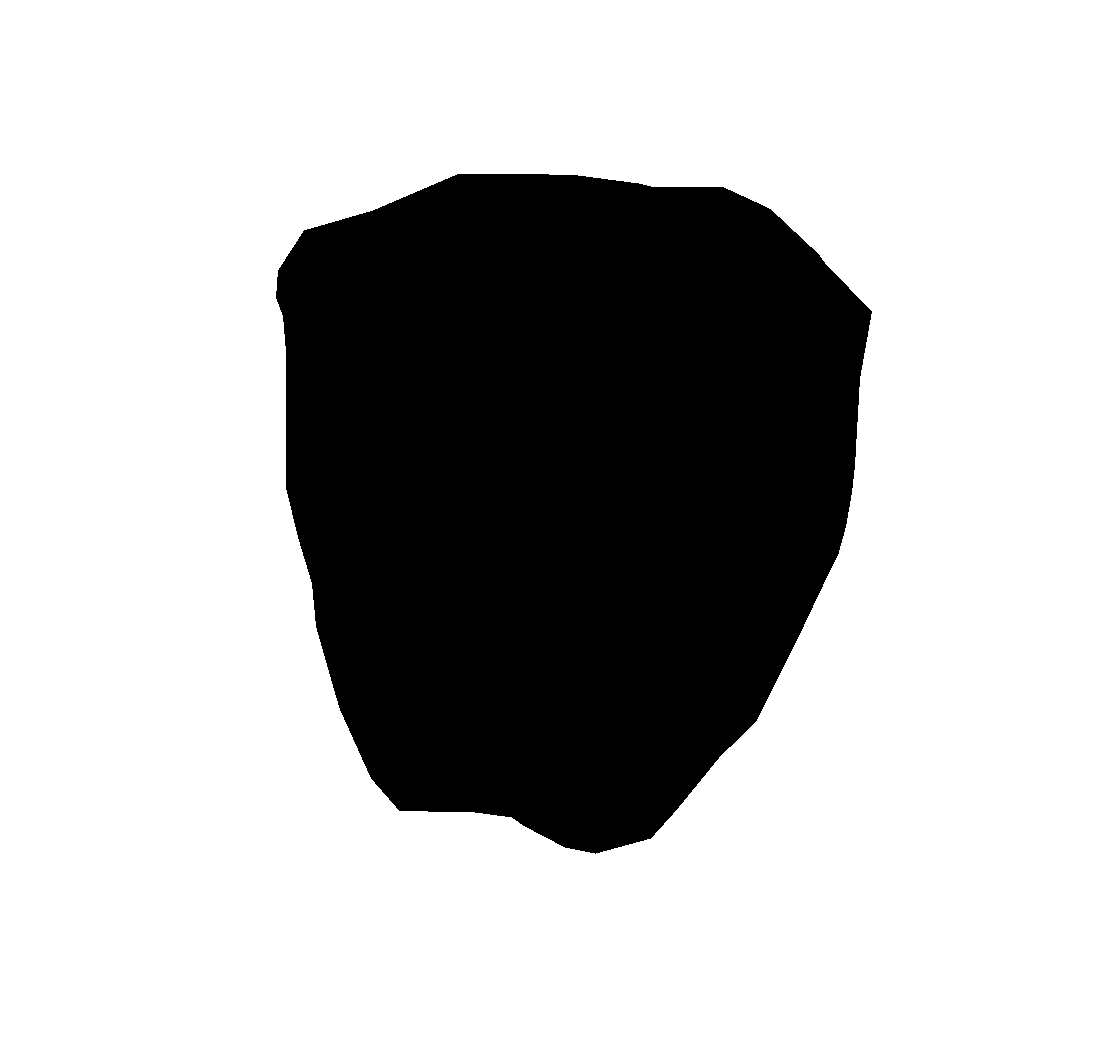

Supplement: Supplementary file 1 [file Data_Sheet_1.zip › tongue/060_tongue_mask.png]

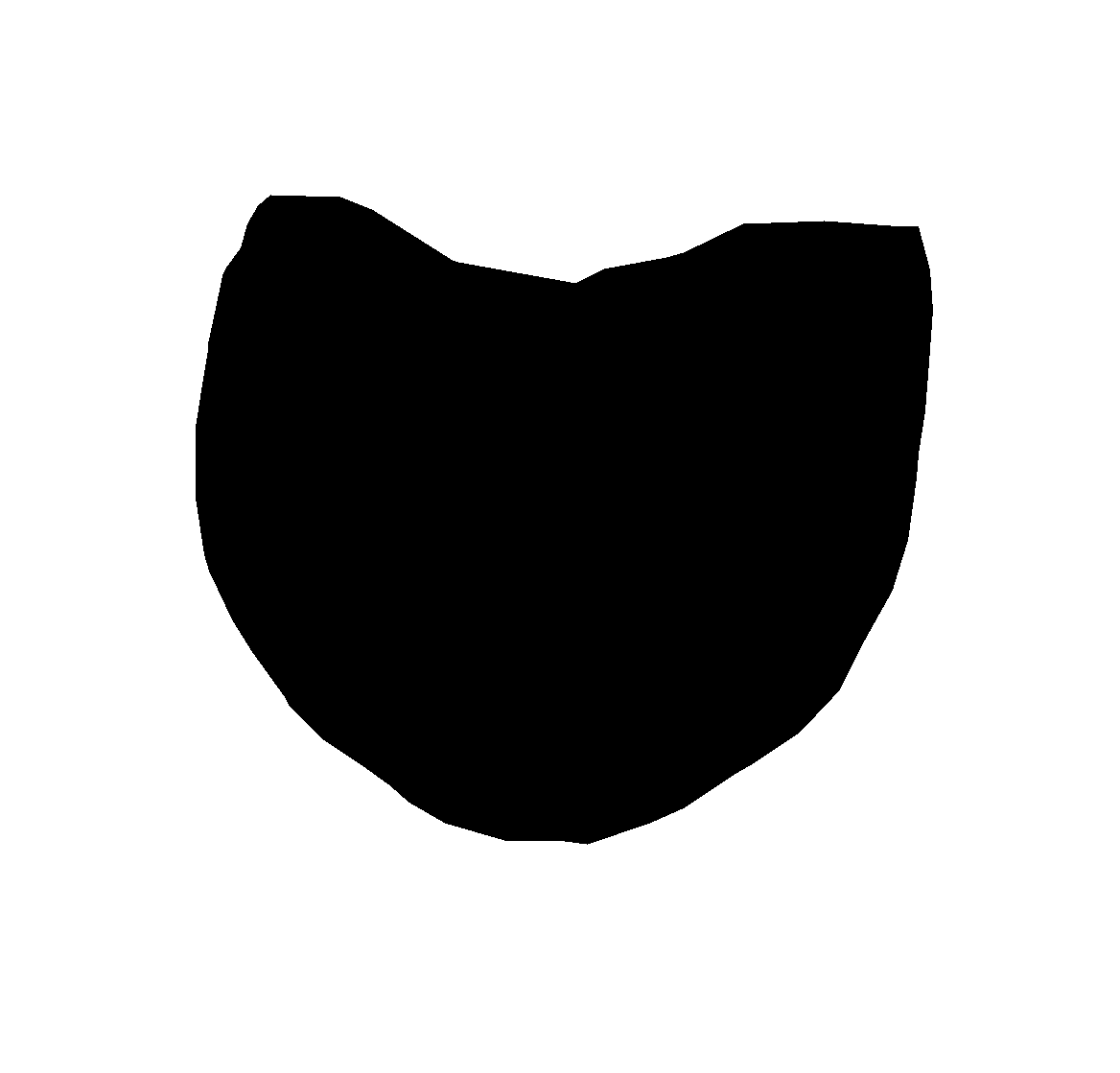

Supplement: Supplementary file 1 [file Data_Sheet_1.zip › tongue/061_tongue_mask.png]

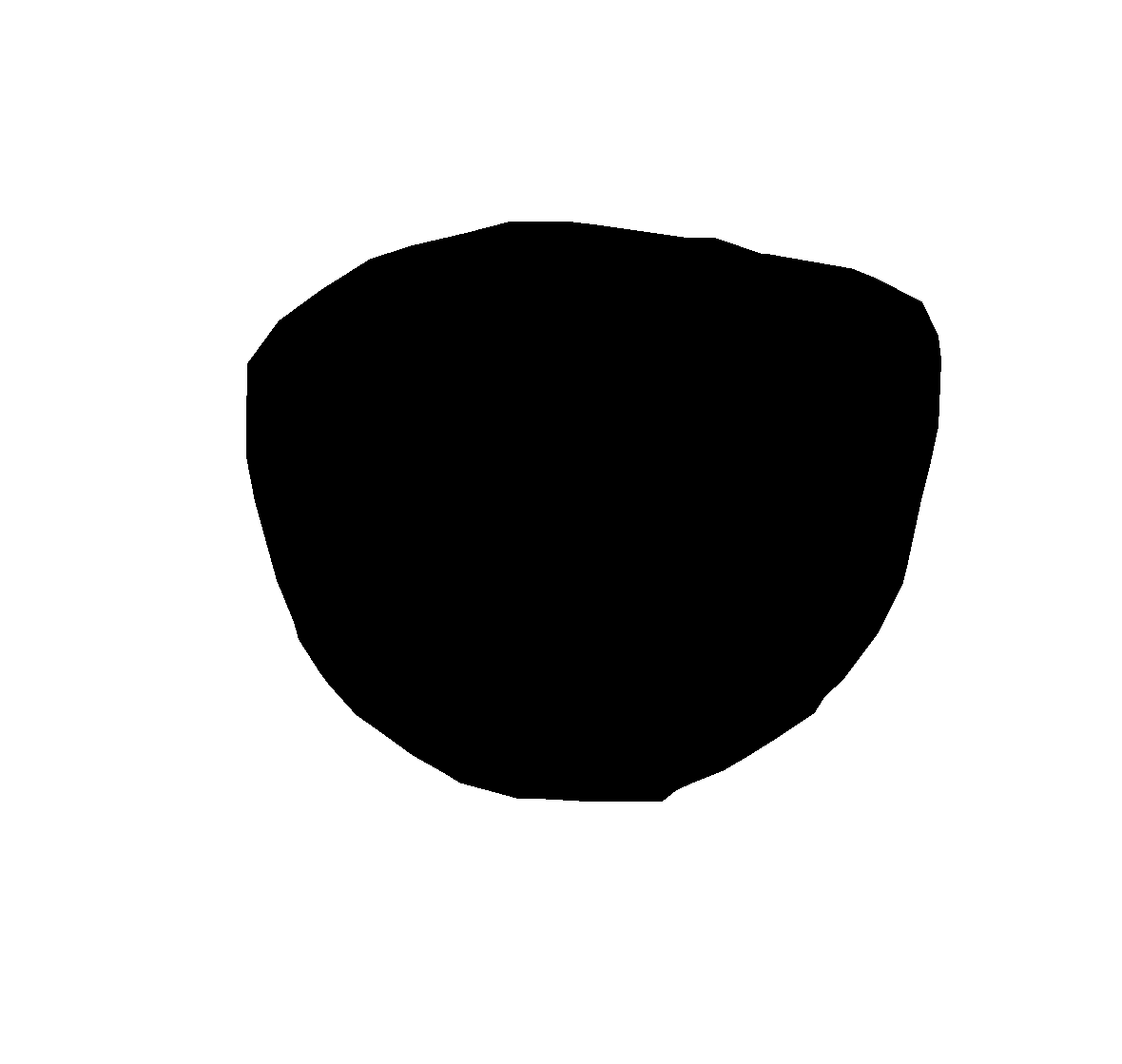

Supplement: Supplementary file 1 [file Data_Sheet_1.zip › tongue/062_tongue_mask.png]

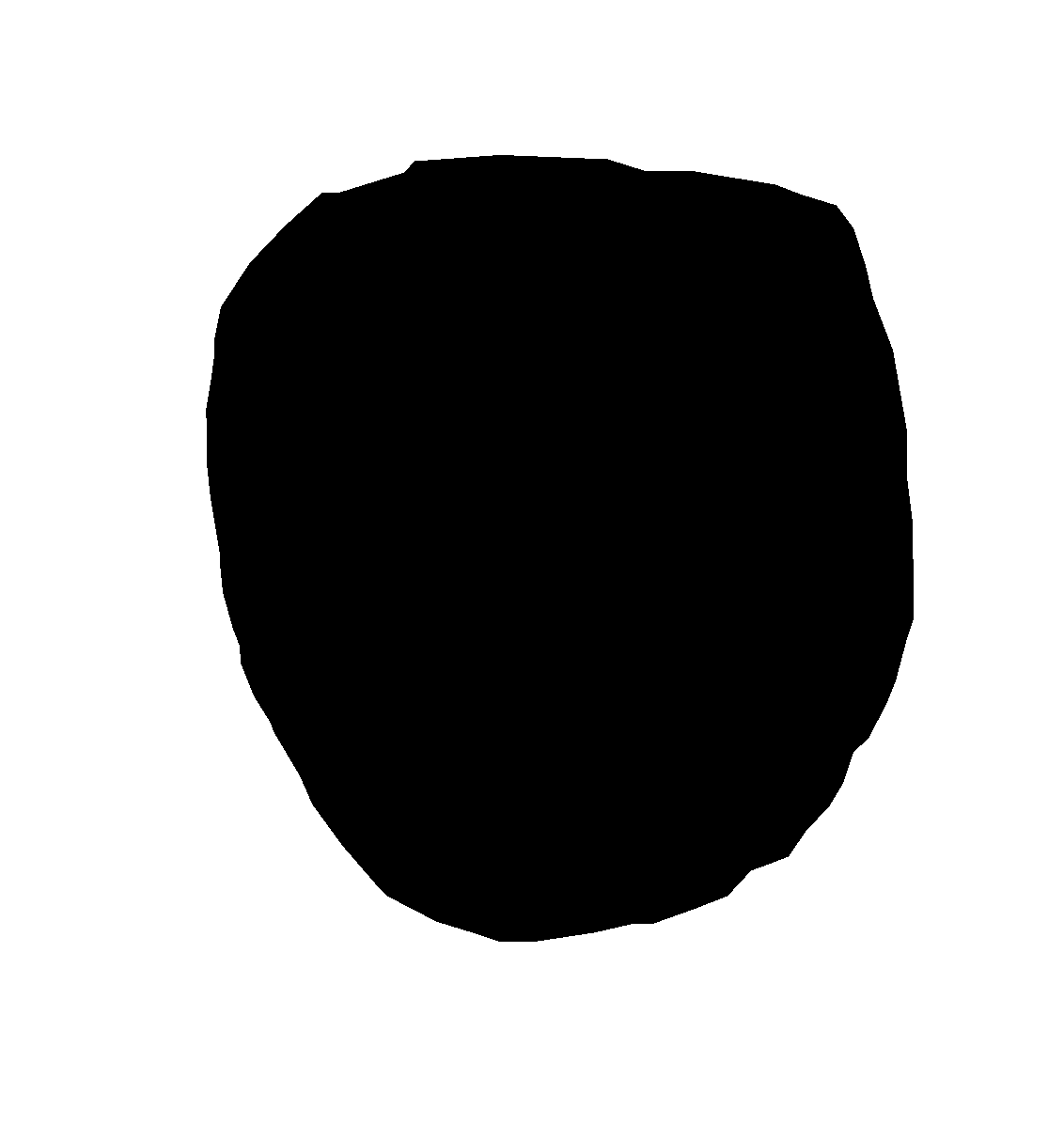

Supplement: Supplementary file 1 [file Data_Sheet_1.zip › tongue/063_tongue_mask.png]

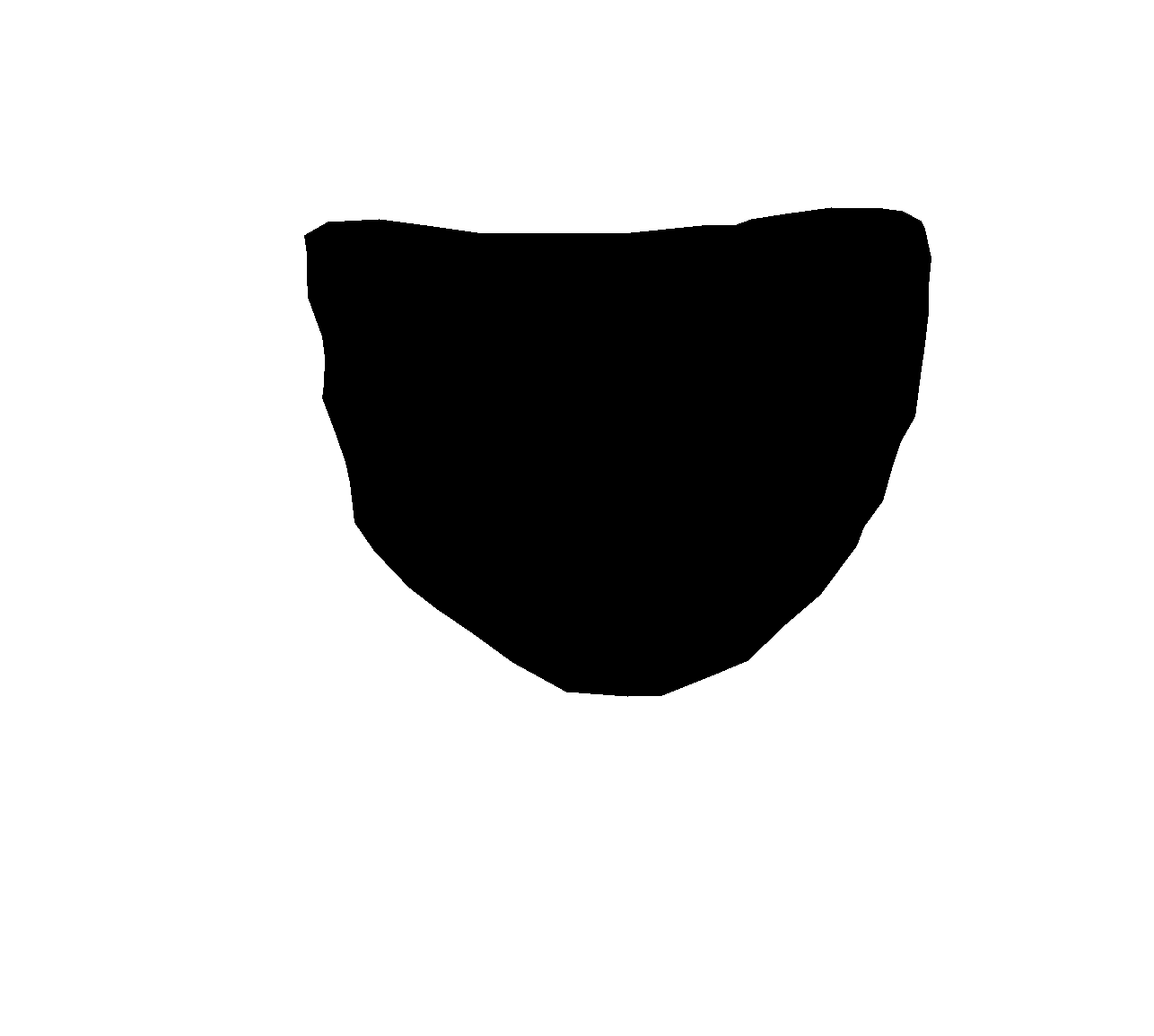

Supplement: Supplementary file 1 [file Data_Sheet_1.zip › tongue/064_tongue_mask.png]

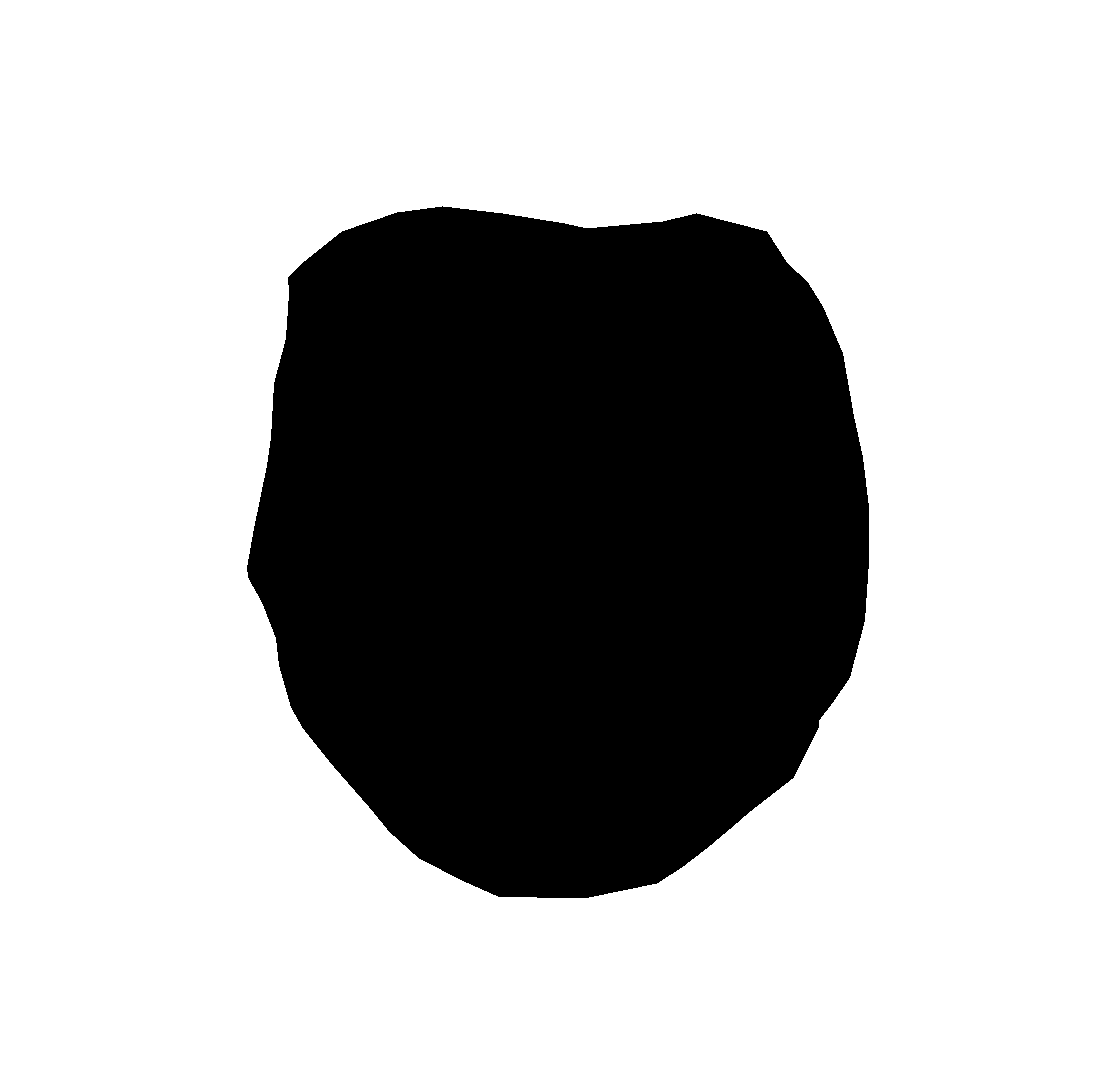

Supplement: Supplementary file 1 [file Data_Sheet_1.zip › tongue/065_tongue_mask.png]

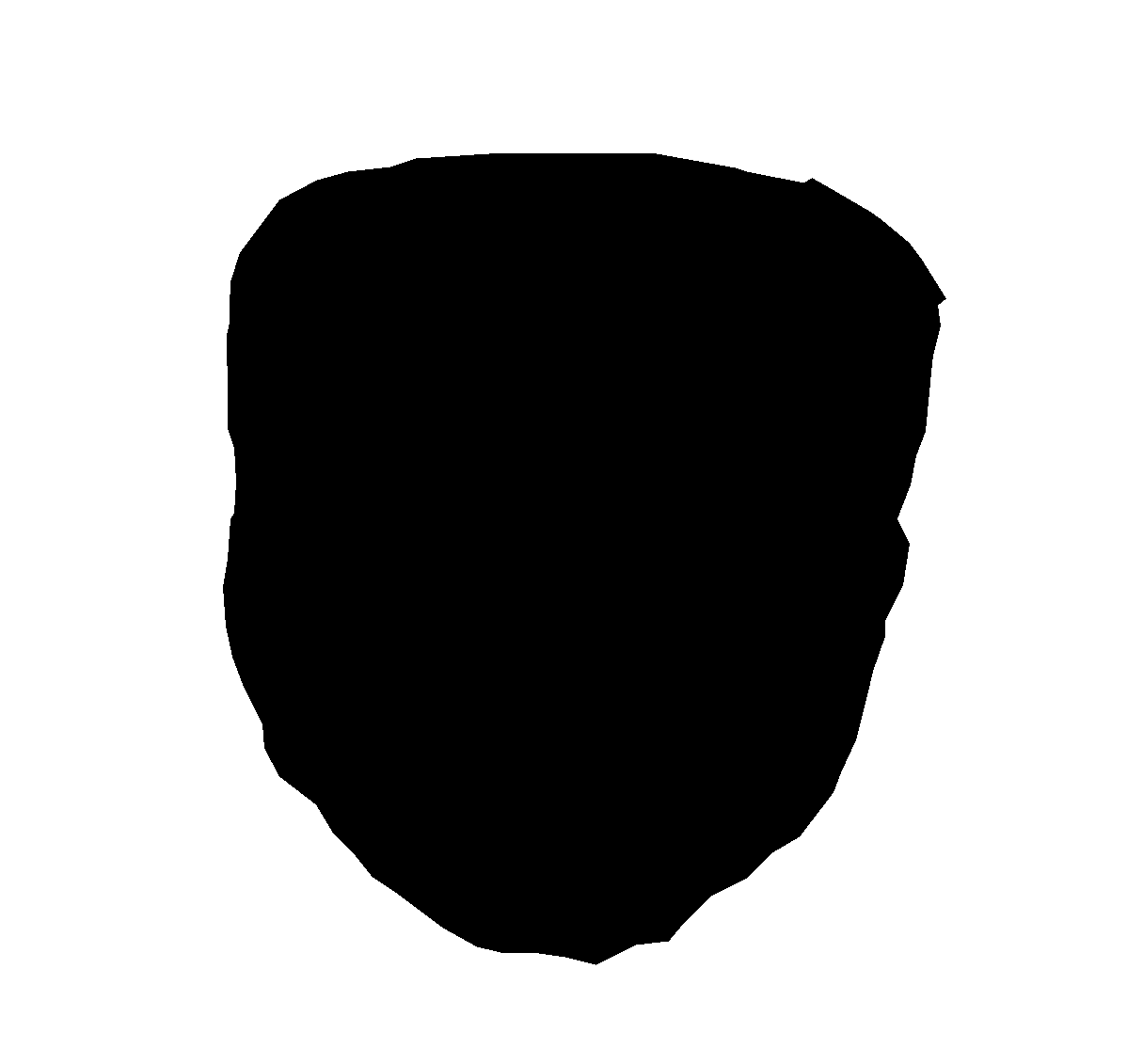

Supplement: Supplementary file 1 [file Data_Sheet_1.zip › tongue/066_tongue_mask.png]

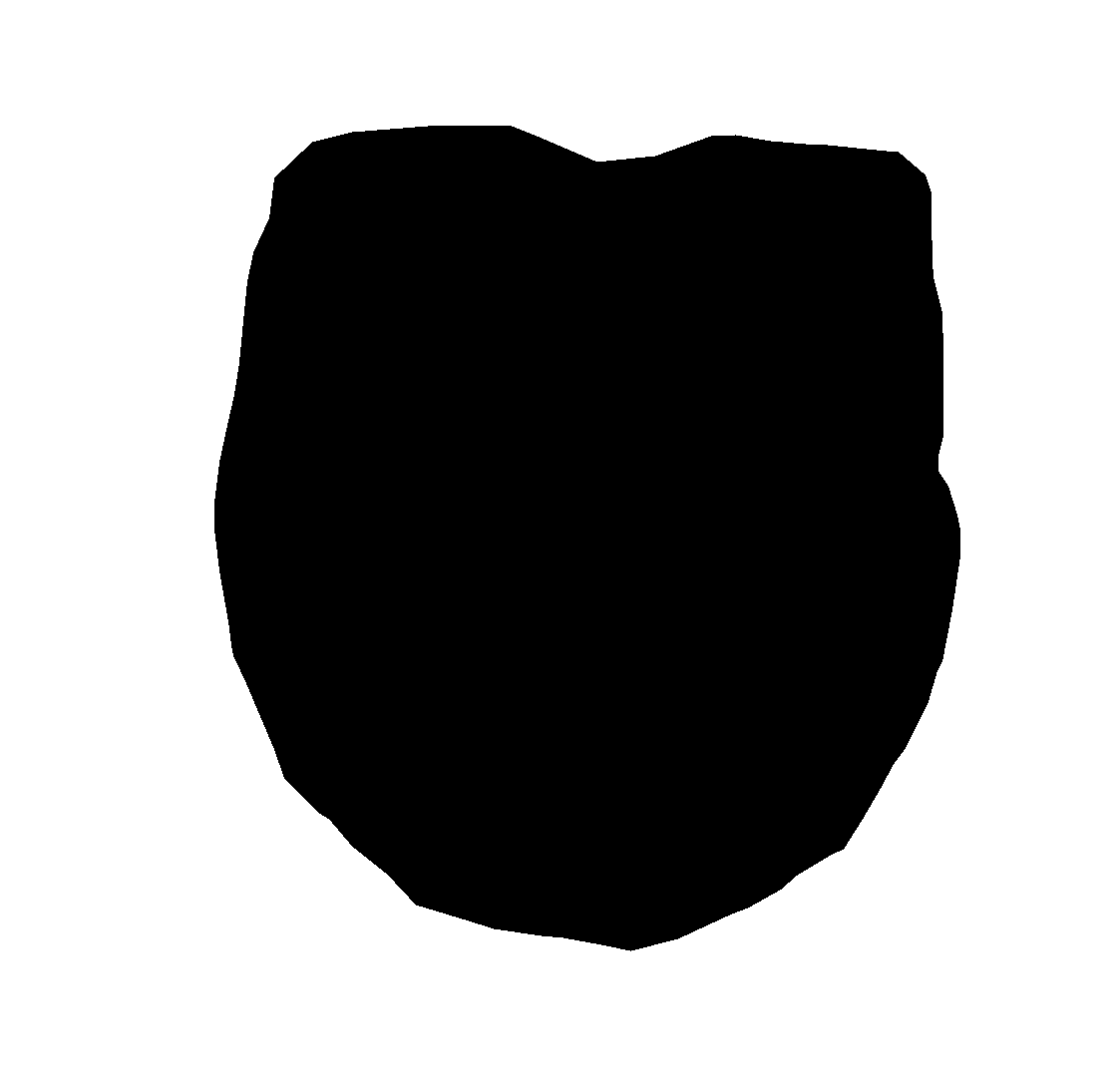

Supplement: Supplementary file 1 [file Data_Sheet_1.zip › tongue/067_tongue_mask.png]

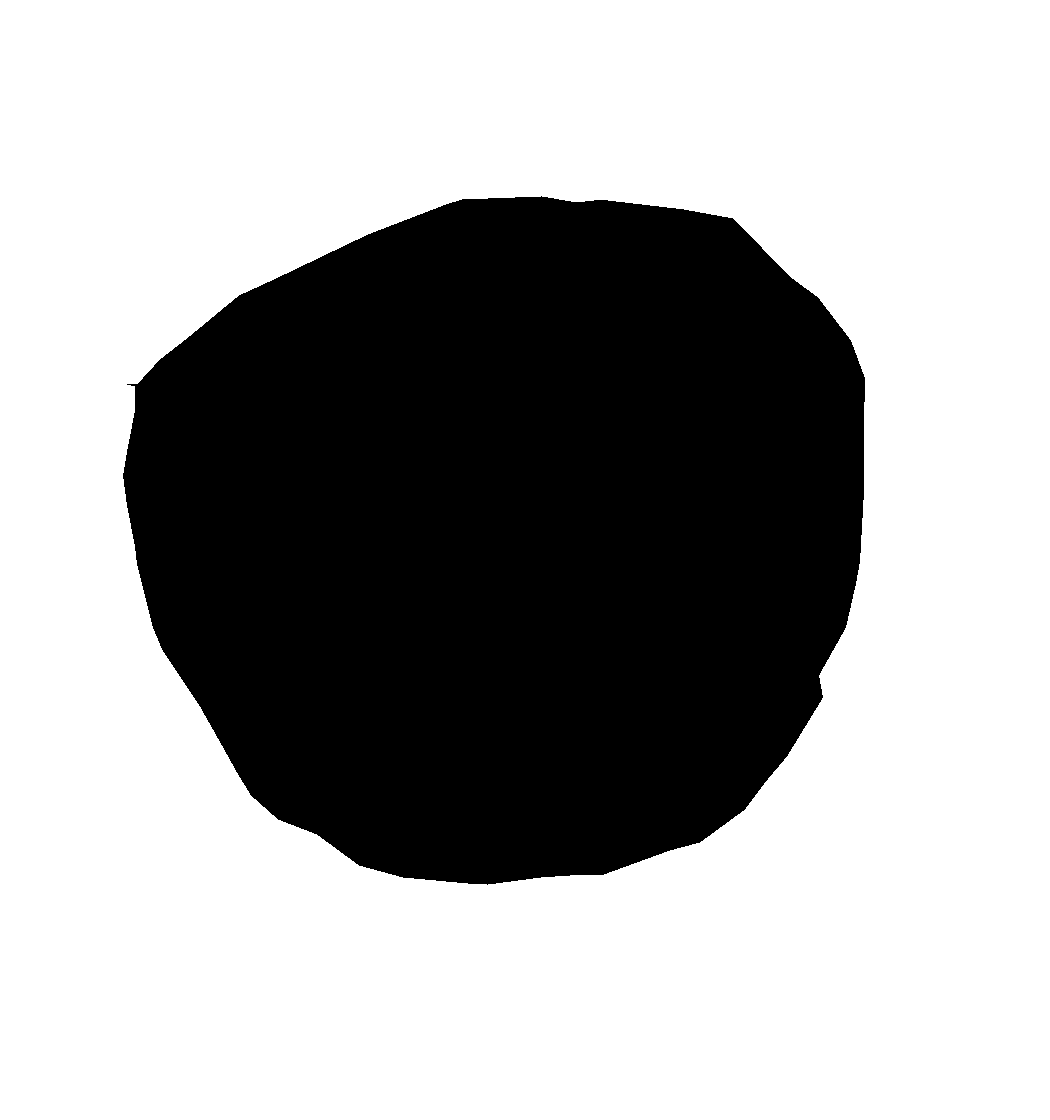

Supplement: Supplementary file 1 [file Data_Sheet_1.zip › tongue/068_tongue_mask.png]

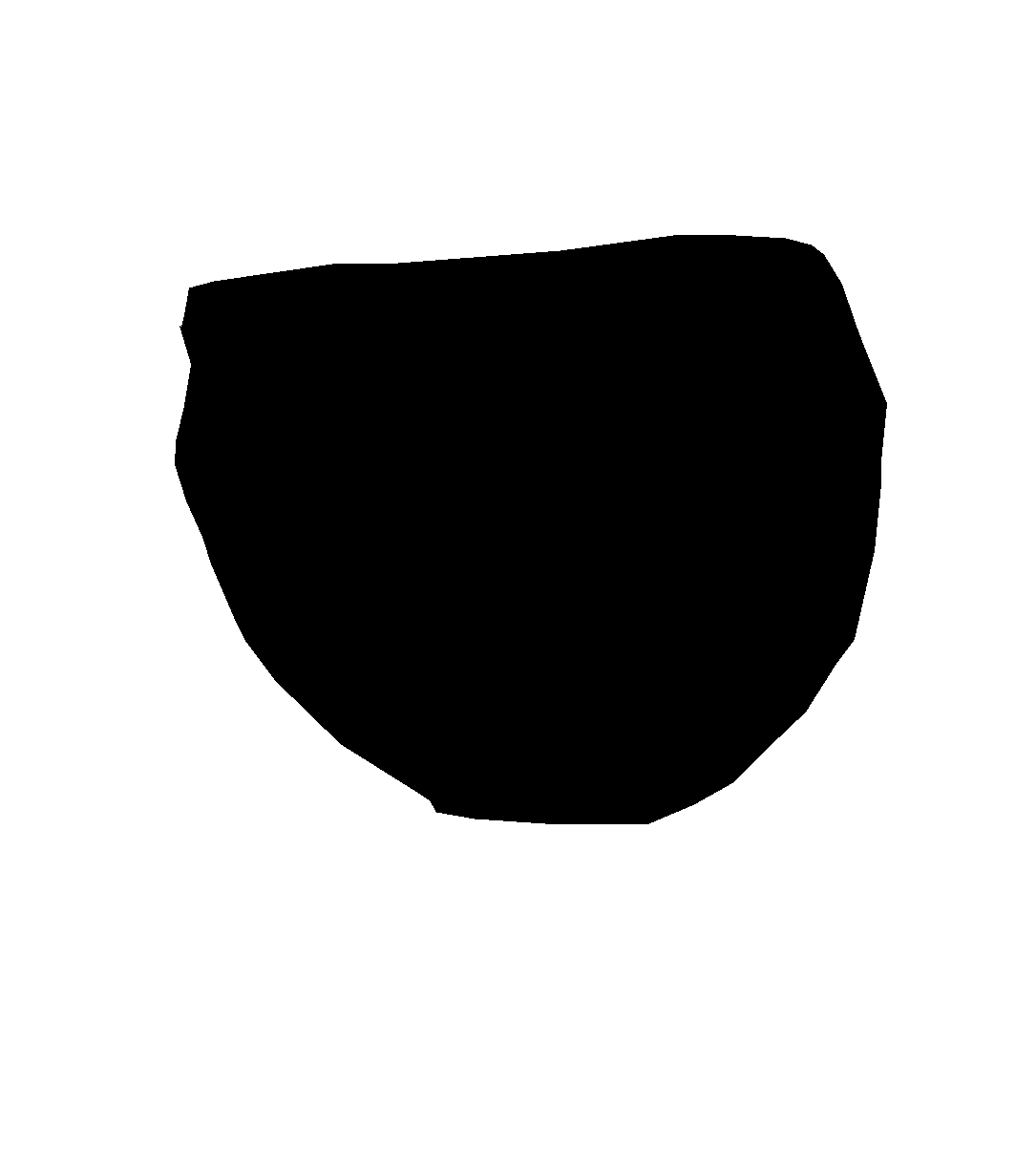

Supplement: Supplementary file 1 [file Data_Sheet_1.zip › tongue/069_tongue_mask.png]

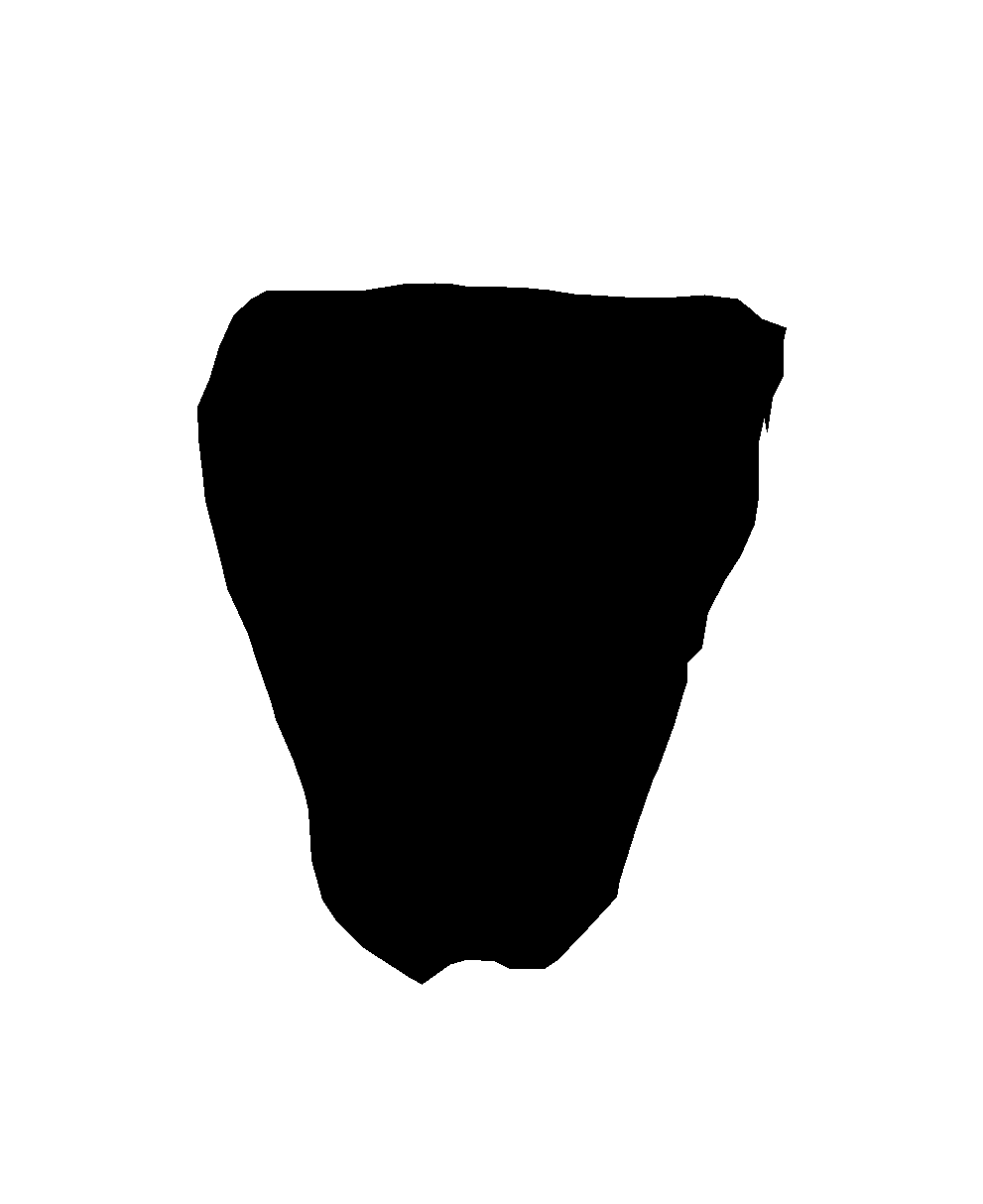

Supplement: Supplementary file 1 [file Data_Sheet_1.zip › tongue/070_tongue_mask.png]

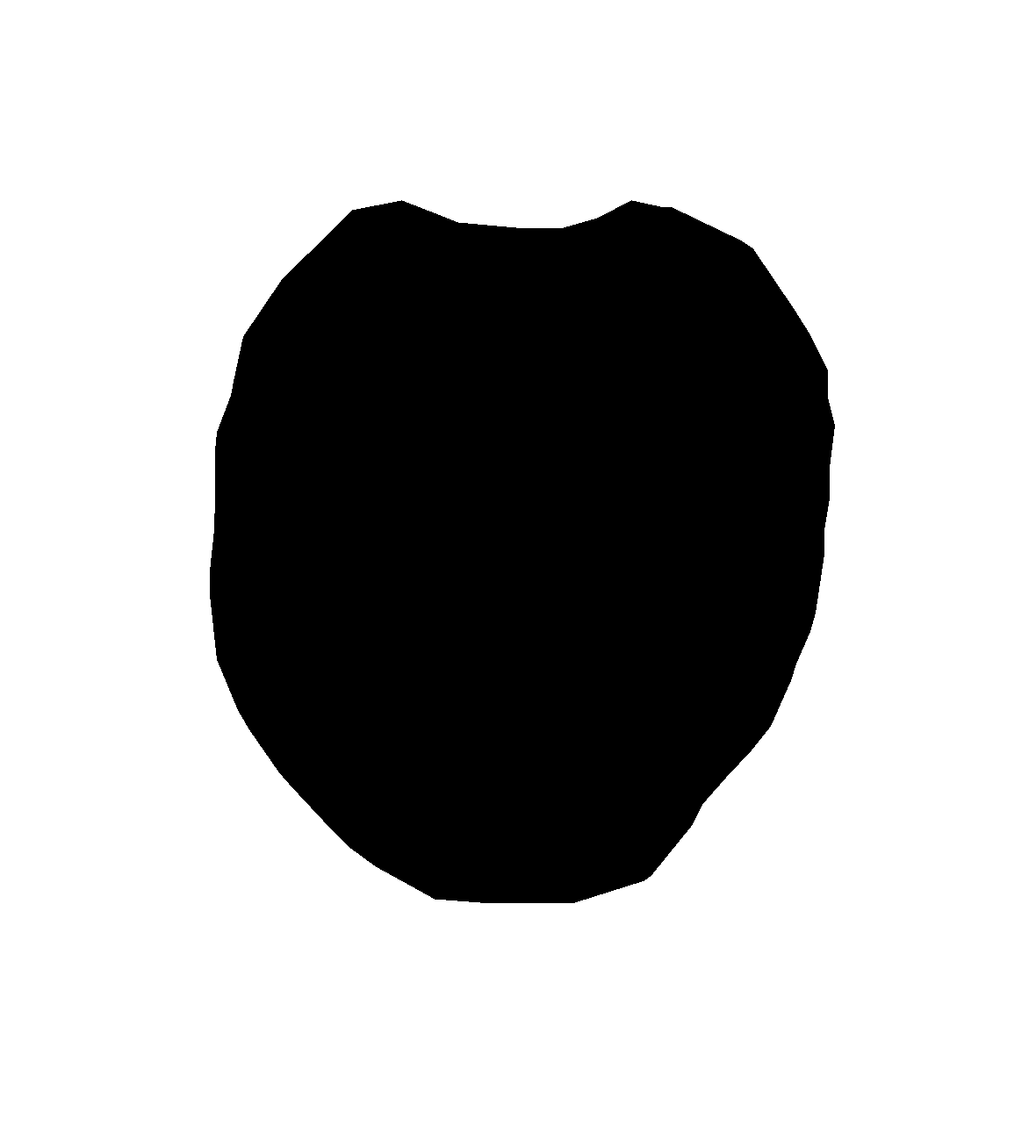

Supplement: Supplementary file 1 [file Data_Sheet_1.zip › tongue/071_tongue_mask.png]

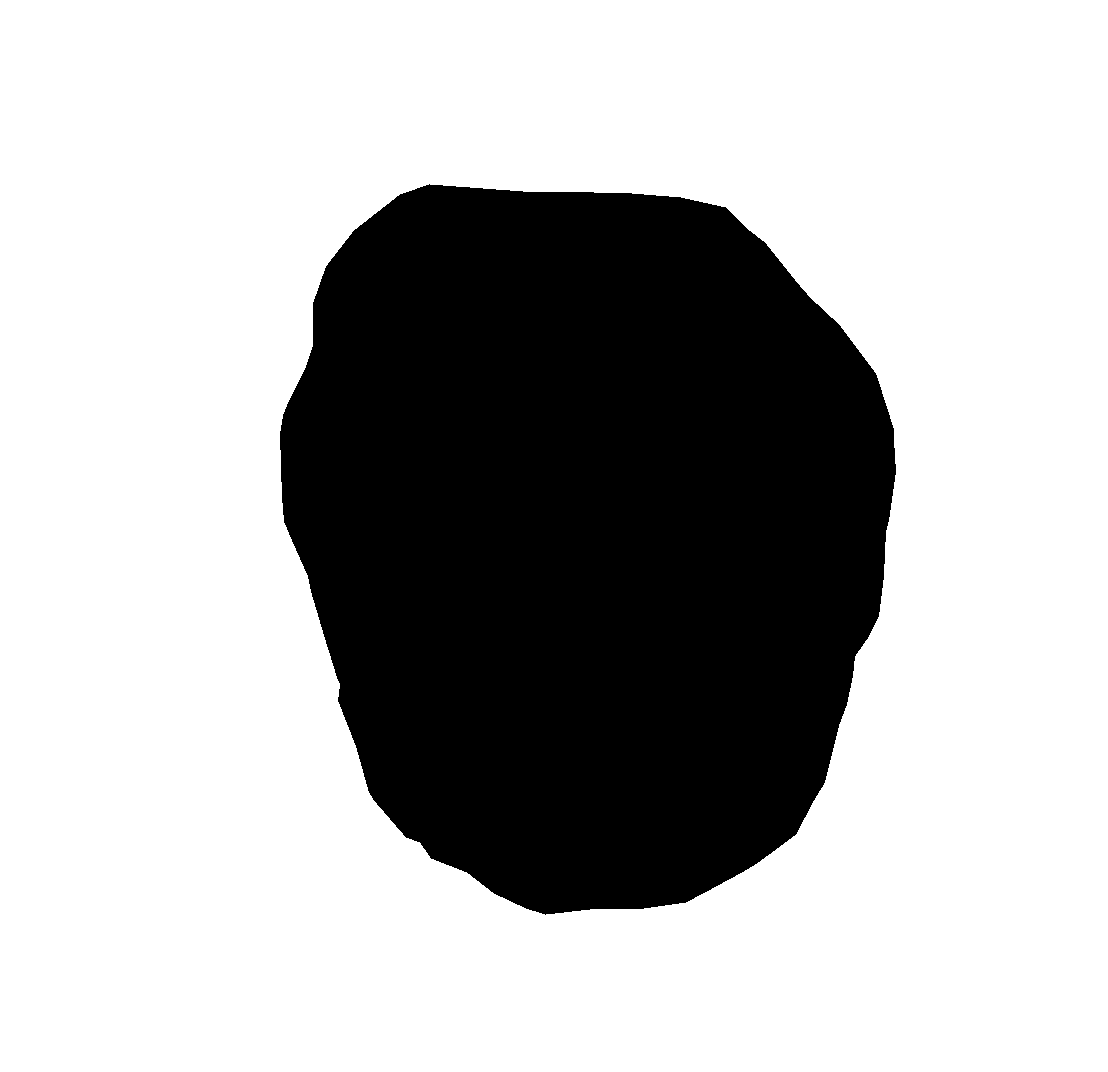

Supplement: Supplementary file 1 [file Data_Sheet_1.zip › tongue/072_tongue_mask.png]

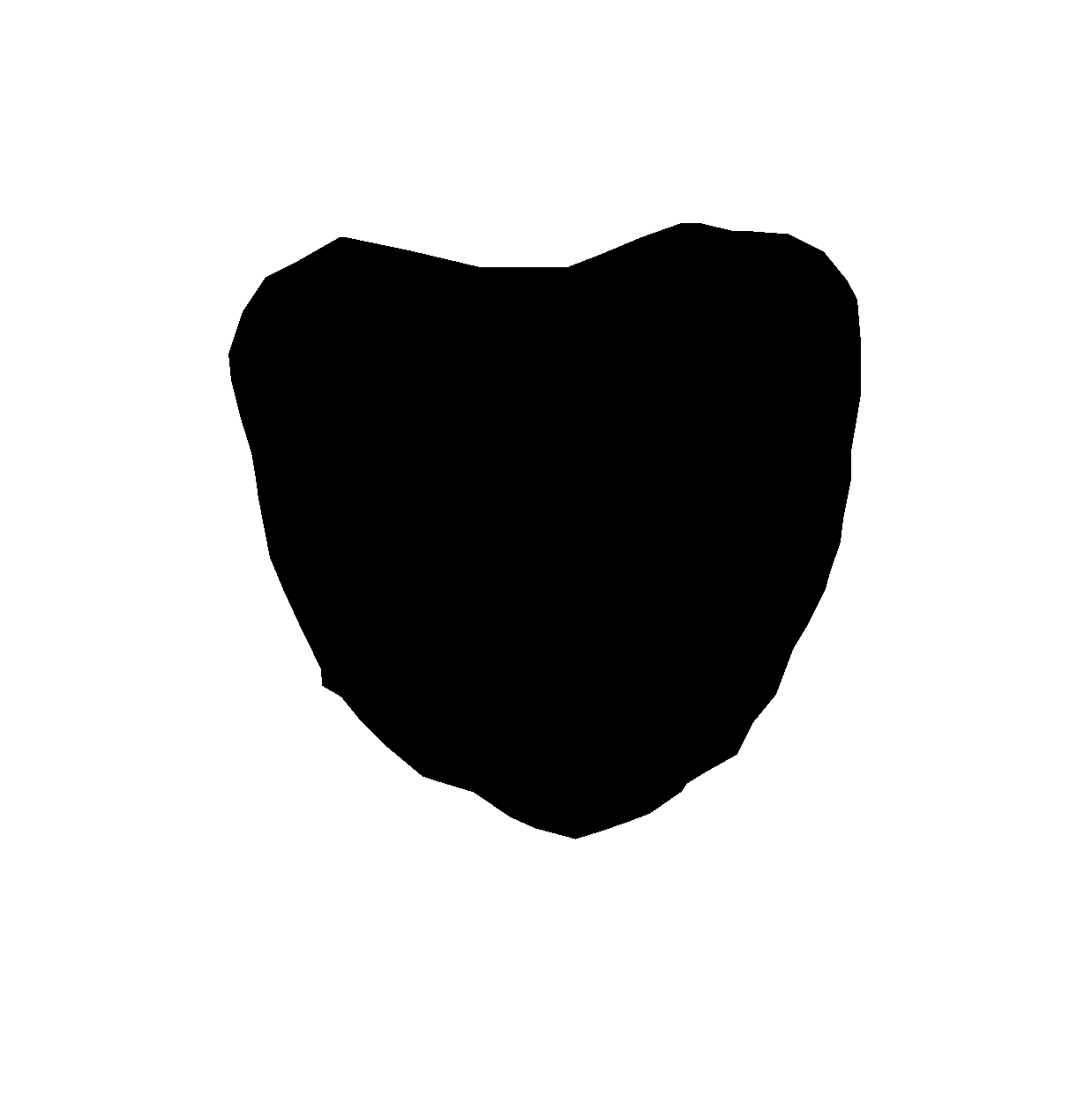

Supplement: Supplementary file 1 [file Data_Sheet_1.zip › tongue/073_tongue_mask.png]

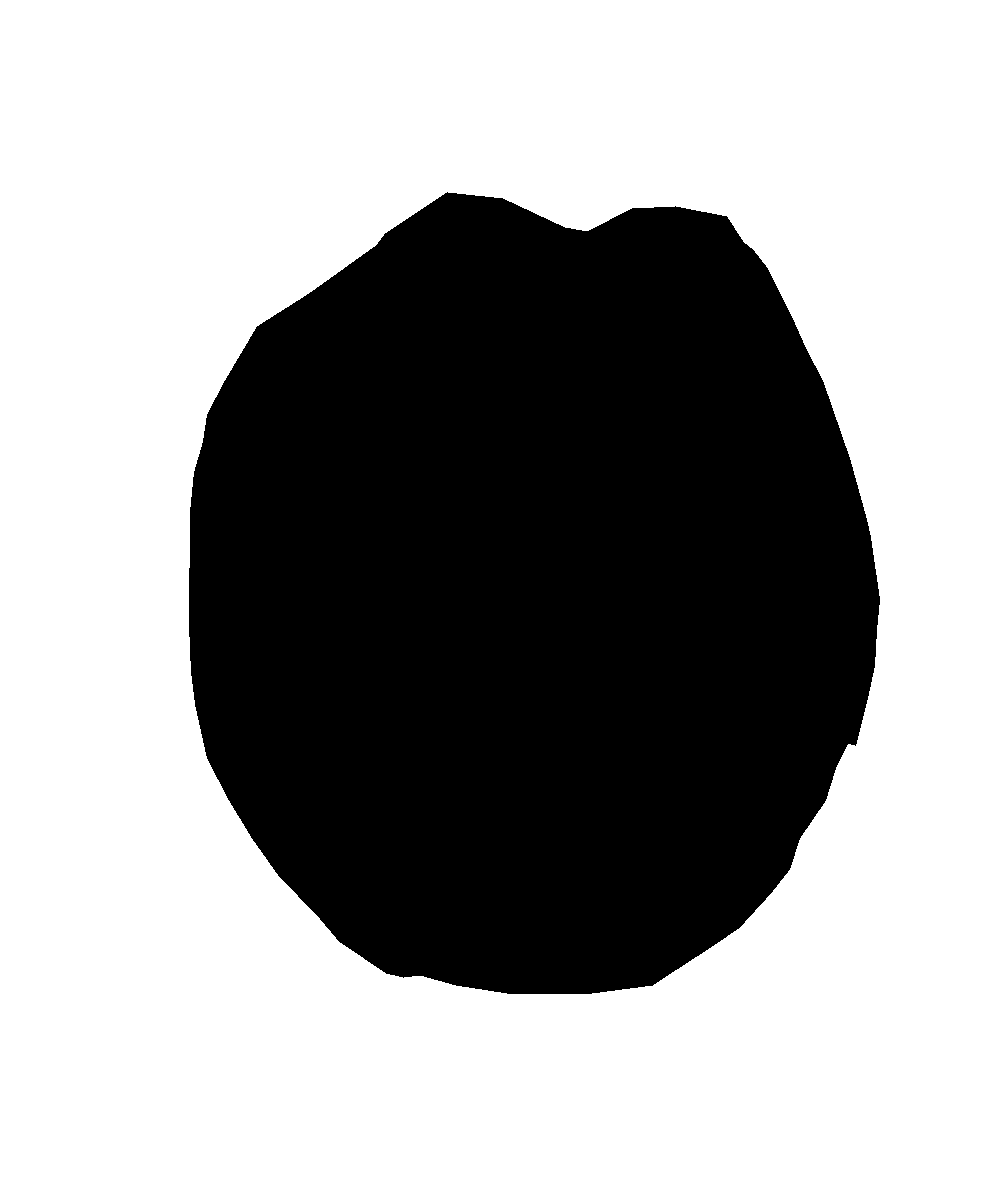

Supplement: Supplementary file 1 [file Data_Sheet_1.zip › tongue/074_tongue_mask.png]

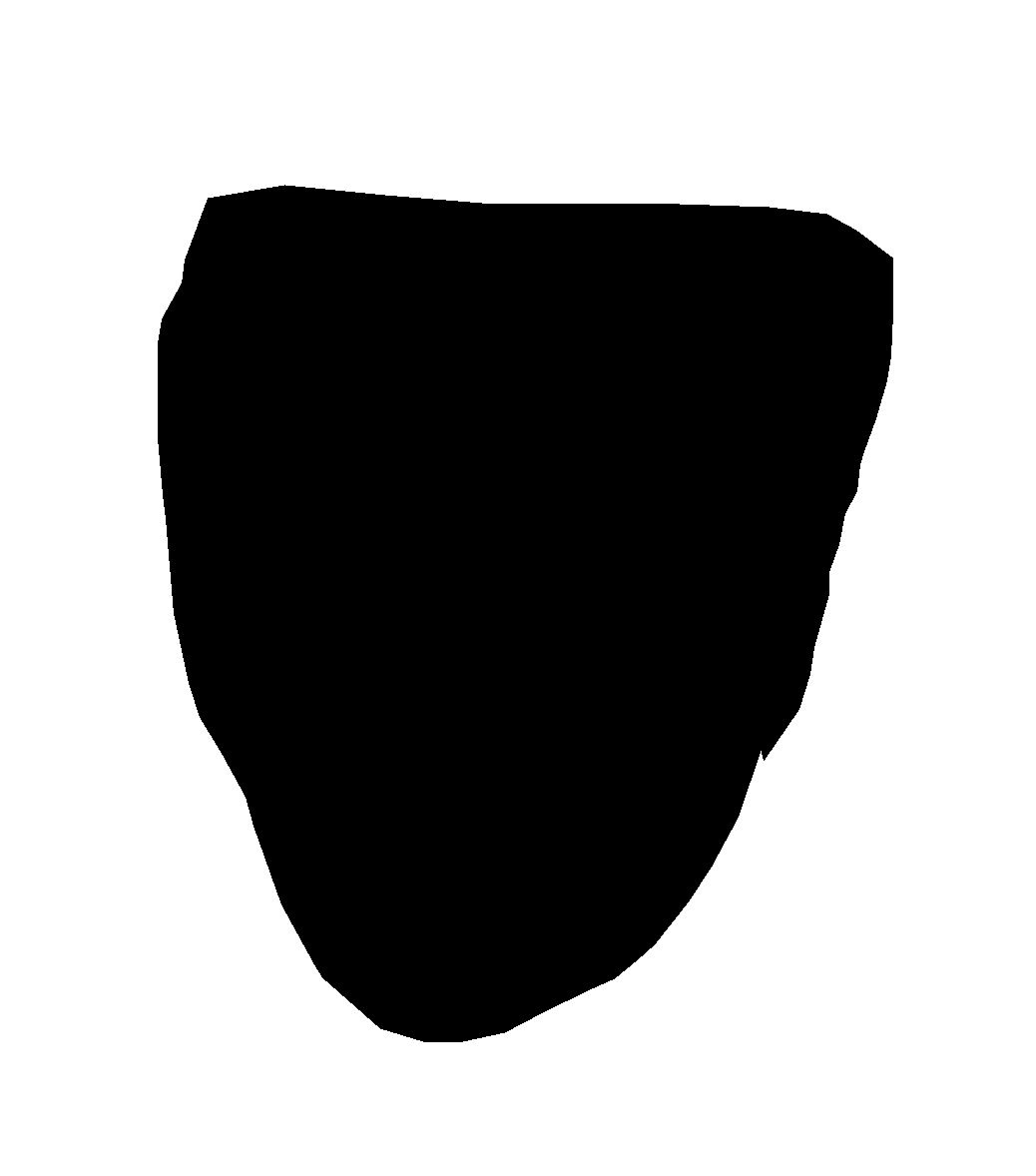

Supplement: Supplementary file 1 [file Data_Sheet_1.zip › tongue/075_tongue_mask.png]

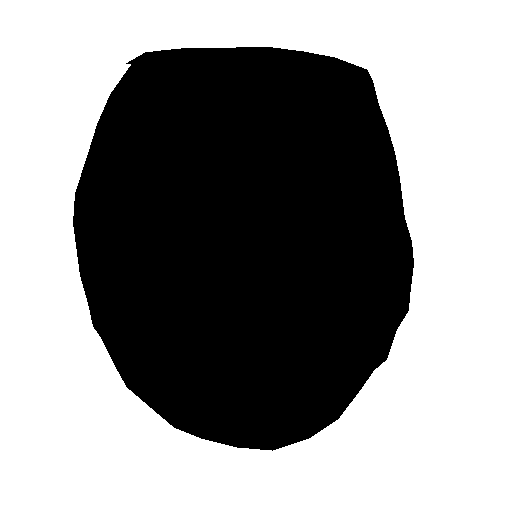

Supplement: Supplementary file 1 [file Data_Sheet_1.zip › tongue/076_tongue_mask.png]

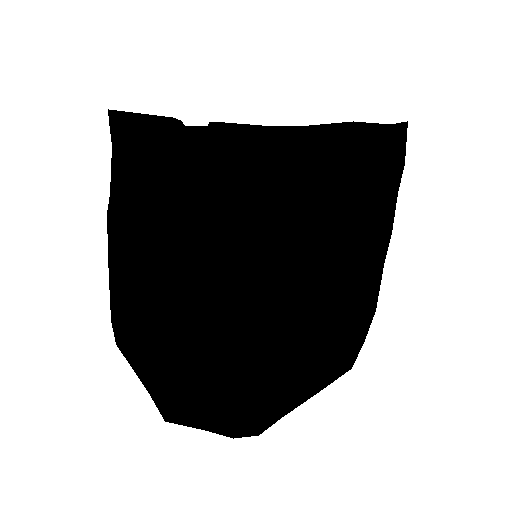

Supplement: Supplementary file 1 [file Data_Sheet_1.zip › tongue/077_tongue_mask.png]

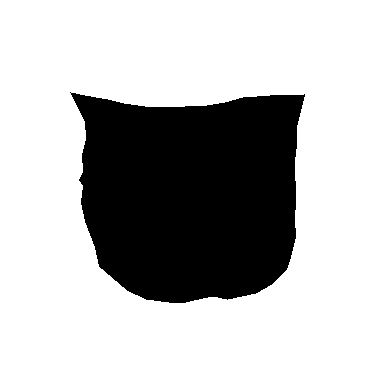

Supplement: Supplementary file 1 [file Data_Sheet_1.zip › tongue/078_tongue_mask.png]

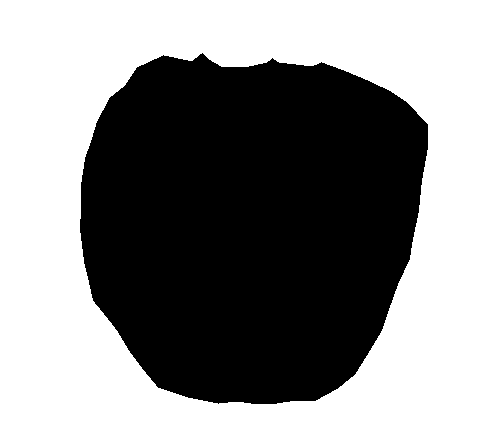

Supplement: Supplementary file 1 [file Data_Sheet_1.zip › tongue/079_tongue_mask.png]

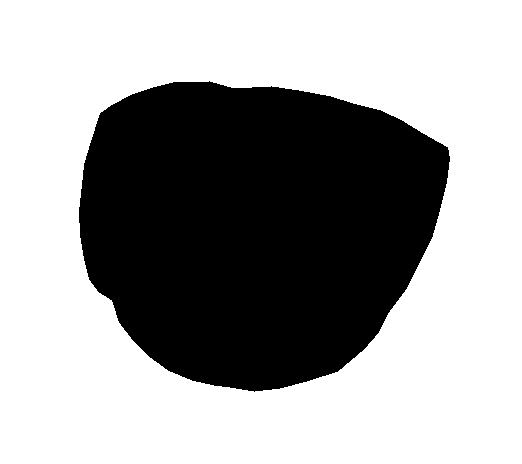

Supplement: Supplementary file 1 [file Data_Sheet_1.zip › tongue/080_tongue_mask.png]

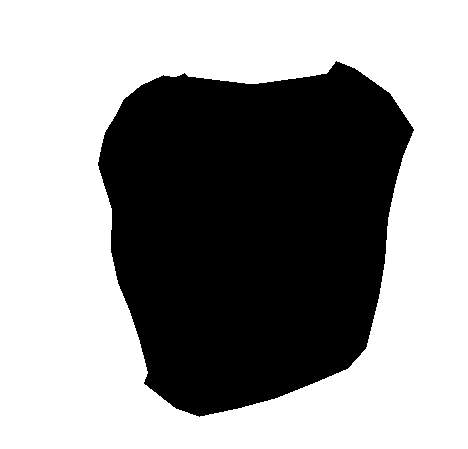

Supplement: Supplementary file 1 [file Data_Sheet_1.zip › tongue/081_tongue_mask.png]

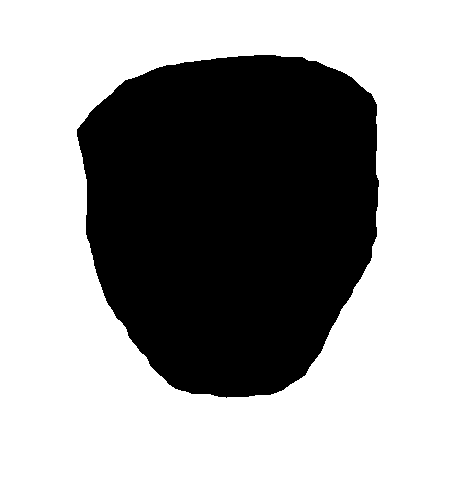

Supplement: Supplementary file 1 [file Data_Sheet_1.zip › tongue/082_tongue_mask.png]

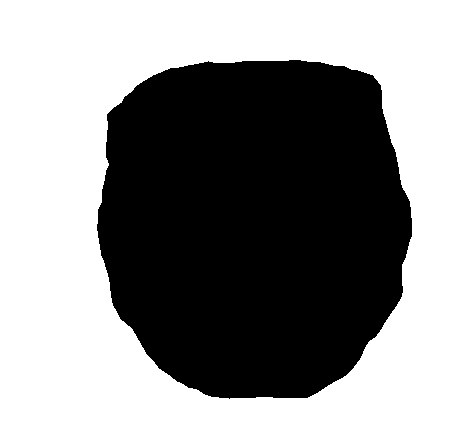

Supplement: Supplementary file 1 [file Data_Sheet_1.zip › tongue/083_tongue_mask.png]

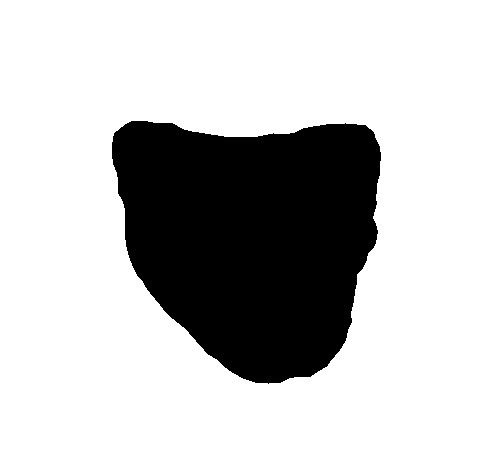

Supplement: Supplementary file 1 [file Data_Sheet_1.zip › tongue/084_tongue_mask.png]

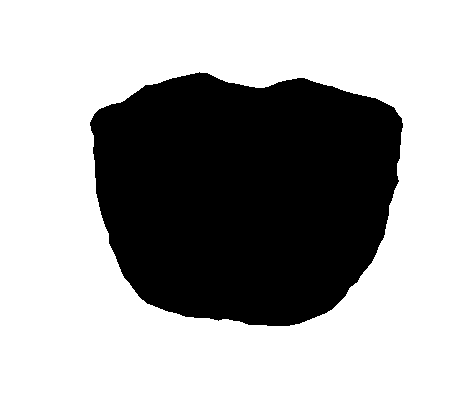

Supplement: Supplementary file 1 [file Data_Sheet_1.zip › tongue/085_tongue_mask.png]

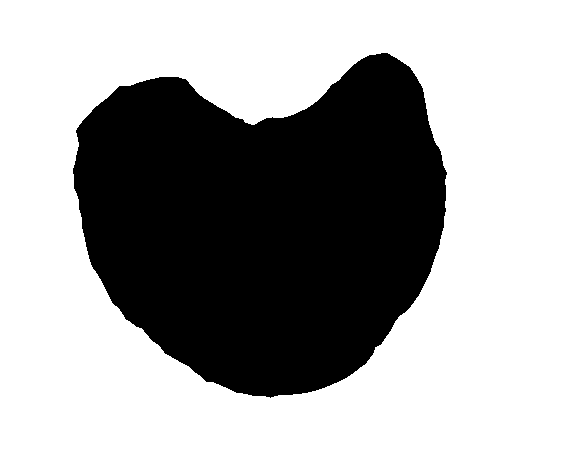

Supplement: Supplementary file 1 [file Data_Sheet_1.zip › tongue/086_tongue_mask.png]

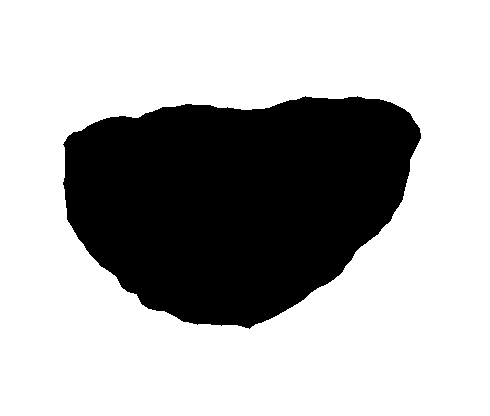

Supplement: Supplementary file 1 [file Data_Sheet_1.zip › tongue/087_tongue_mask.png]

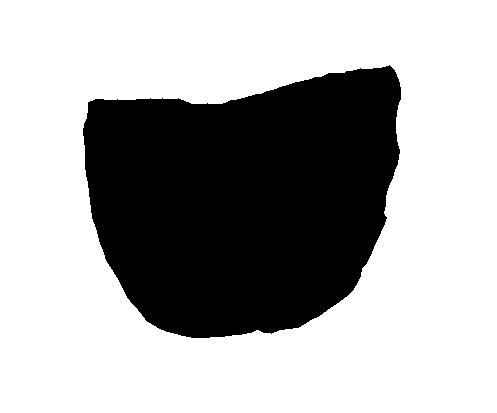

Supplement: Supplementary file 1 [file Data_Sheet_1.zip › tongue/088_tongue_mask.png]

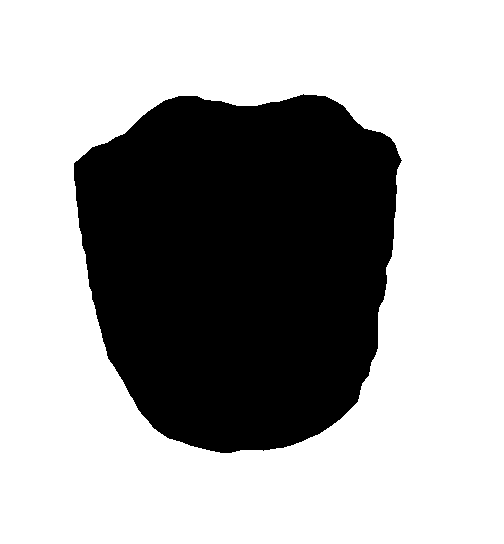

Supplement: Supplementary file 1 [file Data_Sheet_1.zip › tongue/089_tongue_mask.png]

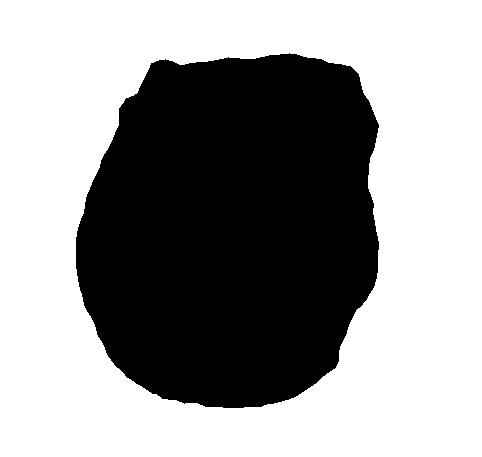

Supplement: Supplementary file 1 [file Data_Sheet_1.zip › tongue/090_tongue_mask.png]

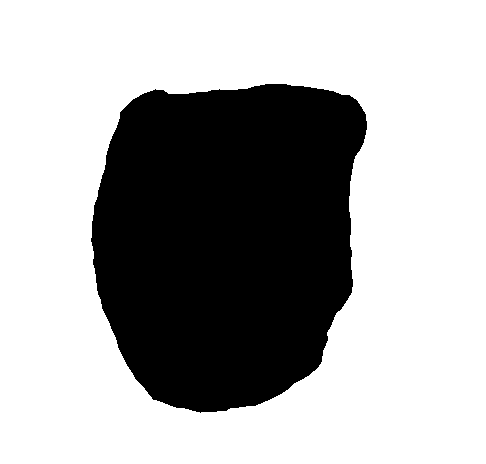

Supplement: Supplementary file 1 [file Data_Sheet_1.zip › tongue/091_tongue_mask.png]

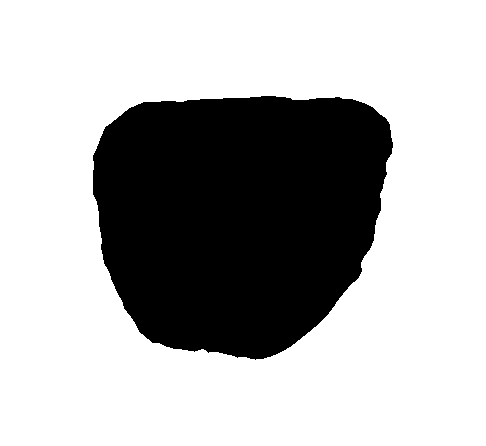

Supplement: Supplementary file 1 [file Data_Sheet_1.zip › tongue/092_tongue_mask.png]

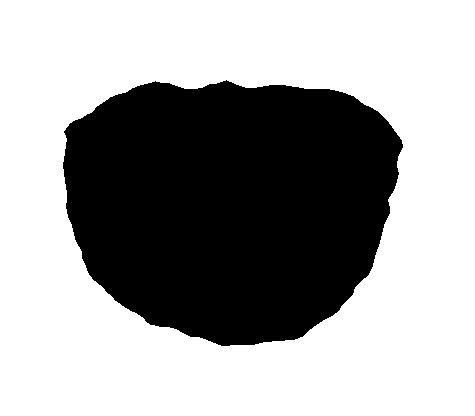

Supplement: Supplementary file 1 [file Data_Sheet_1.zip › tongue/093_tongue_mask.png]

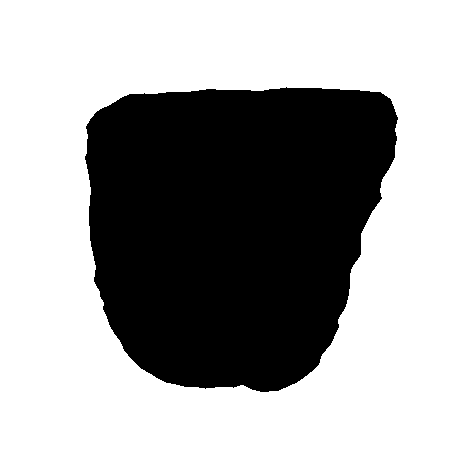

Supplement: Supplementary file 1 [file Data_Sheet_1.zip › tongue/094_tongue_mask.png]

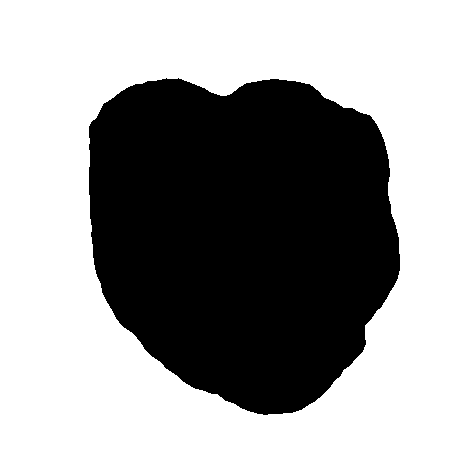

Supplement: Supplementary file 1 [file Data_Sheet_1.zip › tongue/095_tongue_mask.png]

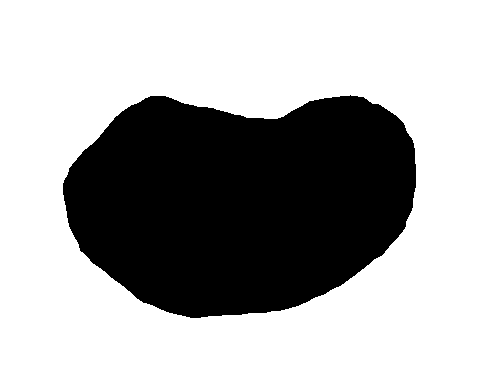

Supplement: Supplementary file 1 [file Data_Sheet_1.zip › tongue/096_tongue_mask.png]

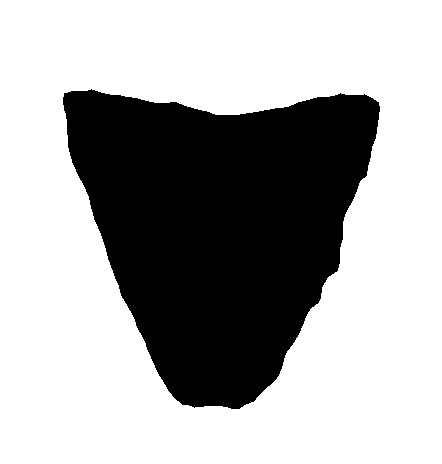

Supplement: Supplementary file 1 [file Data_Sheet_1.zip › tongue/097_tongue_mask.png]

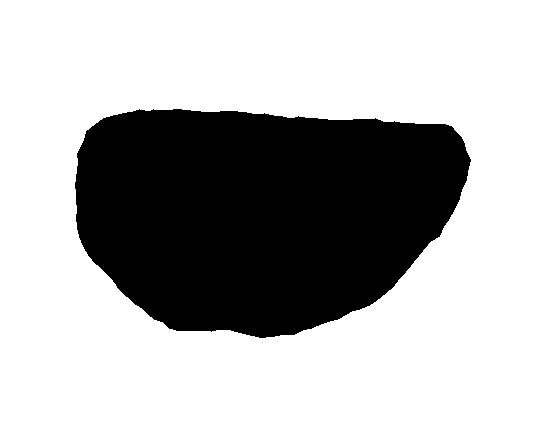

Supplement: Supplementary file 1 [file Data_Sheet_1.zip › tongue/098_tongue_mask.png]

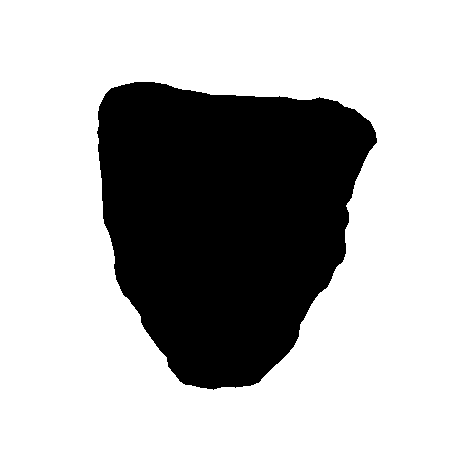

Supplement: Supplementary file 1 [file Data_Sheet_1.zip › tongue/099_tongue_mask.png]

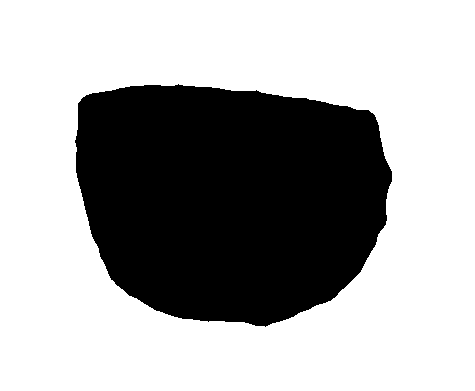

Supplement: Supplementary file 1 [file Data_Sheet_1.zip › tongue/100_tongue_mask.png]
