# Supplementary figures and images for: An explainable dual-modal diagnostic model for coronary artery disease: a feature-gated approach using tongue and facial image features (part 1 of 3)
Source: Front Artif Intell. 2025 Nov 17;8:1662577. doi: 10.3389/frai.2025.1662577 (PMC12665729; doi:10.3389/frai.2025.1662577)

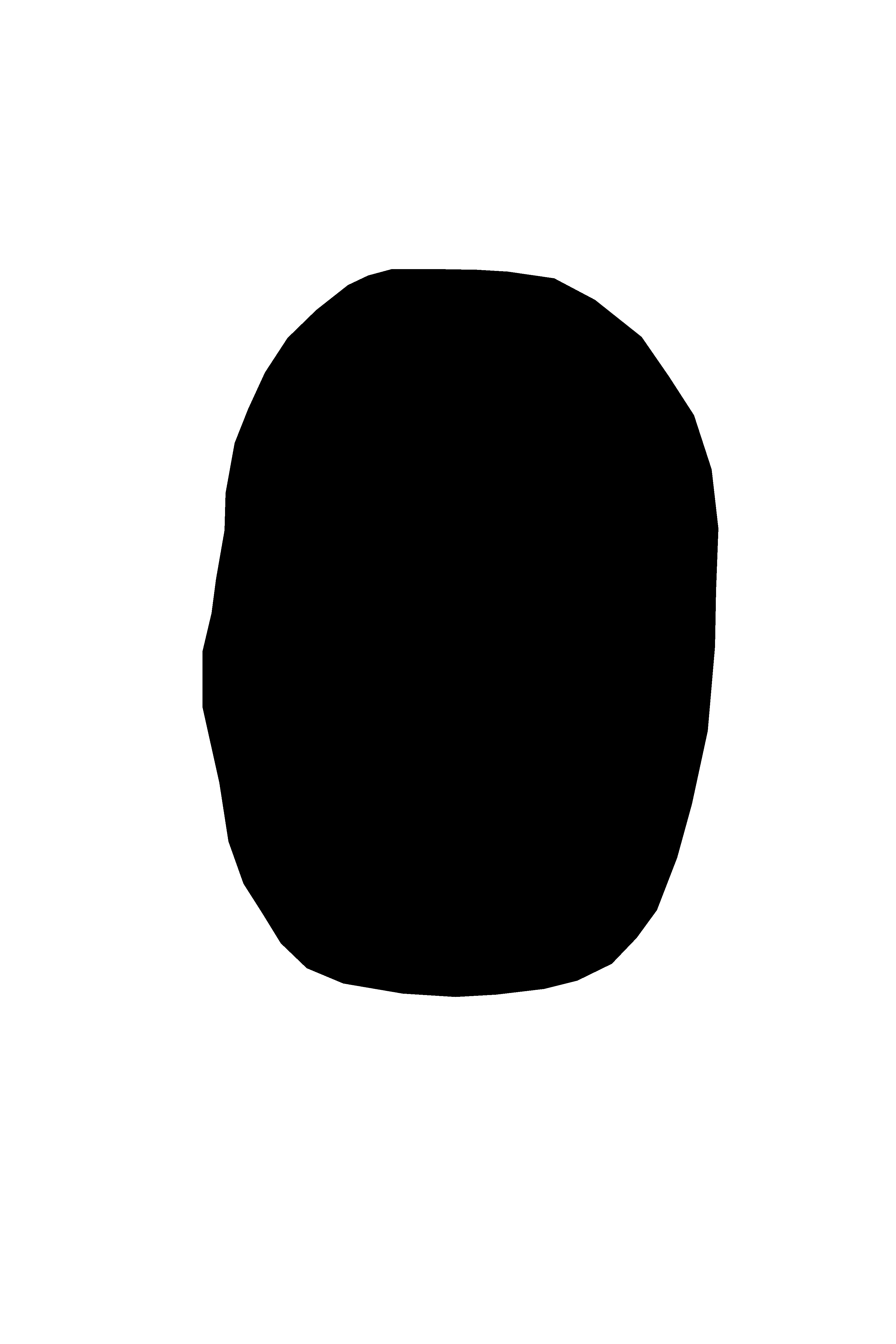

Supplement: Supplementary file 1 [file Data_Sheet_1.zip › face/001_face_mask.png]

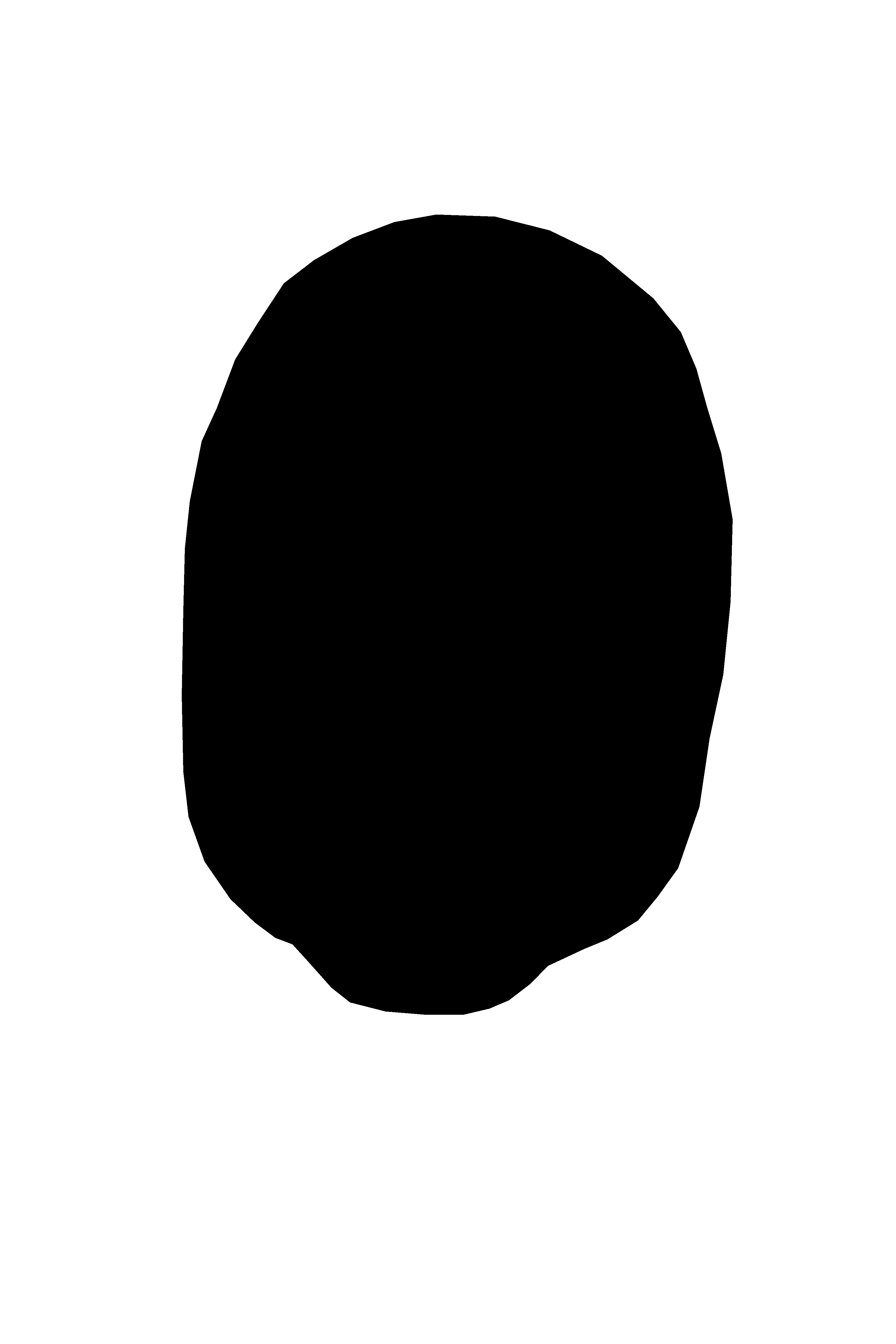

Supplement: Supplementary file 1 [file Data_Sheet_1.zip › face/002_face_mask.png]

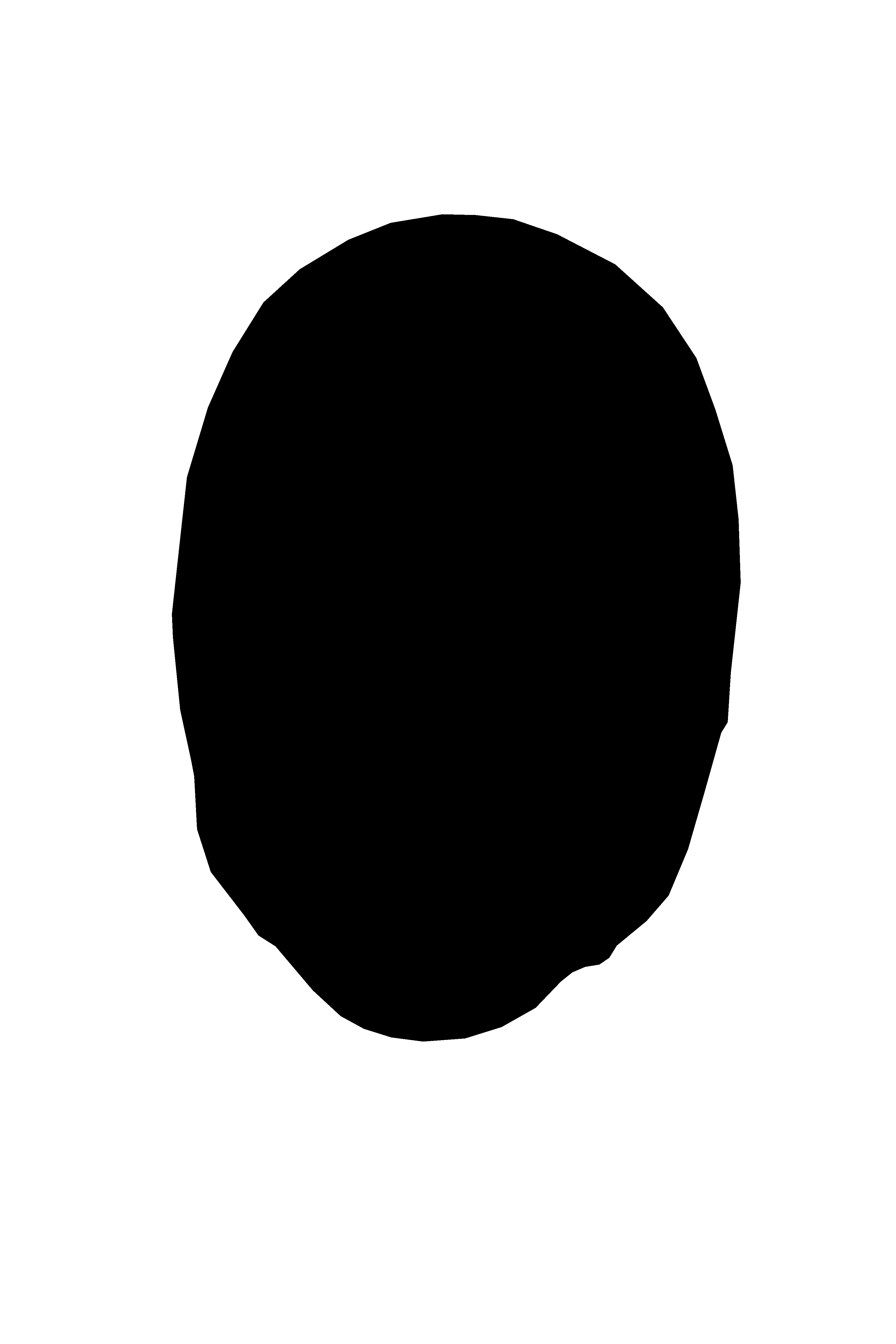

Supplement: Supplementary file 1 [file Data_Sheet_1.zip › face/003_face_mask.png]

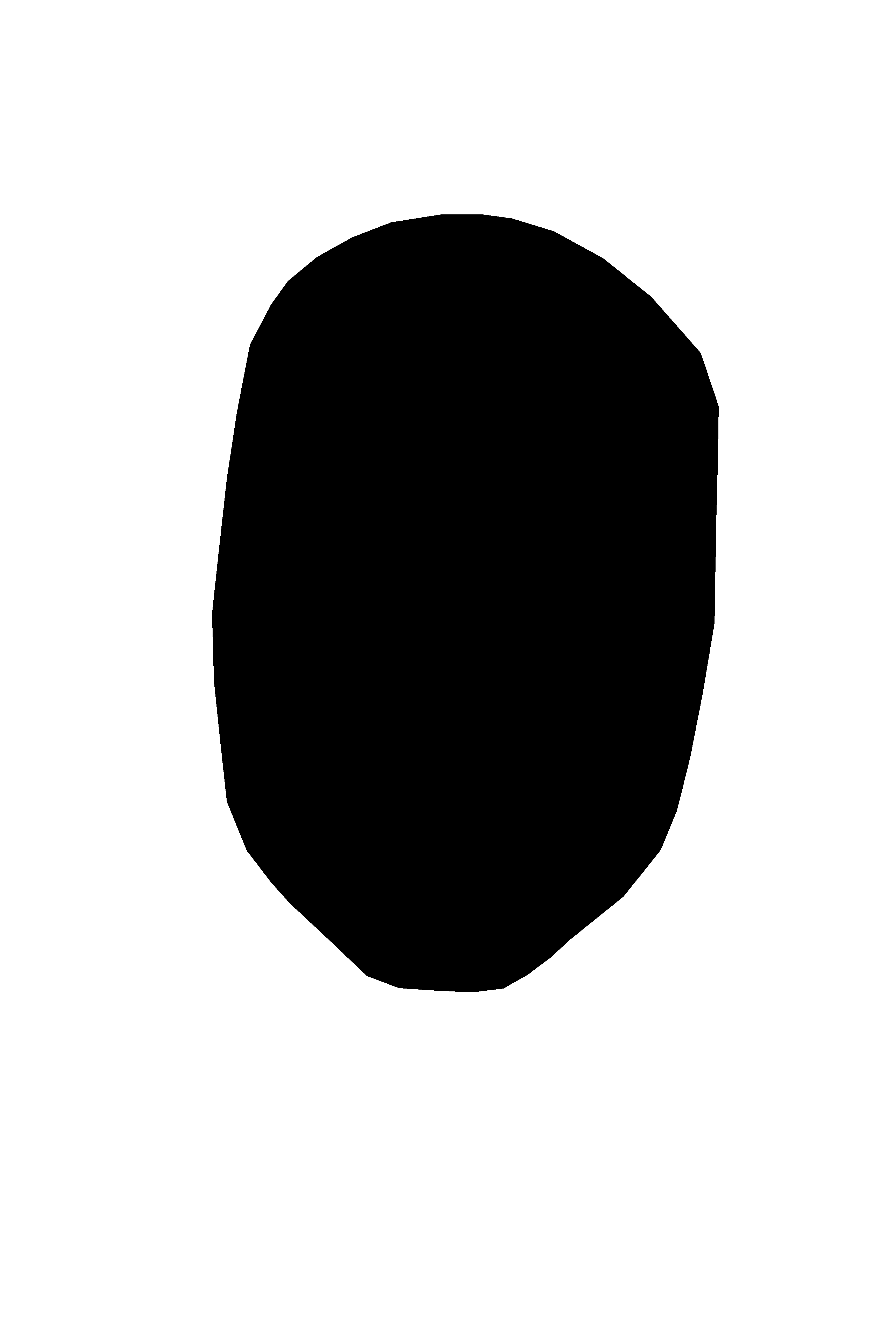

Supplement: Supplementary file 1 [file Data_Sheet_1.zip › face/004_face_mask.png]

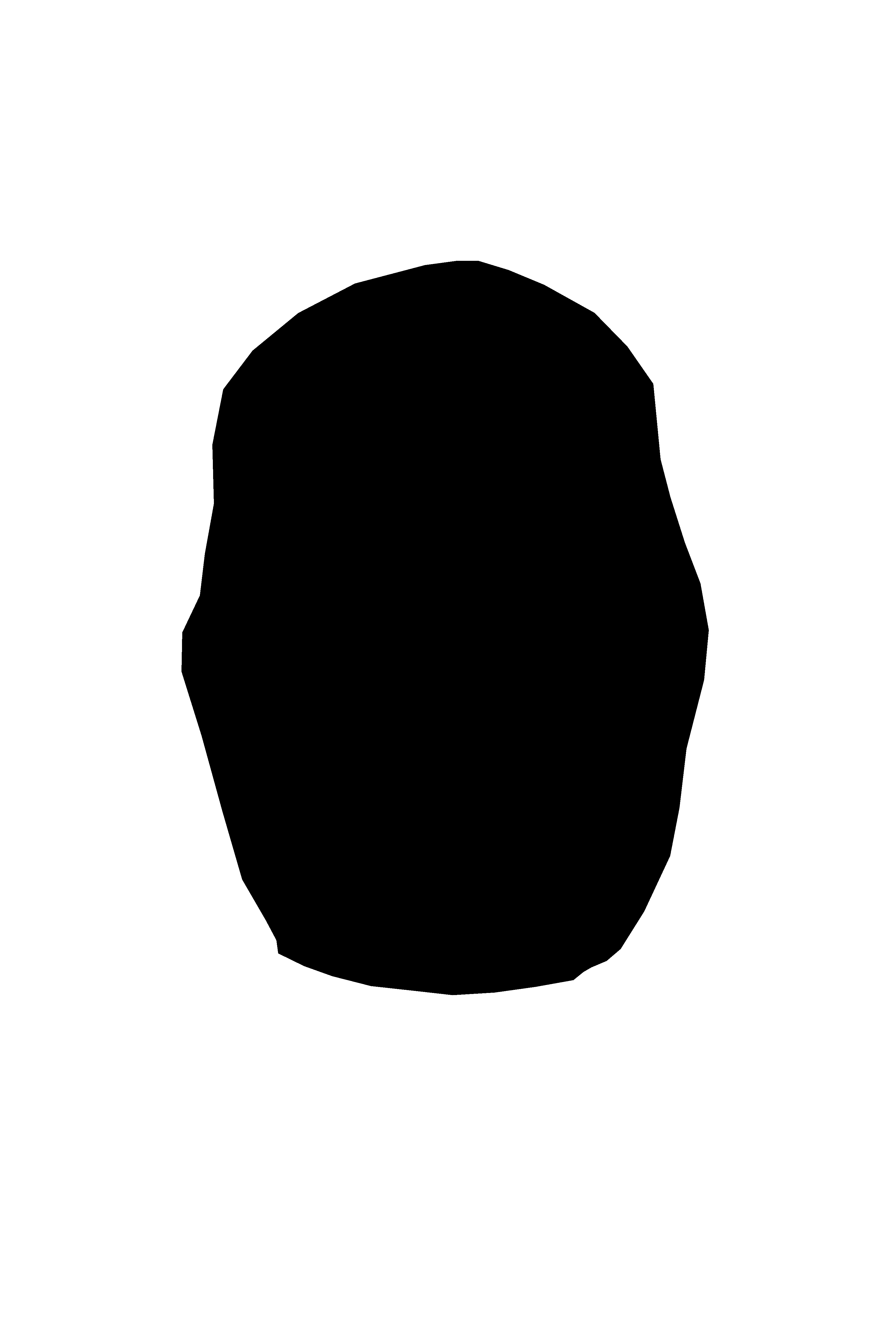

Supplement: Supplementary file 1 [file Data_Sheet_1.zip › face/005_face_mask.png]

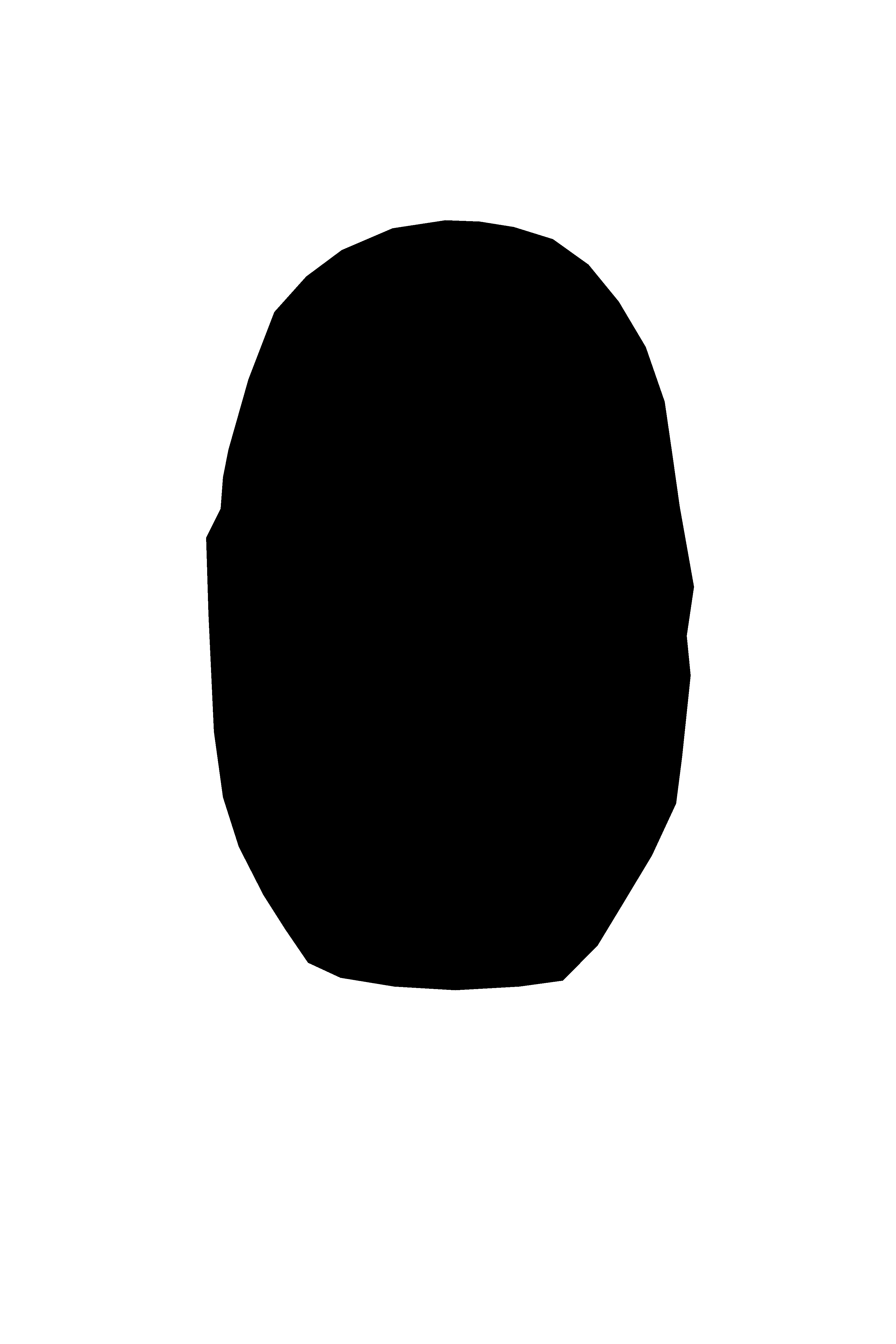

Supplement: Supplementary file 1 [file Data_Sheet_1.zip › face/006_face_mask.png]

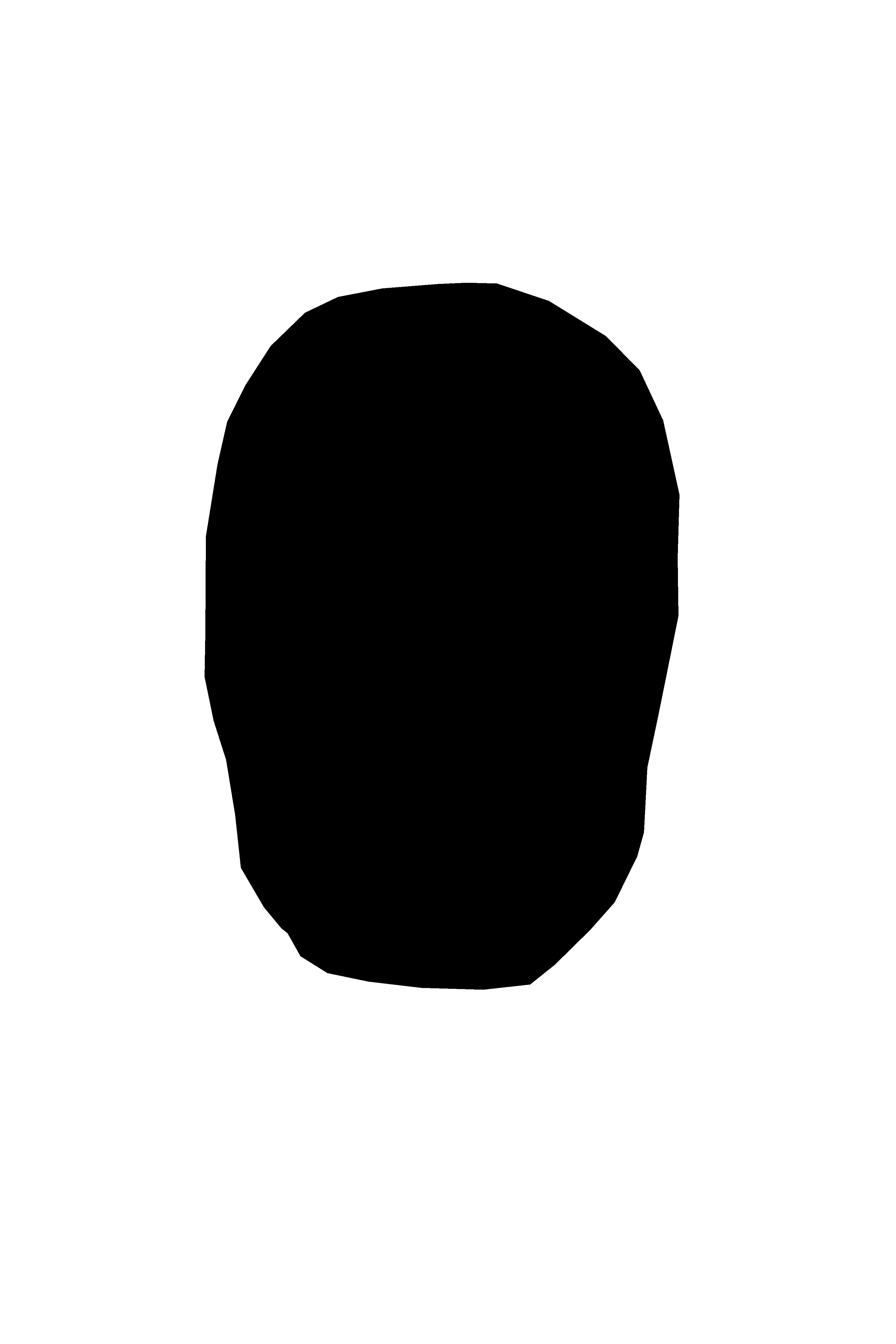

Supplement: Supplementary file 1 [file Data_Sheet_1.zip › face/007_face_mask.png]

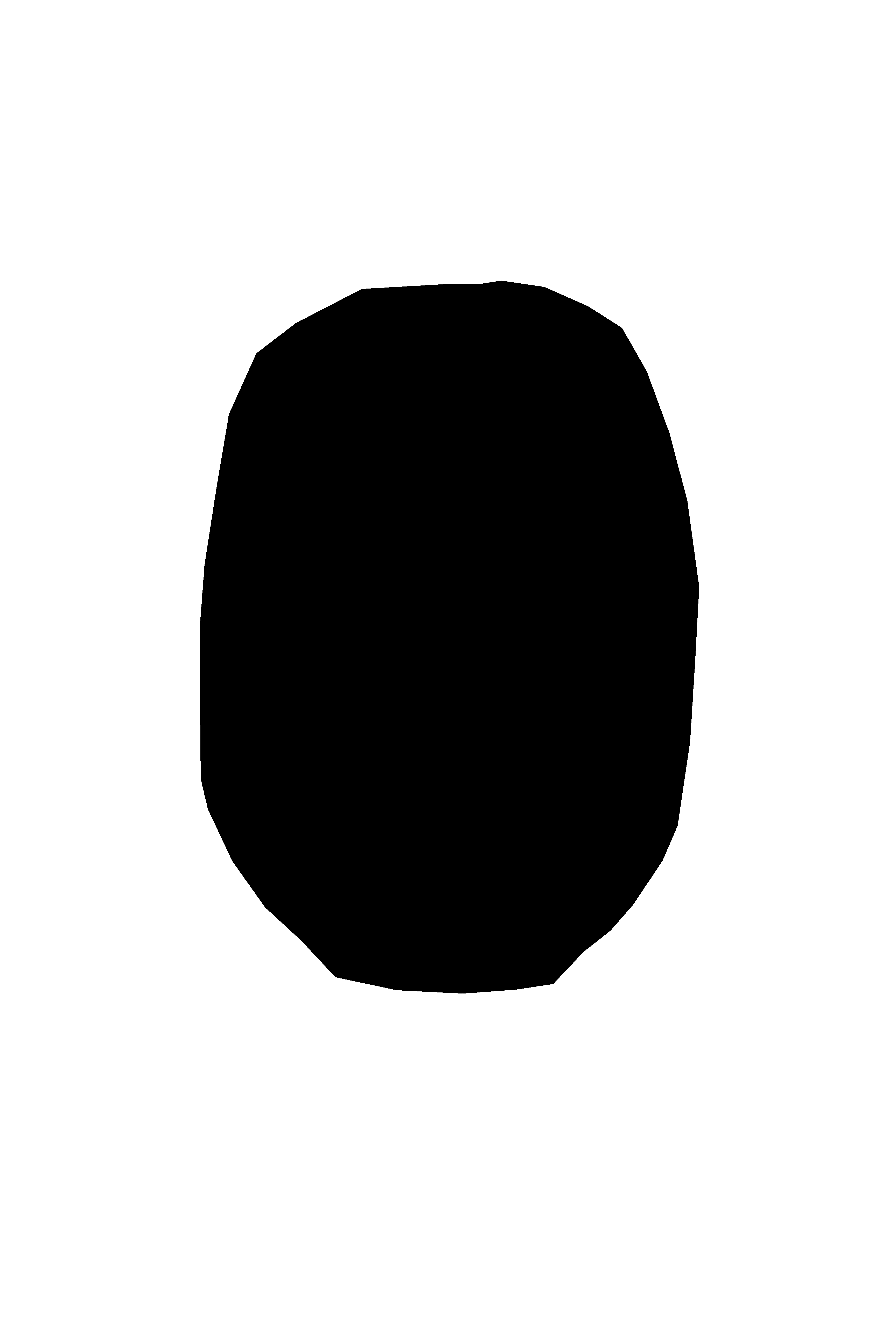

Supplement: Supplementary file 1 [file Data_Sheet_1.zip › face/008_face_mask.png]

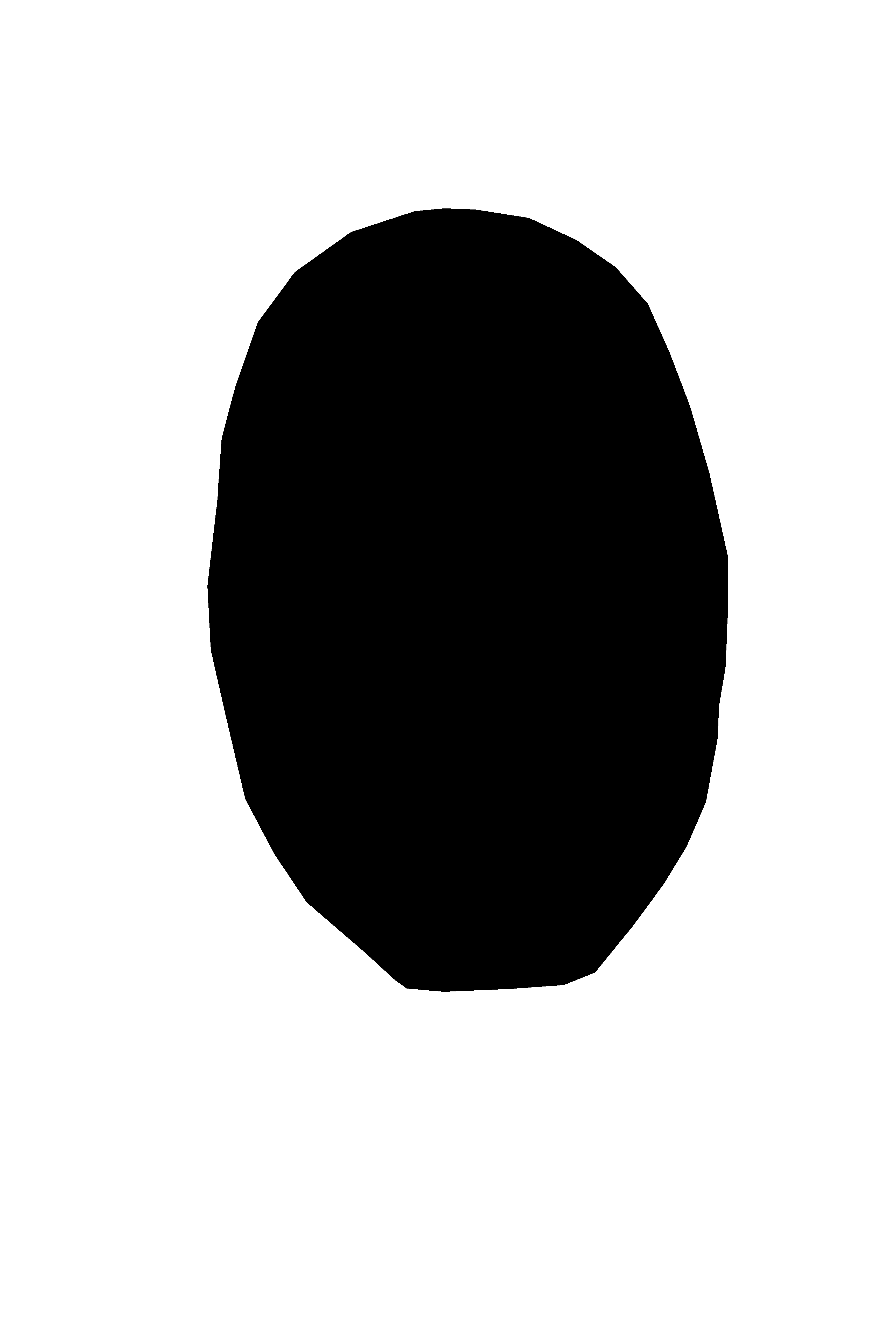

Supplement: Supplementary file 1 [file Data_Sheet_1.zip › face/009_face_mask.png]

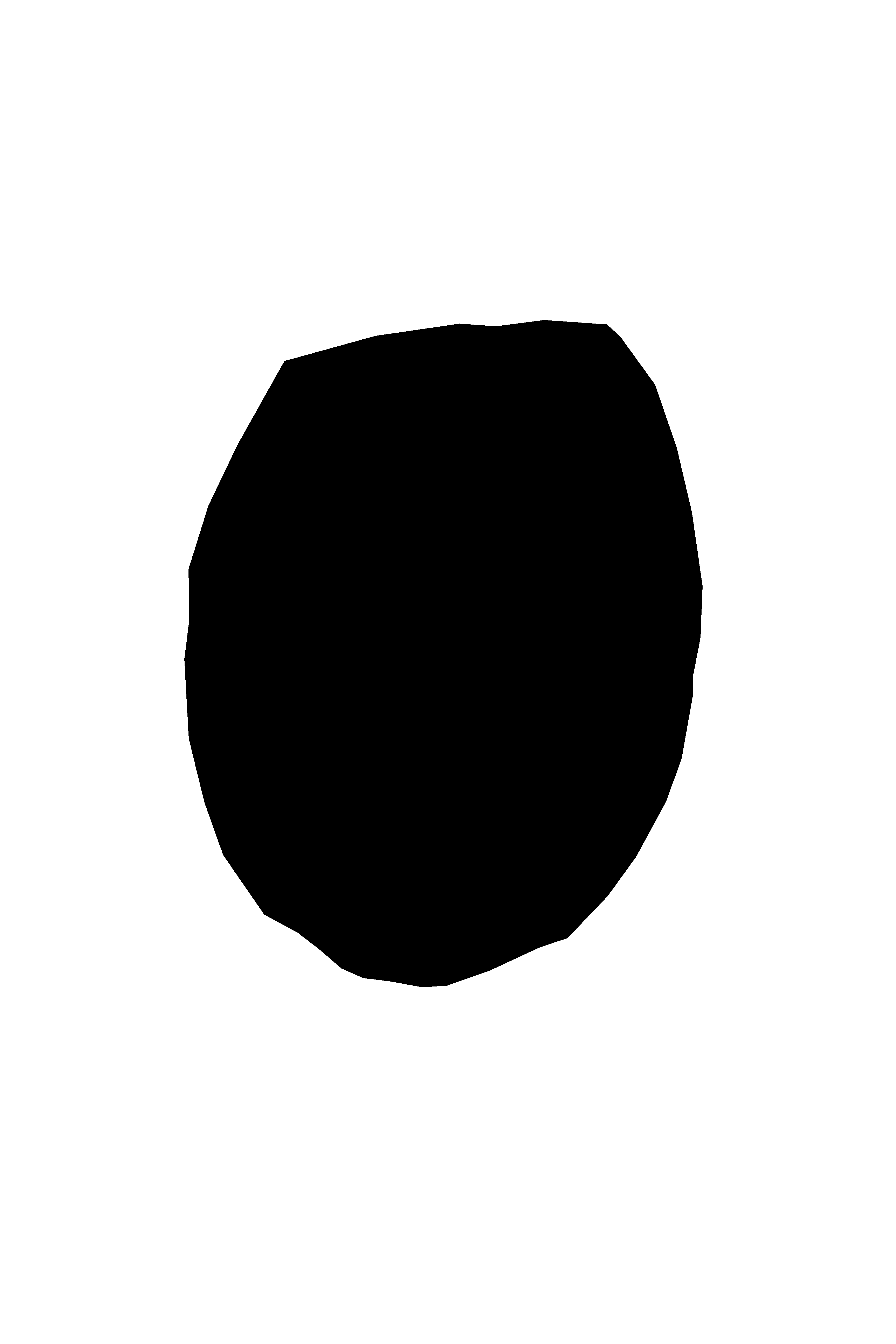

Supplement: Supplementary file 1 [file Data_Sheet_1.zip › face/010_face_mask.png]

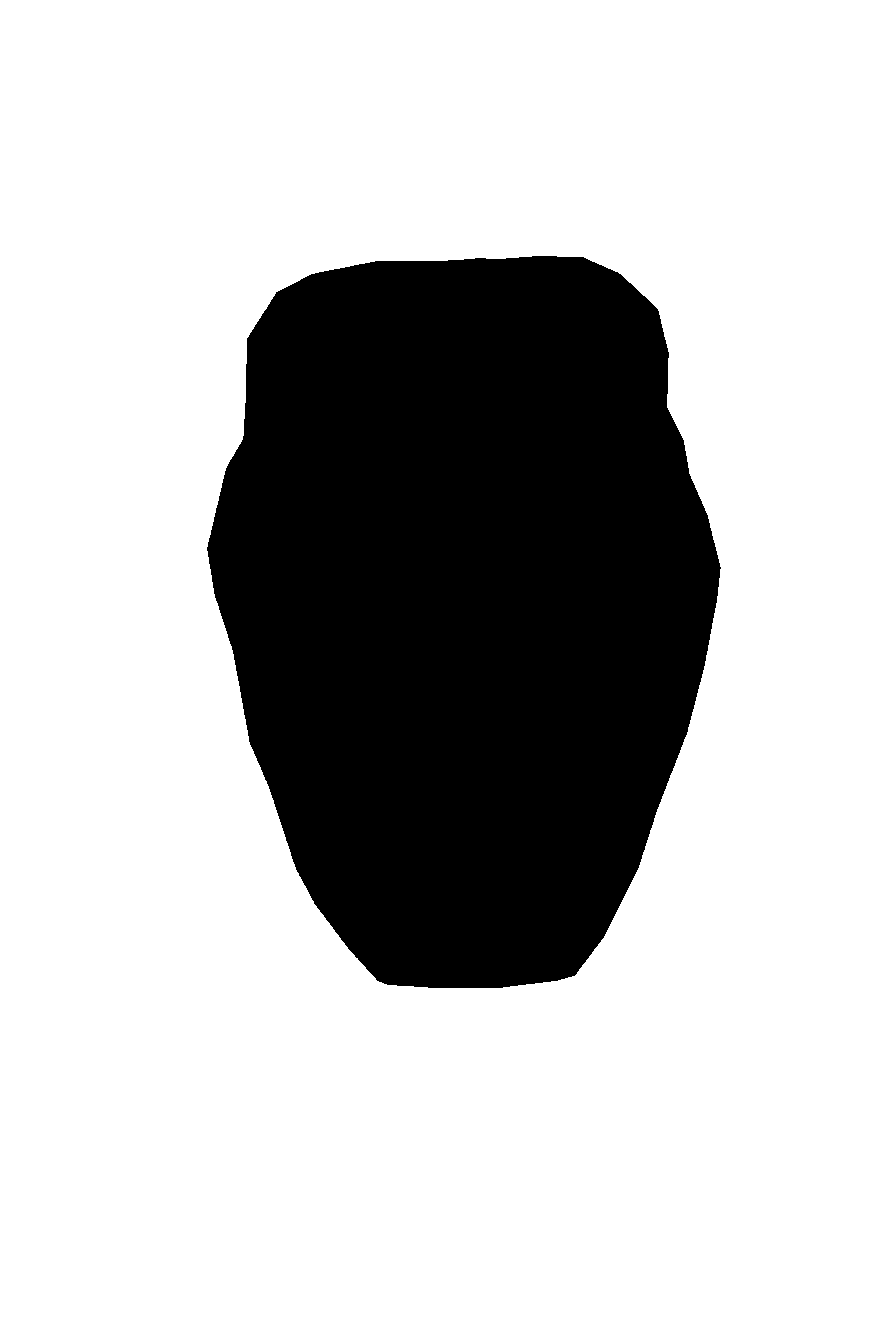

Supplement: Supplementary file 1 [file Data_Sheet_1.zip › face/011_face_mask.png]

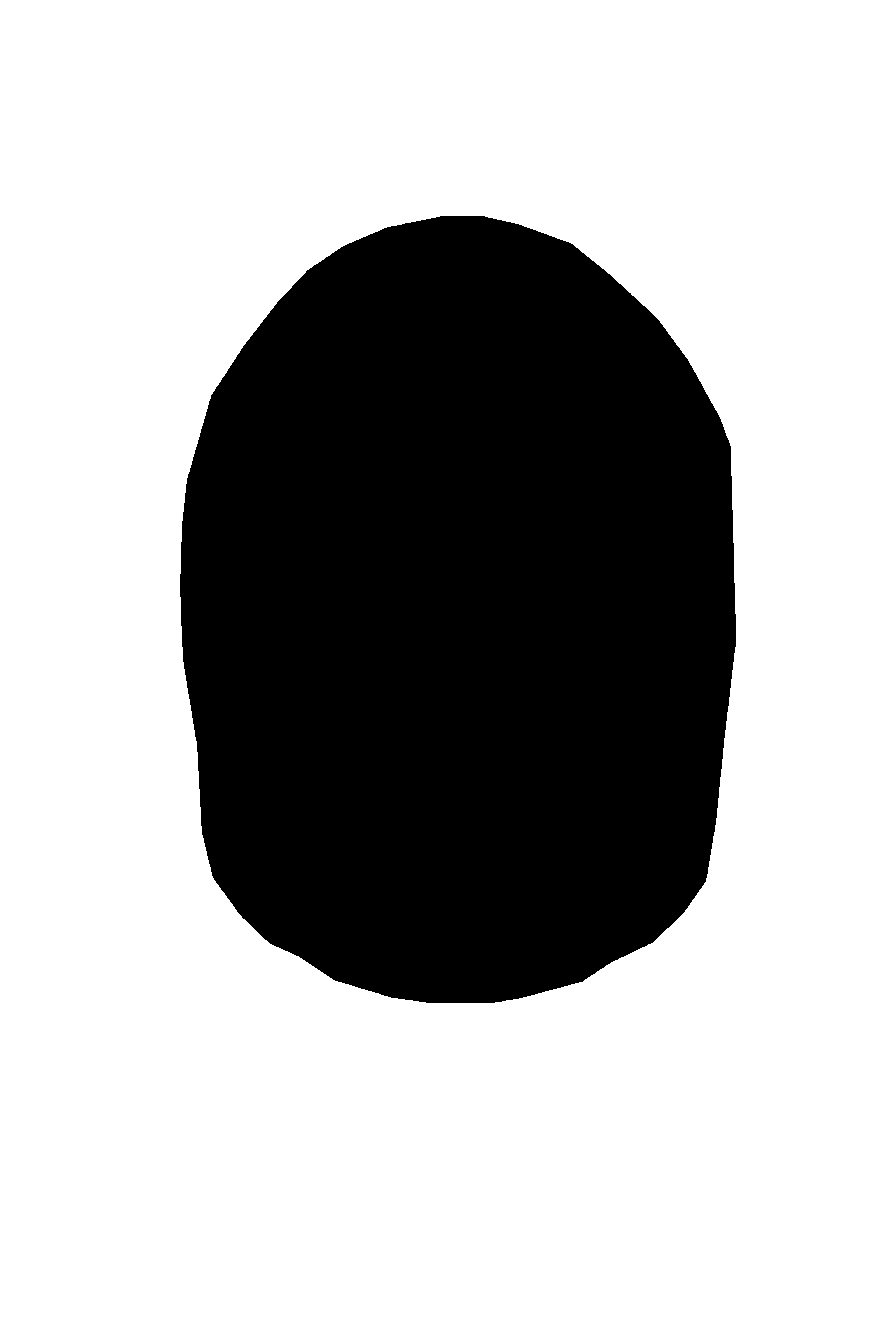

Supplement: Supplementary file 1 [file Data_Sheet_1.zip › face/012_face_mask.png]

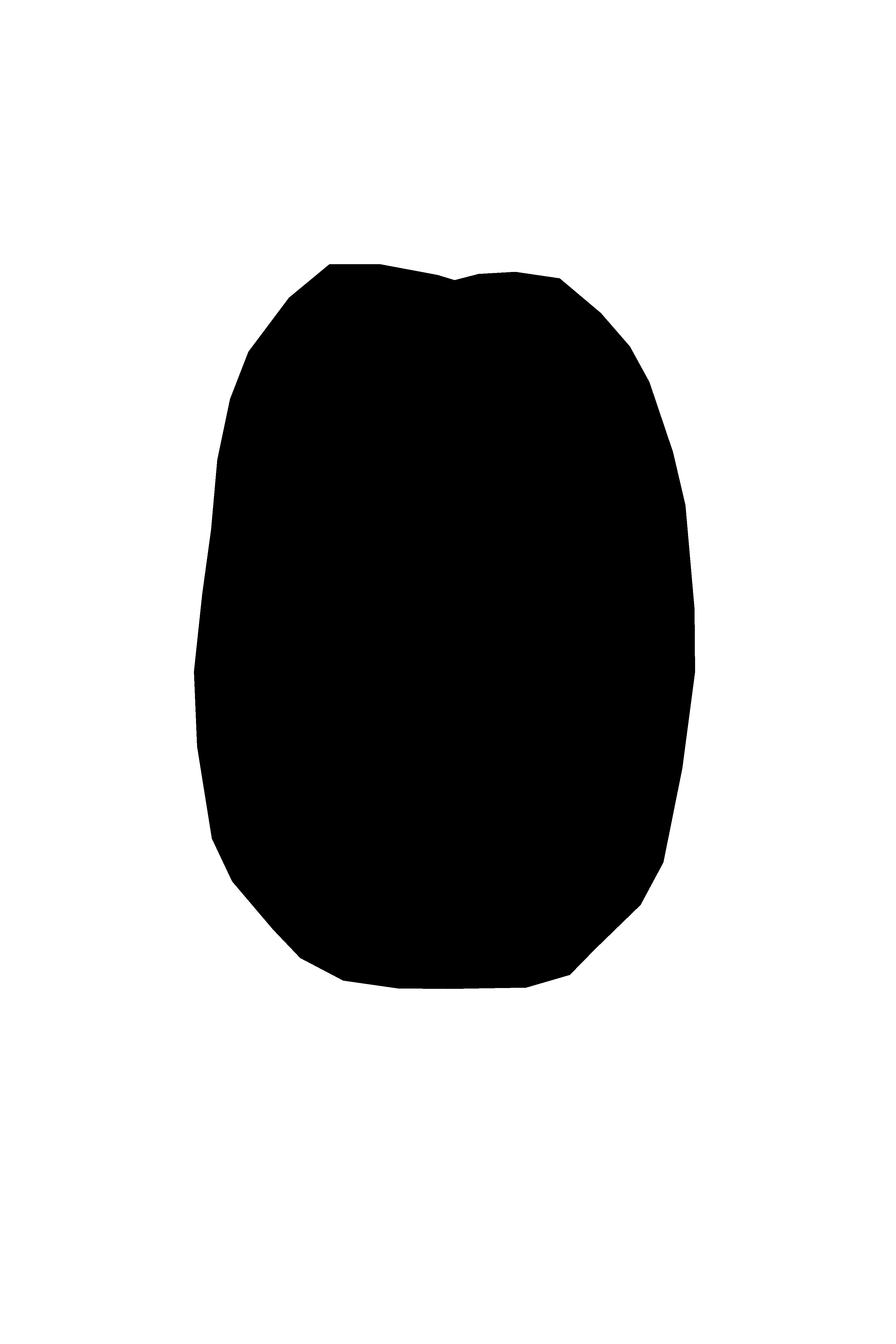

Supplement: Supplementary file 1 [file Data_Sheet_1.zip › face/013_face_mask.png]

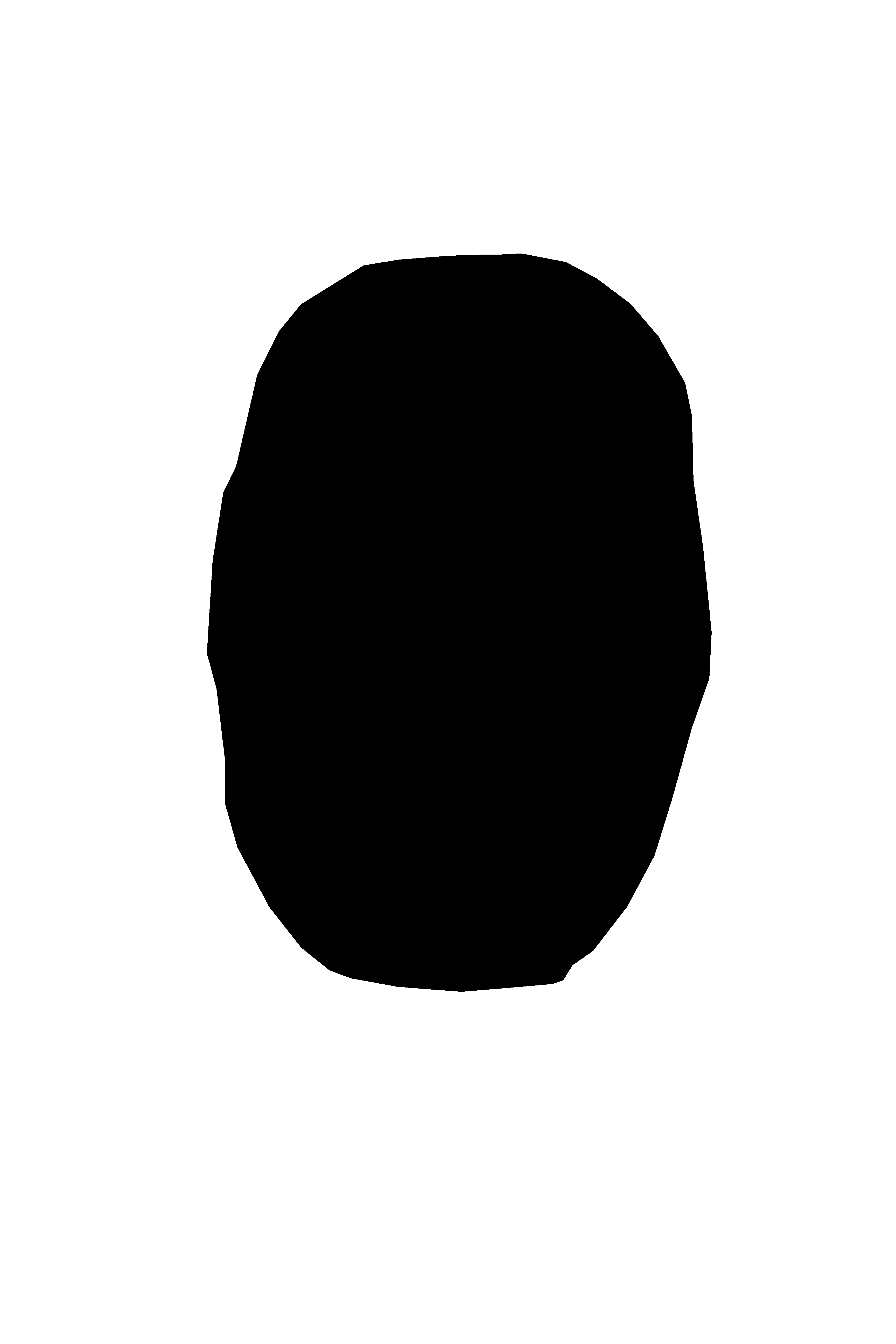

Supplement: Supplementary file 1 [file Data_Sheet_1.zip › face/014_face_mask.png]

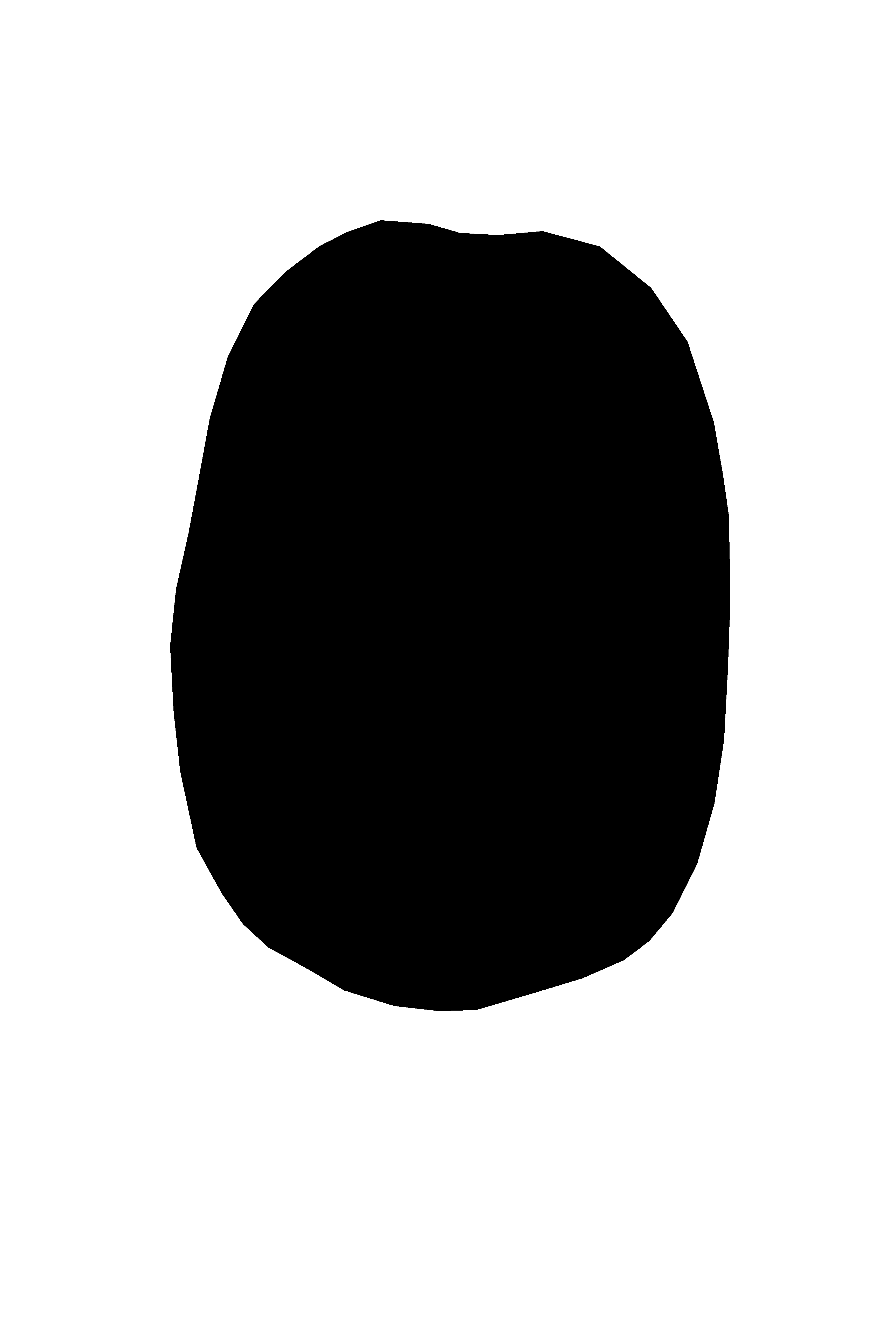

Supplement: Supplementary file 1 [file Data_Sheet_1.zip › face/015_face_mask.png]

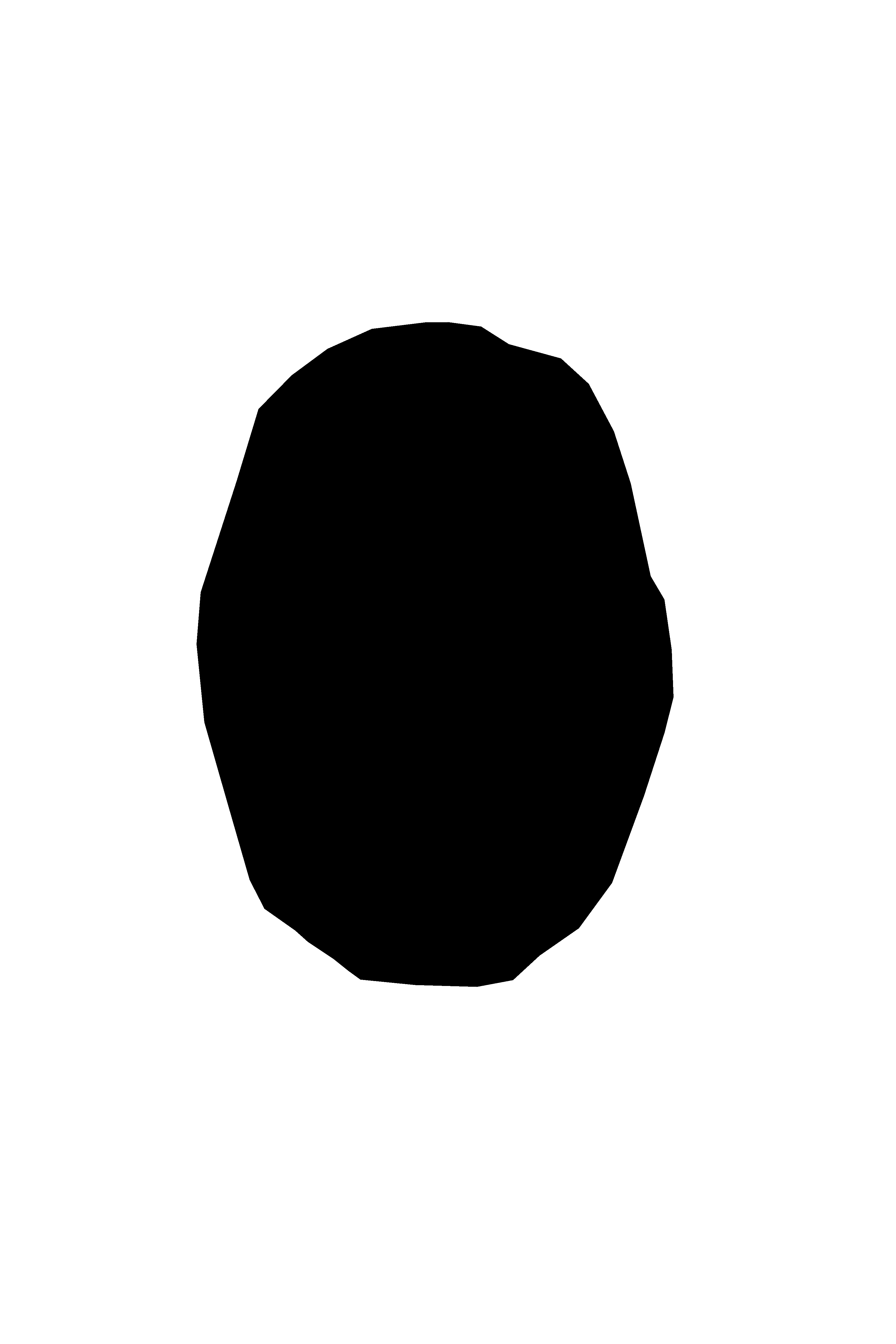

Supplement: Supplementary file 1 [file Data_Sheet_1.zip › face/016_face_mask.png]

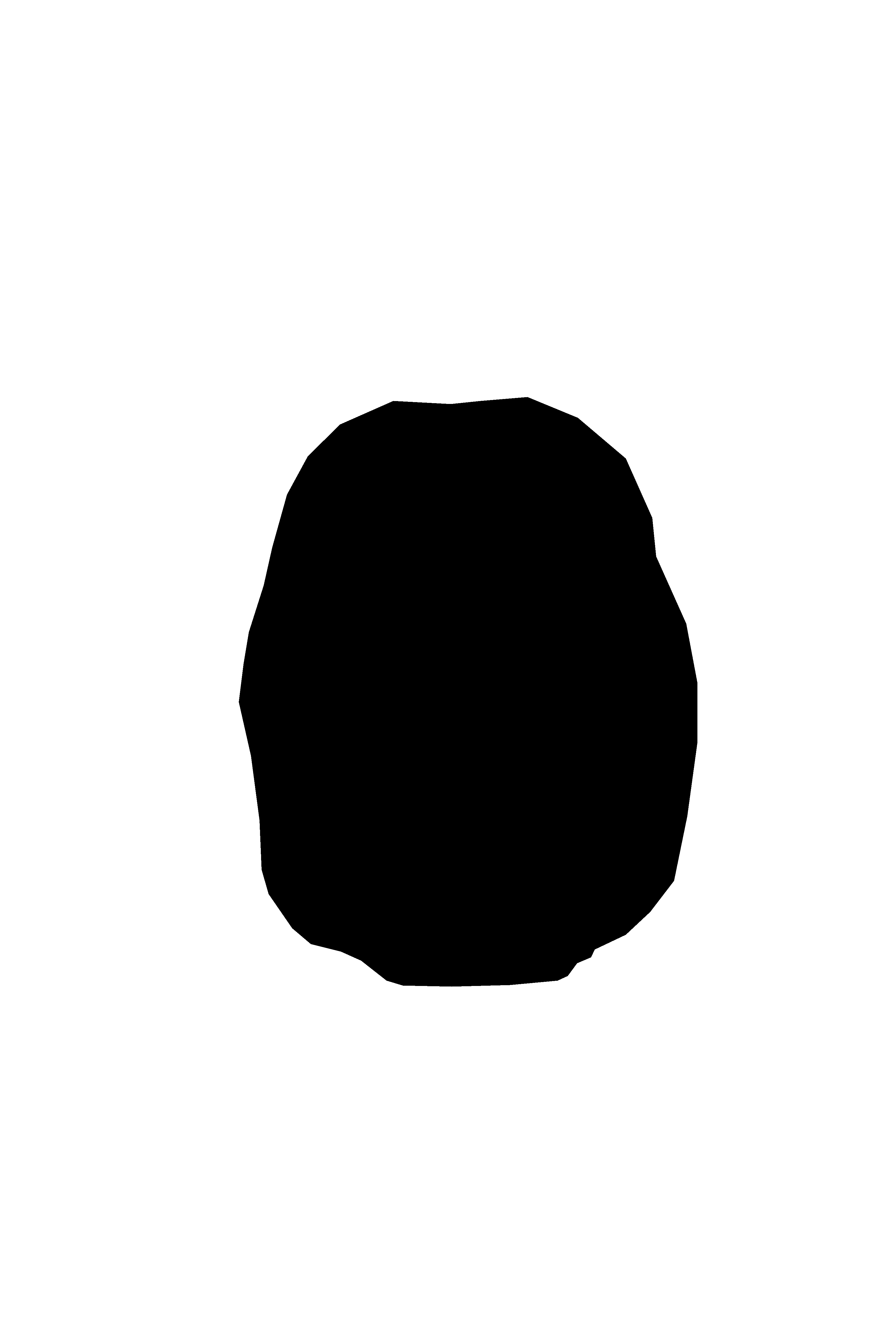

Supplement: Supplementary file 1 [file Data_Sheet_1.zip › face/017_face_mask.png]

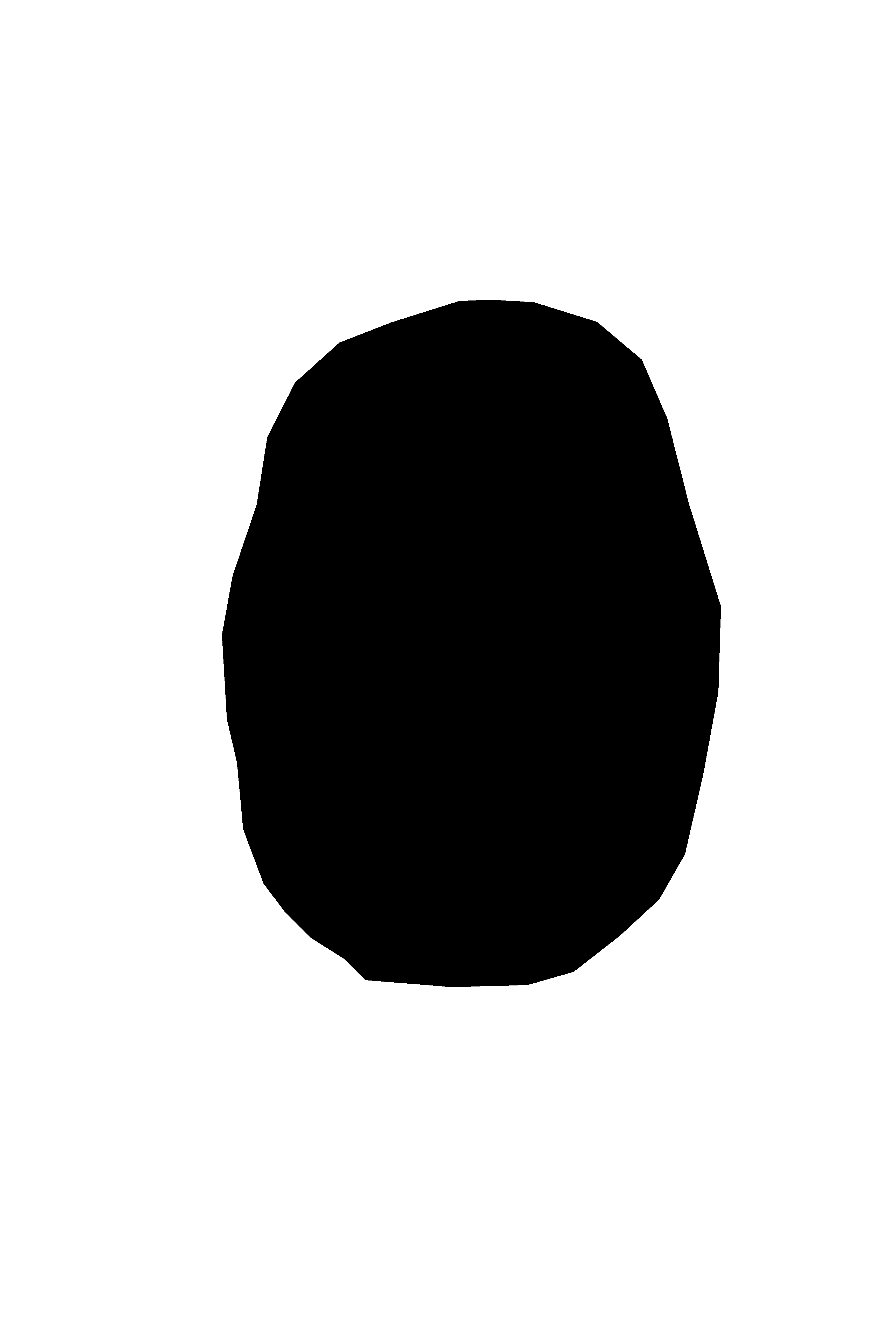

Supplement: Supplementary file 1 [file Data_Sheet_1.zip › face/018_face_mask.png]

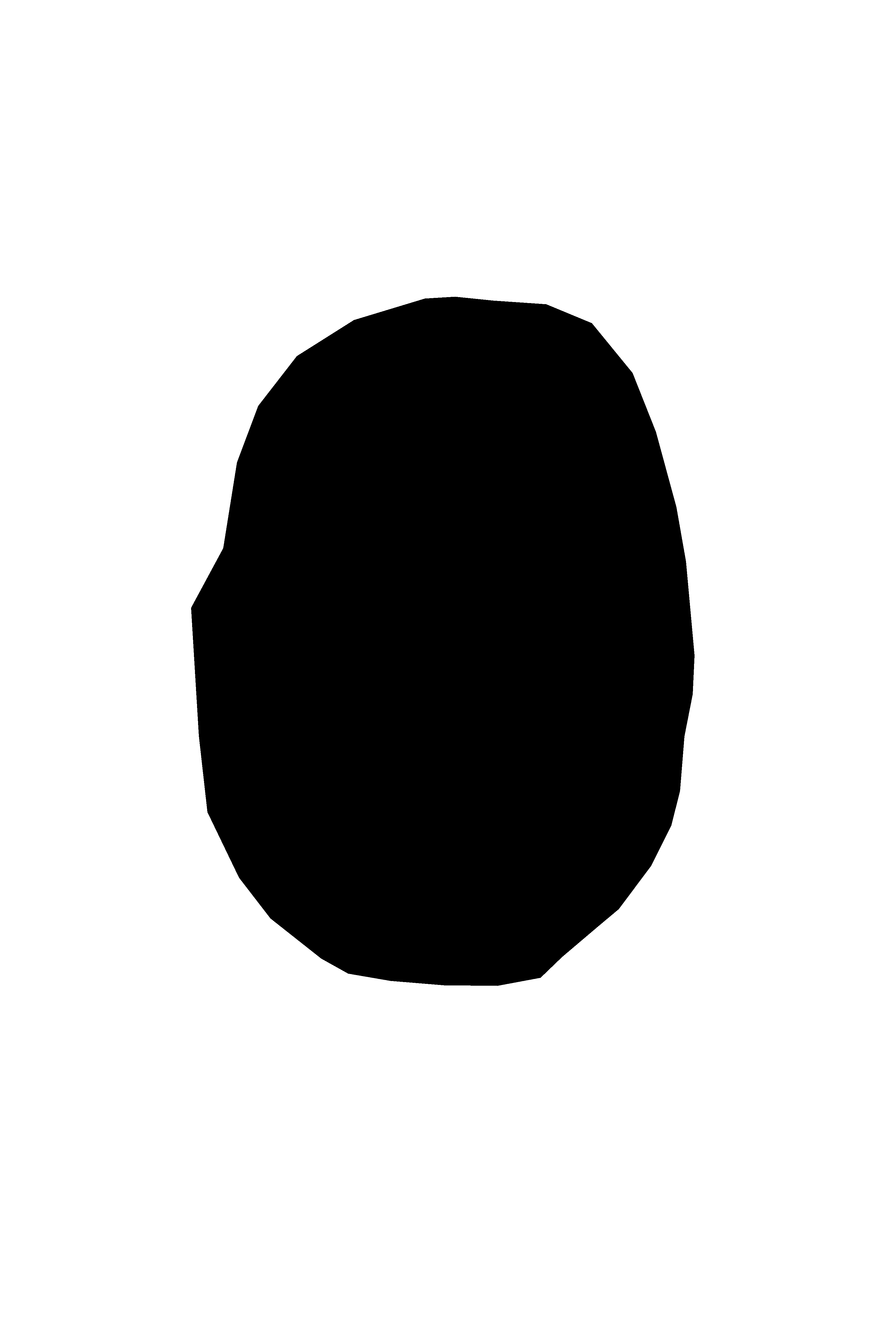

Supplement: Supplementary file 1 [file Data_Sheet_1.zip › face/019_face_mask.png]

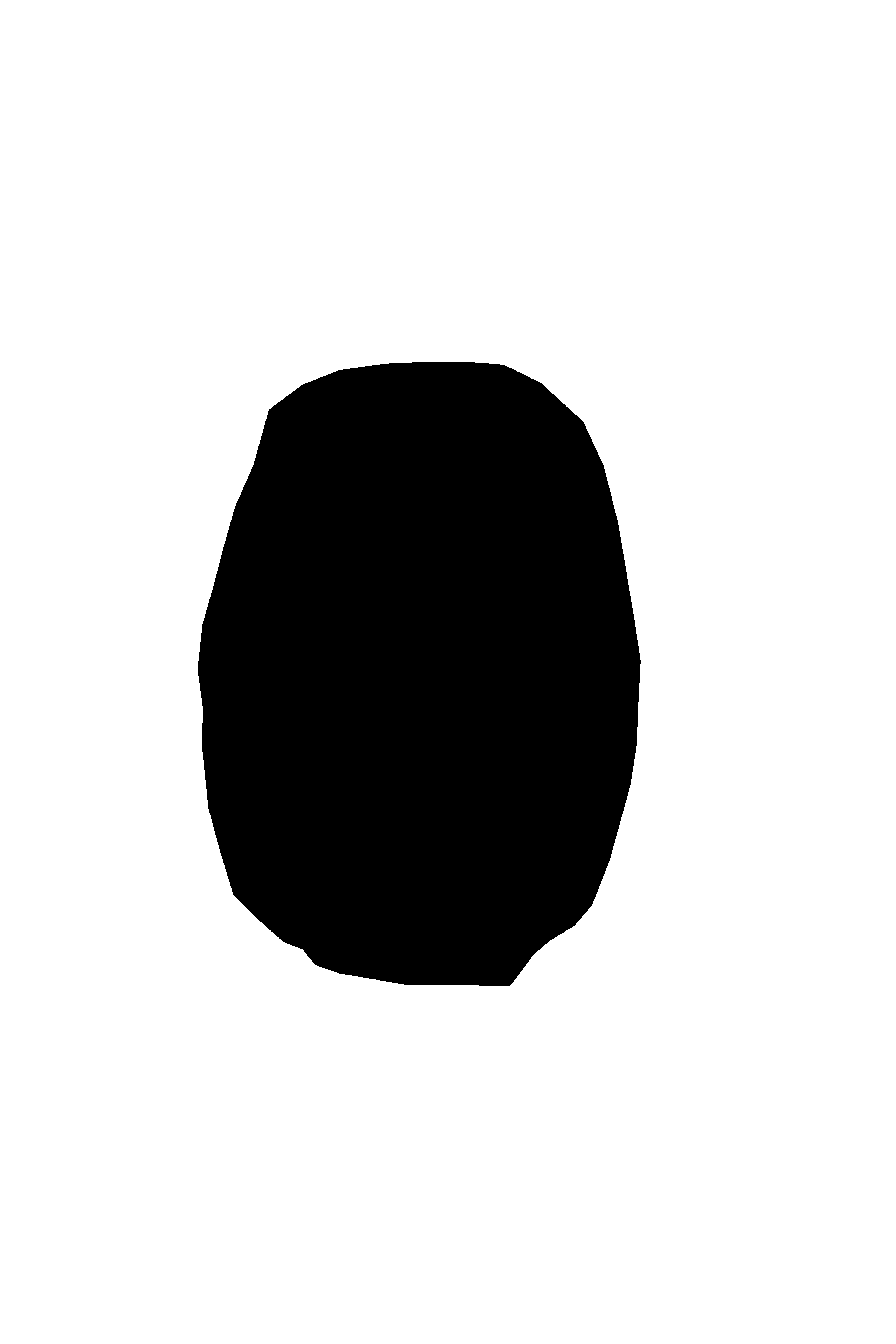

Supplement: Supplementary file 1 [file Data_Sheet_1.zip › face/020_face_mask.png]

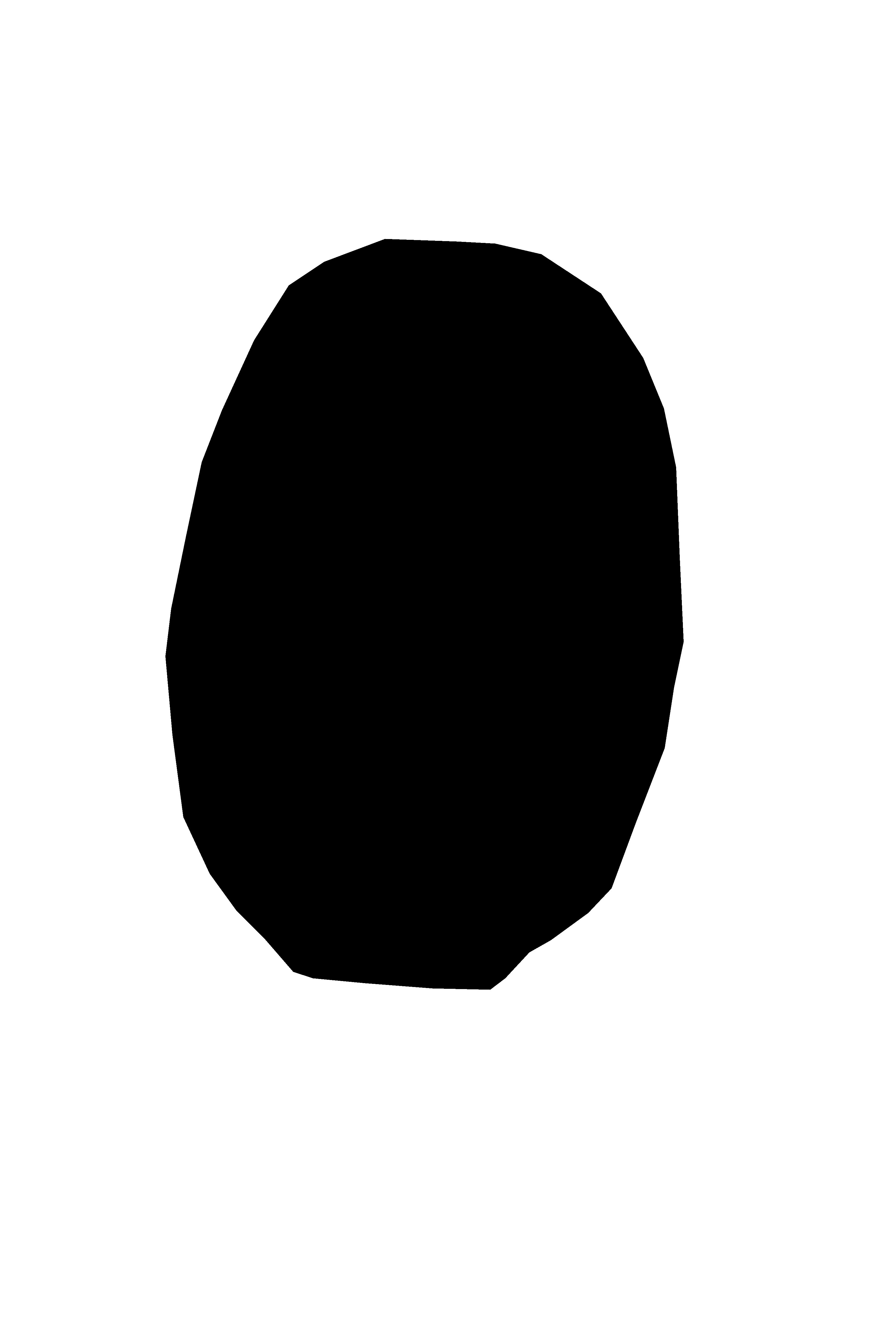

Supplement: Supplementary file 1 [file Data_Sheet_1.zip › face/021_face_mask.png]

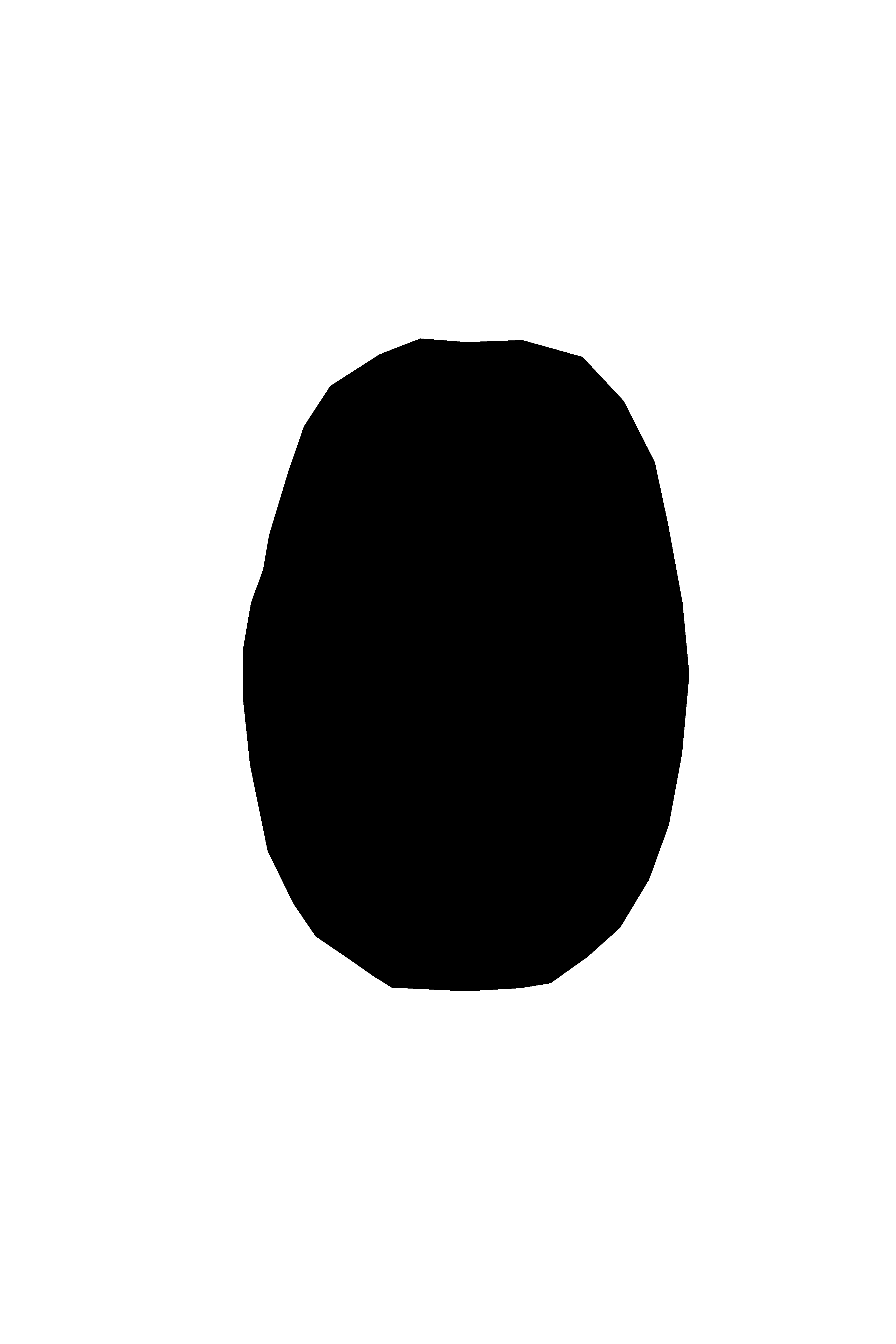

Supplement: Supplementary file 1 [file Data_Sheet_1.zip › face/022_face_mask.png]

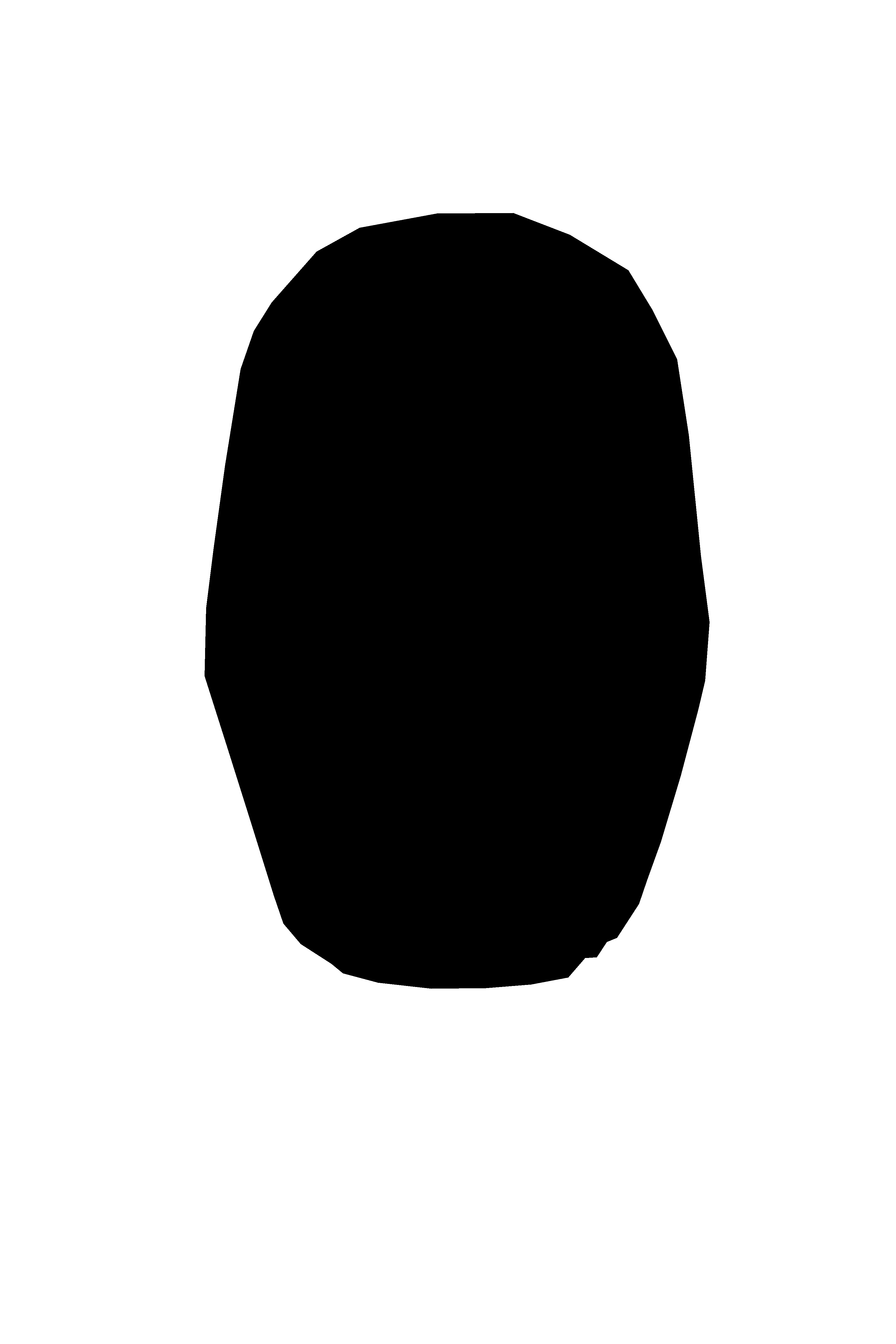

Supplement: Supplementary file 1 [file Data_Sheet_1.zip › face/023_face_mask.png]

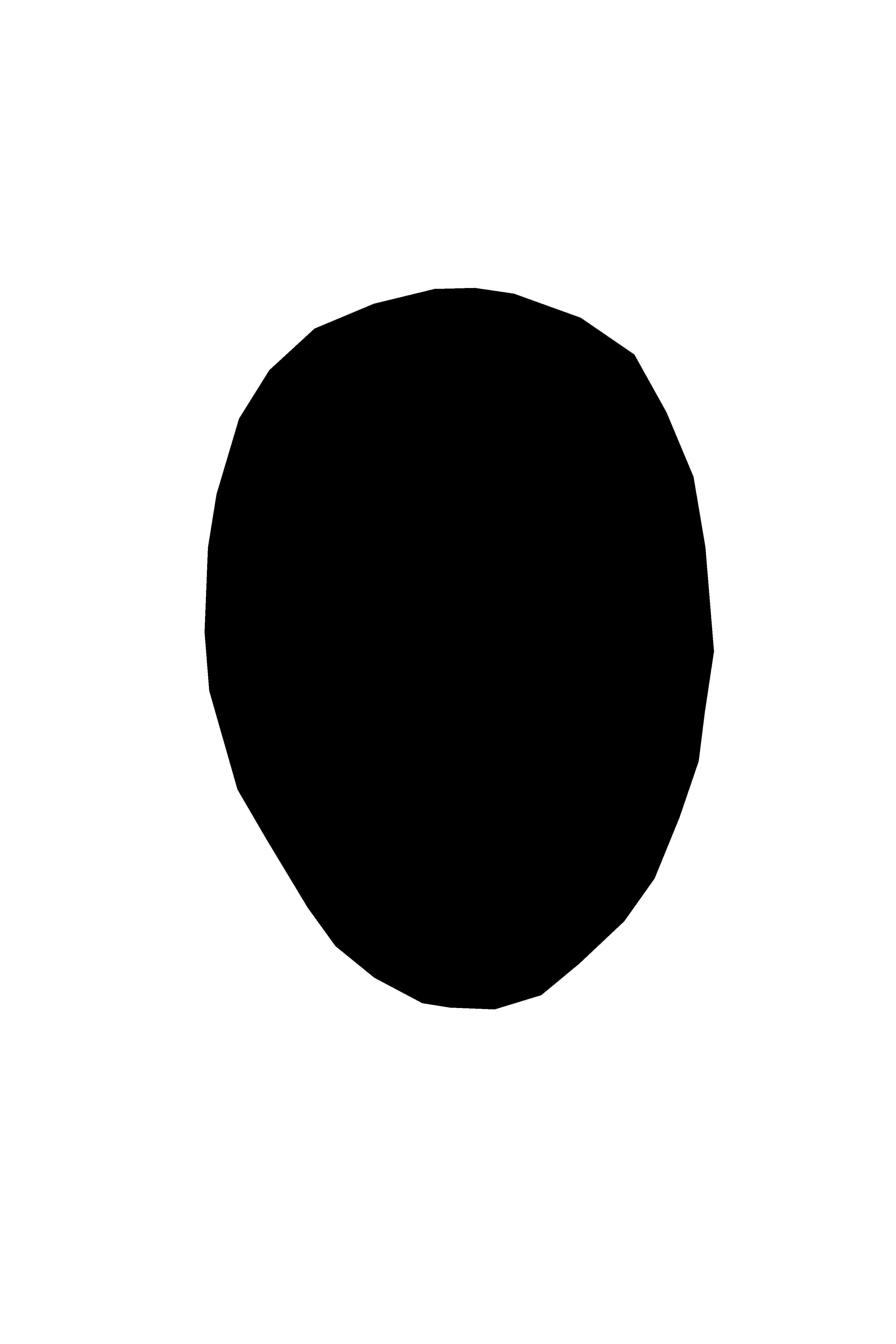

Supplement: Supplementary file 1 [file Data_Sheet_1.zip › face/024_face_mask.png]

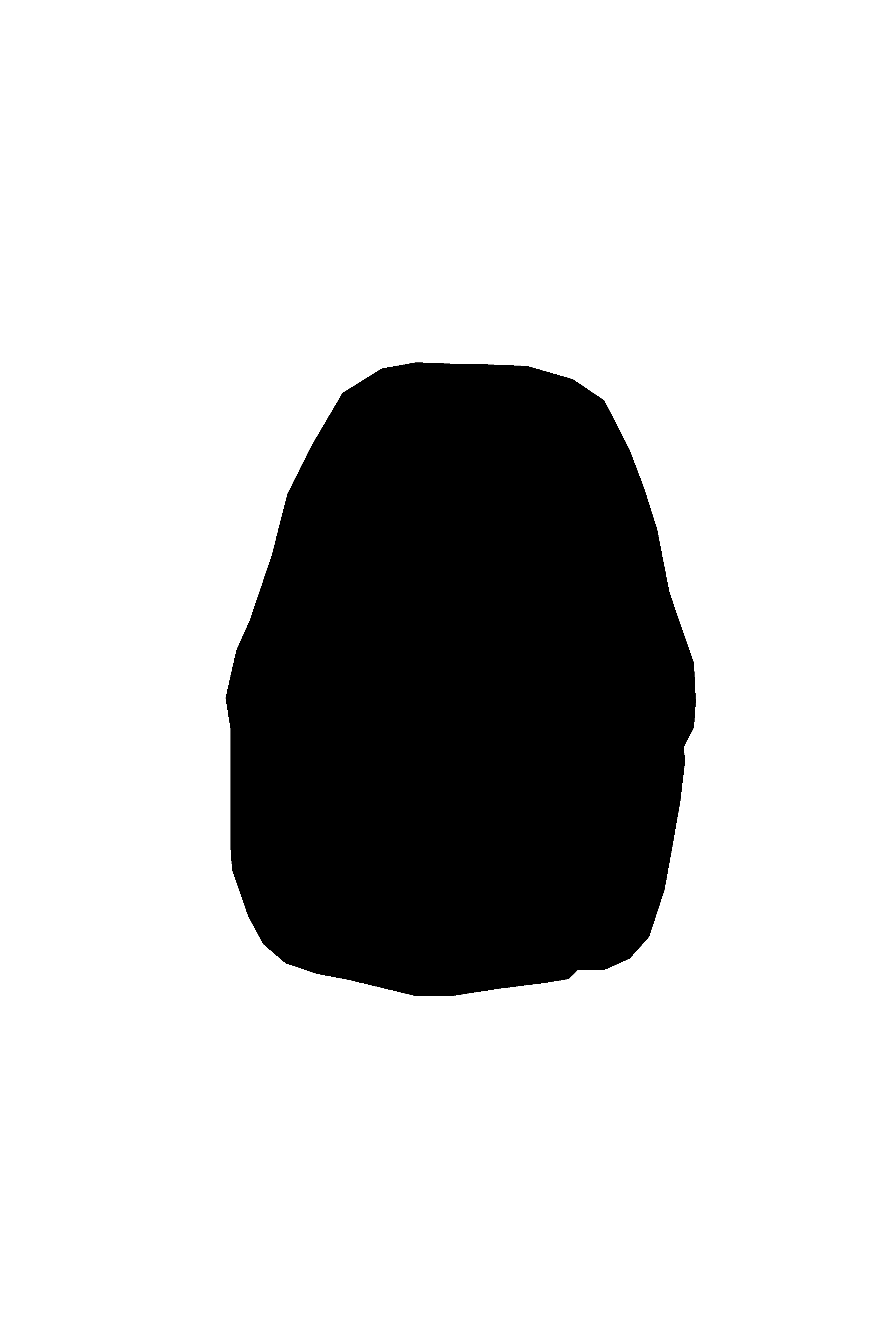

Supplement: Supplementary file 1 [file Data_Sheet_1.zip › face/025_face_mask.png]

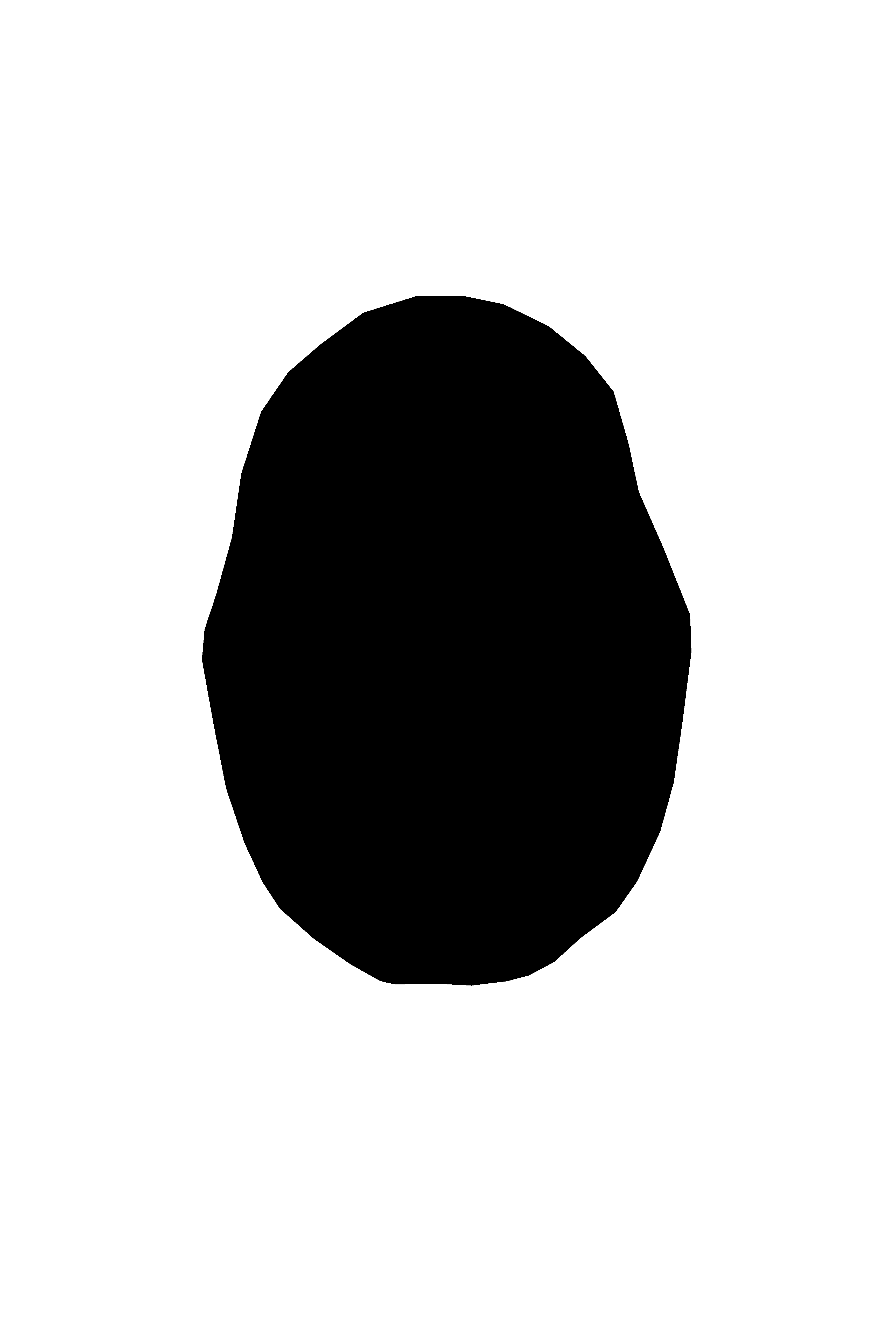

Supplement: Supplementary file 1 [file Data_Sheet_1.zip › face/026_face_mask.png]

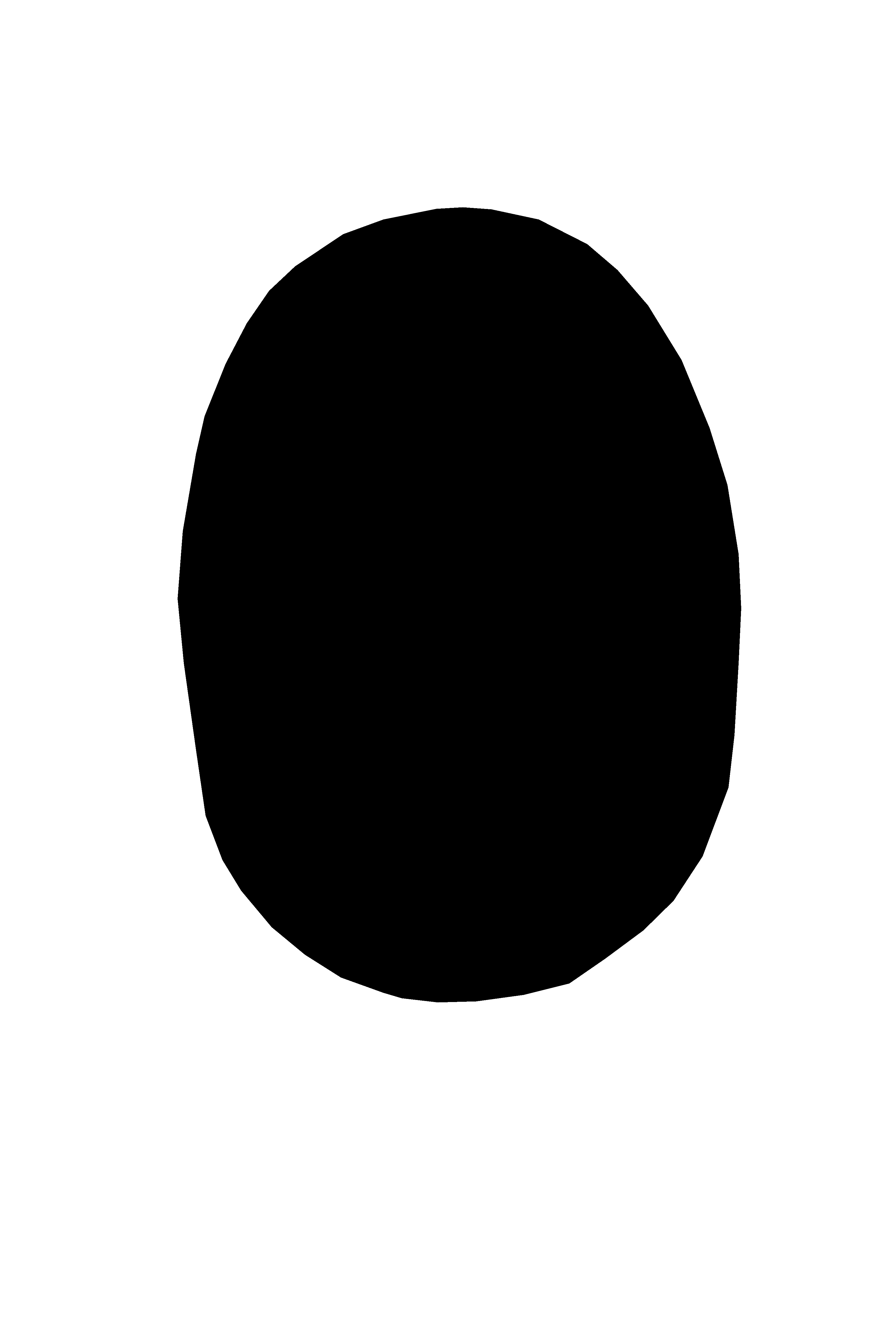

Supplement: Supplementary file 1 [file Data_Sheet_1.zip › face/027_face_mask.png]

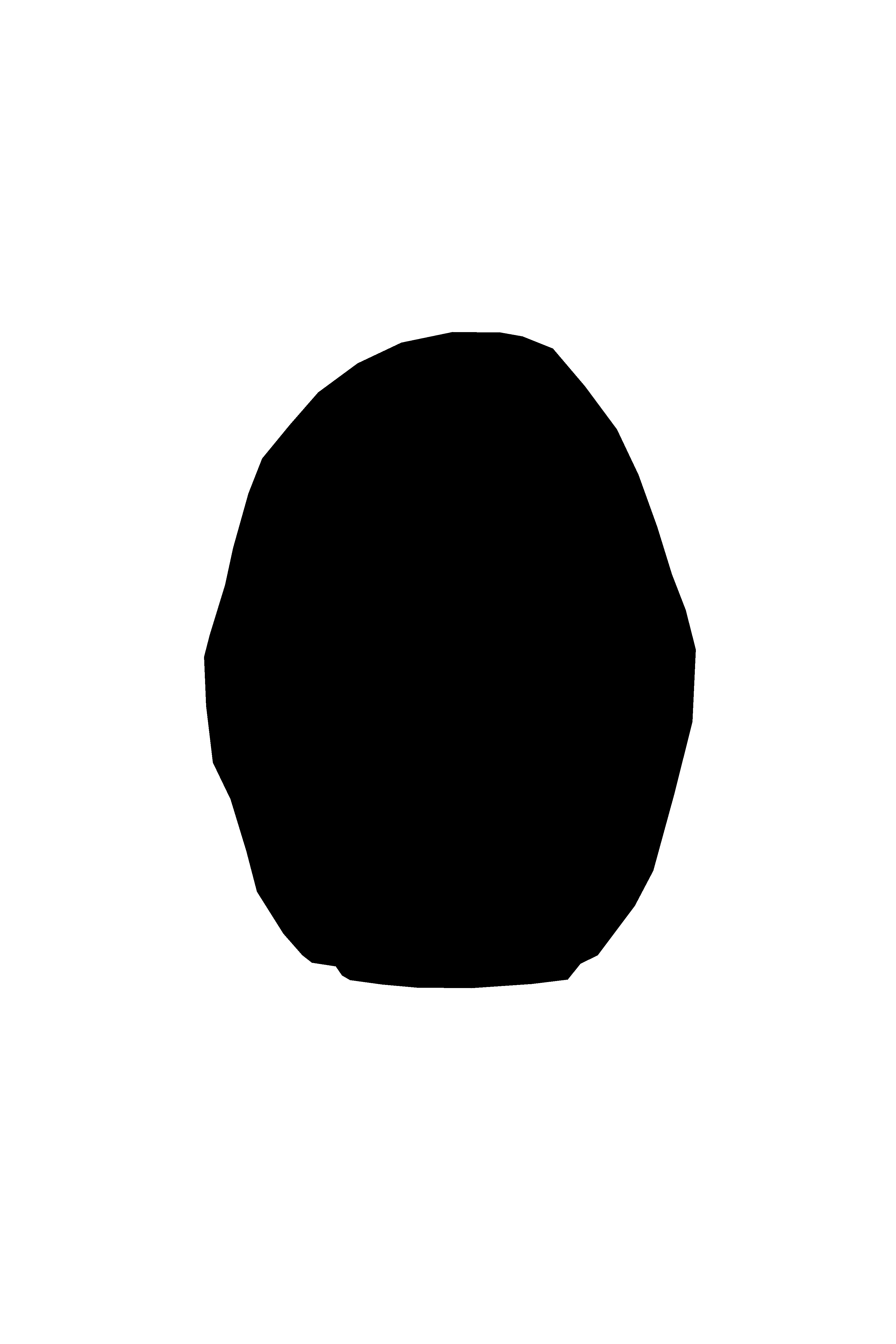

Supplement: Supplementary file 1 [file Data_Sheet_1.zip › face/028_face_mask.png]

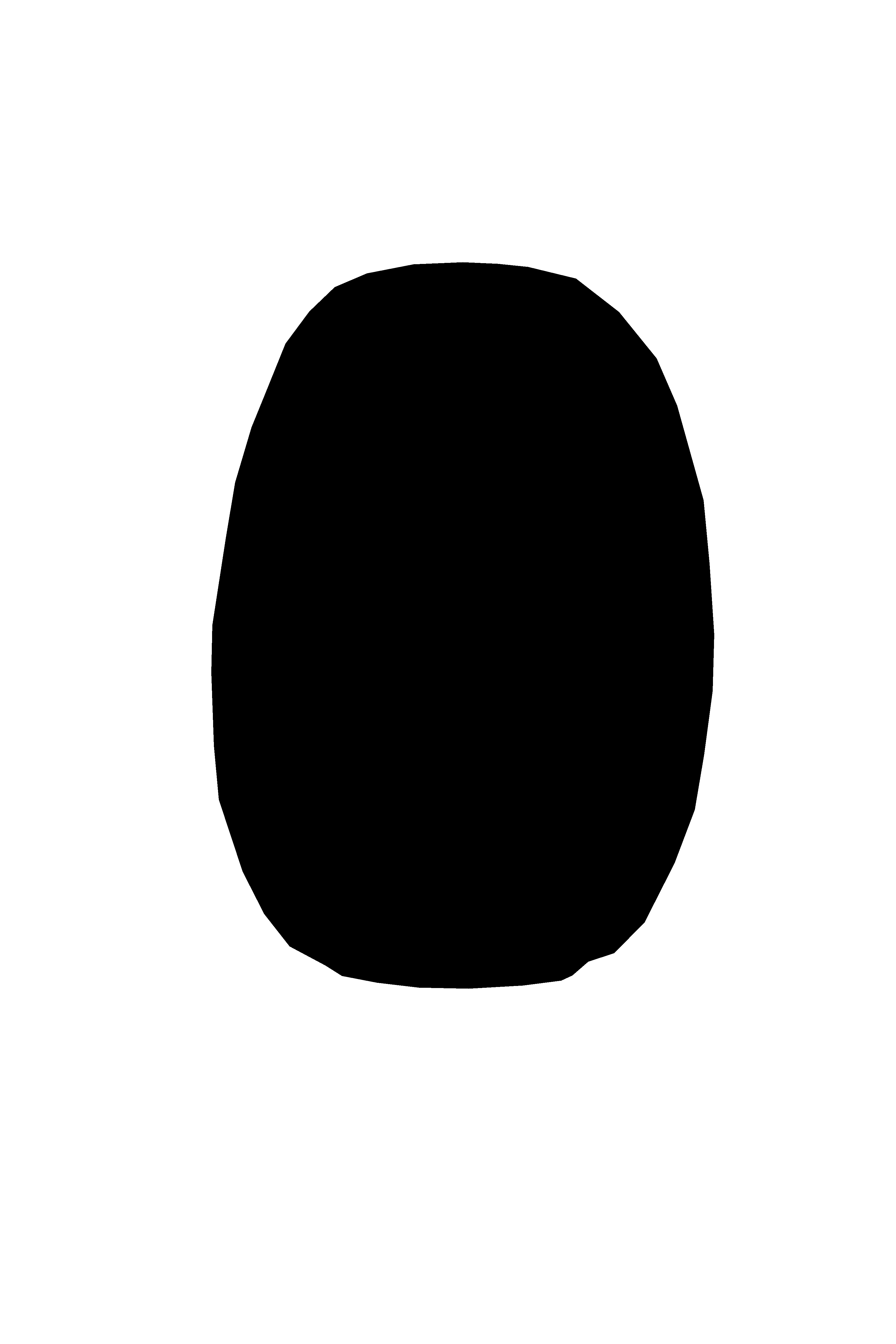

Supplement: Supplementary file 1 [file Data_Sheet_1.zip › face/029_face_mask.png]

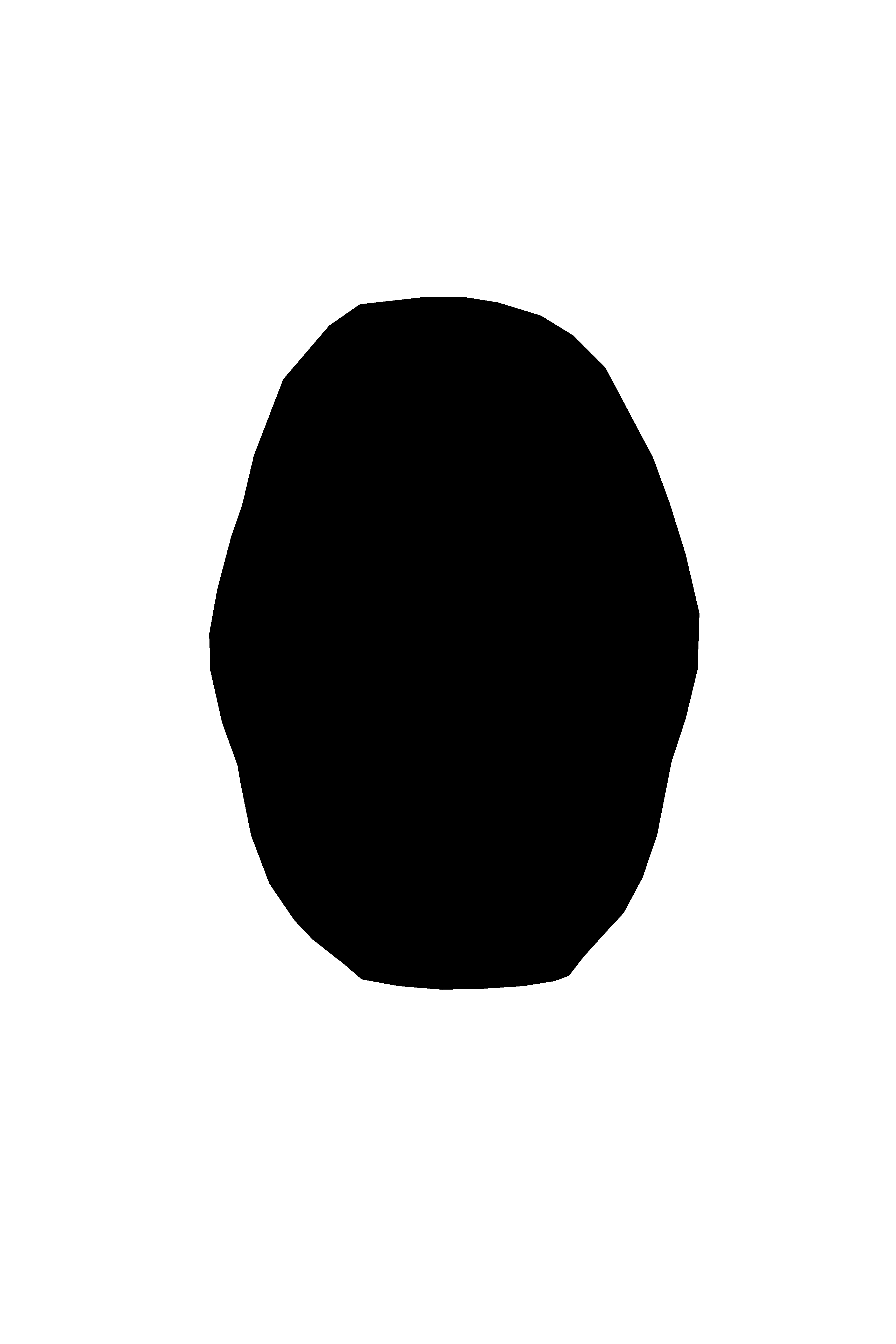

Supplement: Supplementary file 1 [file Data_Sheet_1.zip › face/030_face_mask.png]

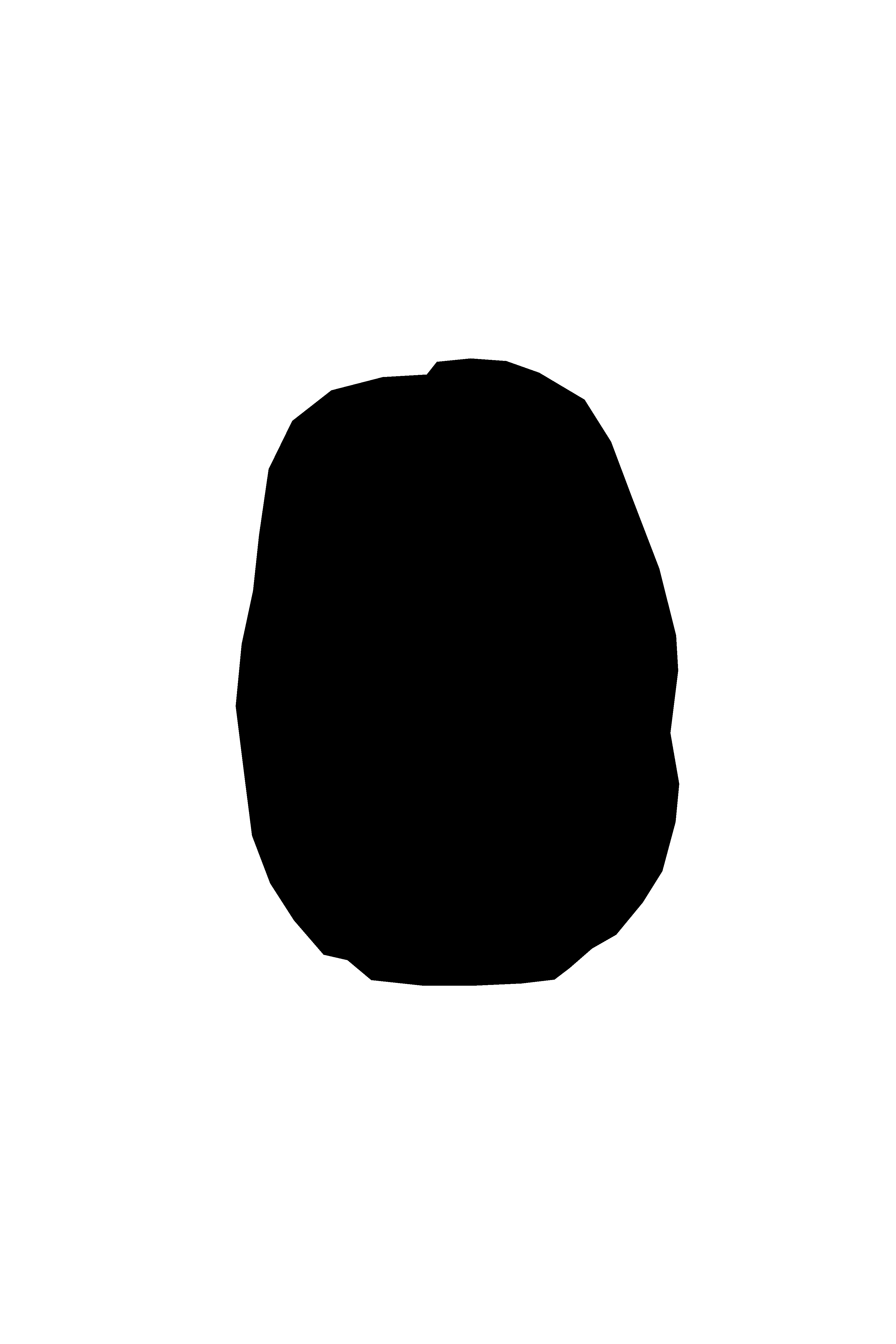

Supplement: Supplementary file 1 [file Data_Sheet_1.zip › face/031_face_mask.png]

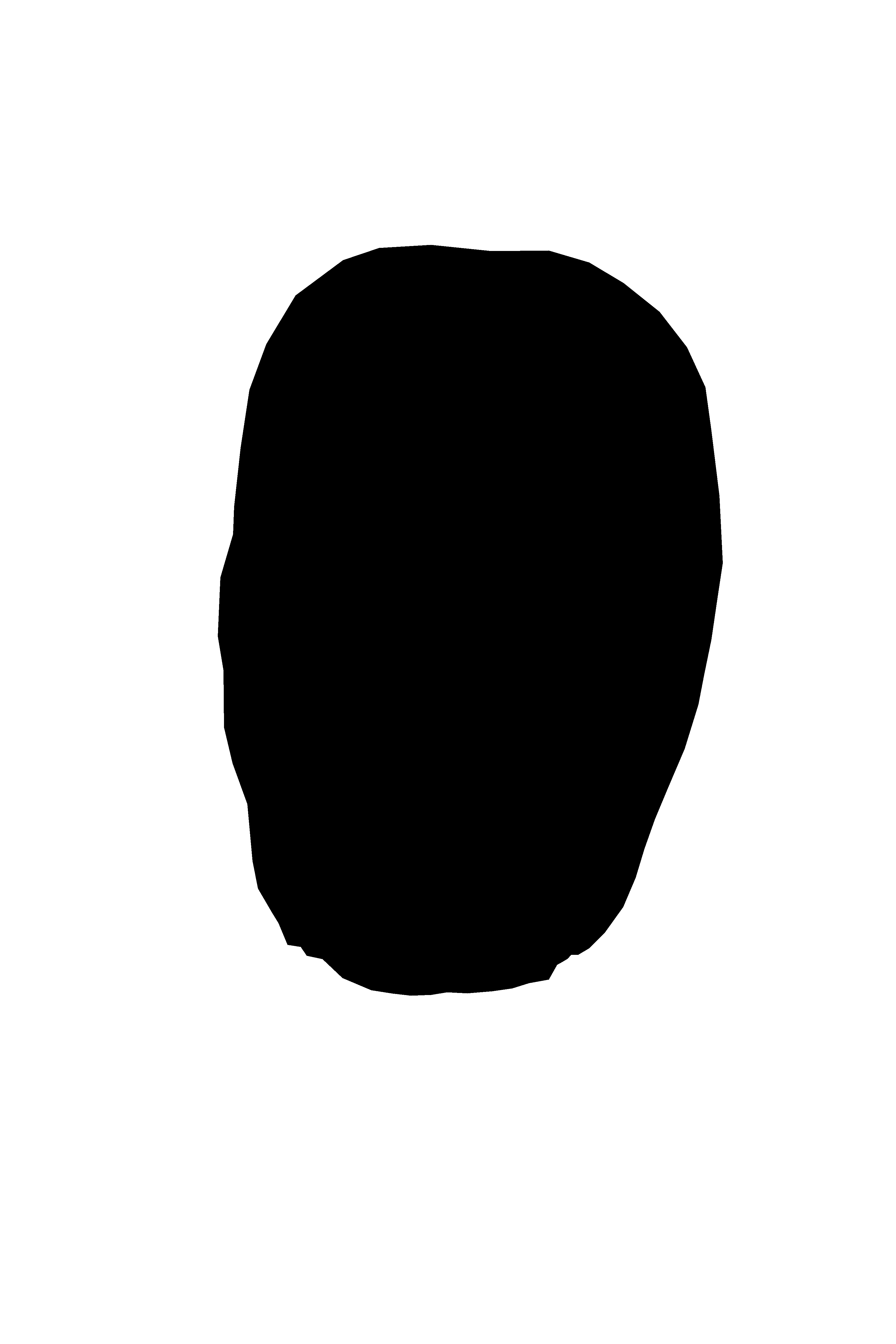

Supplement: Supplementary file 1 [file Data_Sheet_1.zip › face/032_face_mask.png]

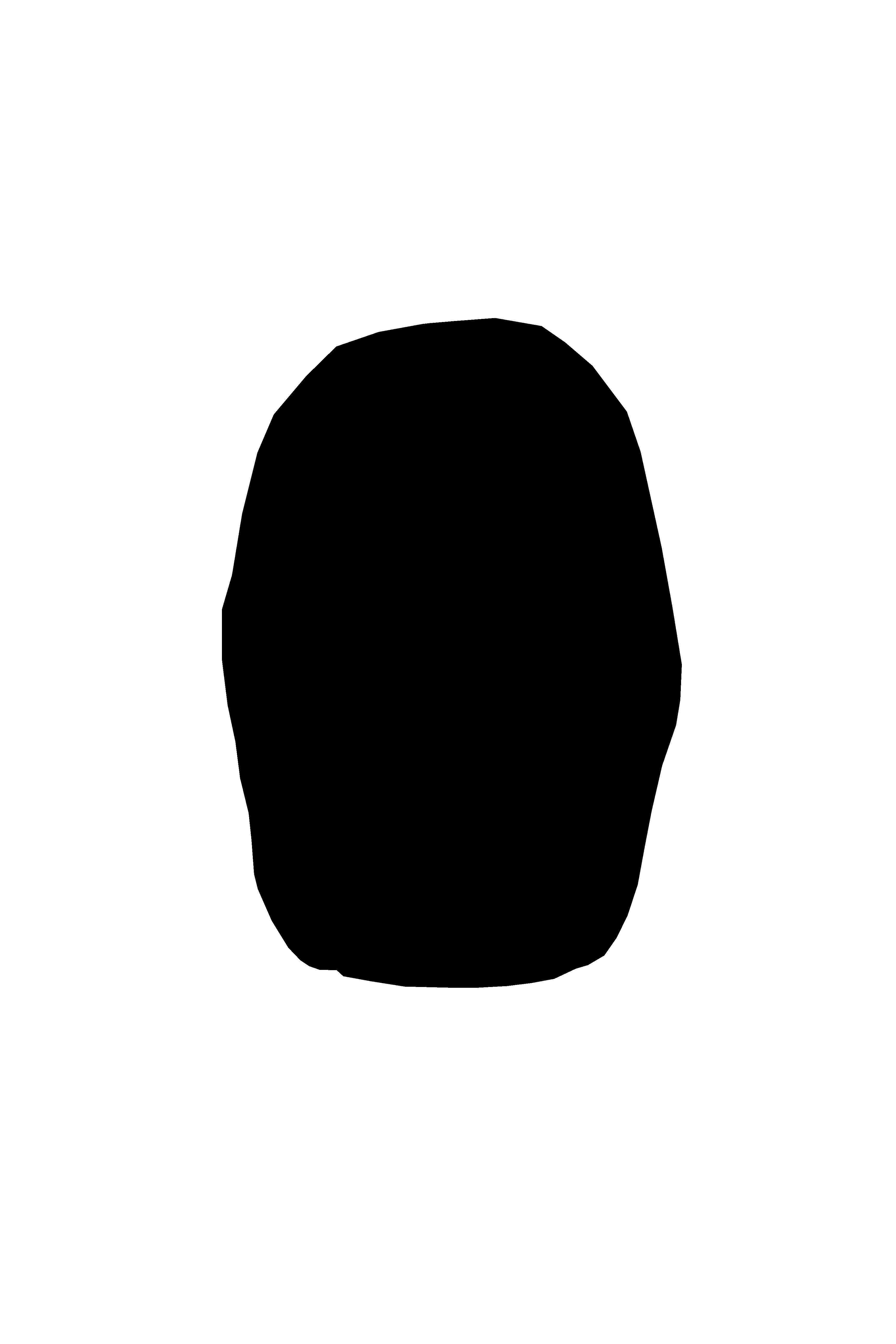

Supplement: Supplementary file 1 [file Data_Sheet_1.zip › face/033_face_mask.png]

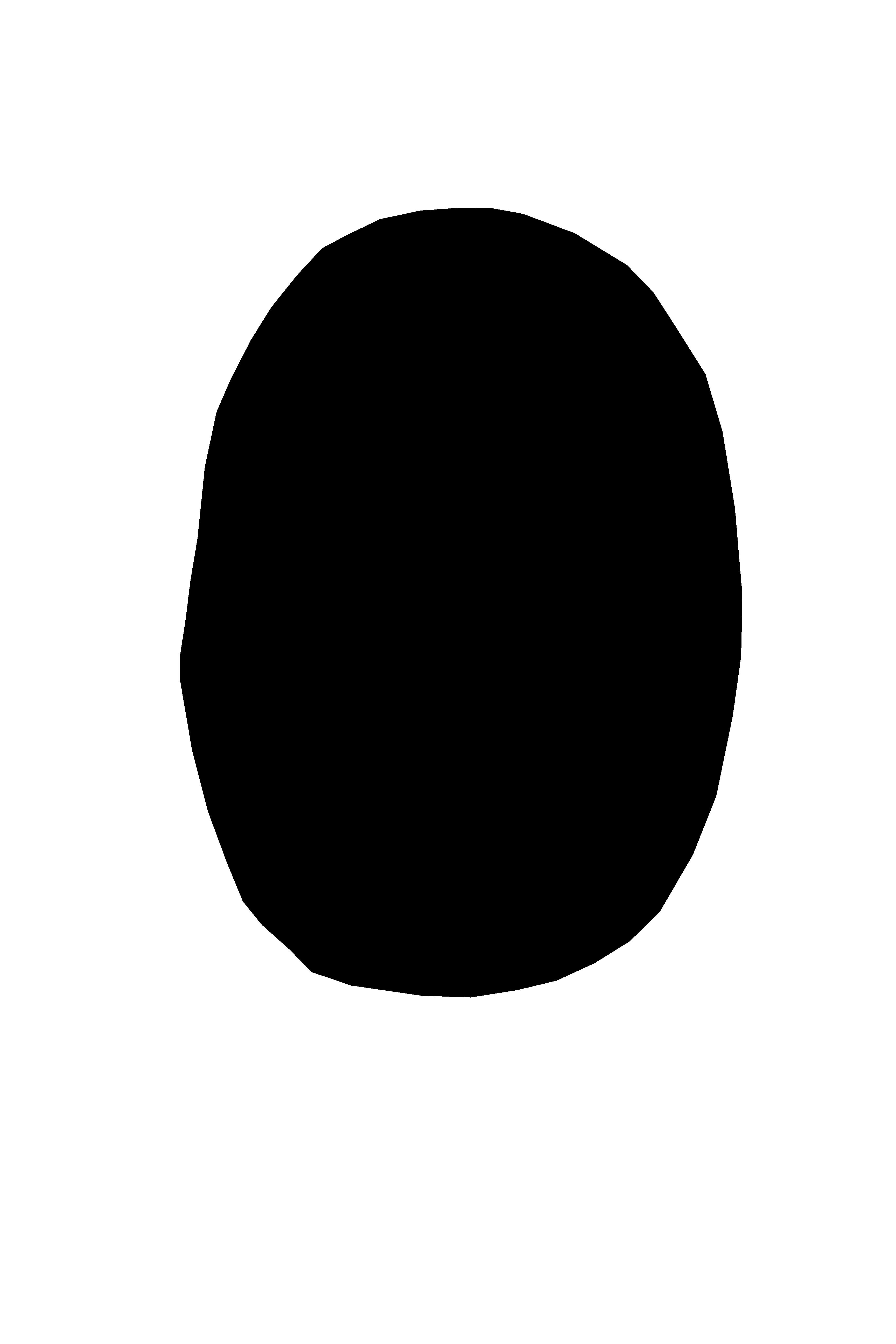

Supplement: Supplementary file 1 [file Data_Sheet_1.zip › face/034_face_mask.png]

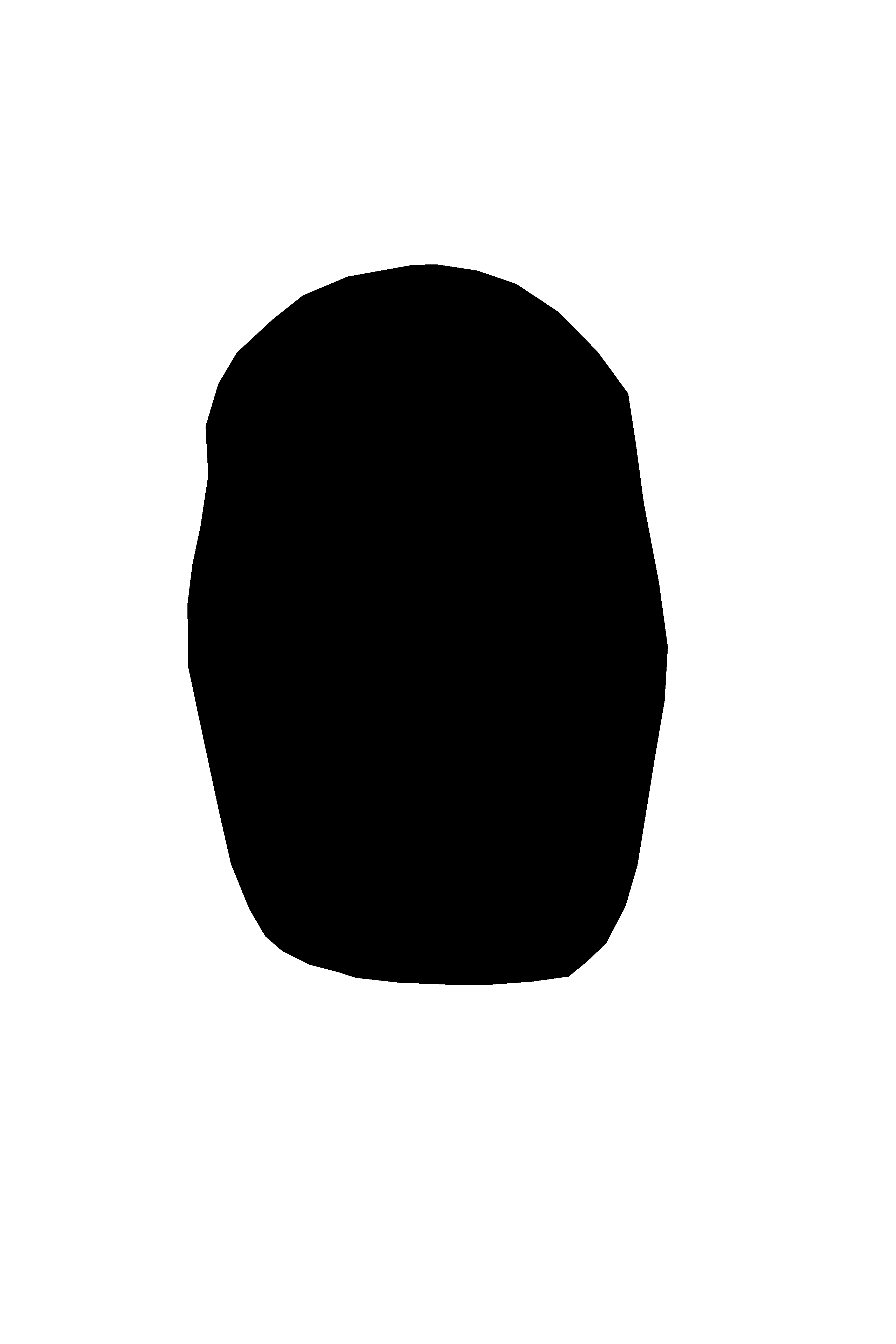

Supplement: Supplementary file 1 [file Data_Sheet_1.zip › face/035_face_mask.png]

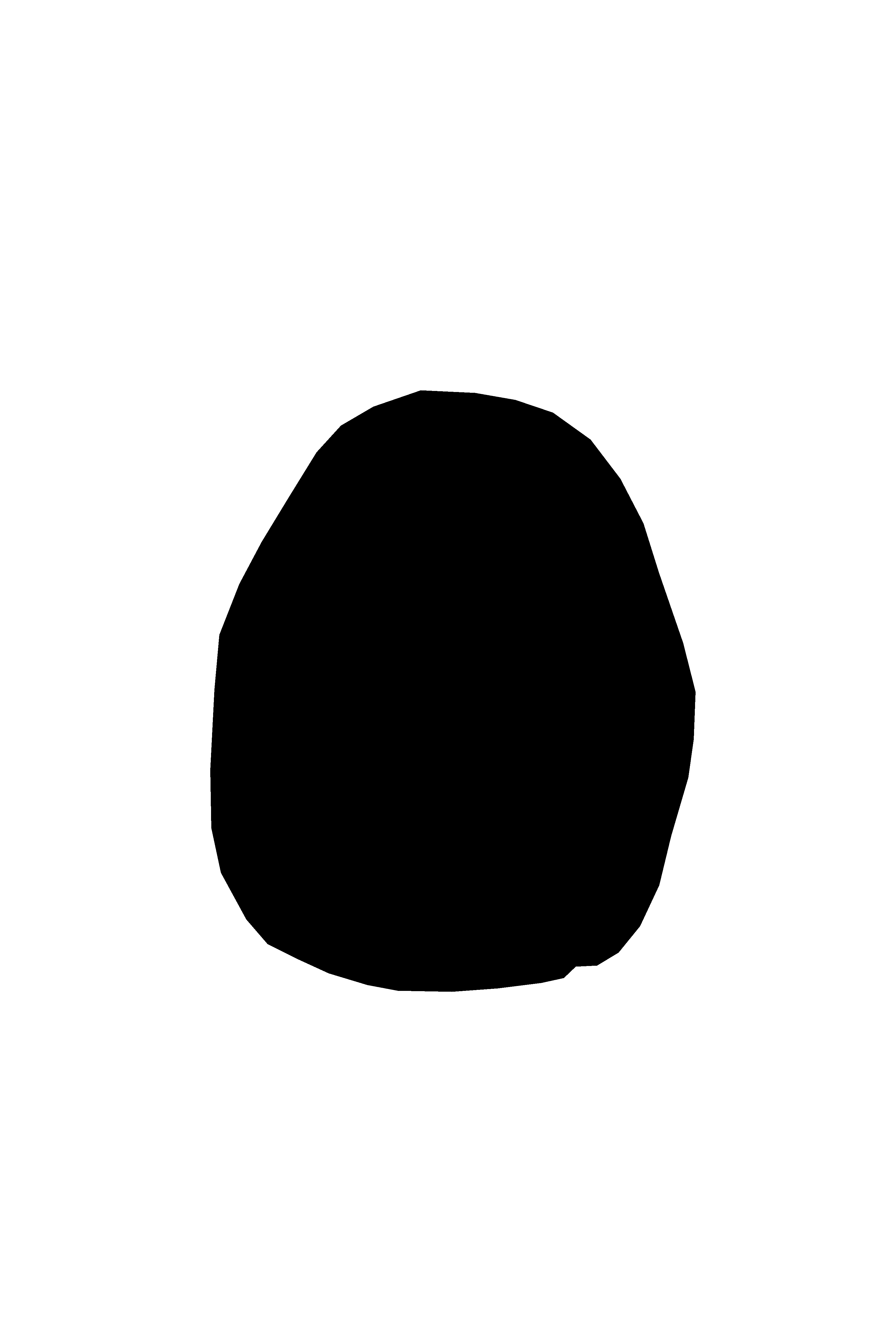

Supplement: Supplementary file 1 [file Data_Sheet_1.zip › face/036_face_mask.png]

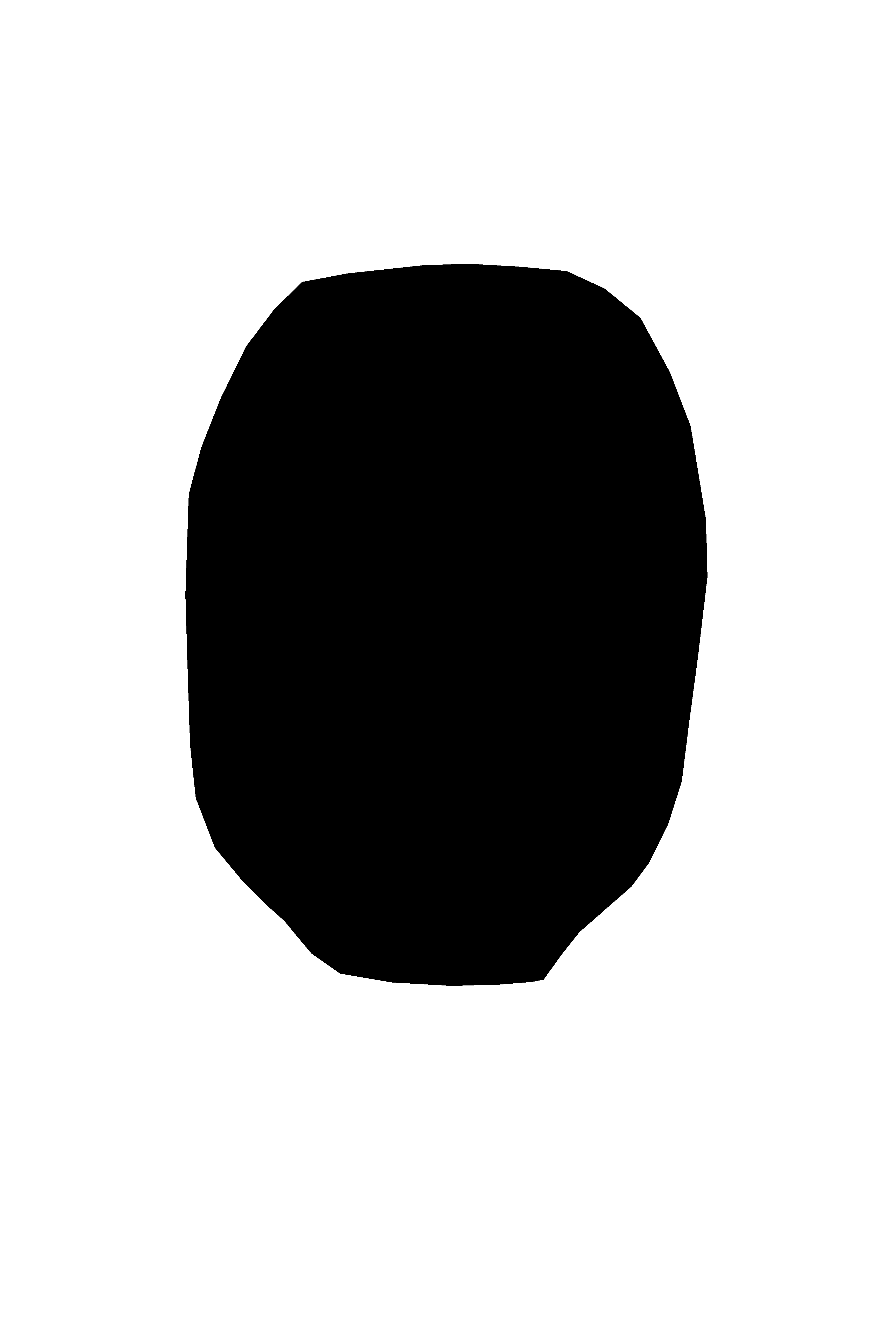

Supplement: Supplementary file 1 [file Data_Sheet_1.zip › face/037_face_mask.png]

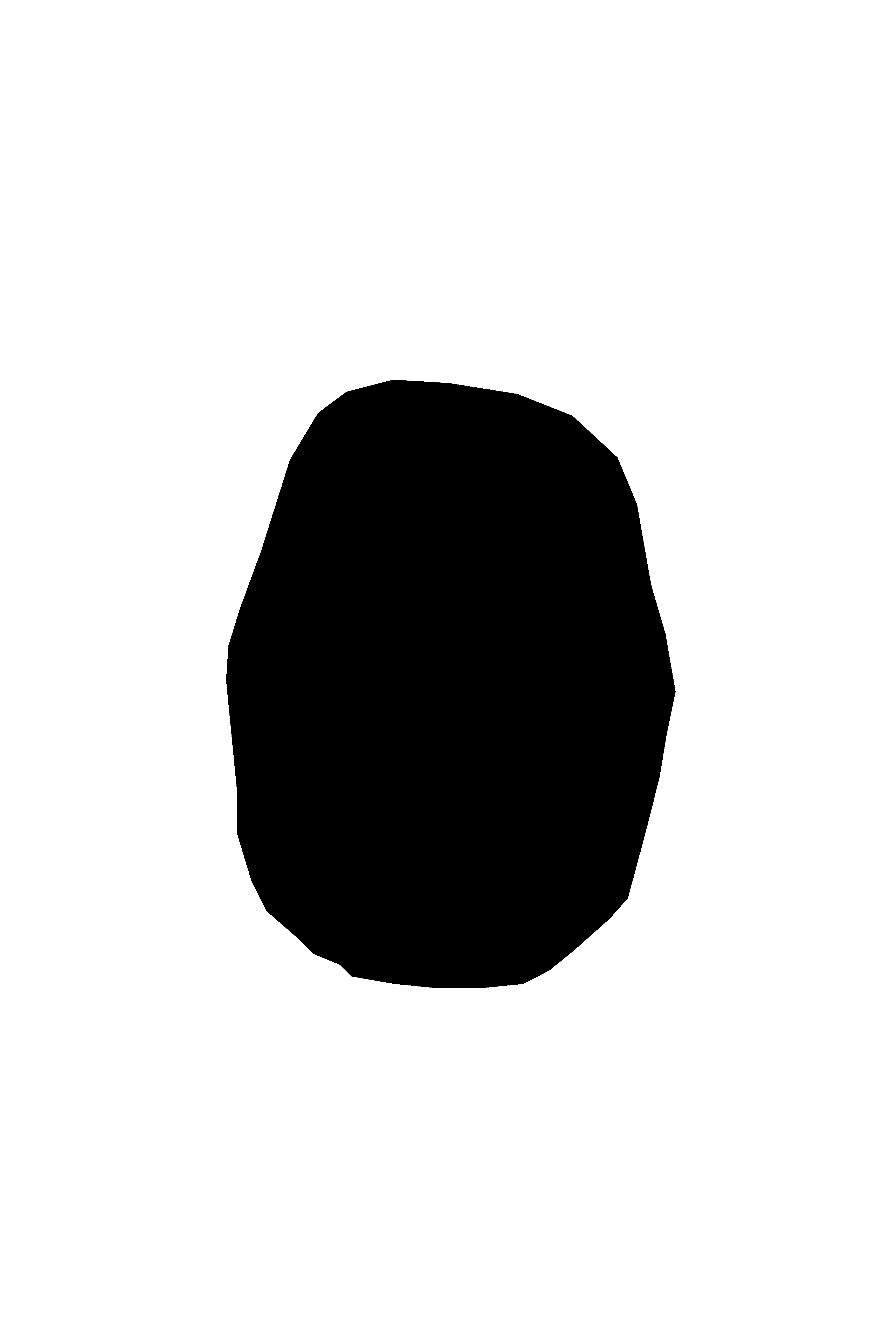

Supplement: Supplementary file 1 [file Data_Sheet_1.zip › face/038_face_mask.png]

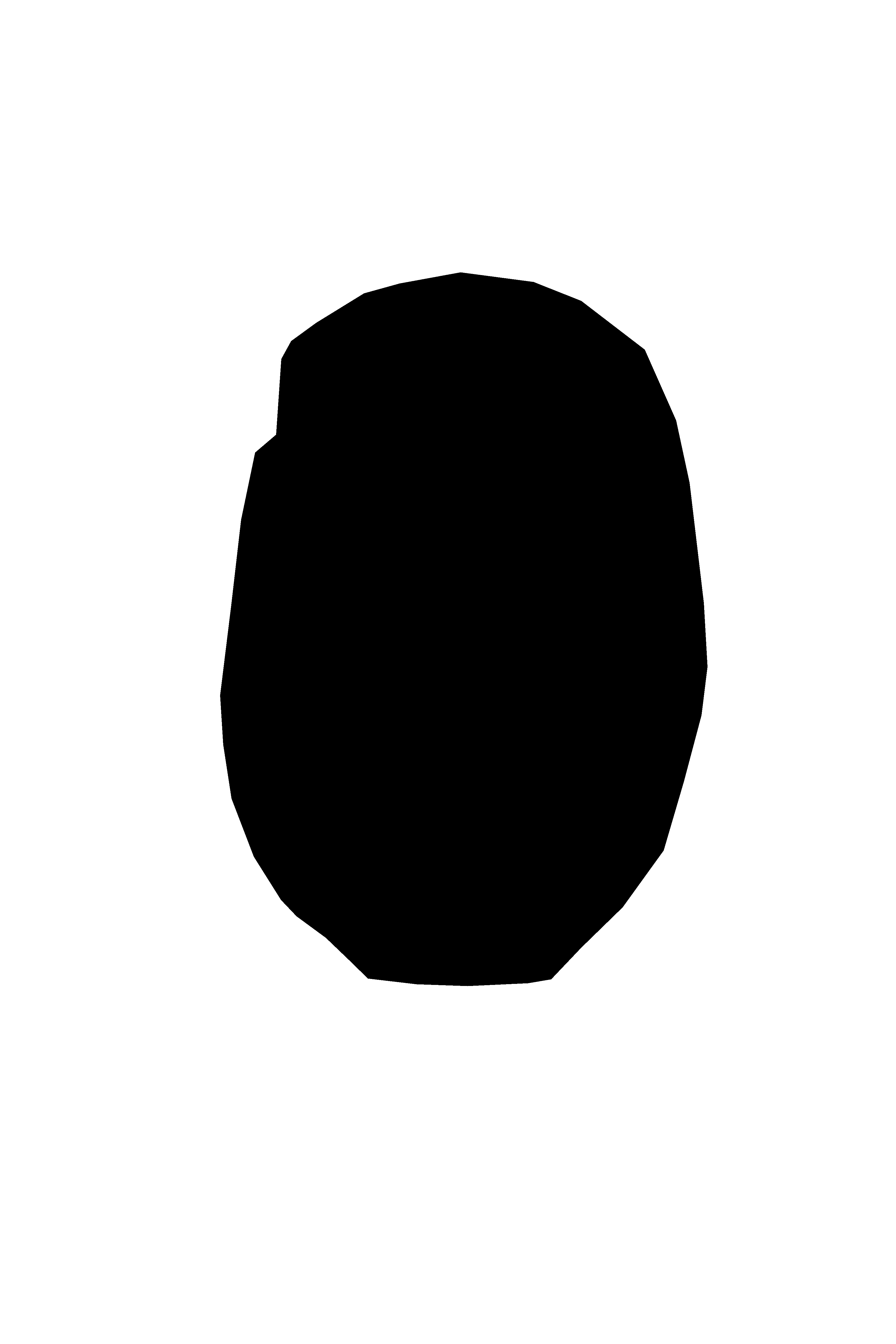

Supplement: Supplementary file 1 [file Data_Sheet_1.zip › face/039_face_mask.png]

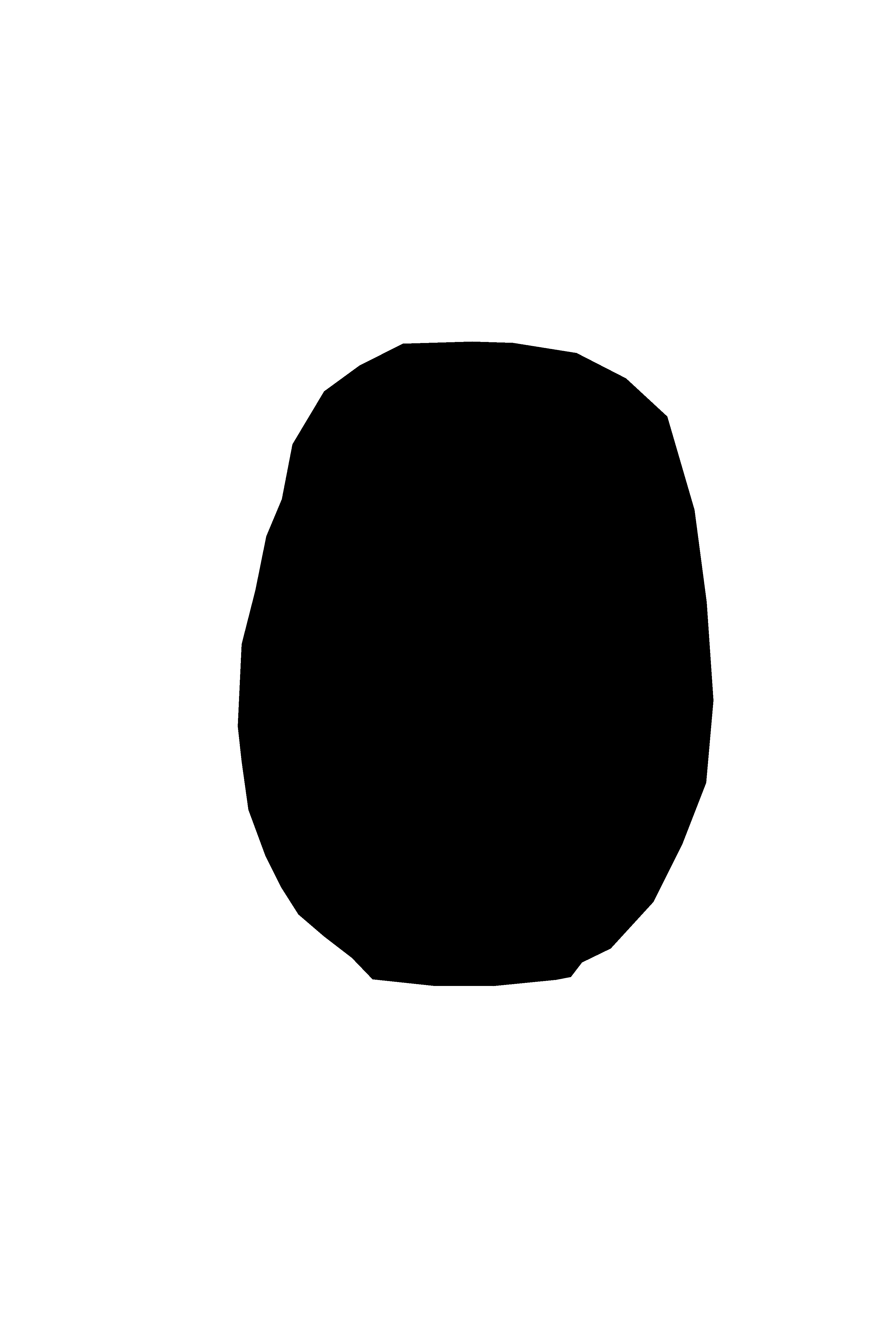

Supplement: Supplementary file 1 [file Data_Sheet_1.zip › face/040_face_mask.png]

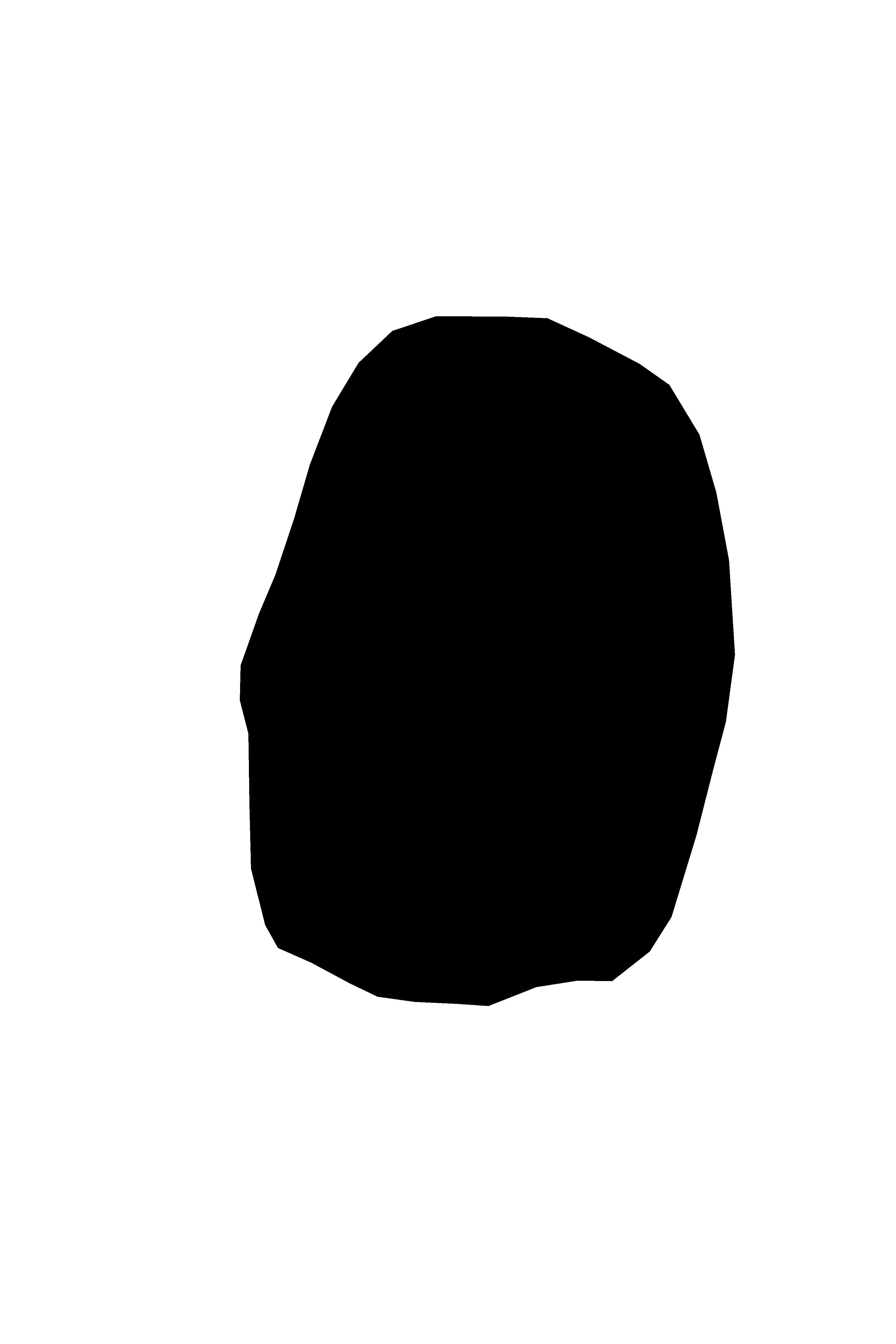

Supplement: Supplementary file 1 [file Data_Sheet_1.zip › face/041_face_mask.png]

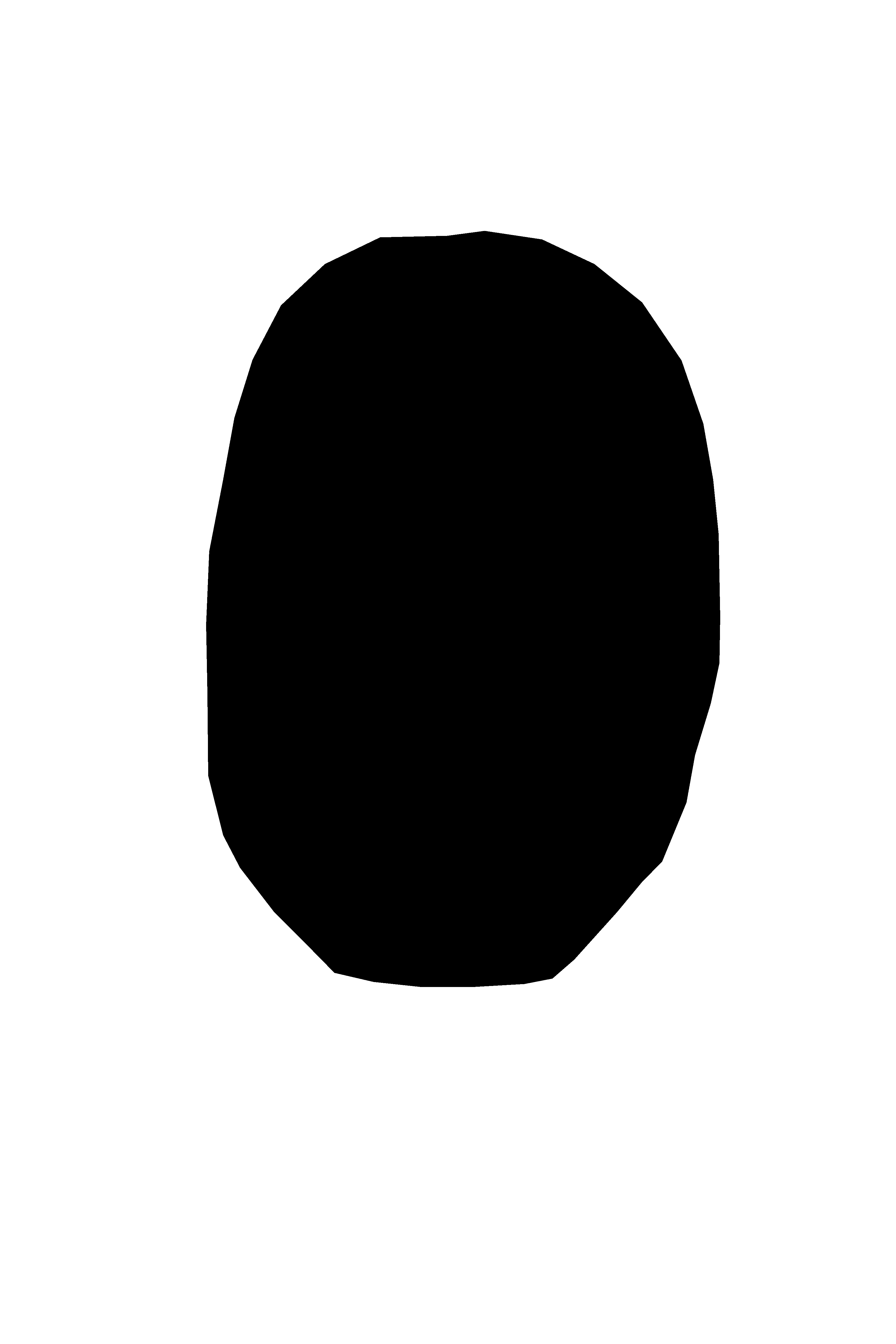

Supplement: Supplementary file 1 [file Data_Sheet_1.zip › face/042_face_mask.png]

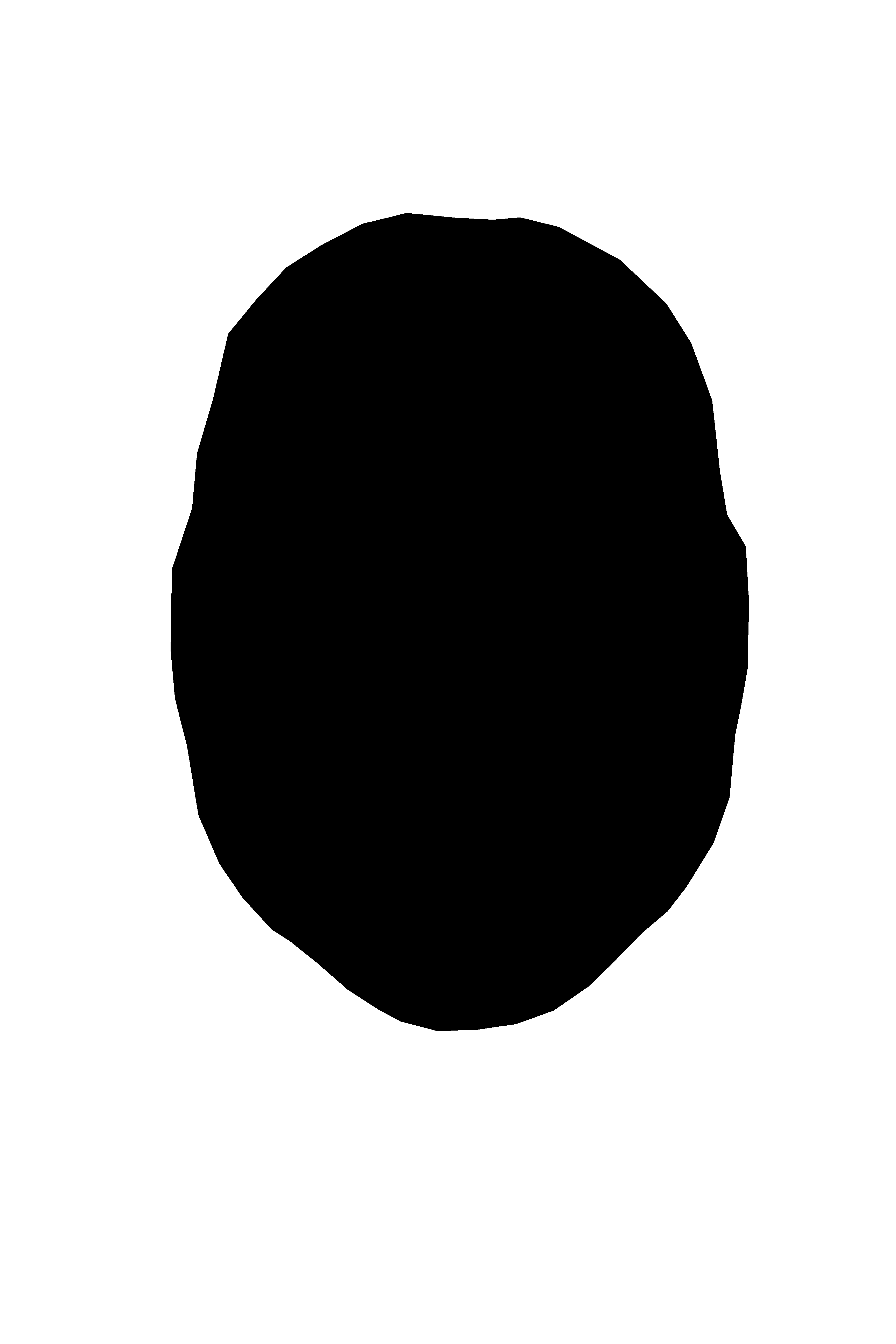

Supplement: Supplementary file 1 [file Data_Sheet_1.zip › face/043_face_mask.png]

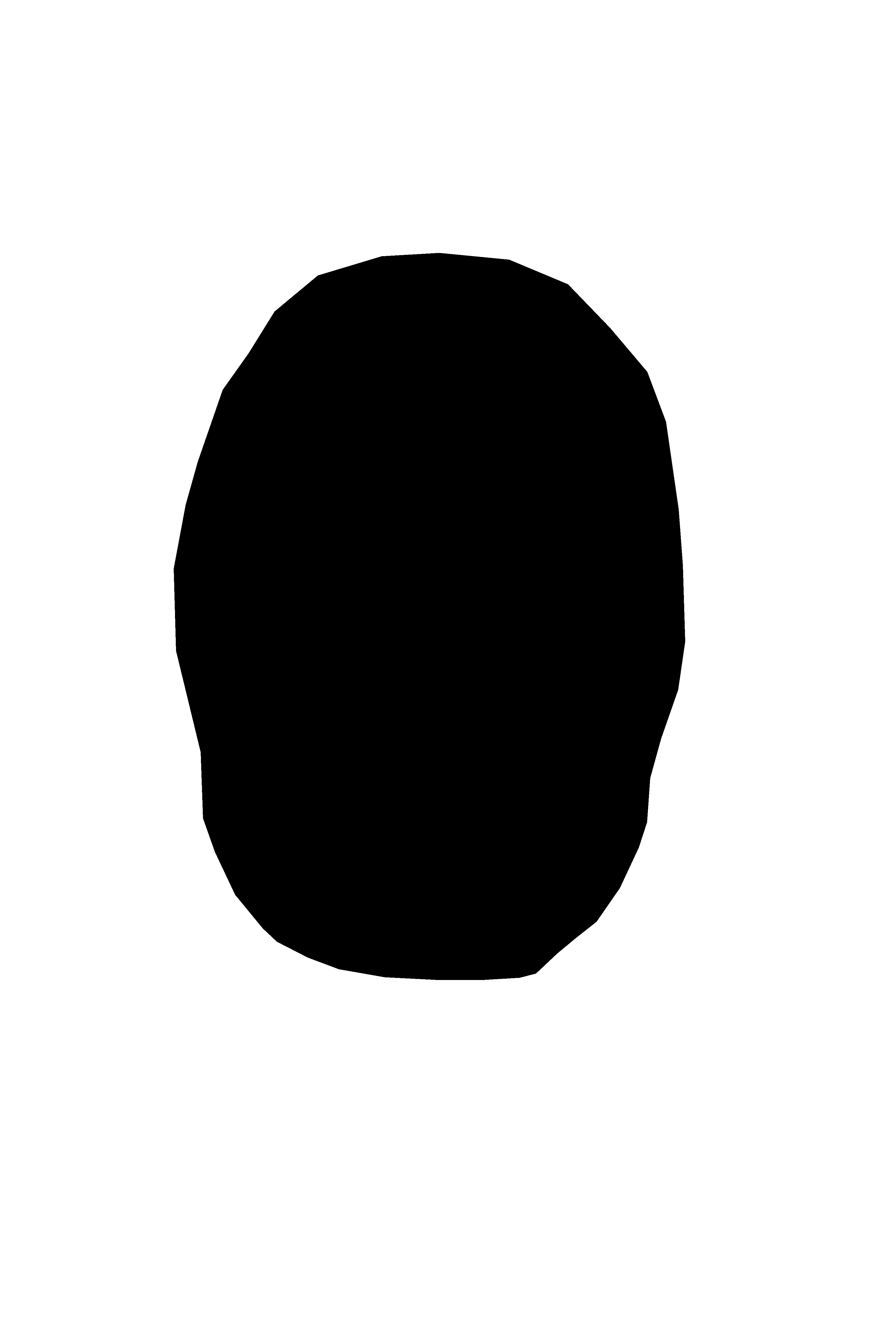

Supplement: Supplementary file 1 [file Data_Sheet_1.zip › face/044_face_mask.png]

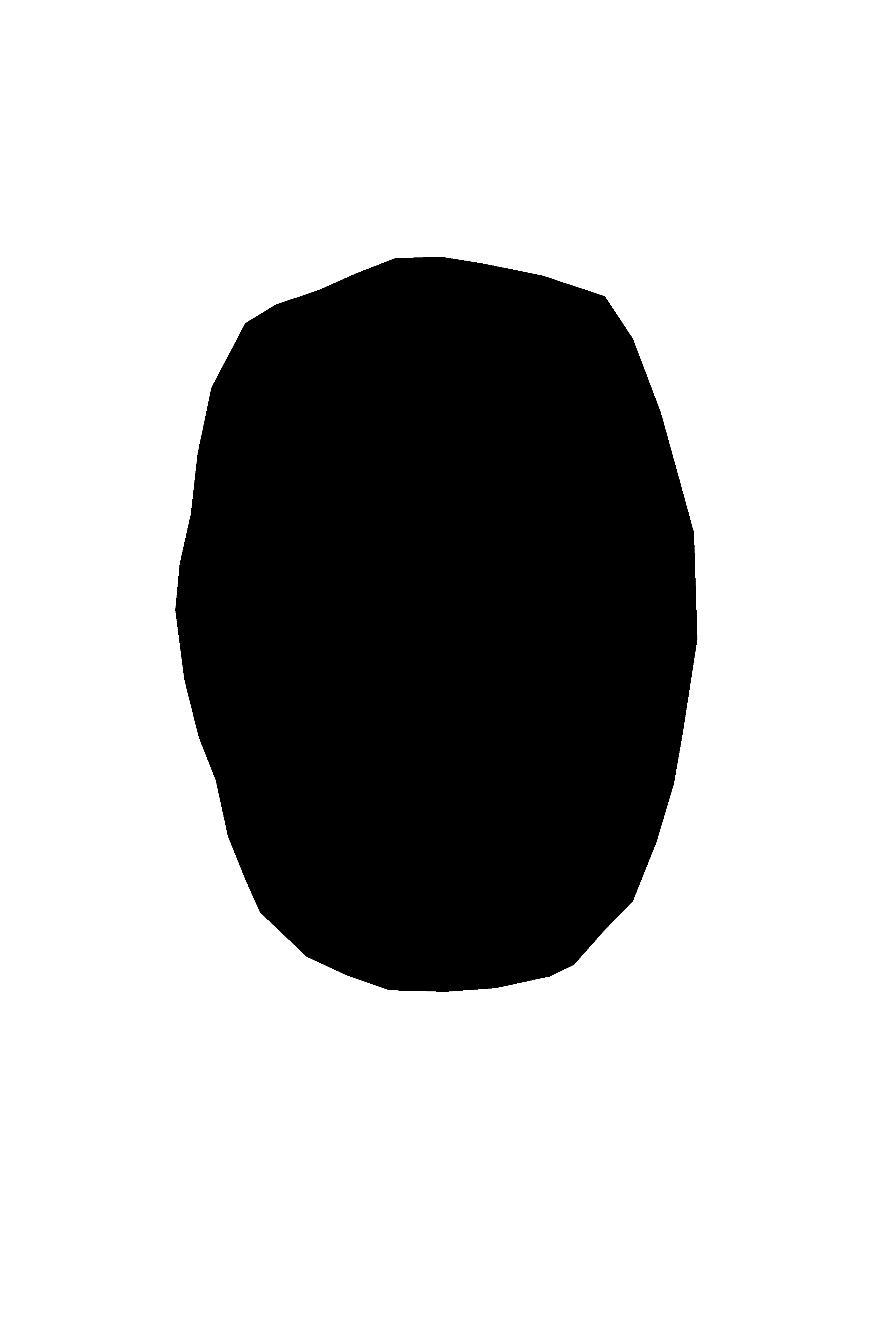

Supplement: Supplementary file 1 [file Data_Sheet_1.zip › face/045_face_mask.png]

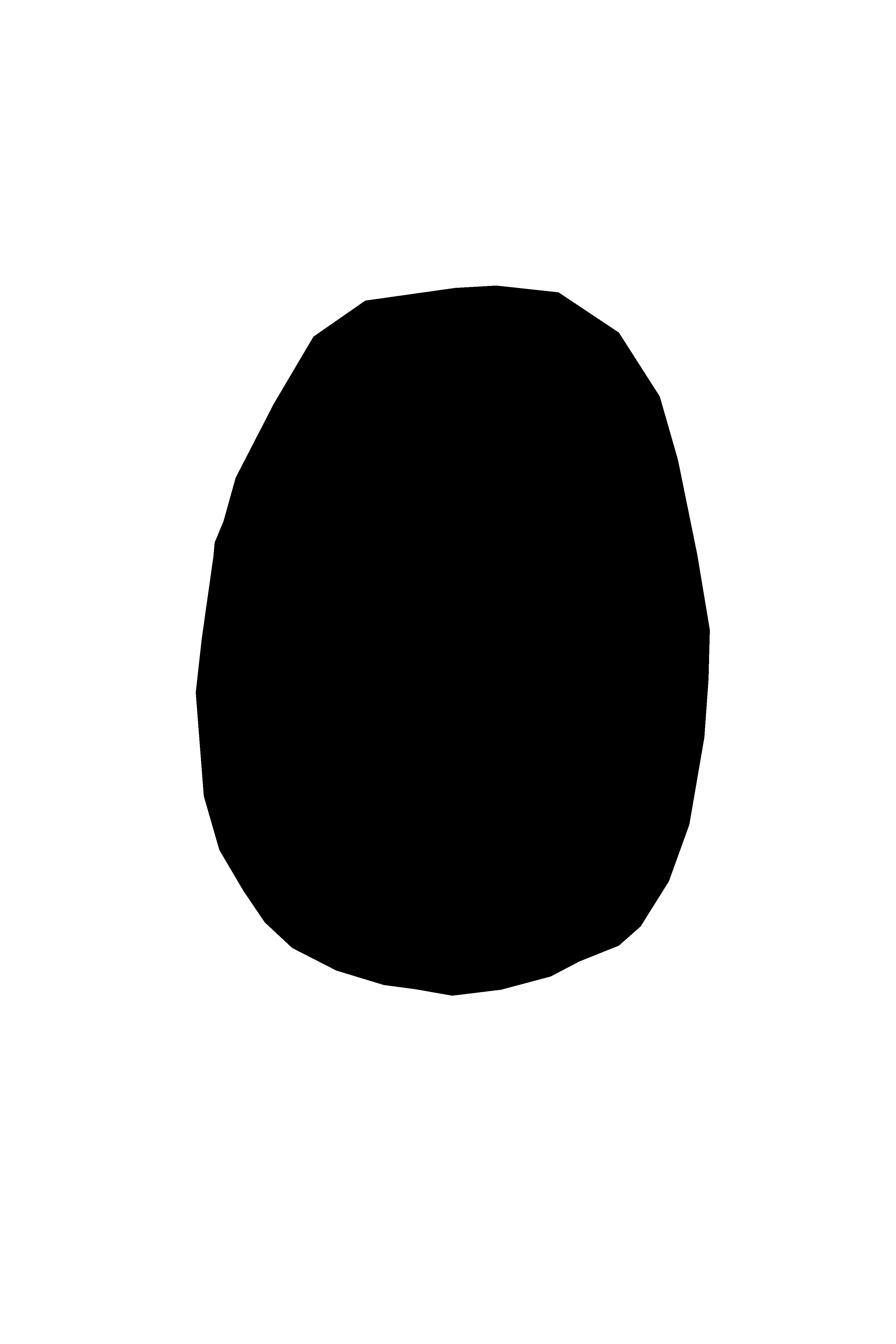

Supplement: Supplementary file 1 [file Data_Sheet_1.zip › face/046_face_mask.png]

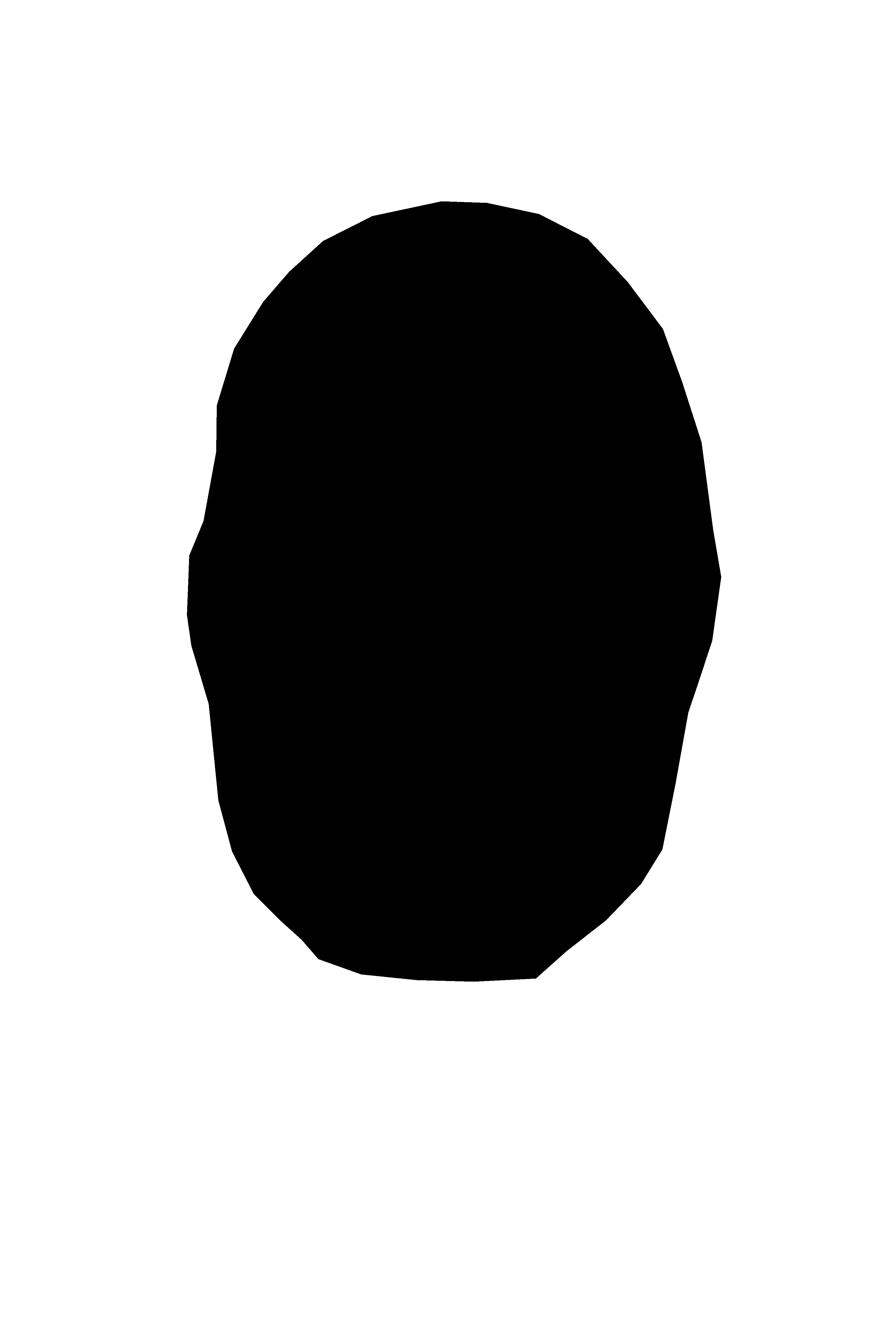

Supplement: Supplementary file 1 [file Data_Sheet_1.zip › face/047_face_mask.png]

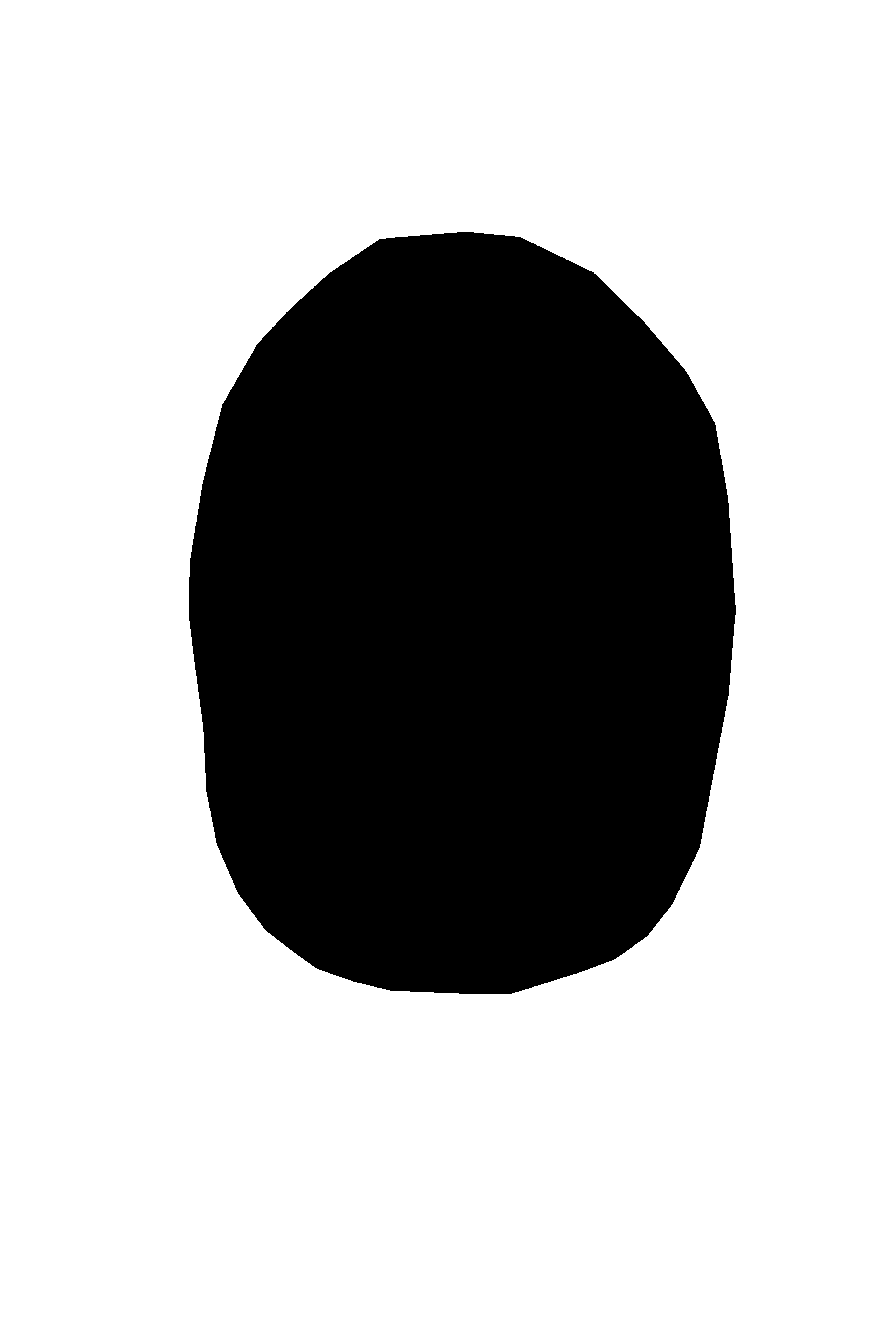

Supplement: Supplementary file 1 [file Data_Sheet_1.zip › face/048_face_mask.png]

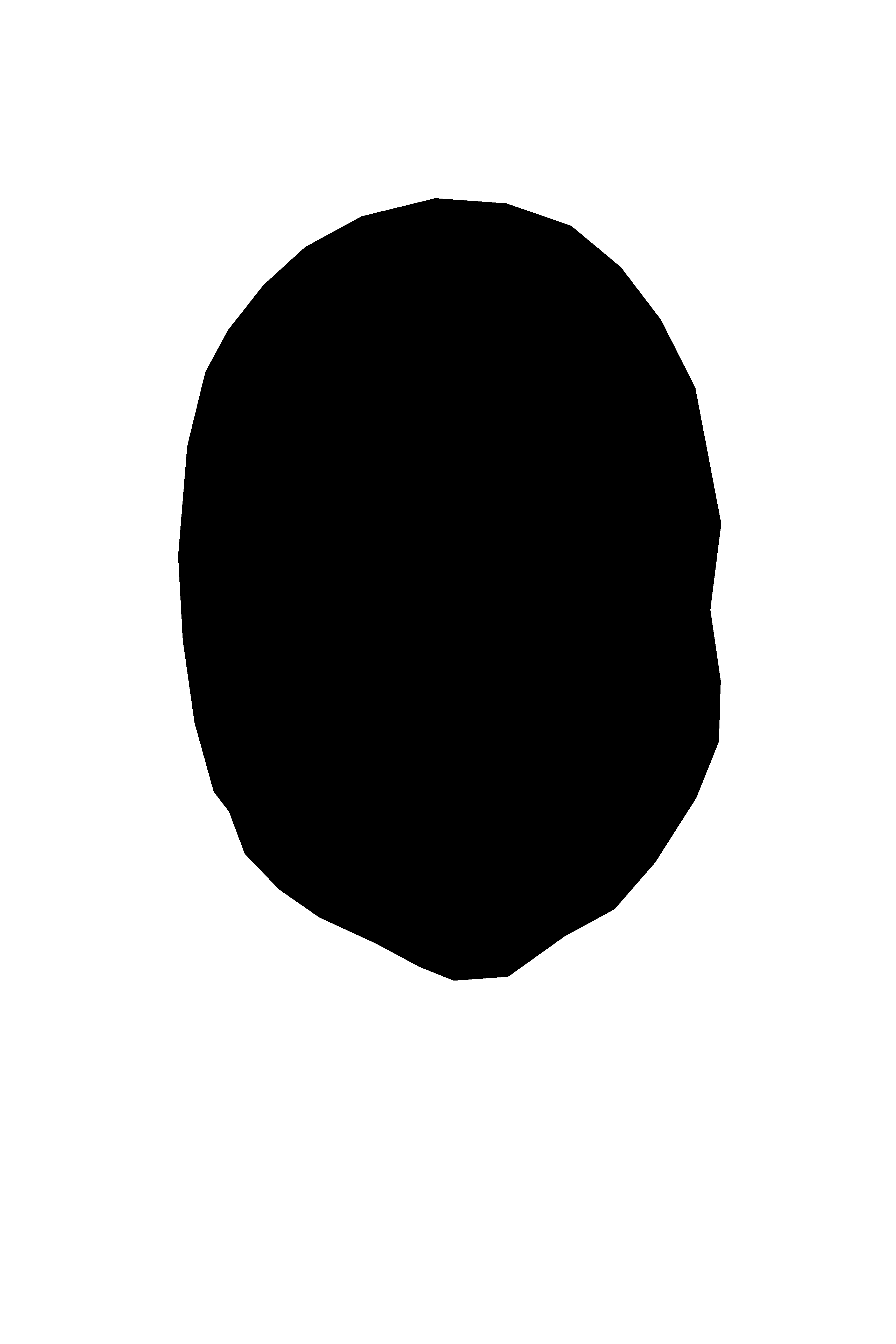

Supplement: Supplementary file 1 [file Data_Sheet_1.zip › face/049_face_mask.png]

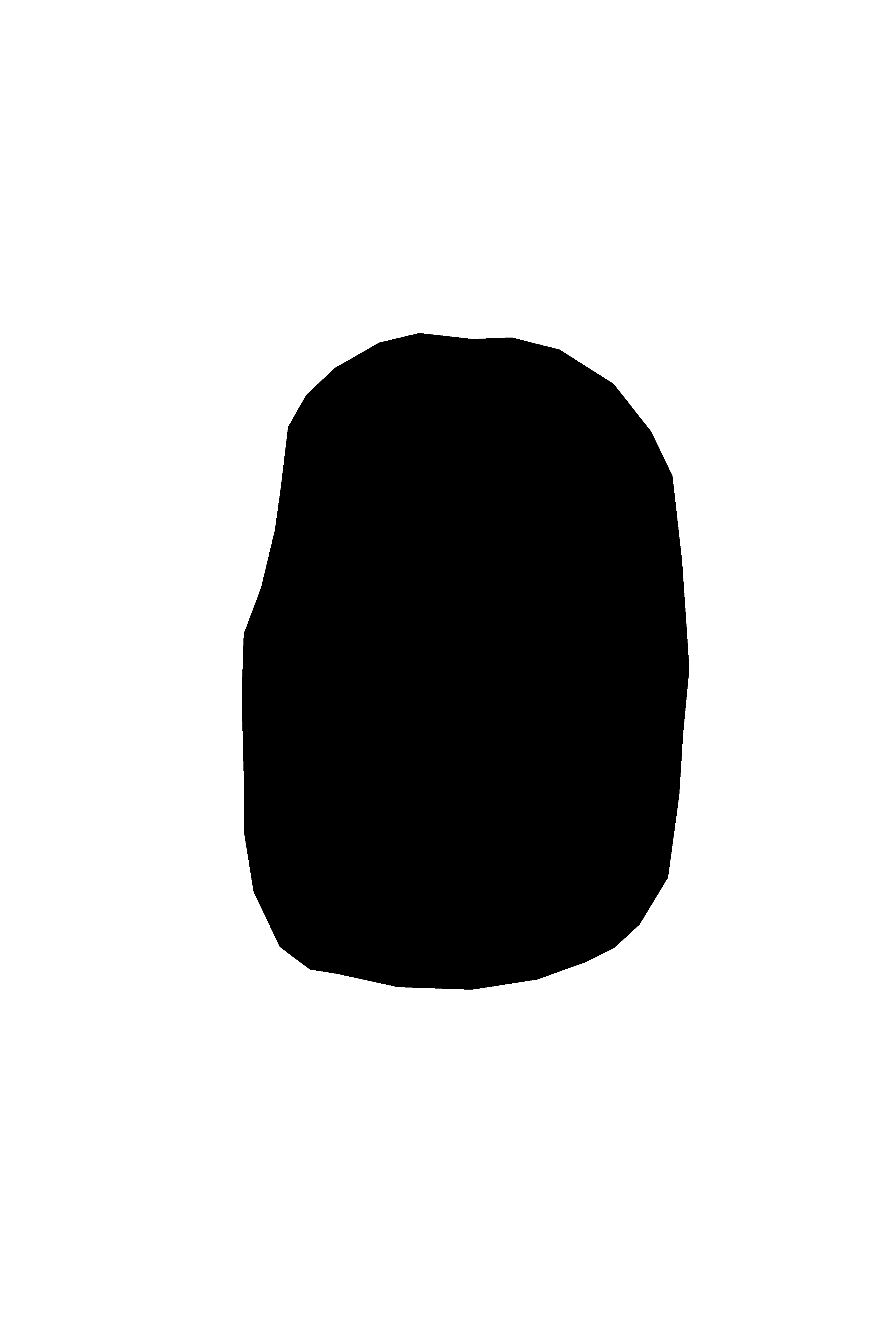

Supplement: Supplementary file 1 [file Data_Sheet_1.zip › face/050_face_mask.png]

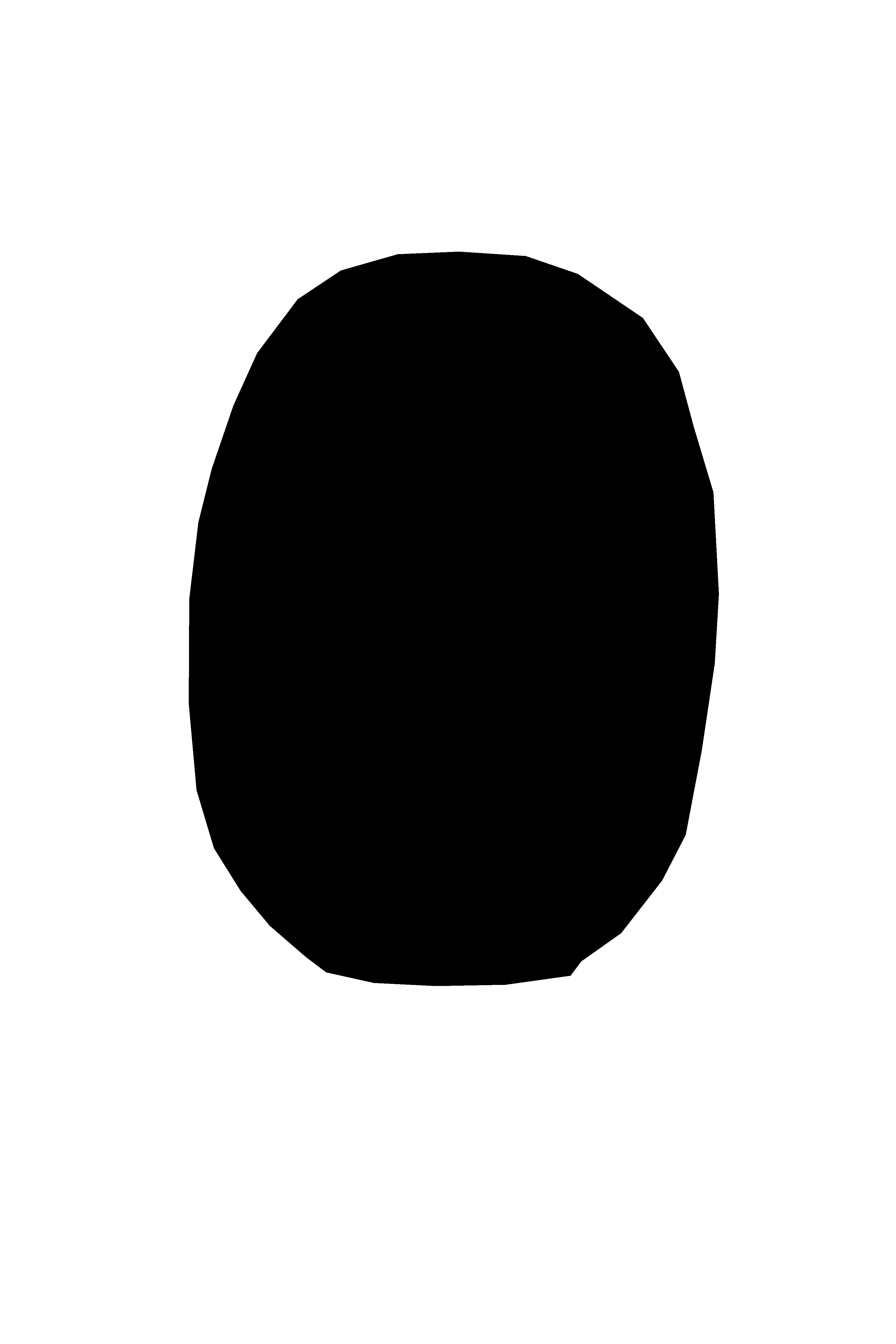

Supplement: Supplementary file 1 [file Data_Sheet_1.zip › face/051_face_mask.png]

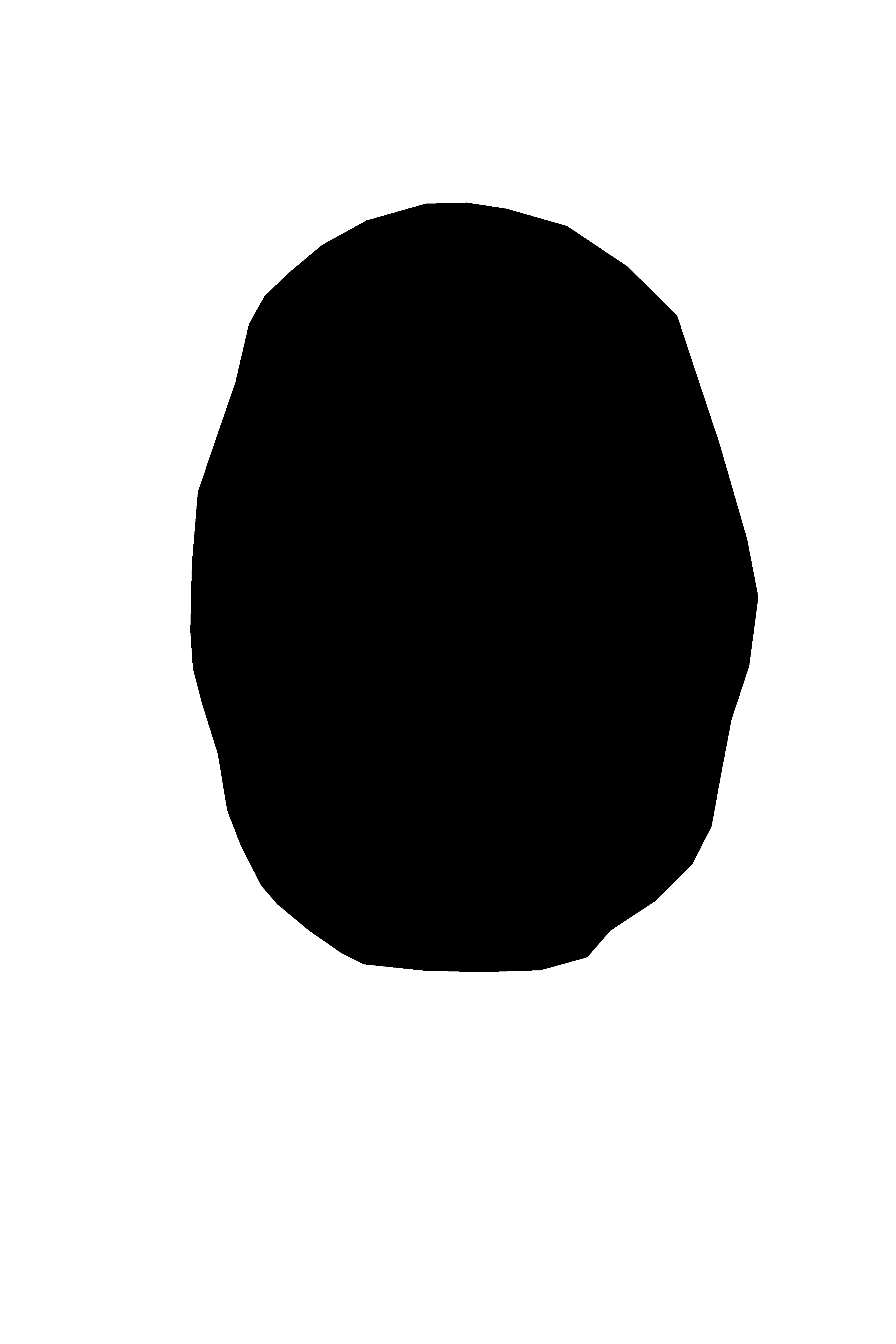

Supplement: Supplementary file 1 [file Data_Sheet_1.zip › face/052_face_mask.png]

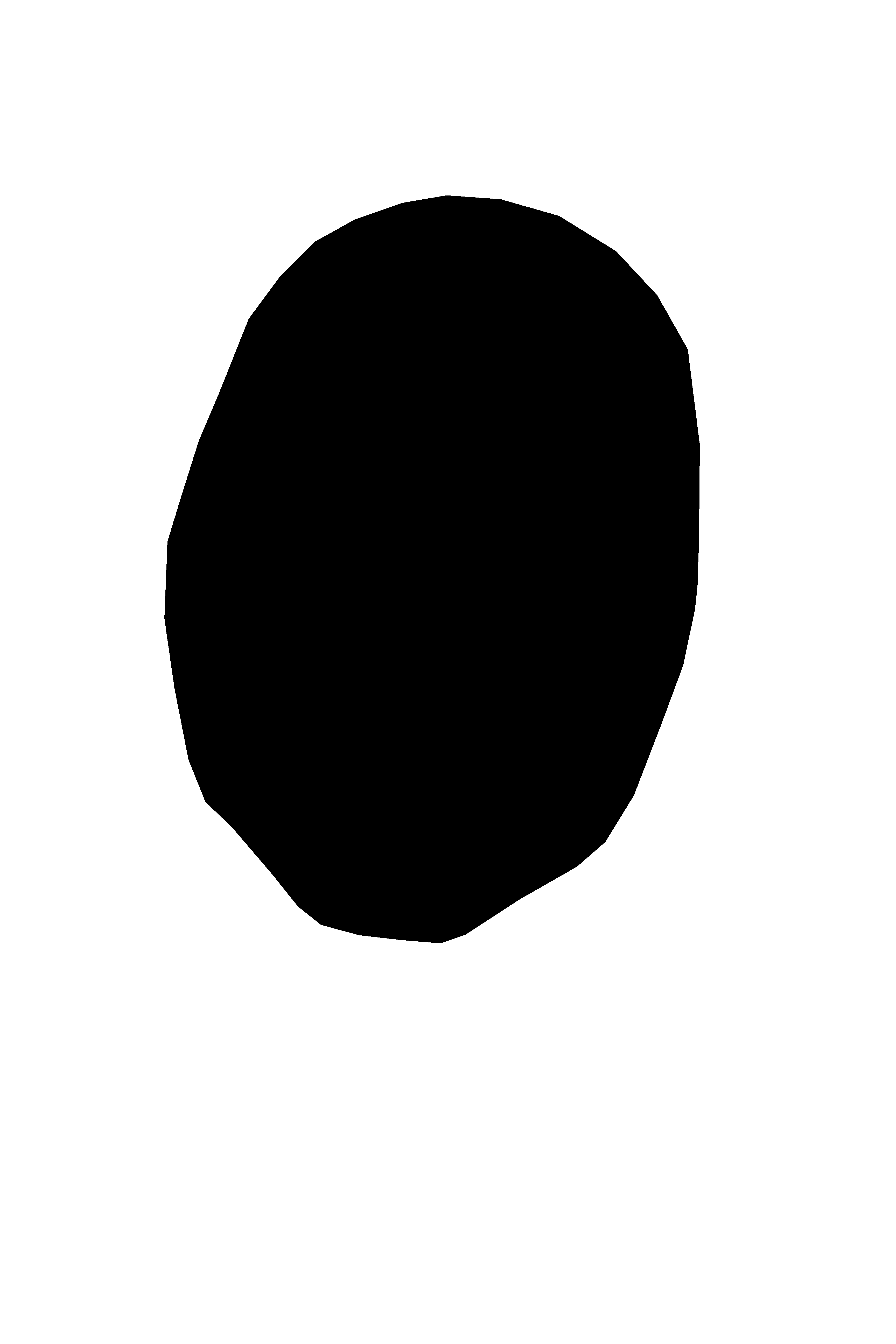

Supplement: Supplementary file 1 [file Data_Sheet_1.zip › face/053_face_mask.png]

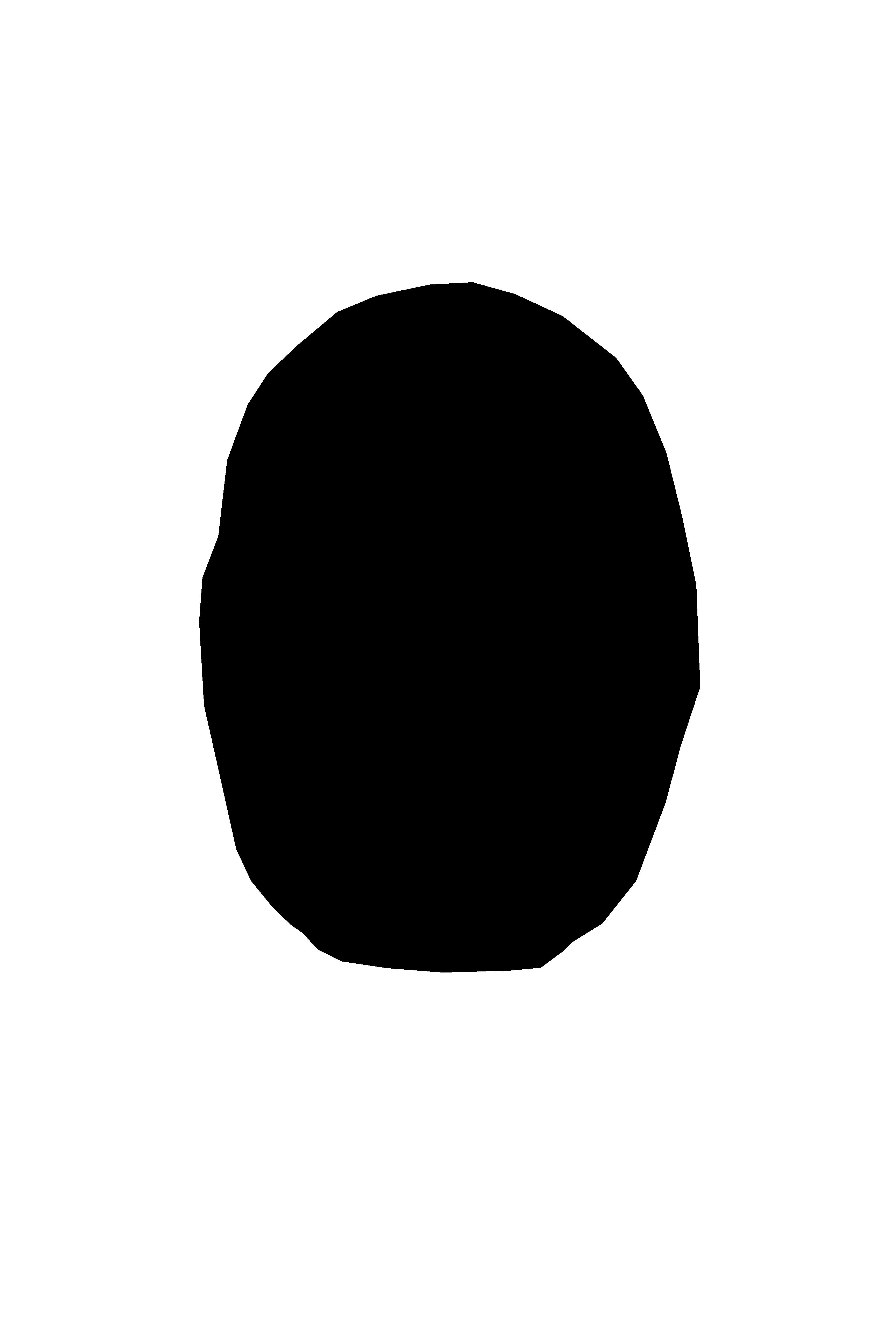

Supplement: Supplementary file 1 [file Data_Sheet_1.zip › face/054_face_mask.png]

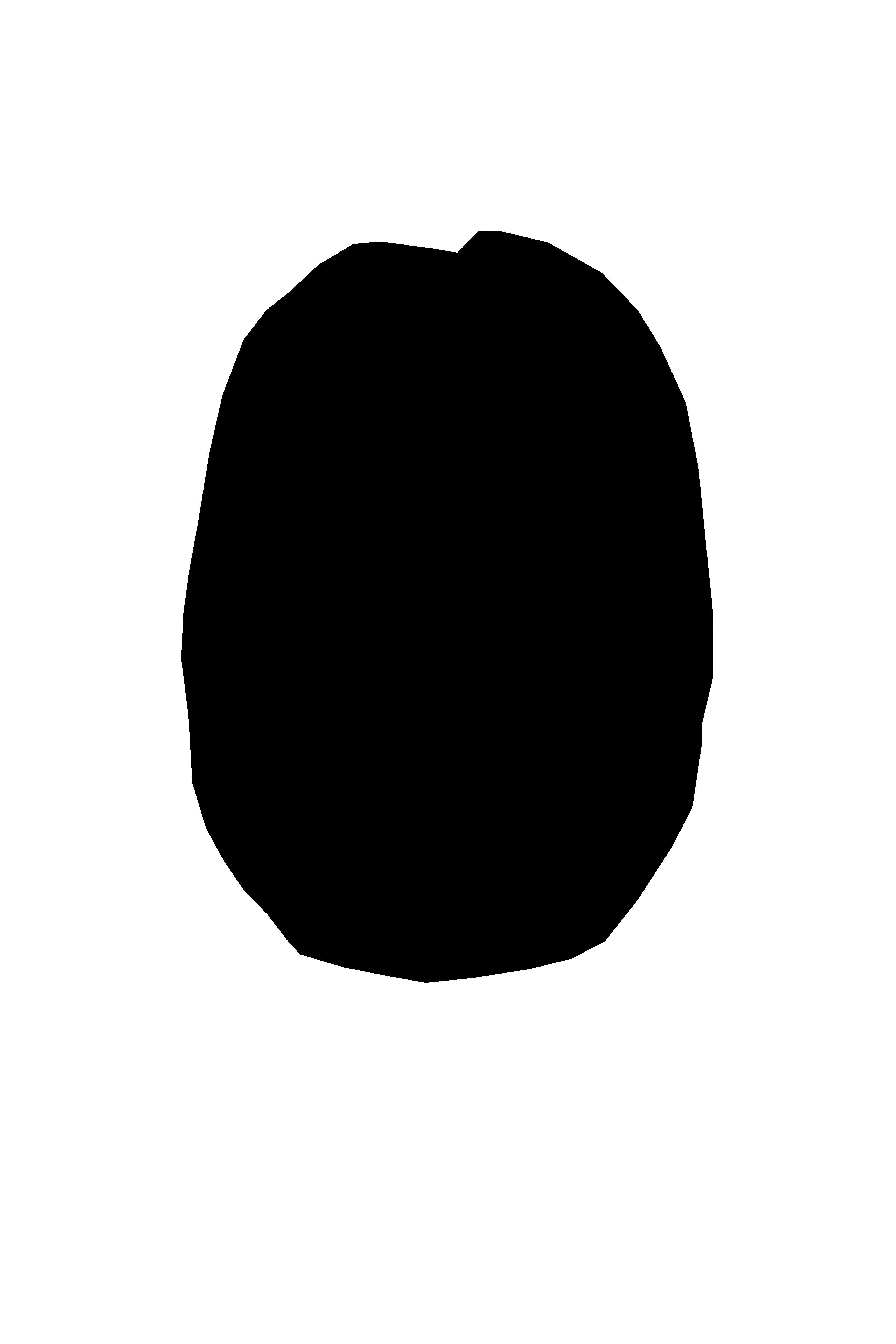

Supplement: Supplementary file 1 [file Data_Sheet_1.zip › face/055_face_mask.png]

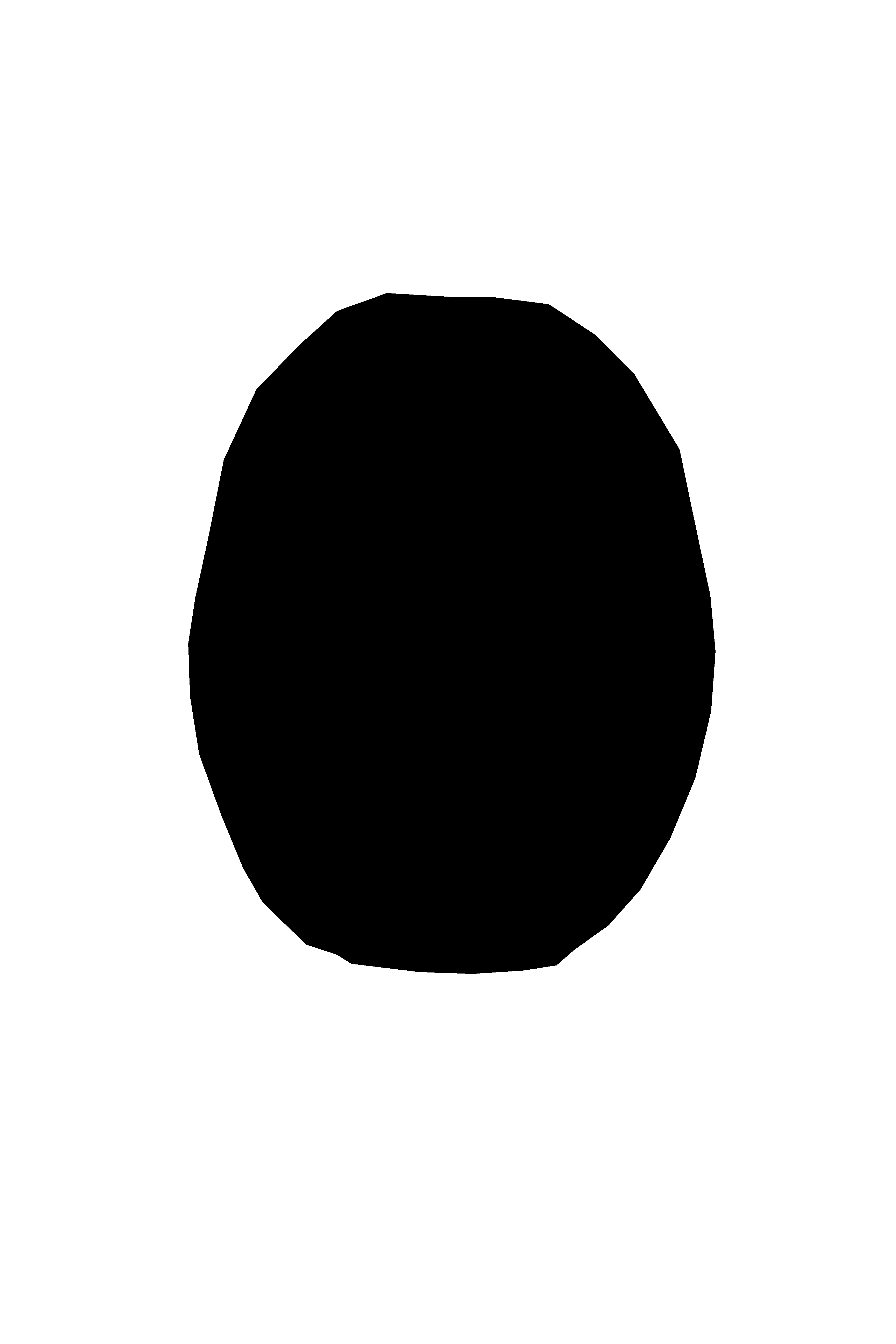

Supplement: Supplementary file 1 [file Data_Sheet_1.zip › face/056_face_mask.png]

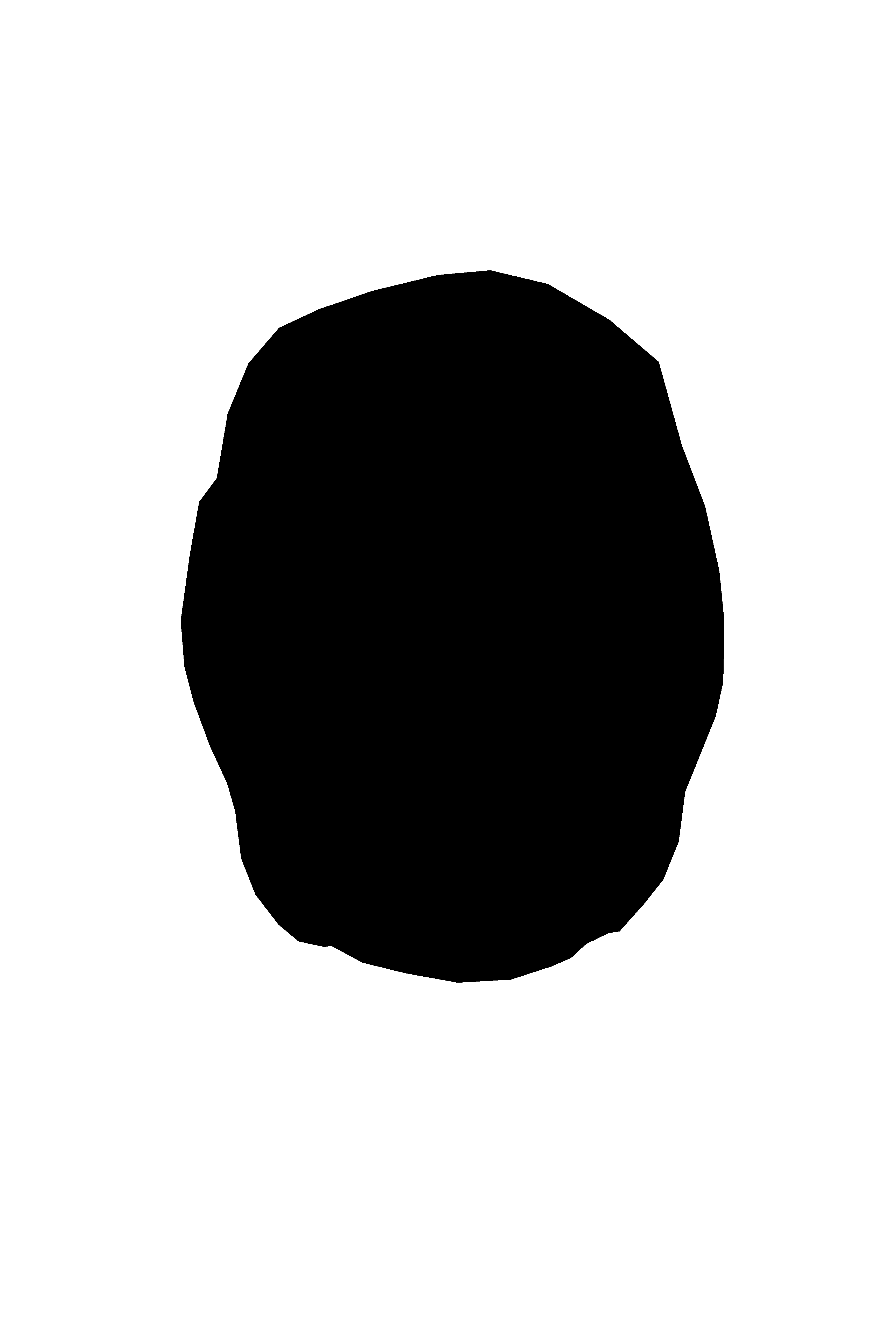

Supplement: Supplementary file 1 [file Data_Sheet_1.zip › face/057_face_mask.png]

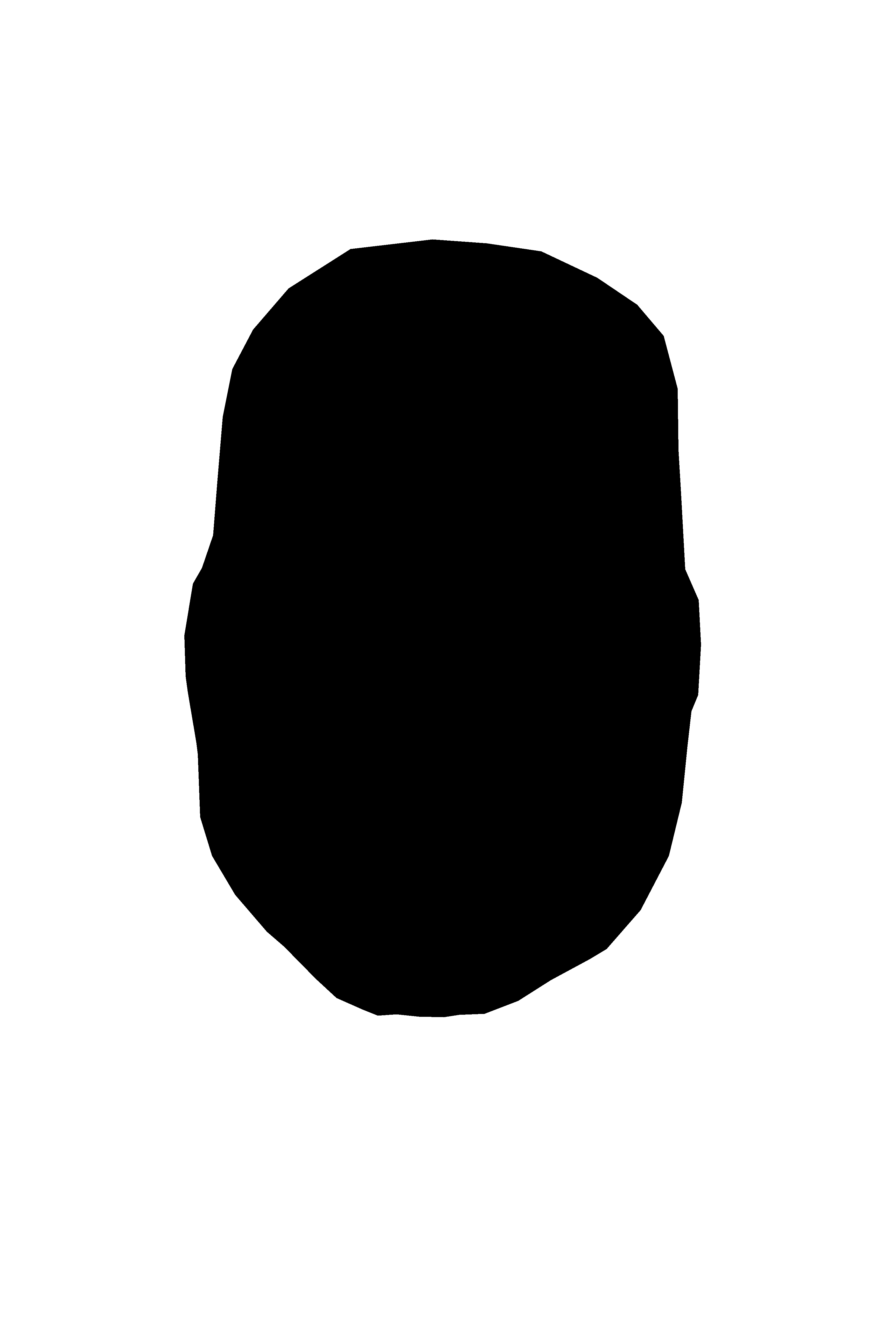

Supplement: Supplementary file 1 [file Data_Sheet_1.zip › face/058_face_mask.png]

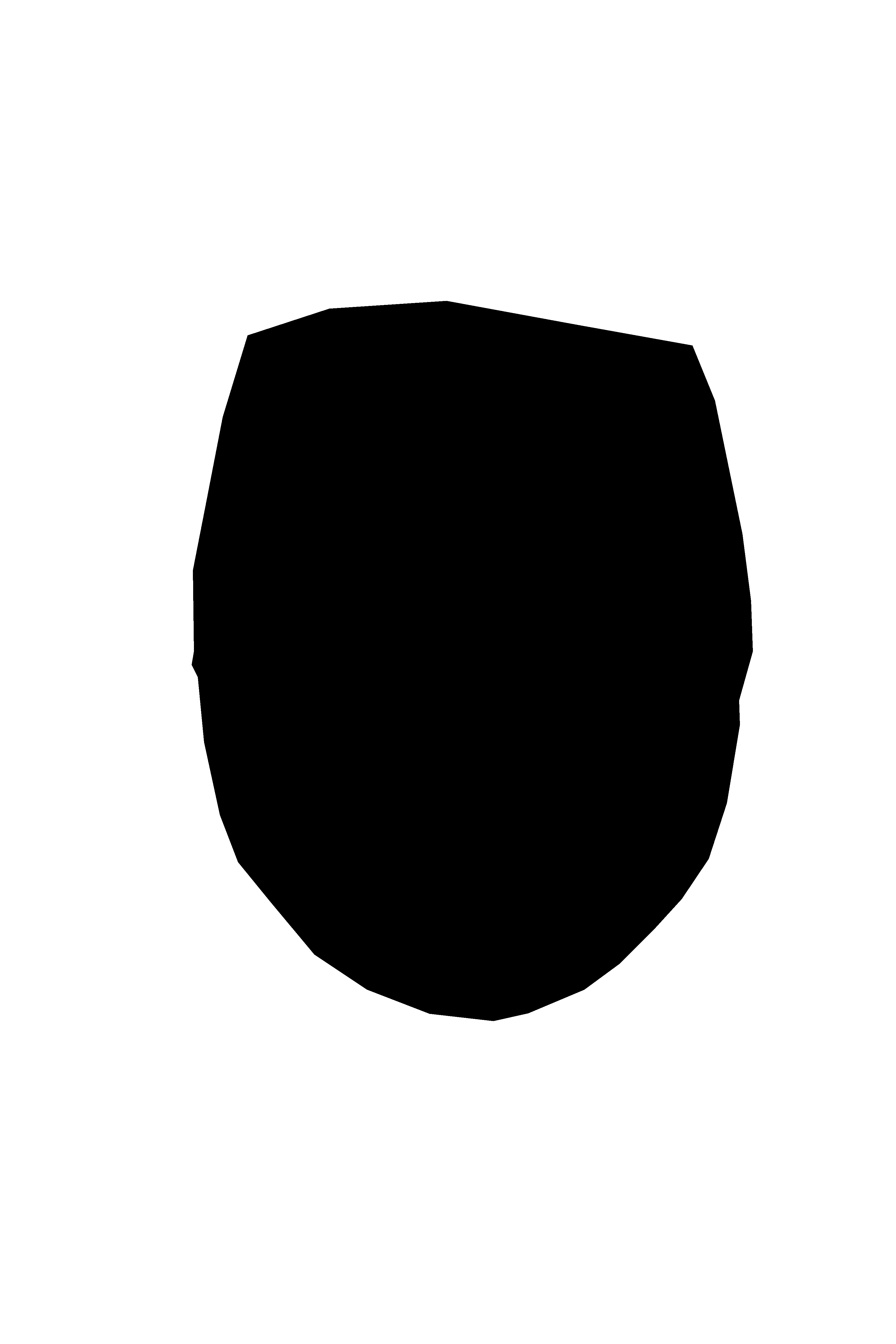

Supplement: Supplementary file 1 [file Data_Sheet_1.zip › face/059_face_mask.png]

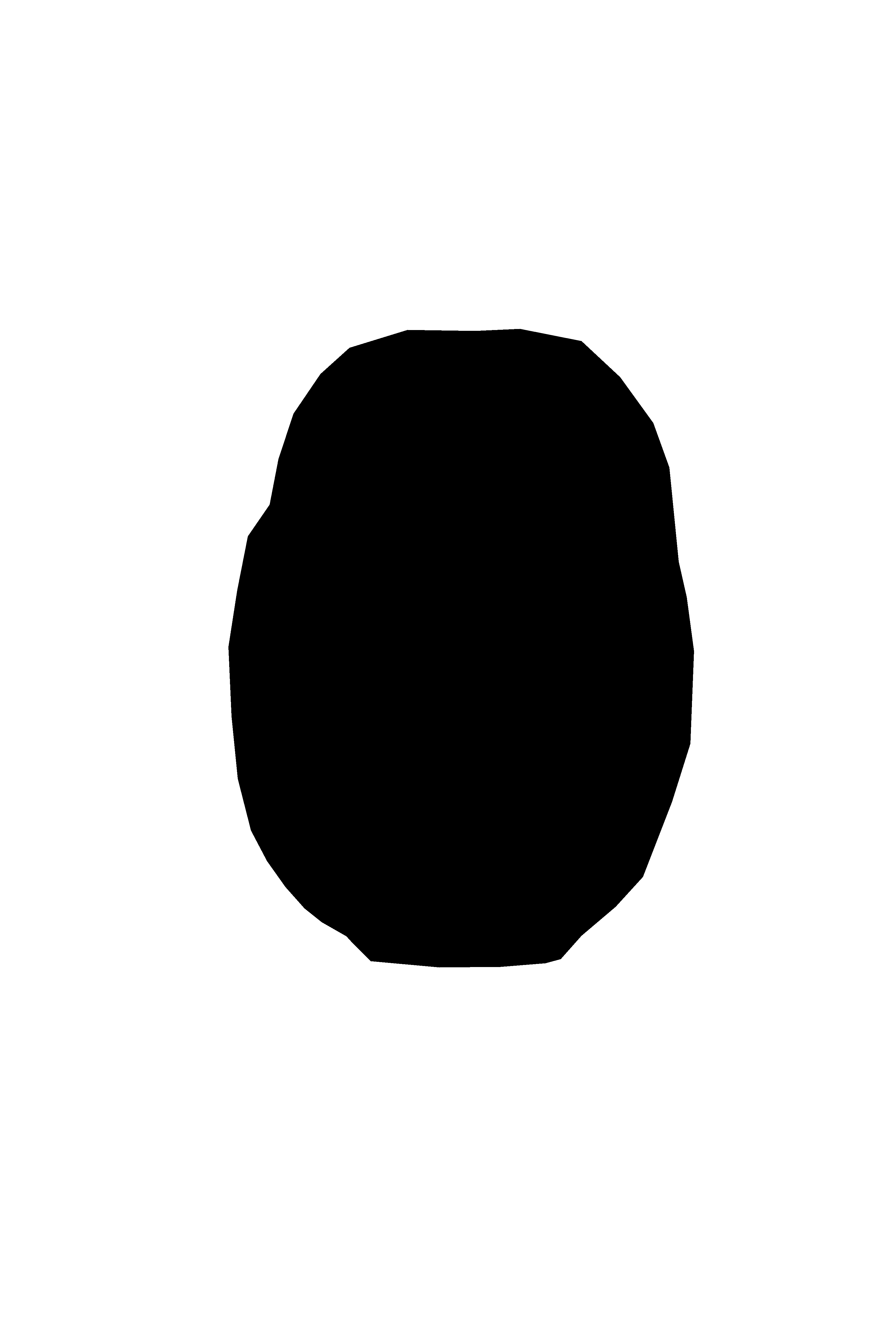

Supplement: Supplementary file 1 [file Data_Sheet_1.zip › face/060_face_mask.png]

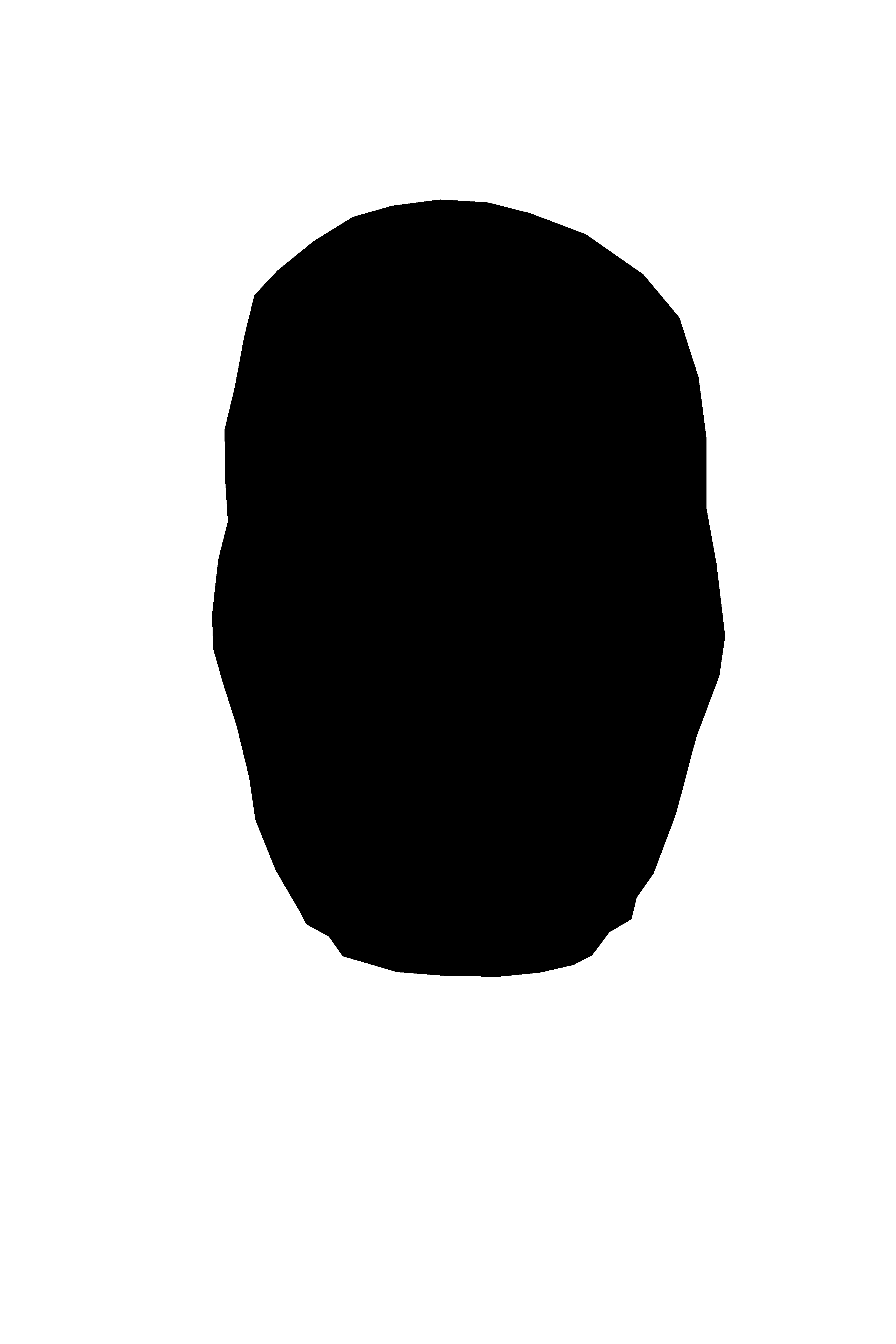

Supplement: Supplementary file 1 [file Data_Sheet_1.zip › face/061_face_mask.png]

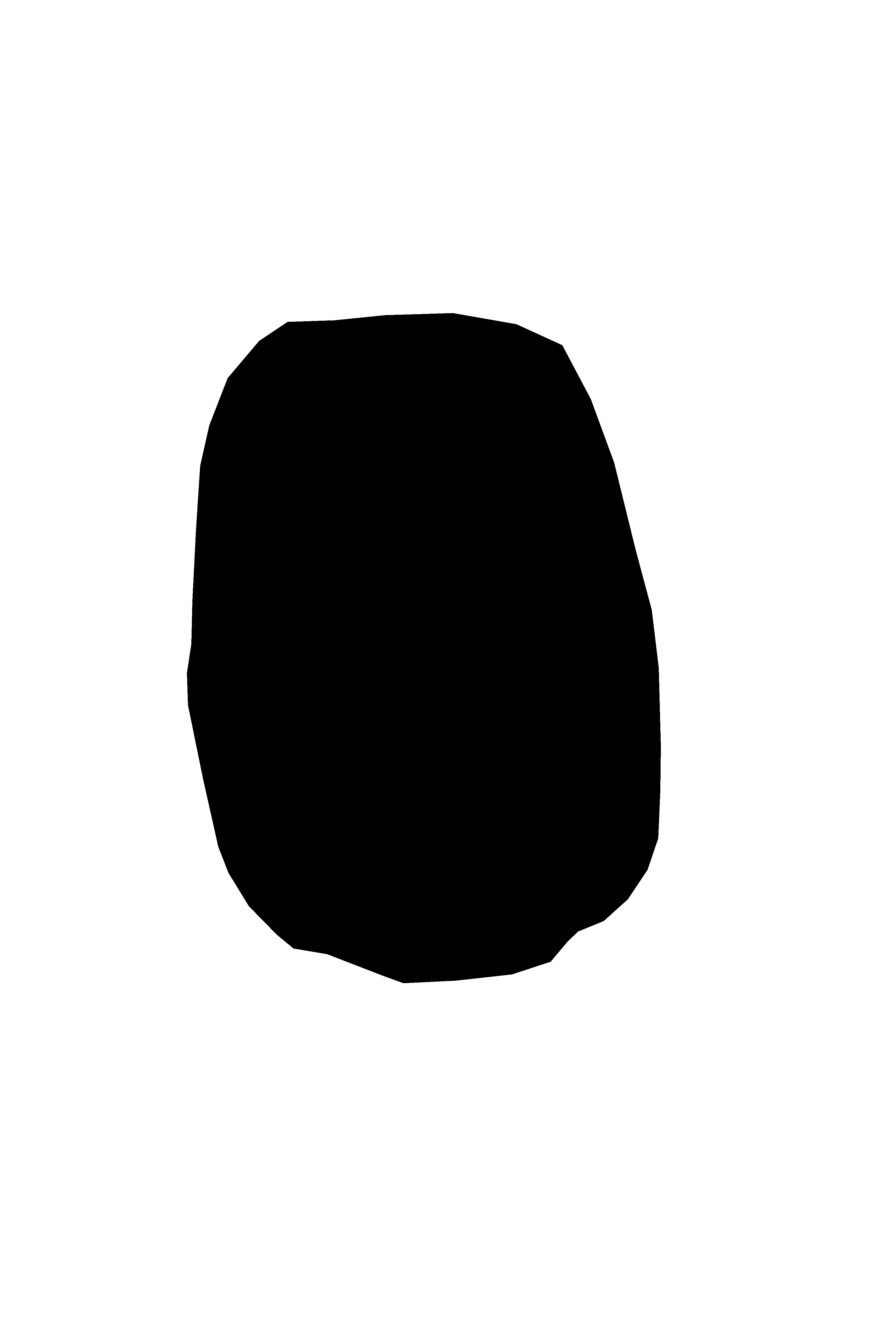

Supplement: Supplementary file 1 [file Data_Sheet_1.zip › face/062_face_mask.png]

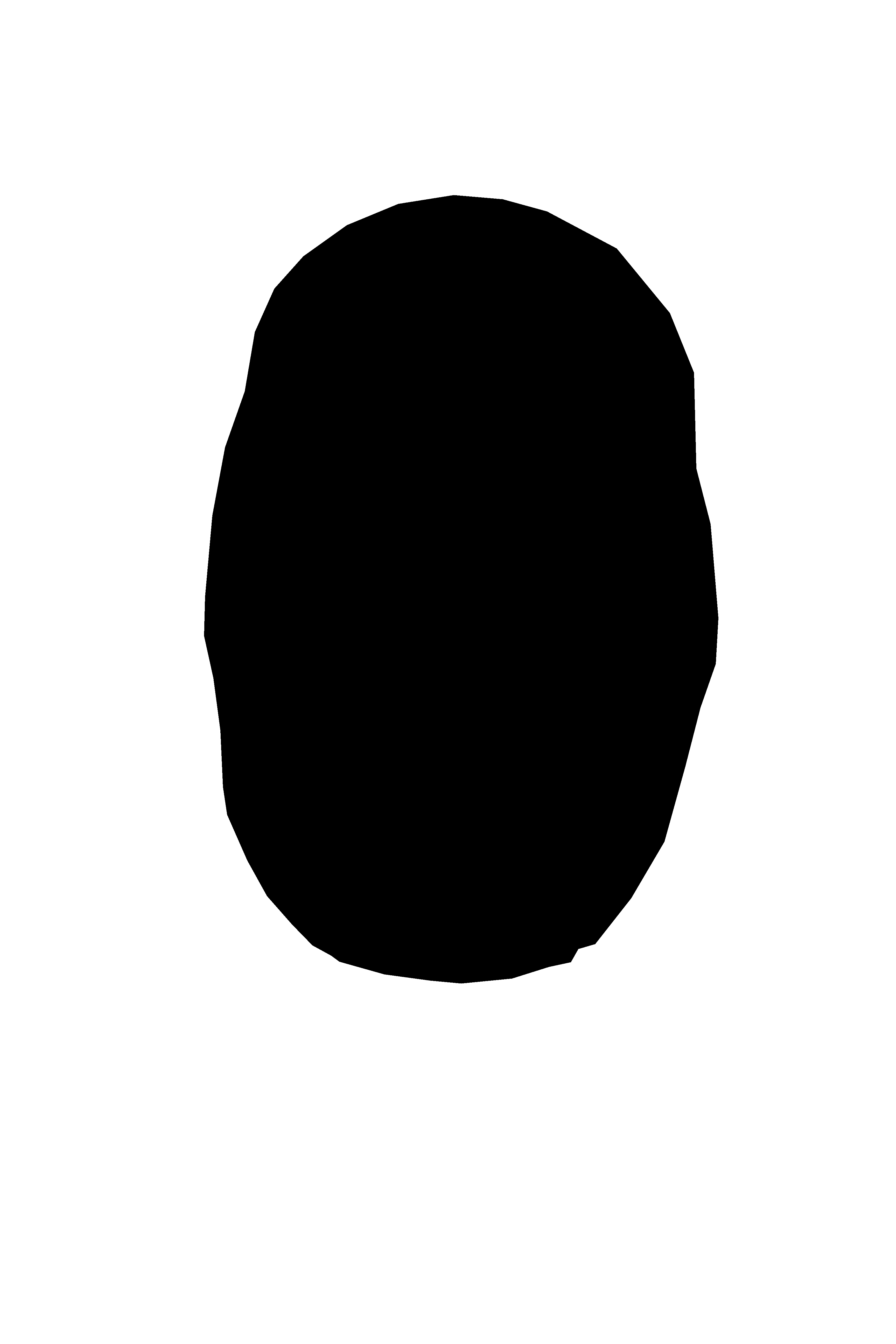

Supplement: Supplementary file 1 [file Data_Sheet_1.zip › face/063_face_mask.png]

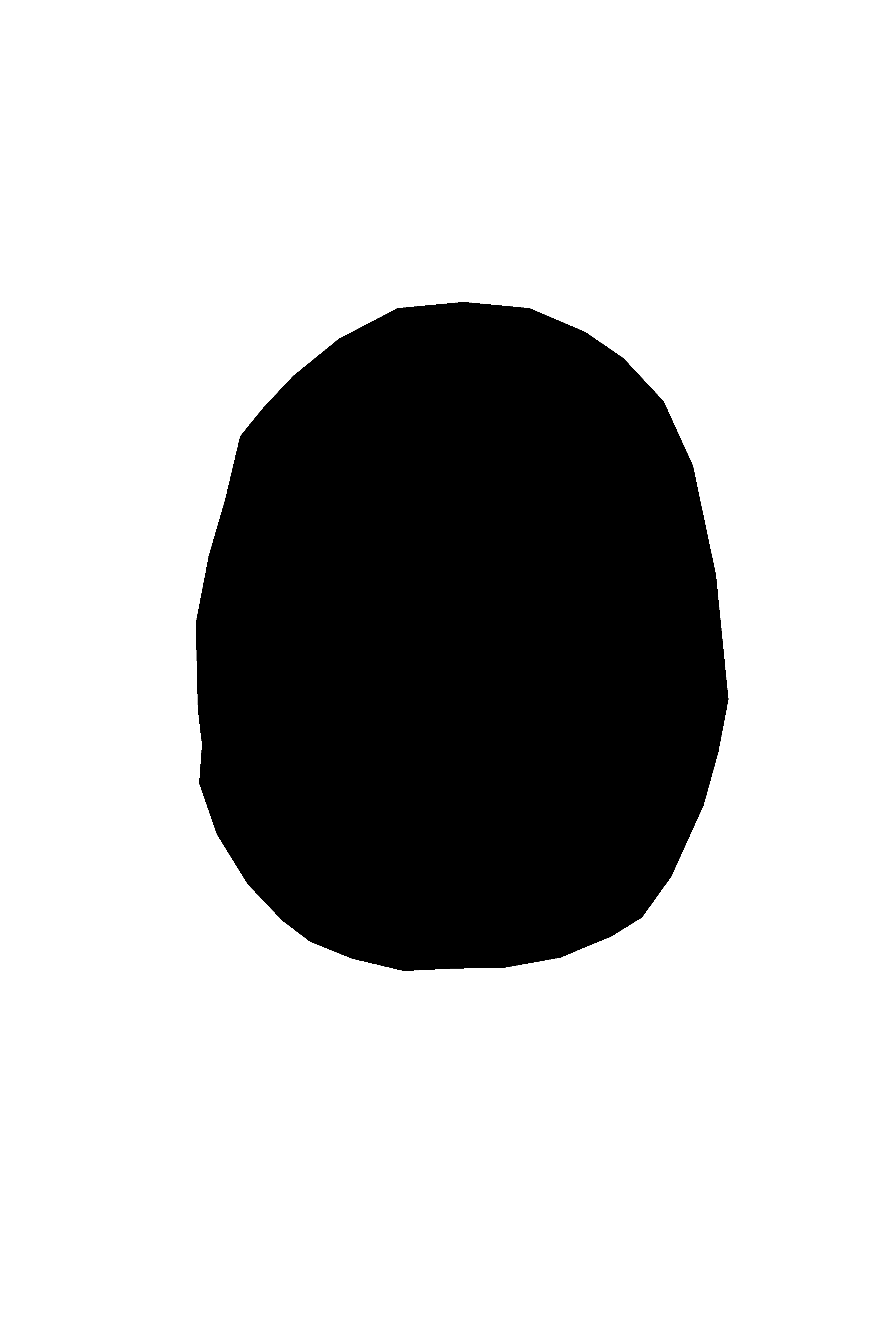

Supplement: Supplementary file 1 [file Data_Sheet_1.zip › face/064_face_mask.png]

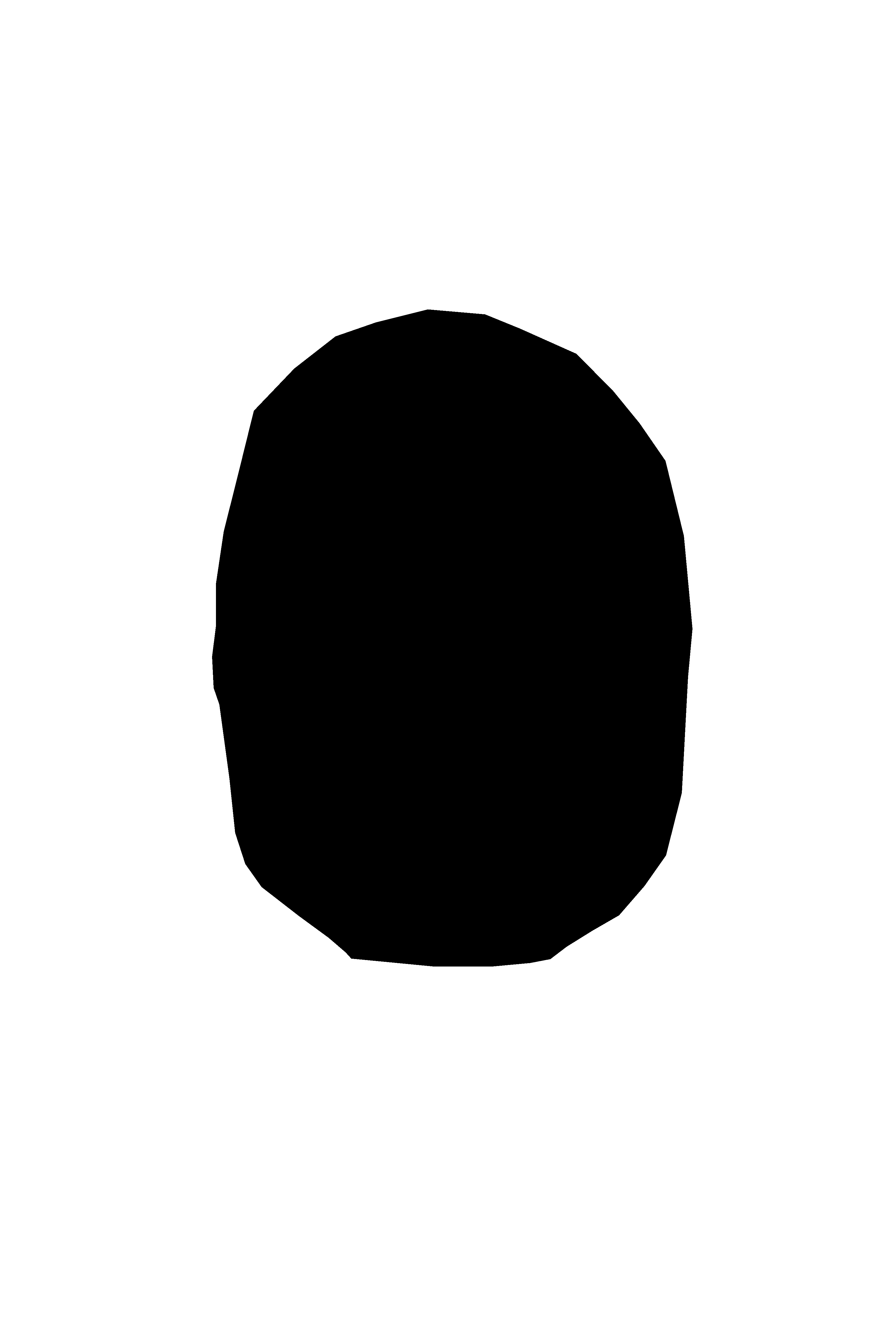

Supplement: Supplementary file 1 [file Data_Sheet_1.zip › face/065_face_mask.png]

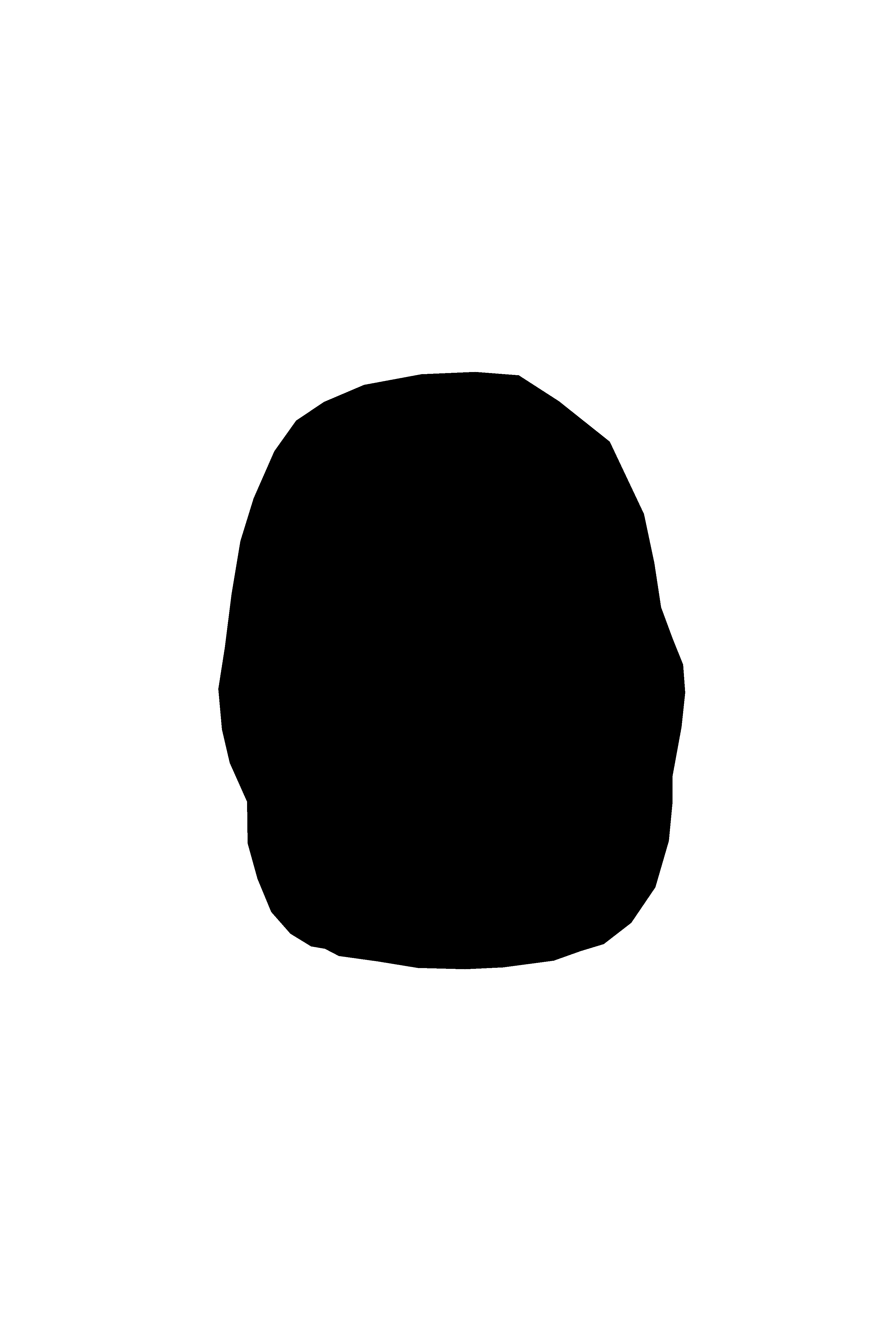

Supplement: Supplementary file 1 [file Data_Sheet_1.zip › face/066_face_mask.png]

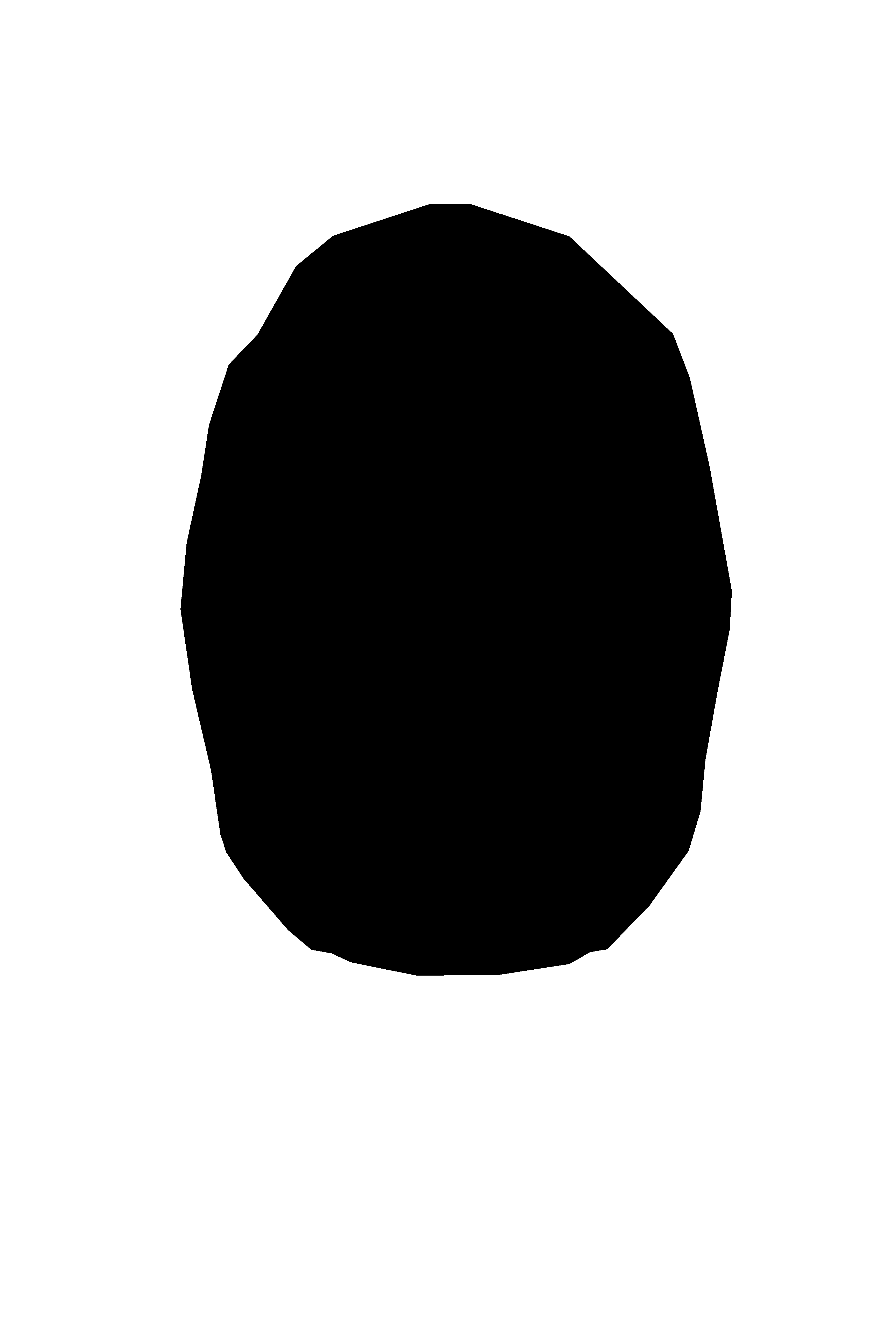

Supplement: Supplementary file 1 [file Data_Sheet_1.zip › face/067_face_mask.png]

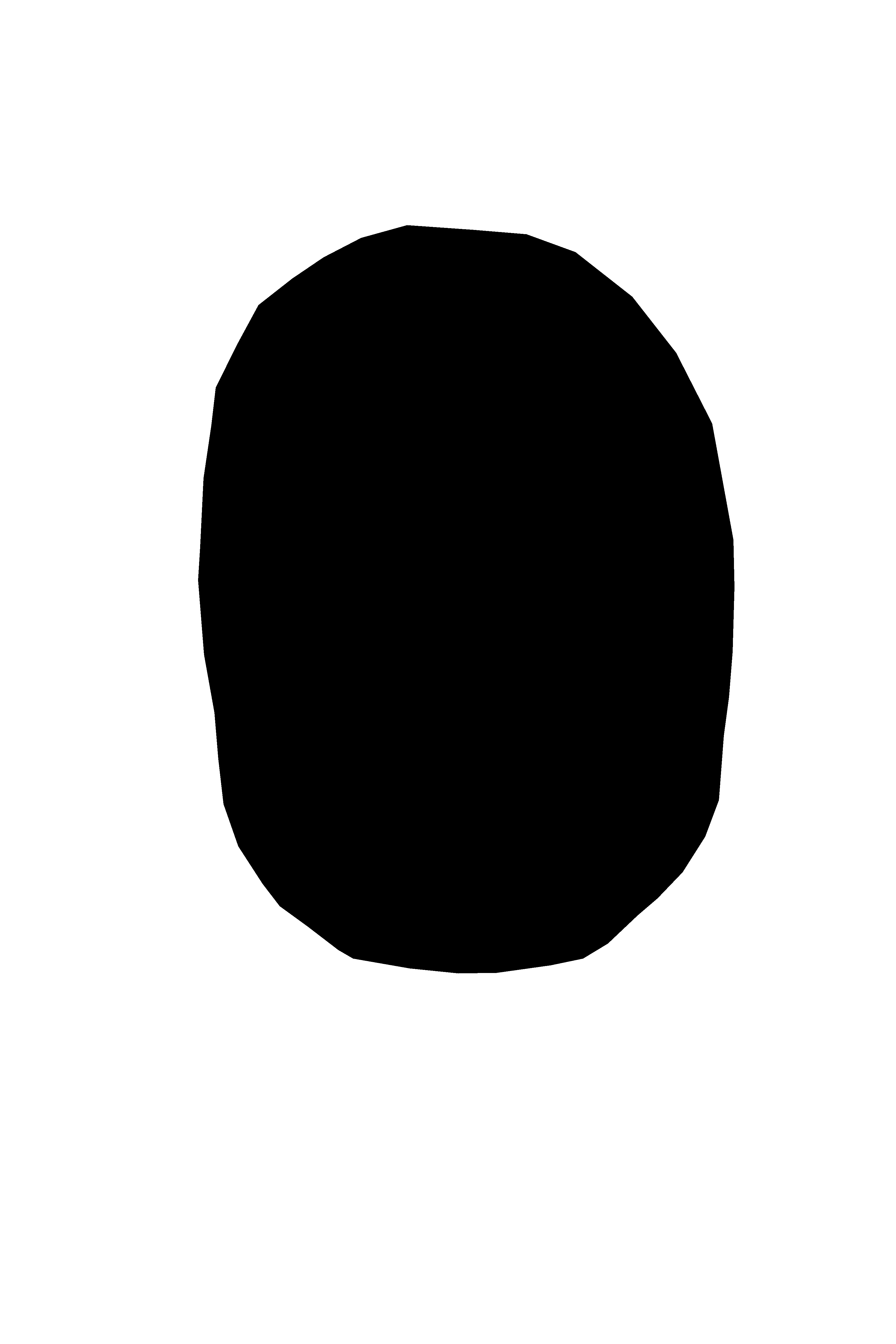

Supplement: Supplementary file 1 [file Data_Sheet_1.zip › face/068_face_mask.png]

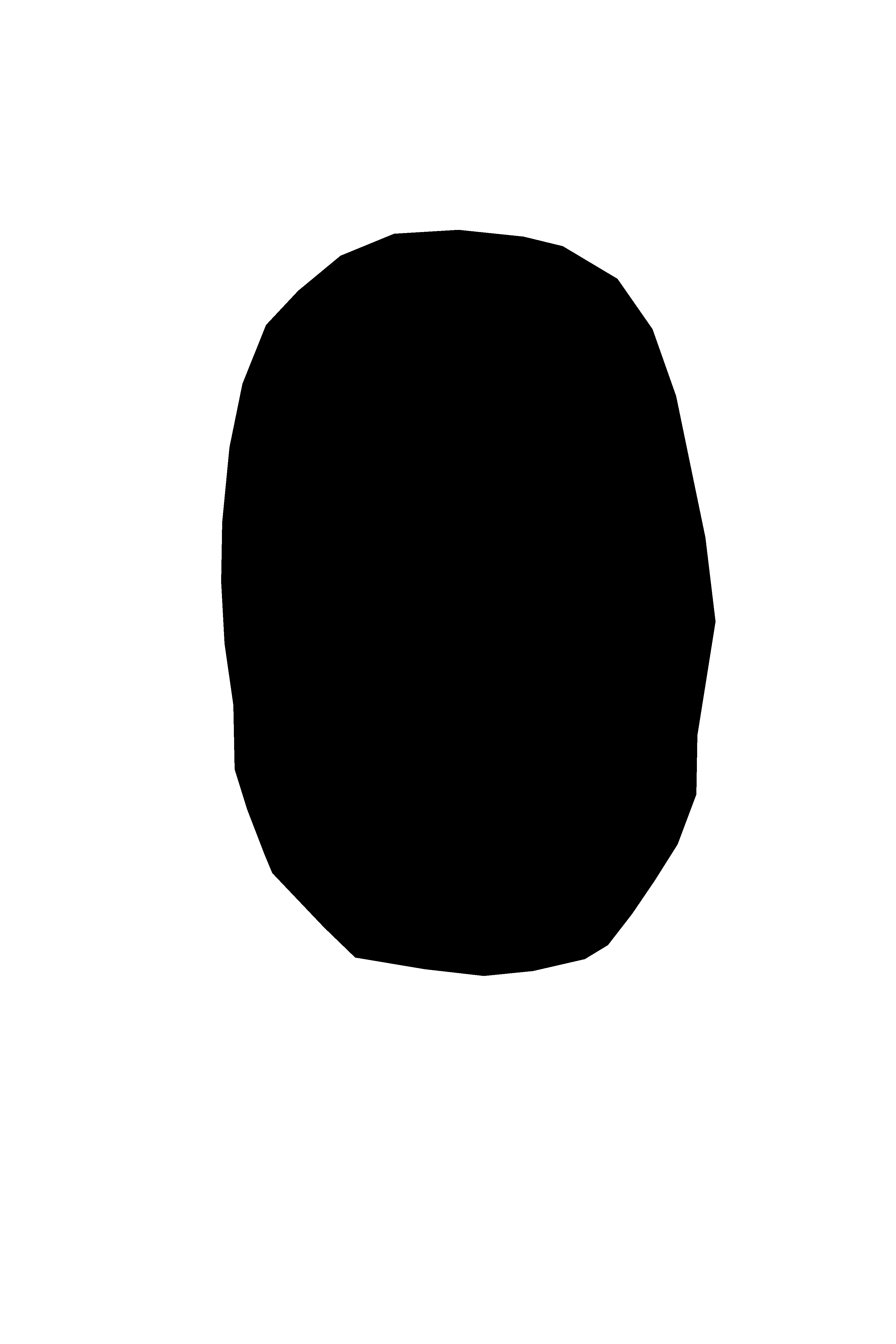

Supplement: Supplementary file 1 [file Data_Sheet_1.zip › face/069_face_mask.png]

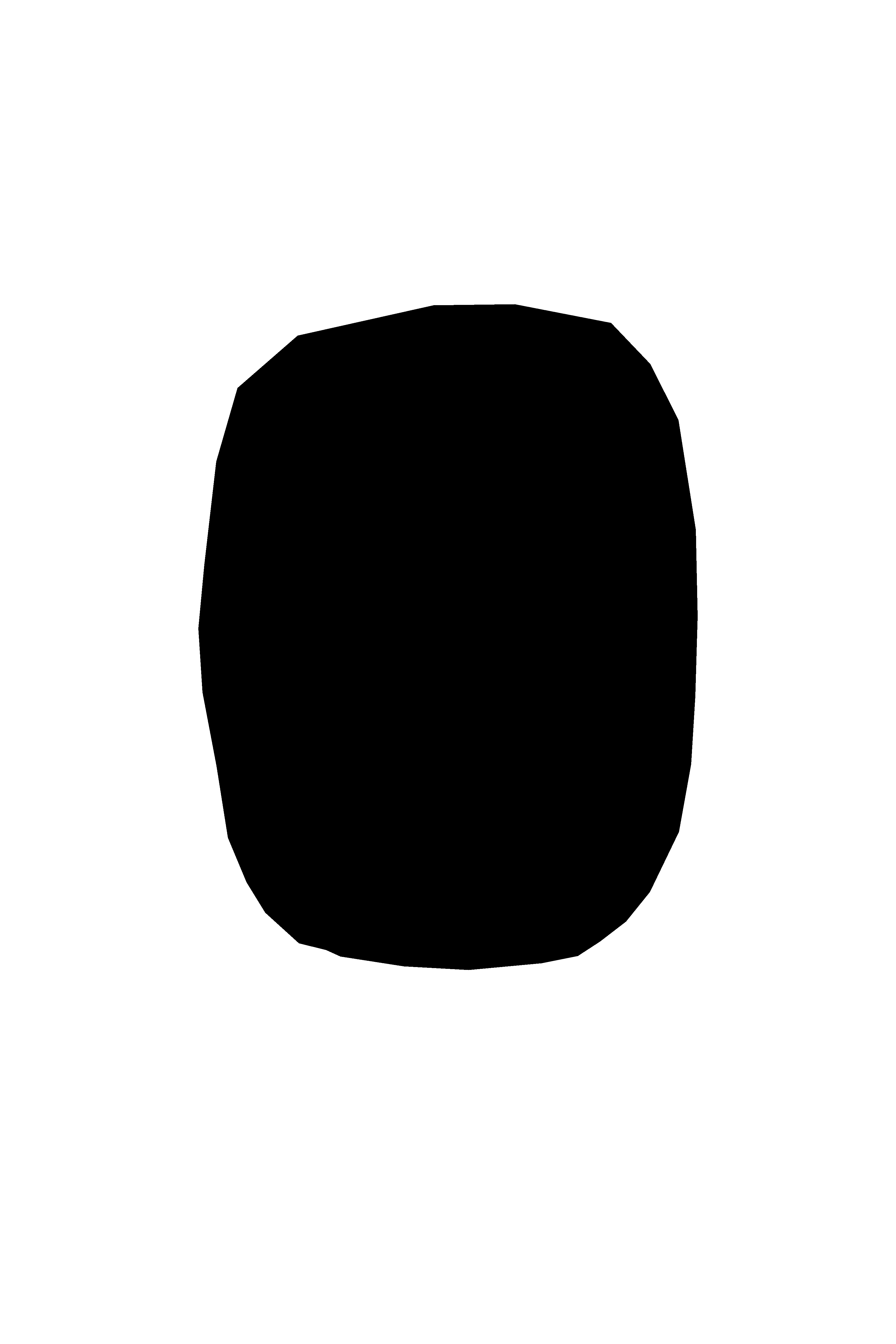

Supplement: Supplementary file 1 [file Data_Sheet_1.zip › face/070_face_mask.png]

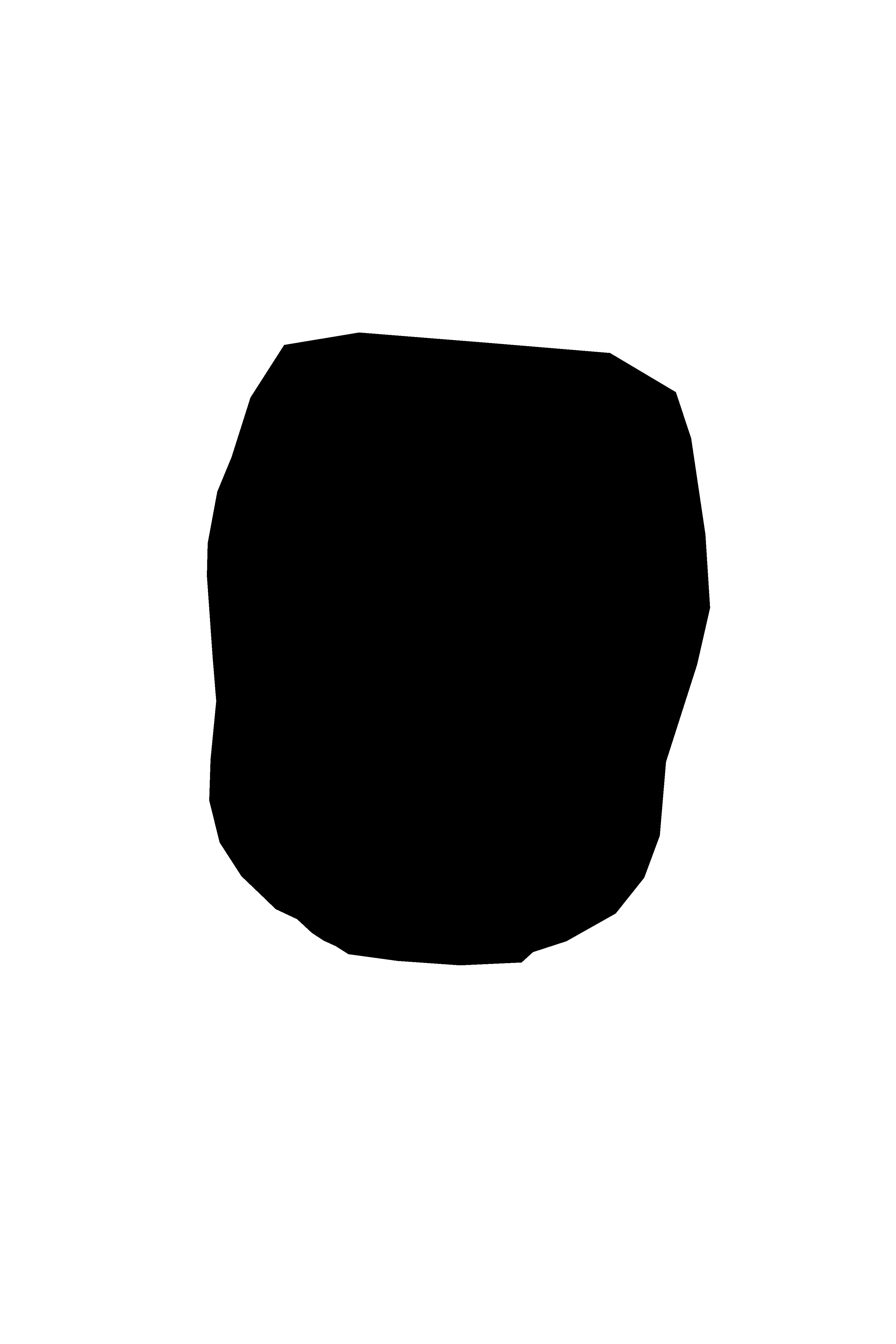

Supplement: Supplementary file 1 [file Data_Sheet_1.zip › face/071_face_mask.png]

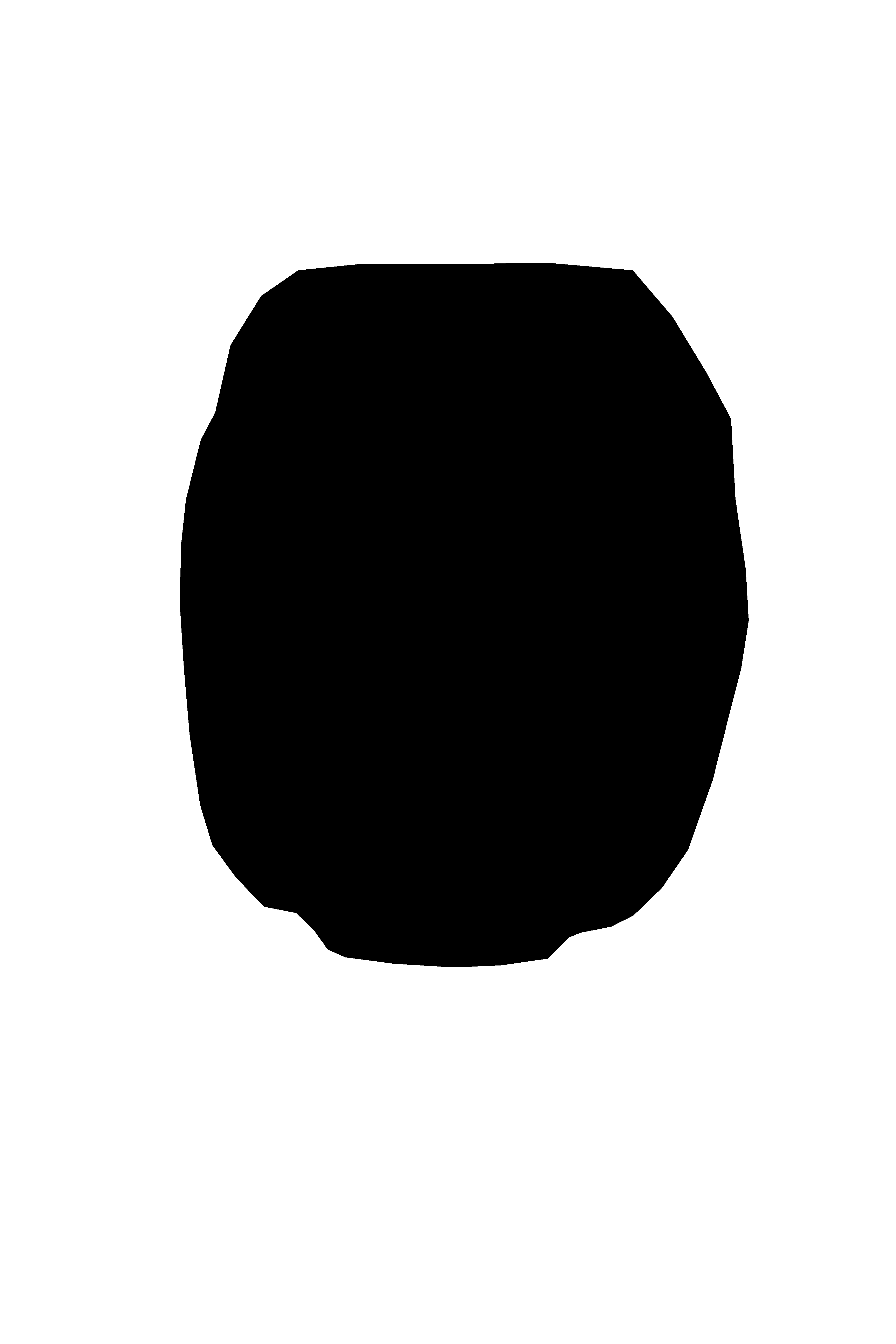

Supplement: Supplementary file 1 [file Data_Sheet_1.zip › face/072_face_mask.png]

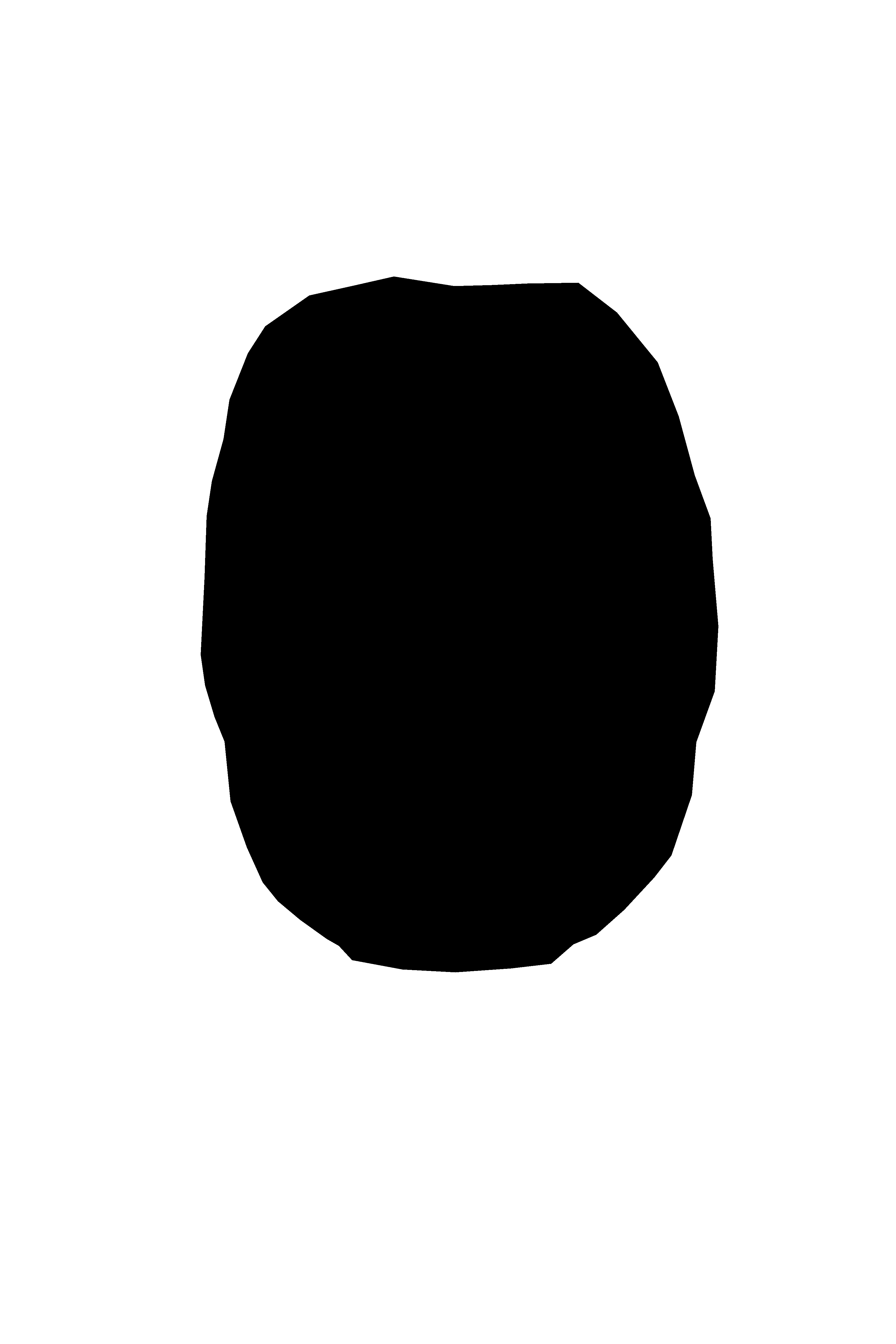

Supplement: Supplementary file 1 [file Data_Sheet_1.zip › face/073_face_mask.png]

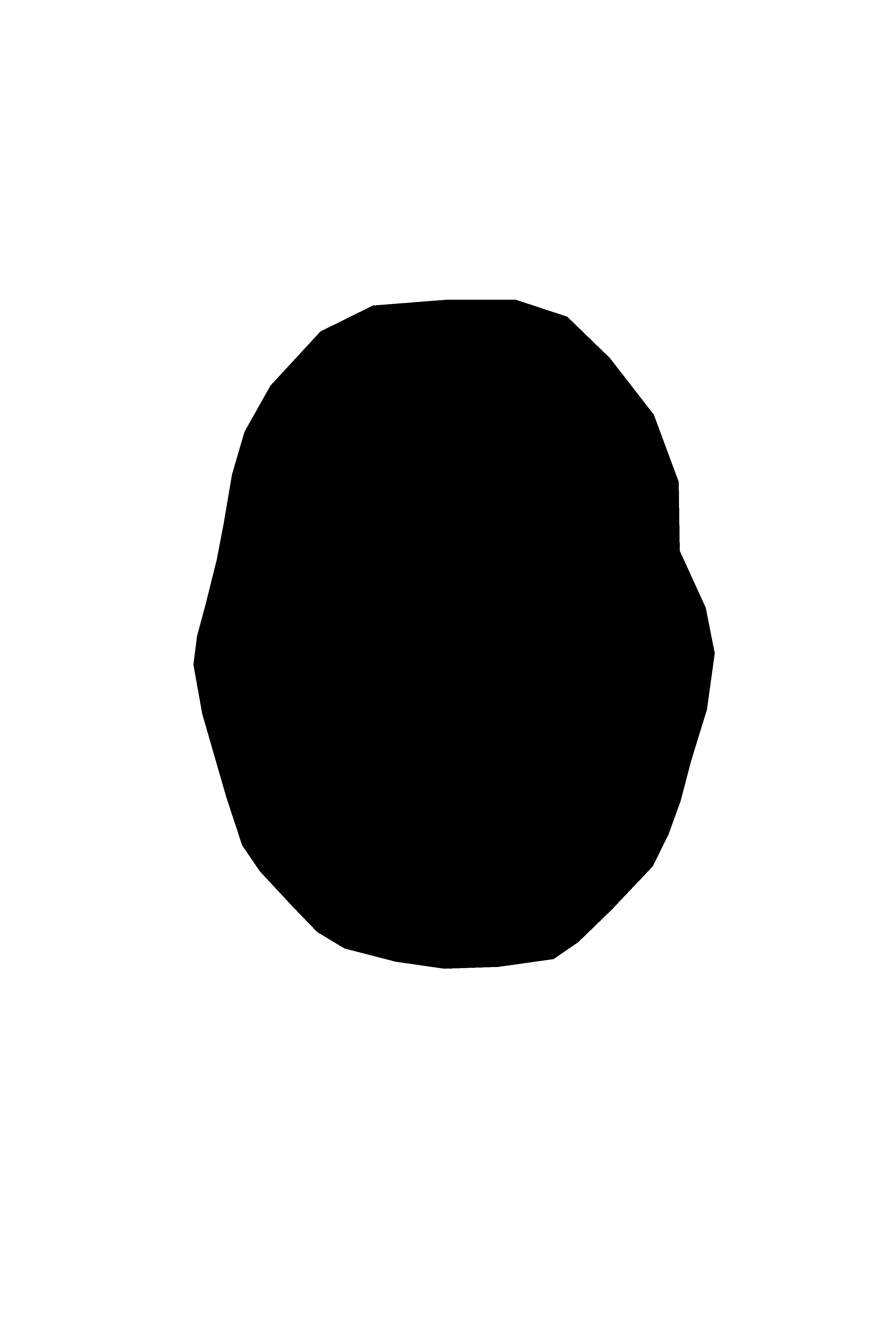

Supplement: Supplementary file 1 [file Data_Sheet_1.zip › face/074_face_mask.png]

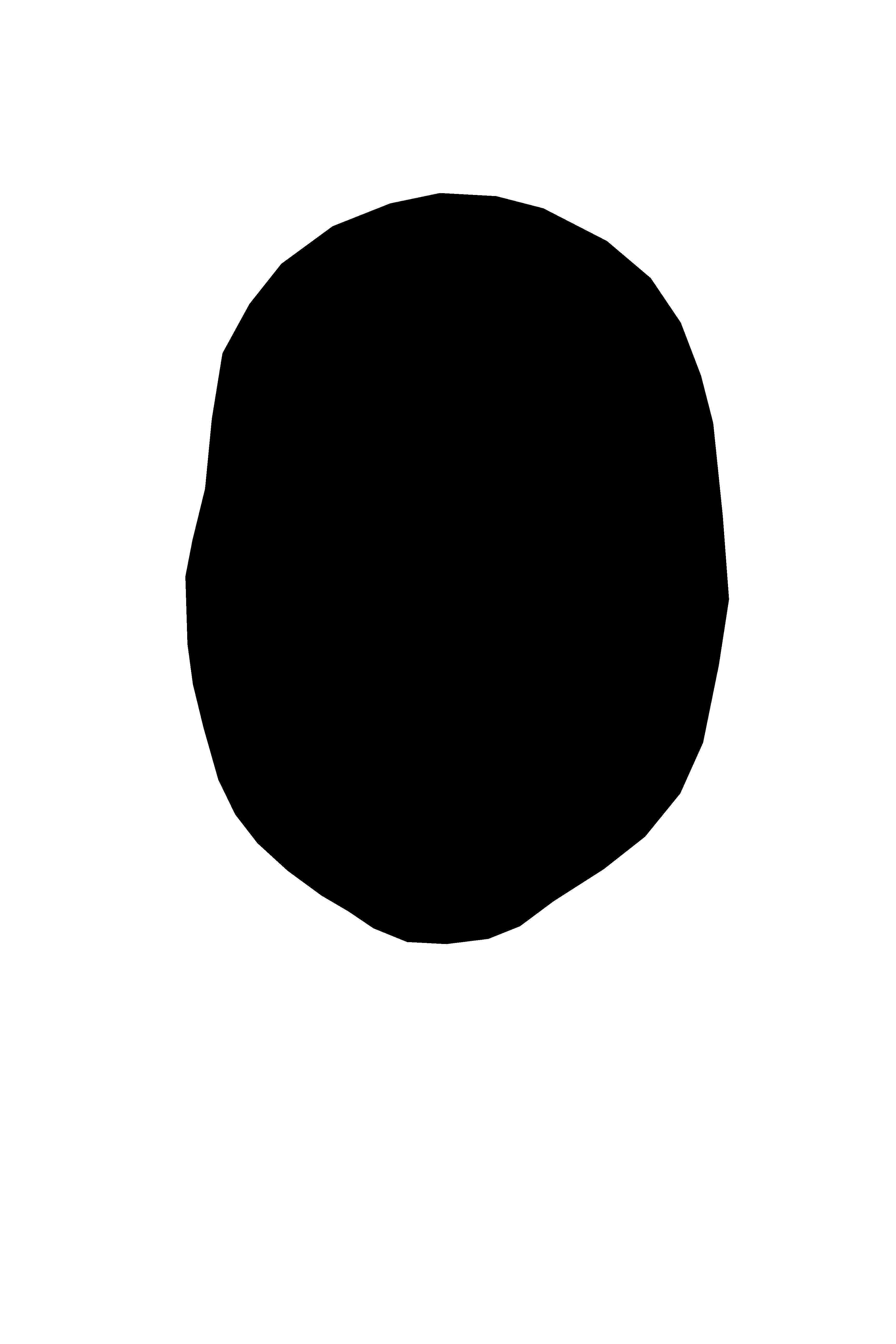

Supplement: Supplementary file 1 [file Data_Sheet_1.zip › face/075_face_mask.png]

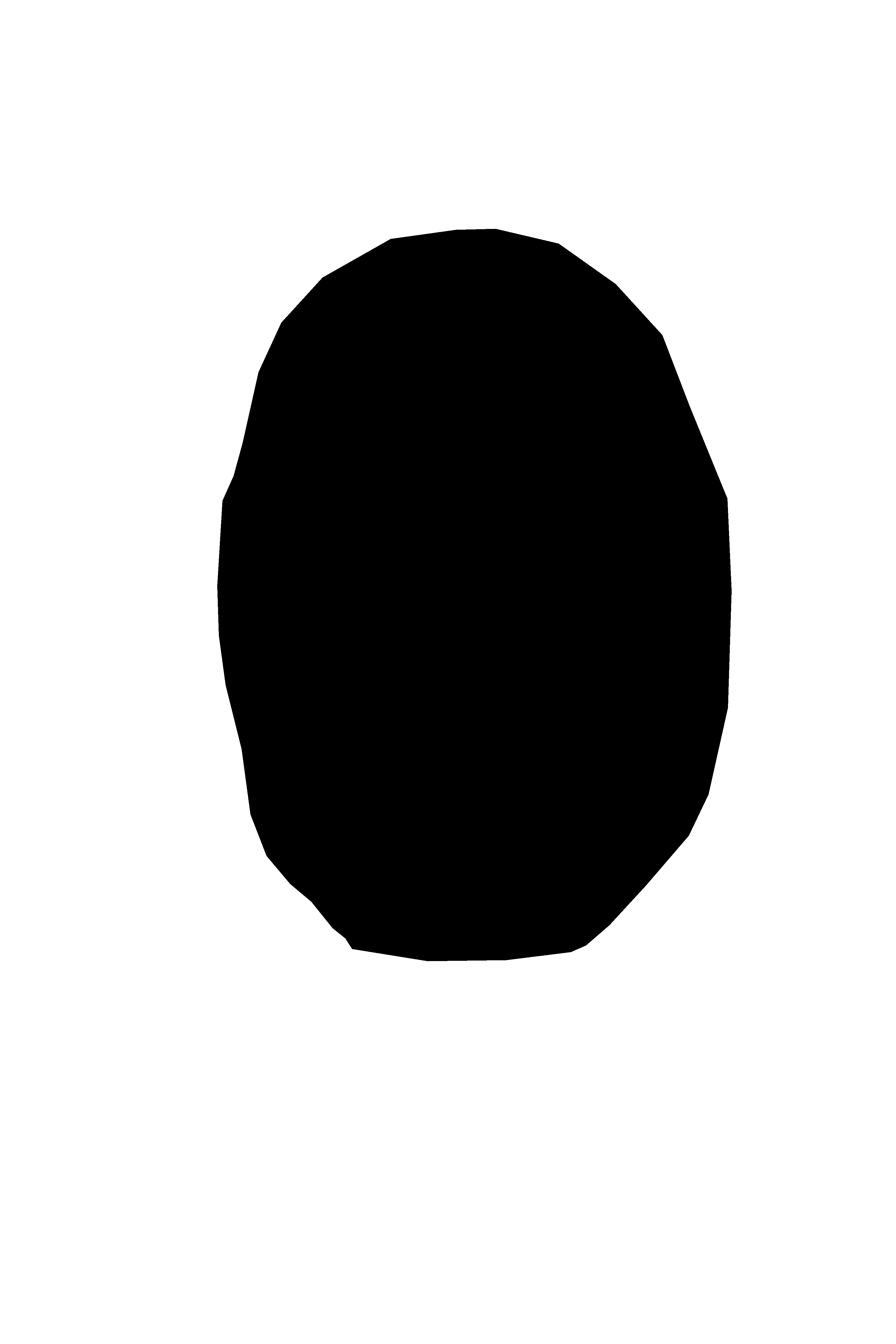

Supplement: Supplementary file 1 [file Data_Sheet_1.zip › face/076_face_mask.png]

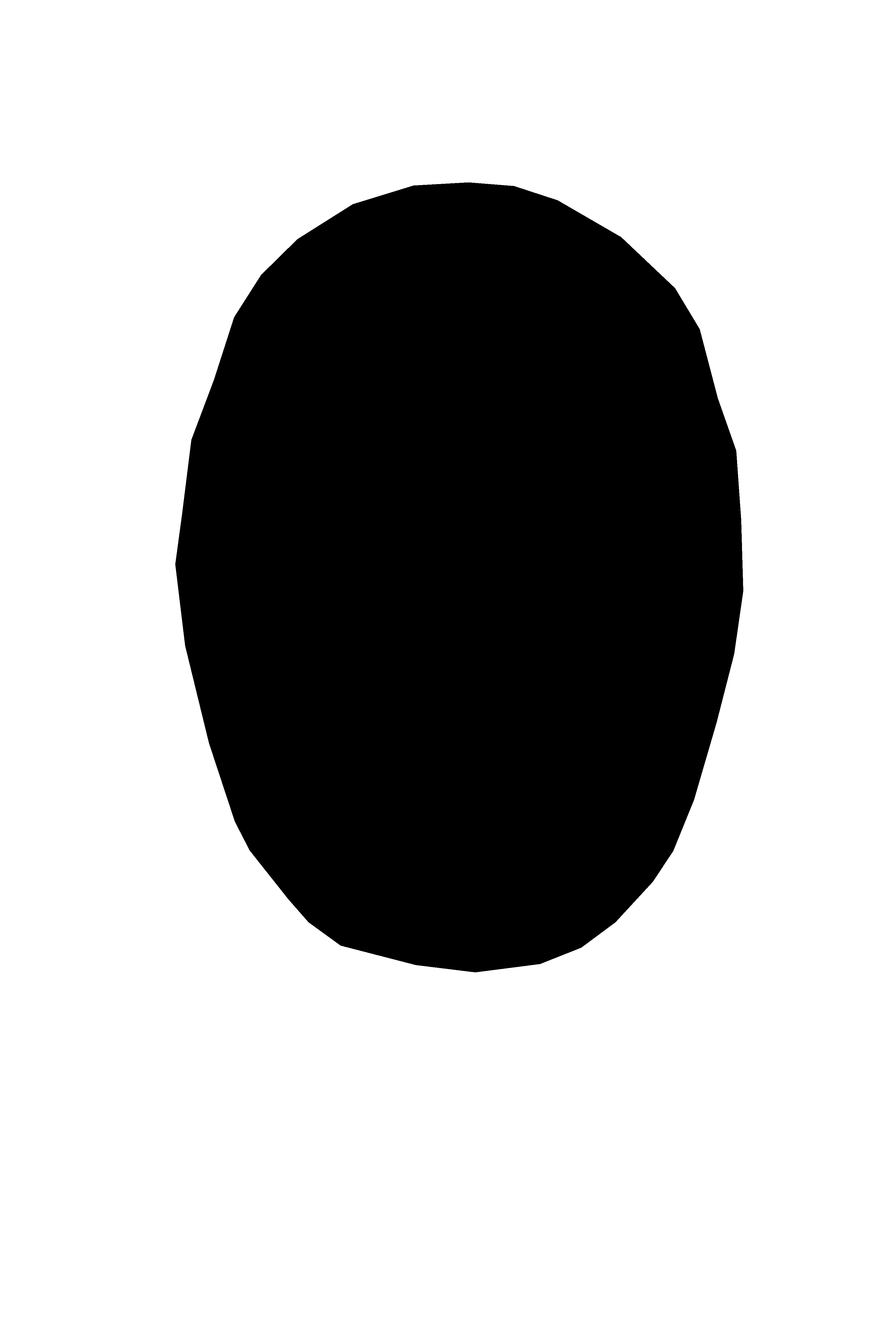

Supplement: Supplementary file 1 [file Data_Sheet_1.zip › face/077_face_mask.png]

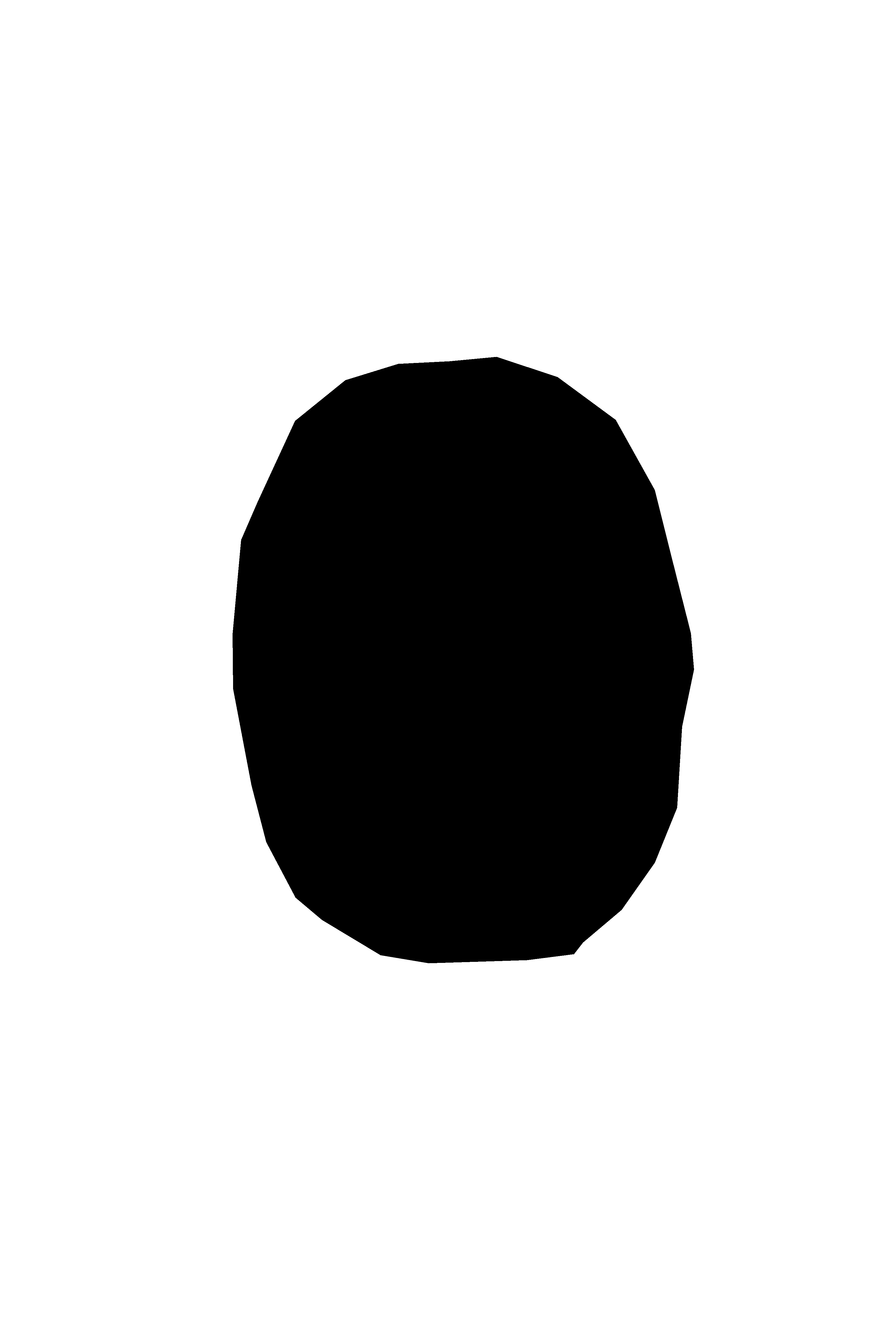

Supplement: Supplementary file 1 [file Data_Sheet_1.zip › face/078_face_mask.png]

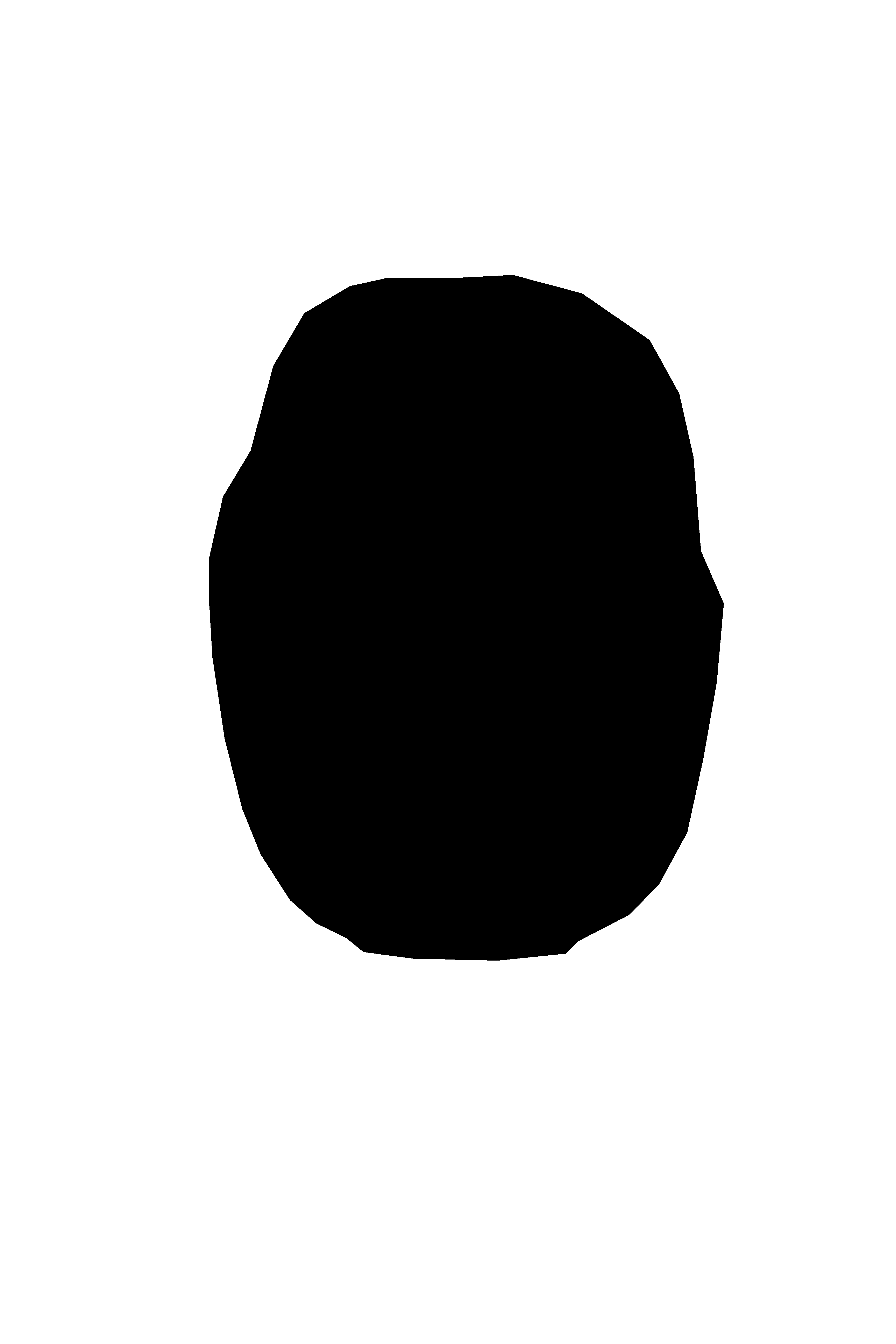

Supplement: Supplementary file 1 [file Data_Sheet_1.zip › face/079_face_mask.png]

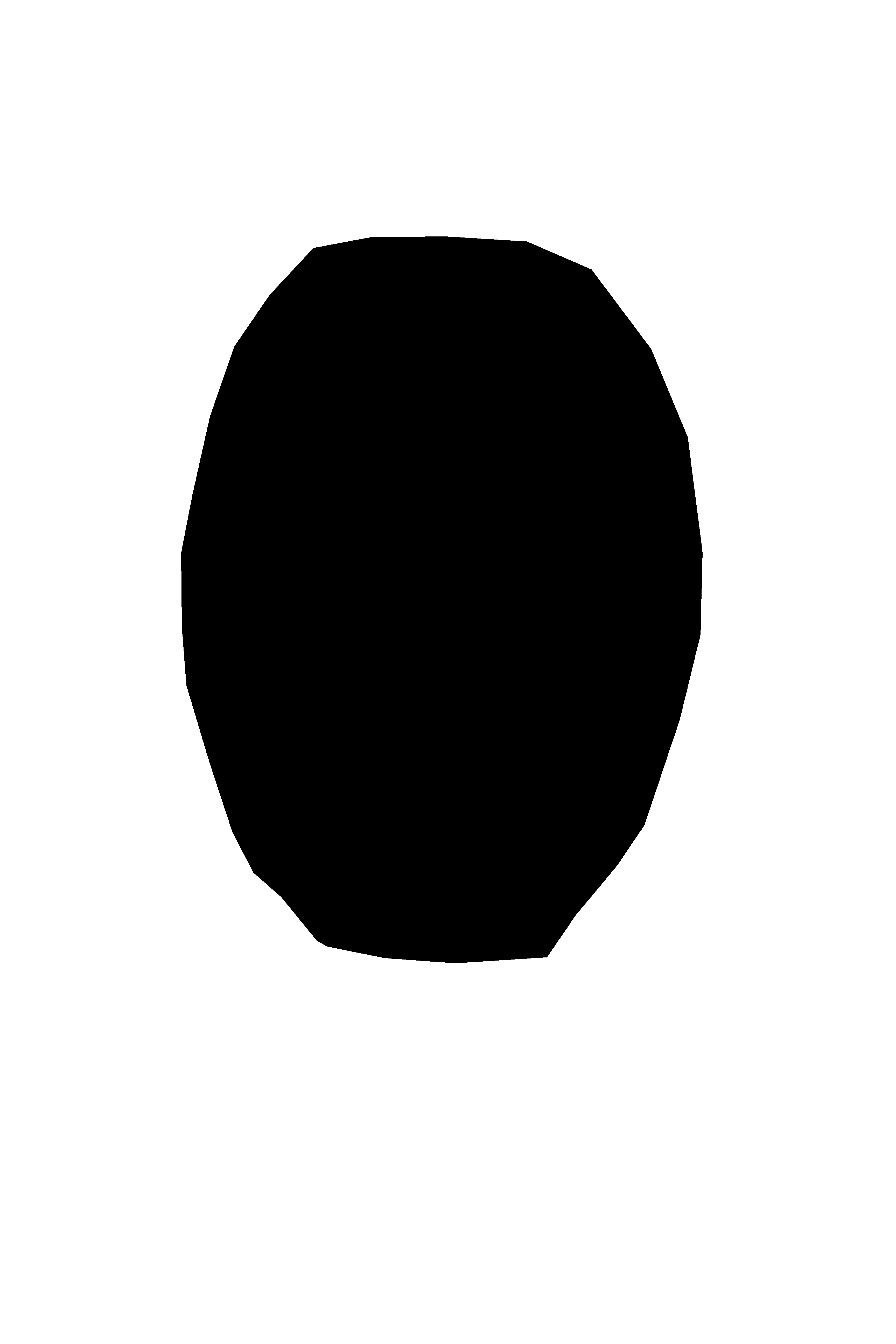

Supplement: Supplementary file 1 [file Data_Sheet_1.zip › face/080_face_mask.png]

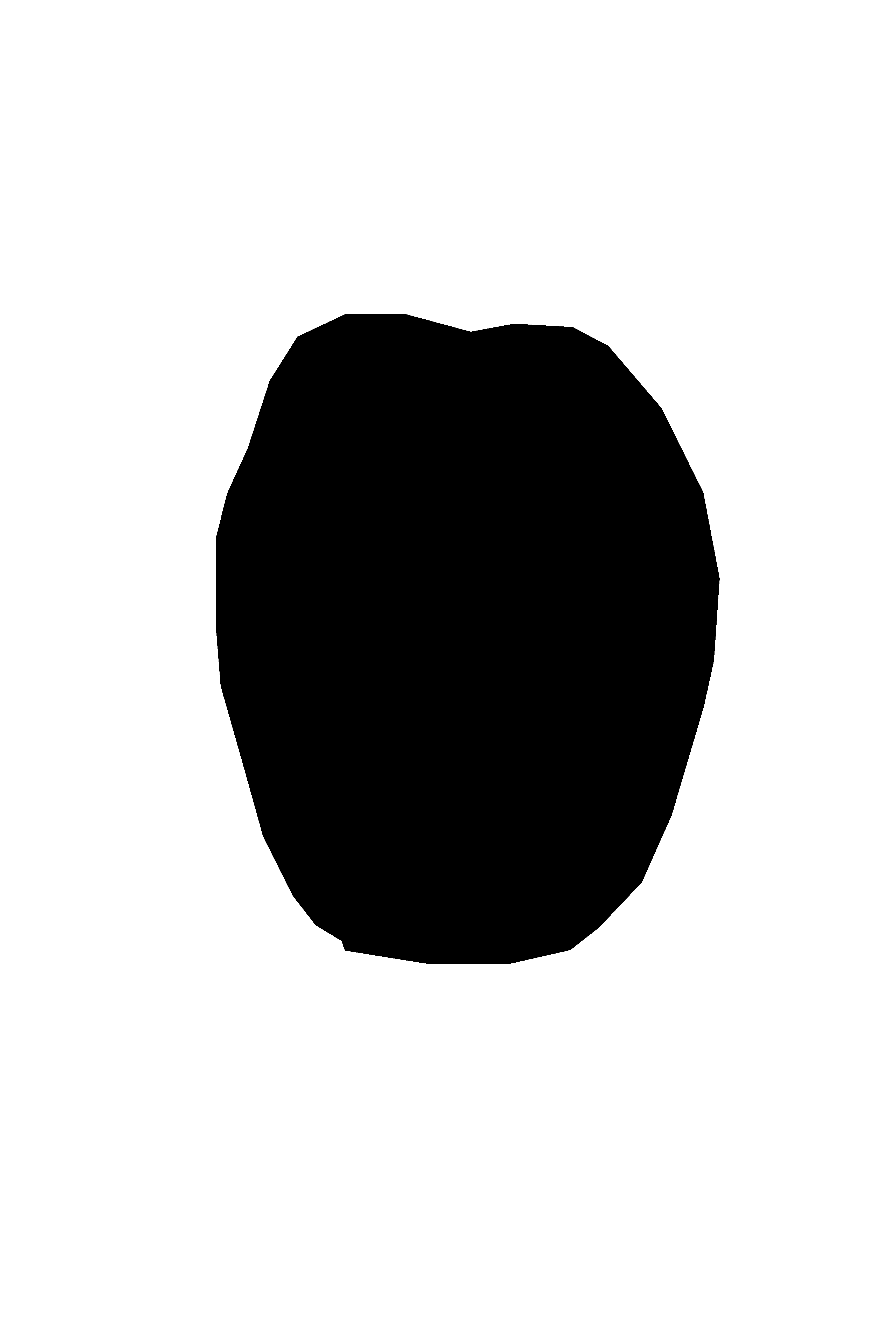

Supplement: Supplementary file 1 [file Data_Sheet_1.zip › face/081_face_mask.png]

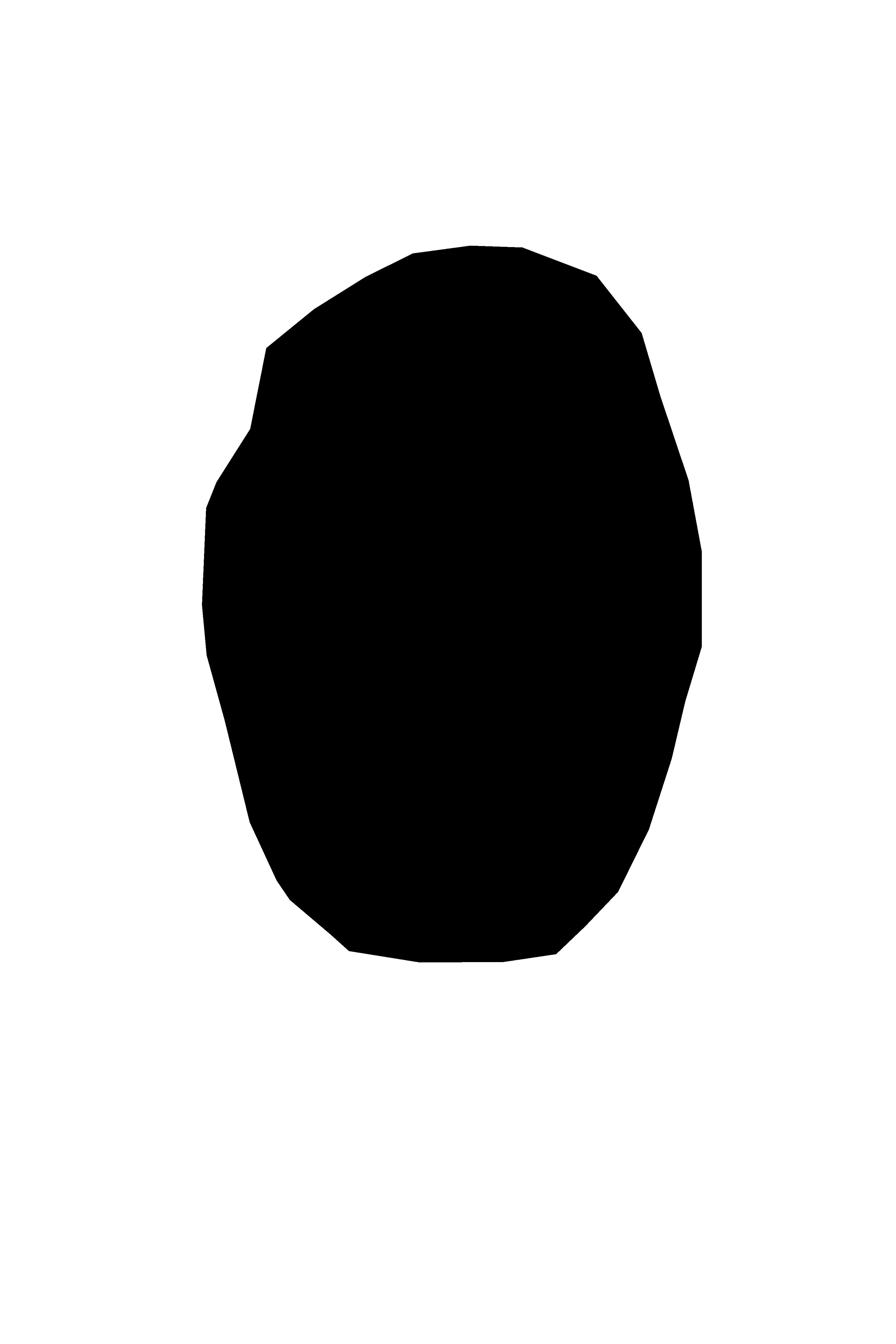

Supplement: Supplementary file 1 [file Data_Sheet_1.zip › face/082_face_mask.png]

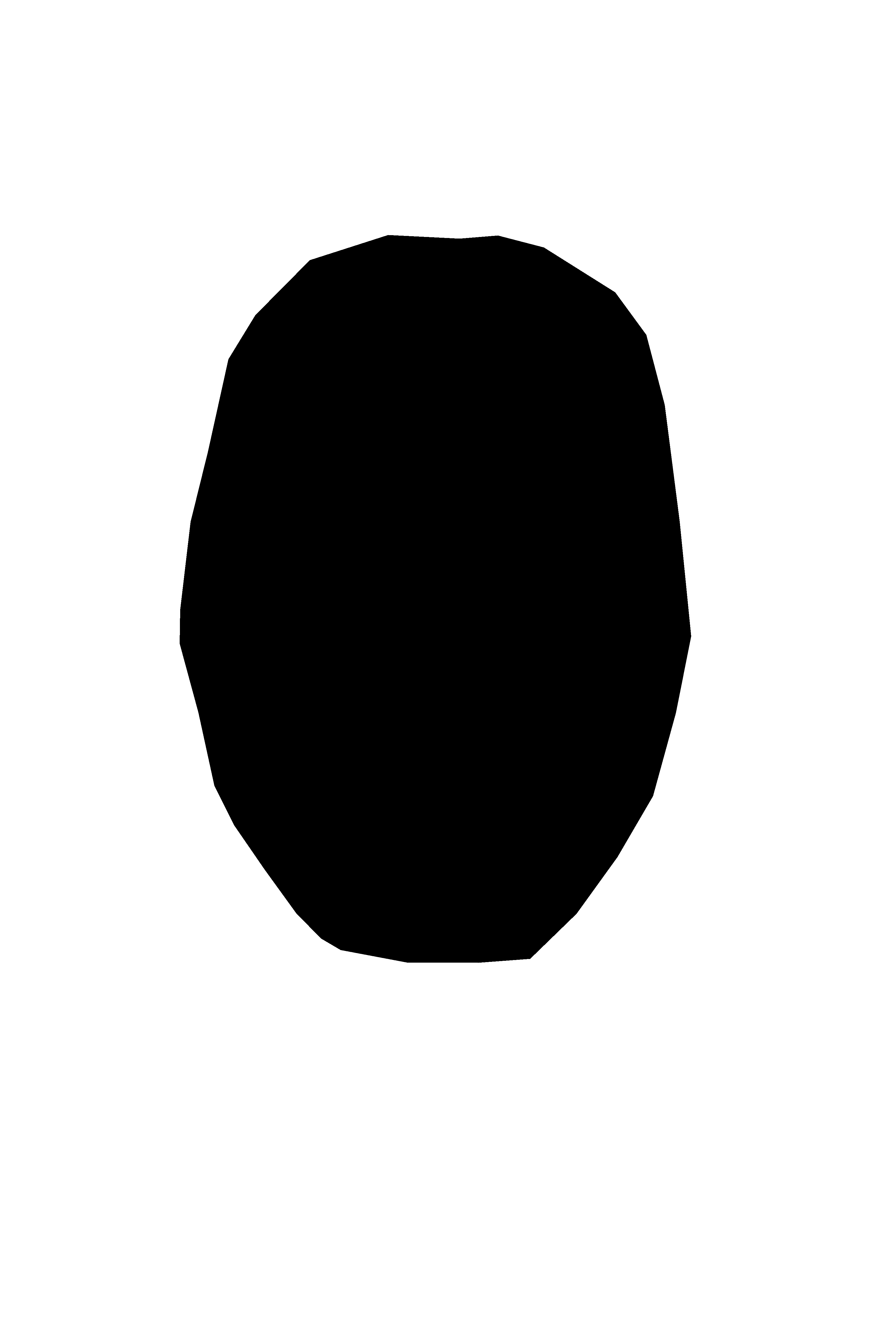

Supplement: Supplementary file 1 [file Data_Sheet_1.zip › face/083_face_mask.png]

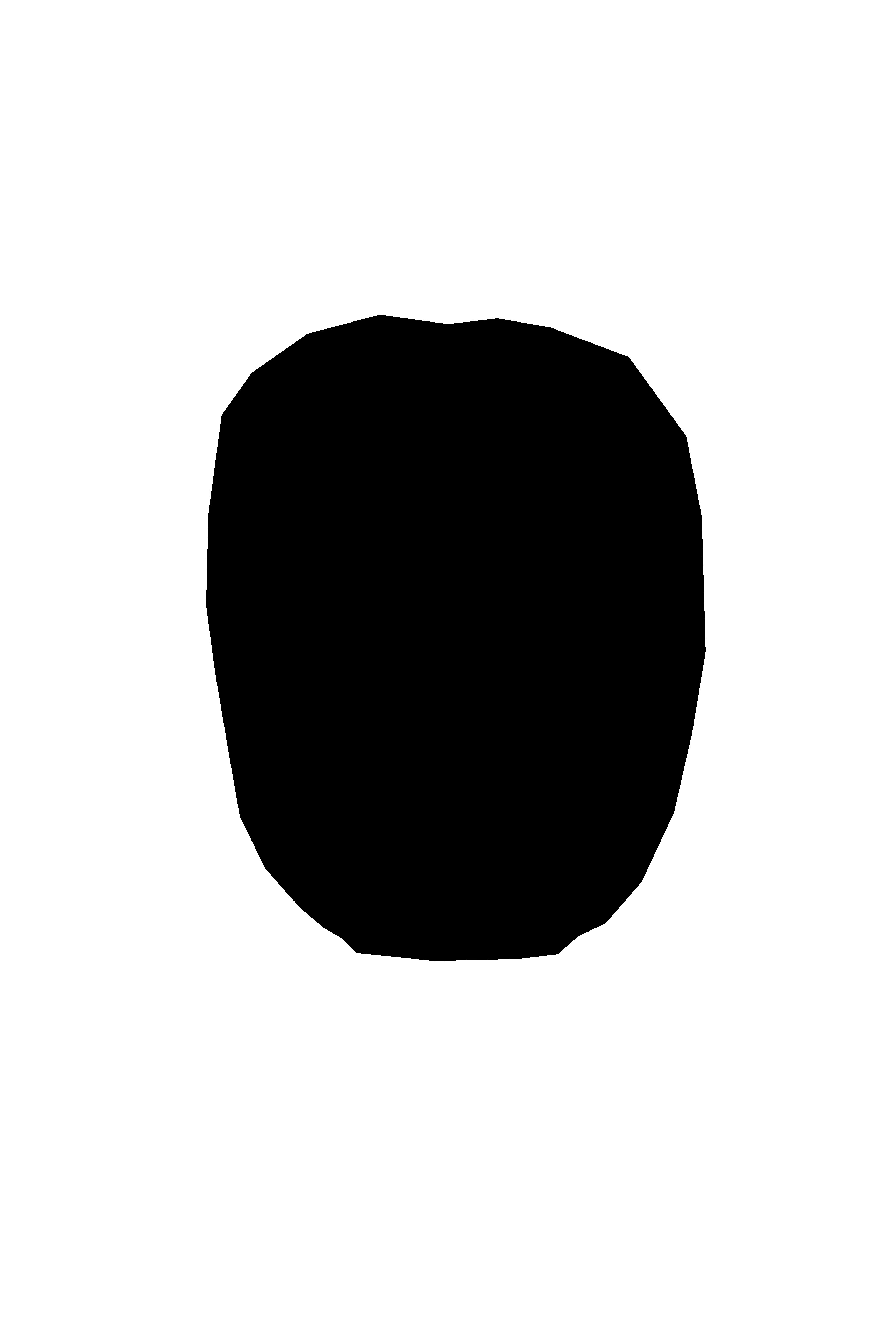

Supplement: Supplementary file 1 [file Data_Sheet_1.zip › face/084_face_mask.png]

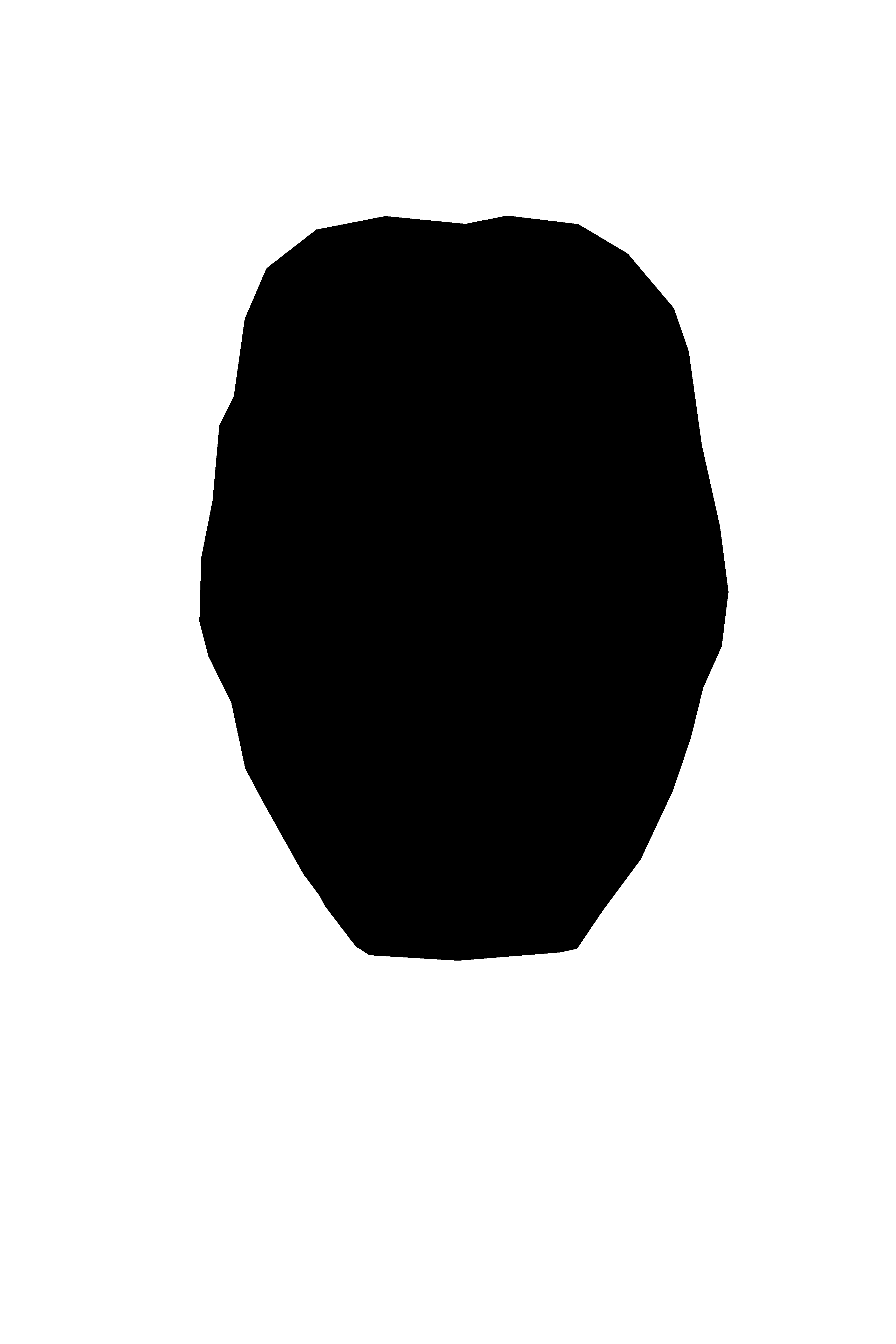

Supplement: Supplementary file 1 [file Data_Sheet_1.zip › face/085_face_mask.png]

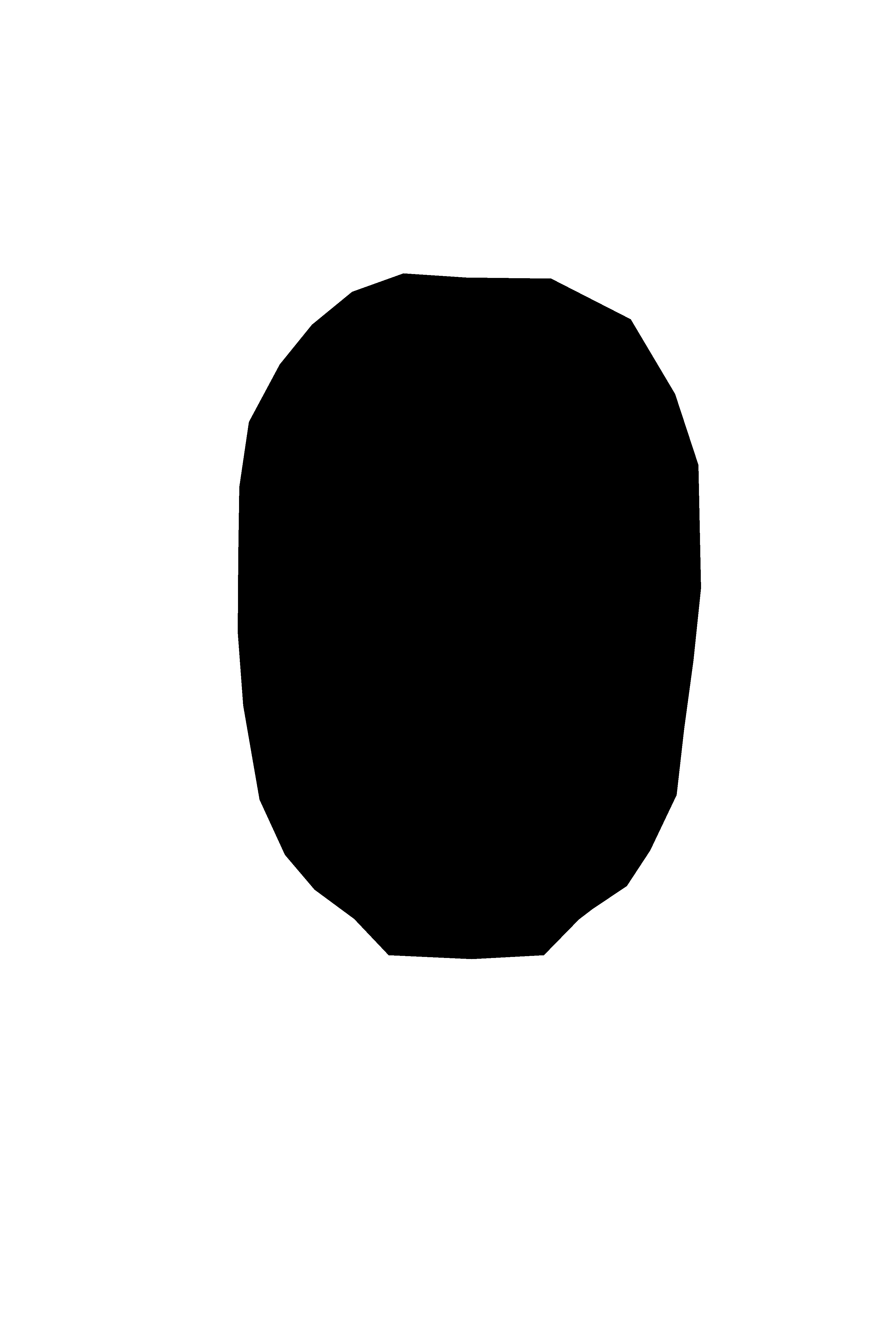

Supplement: Supplementary file 1 [file Data_Sheet_1.zip › face/086_face_mask.png]

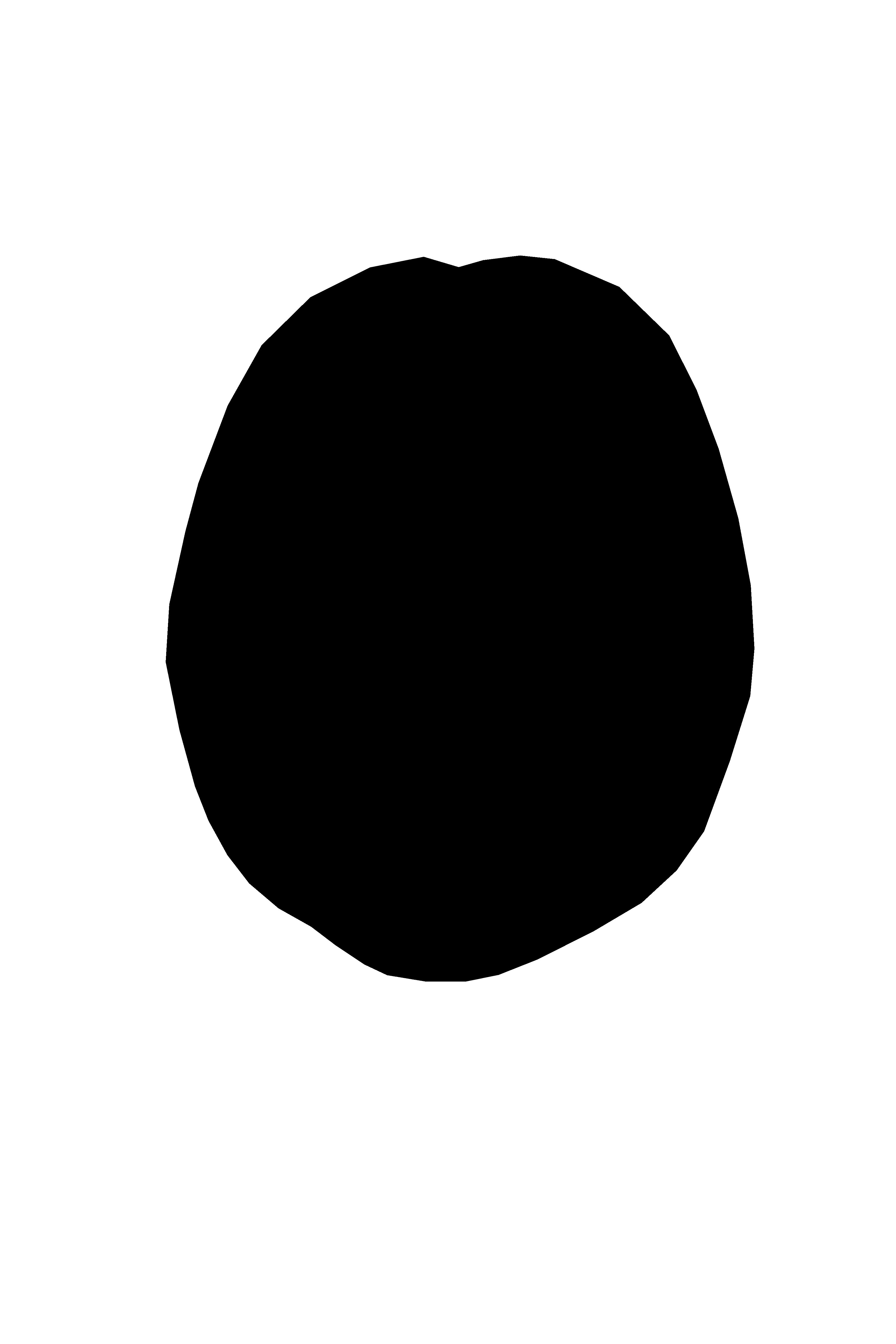

Supplement: Supplementary file 1 [file Data_Sheet_1.zip › face/087_face_mask.png]

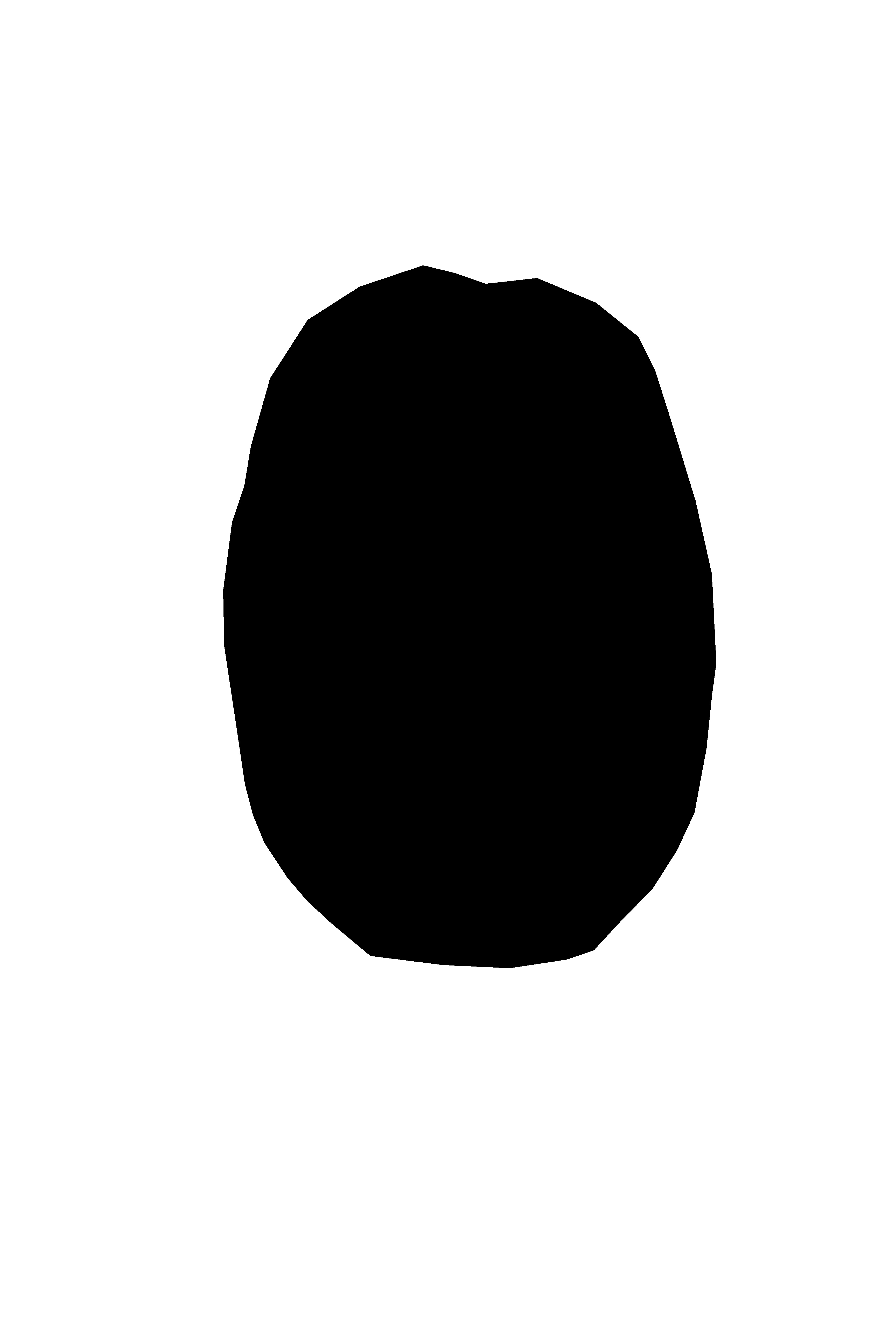

Supplement: Supplementary file 1 [file Data_Sheet_1.zip › face/088_face_mask.png]

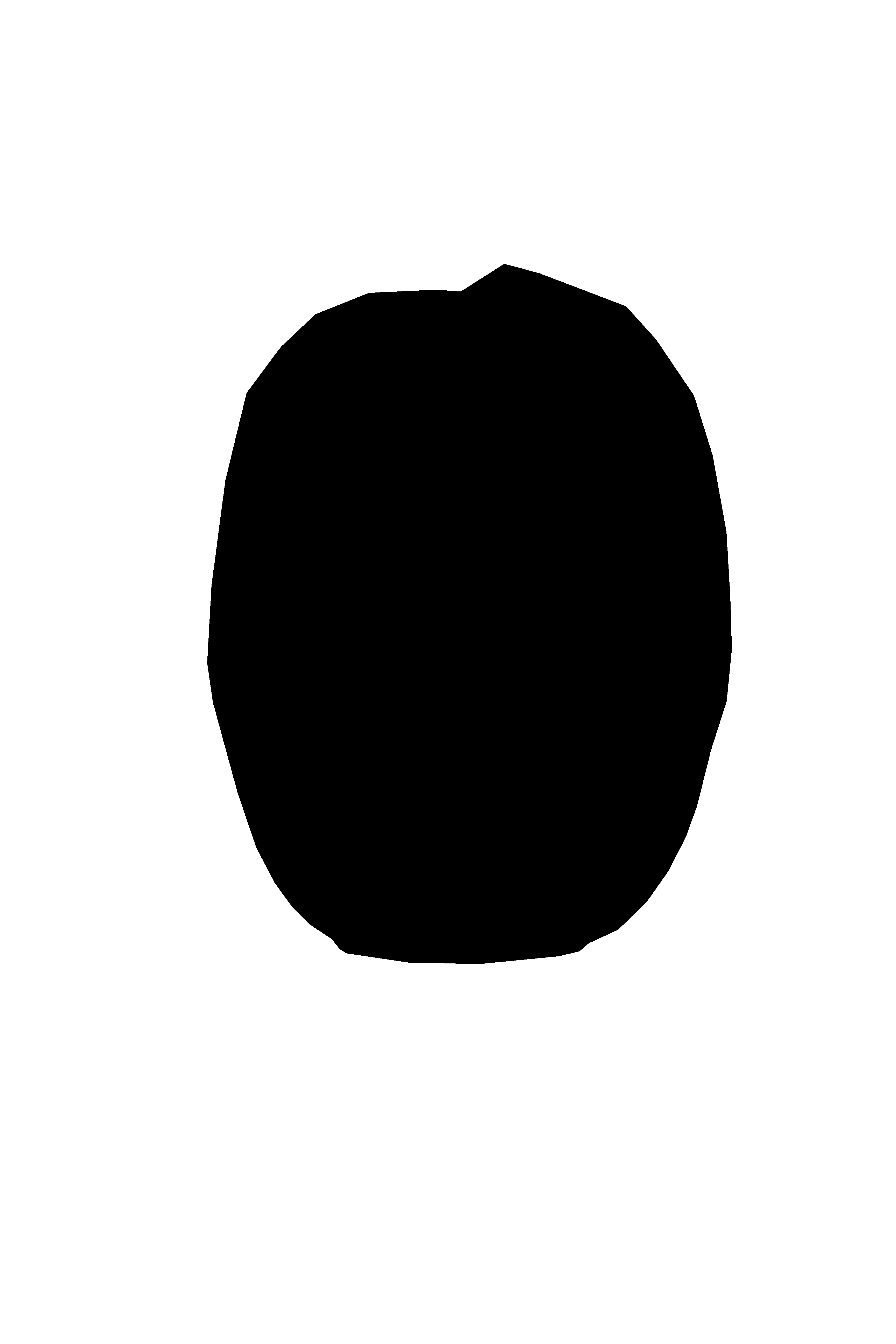

Supplement: Supplementary file 1 [file Data_Sheet_1.zip › face/089_face_mask.png]

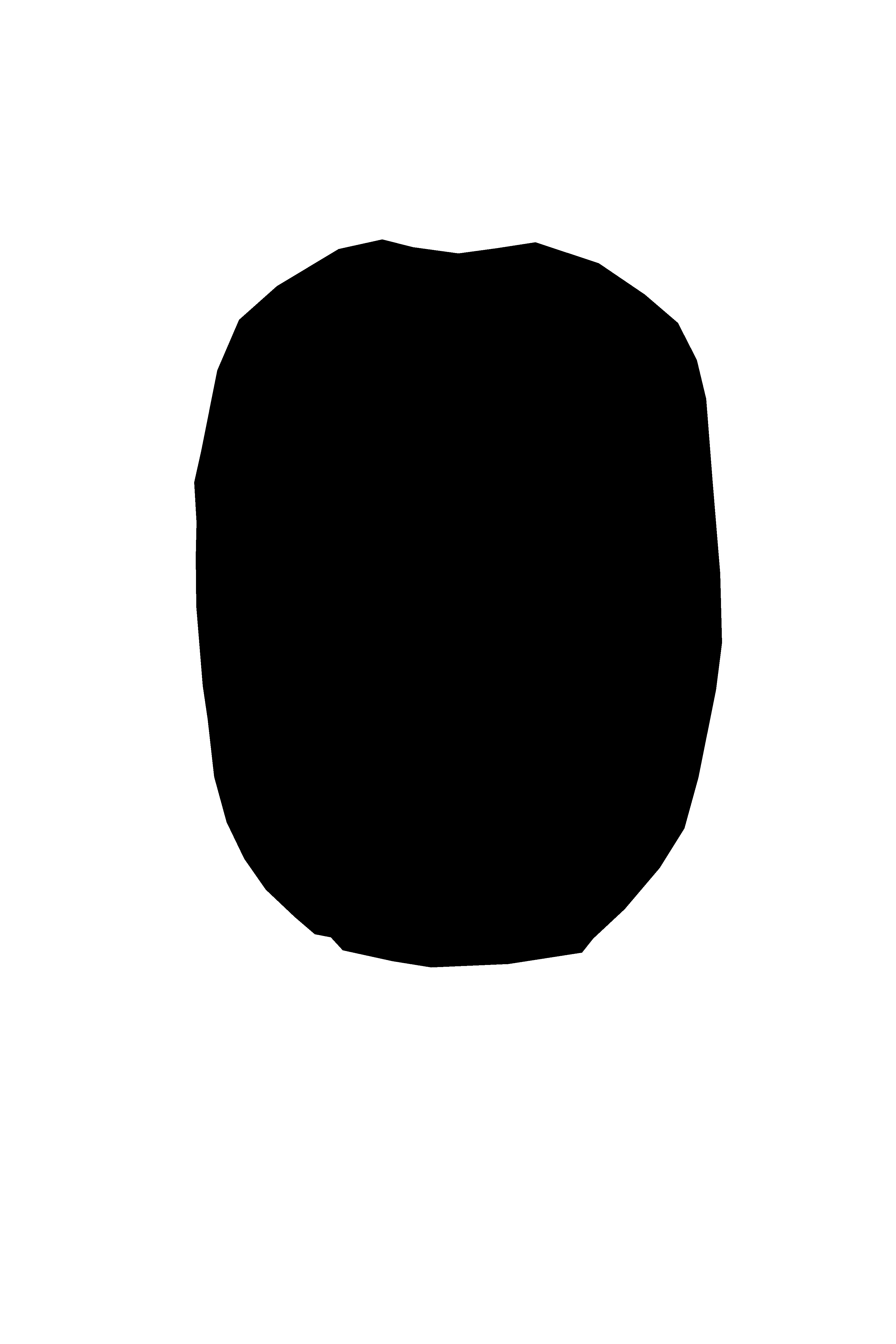

Supplement: Supplementary file 1 [file Data_Sheet_1.zip › face/090_face_mask.png]

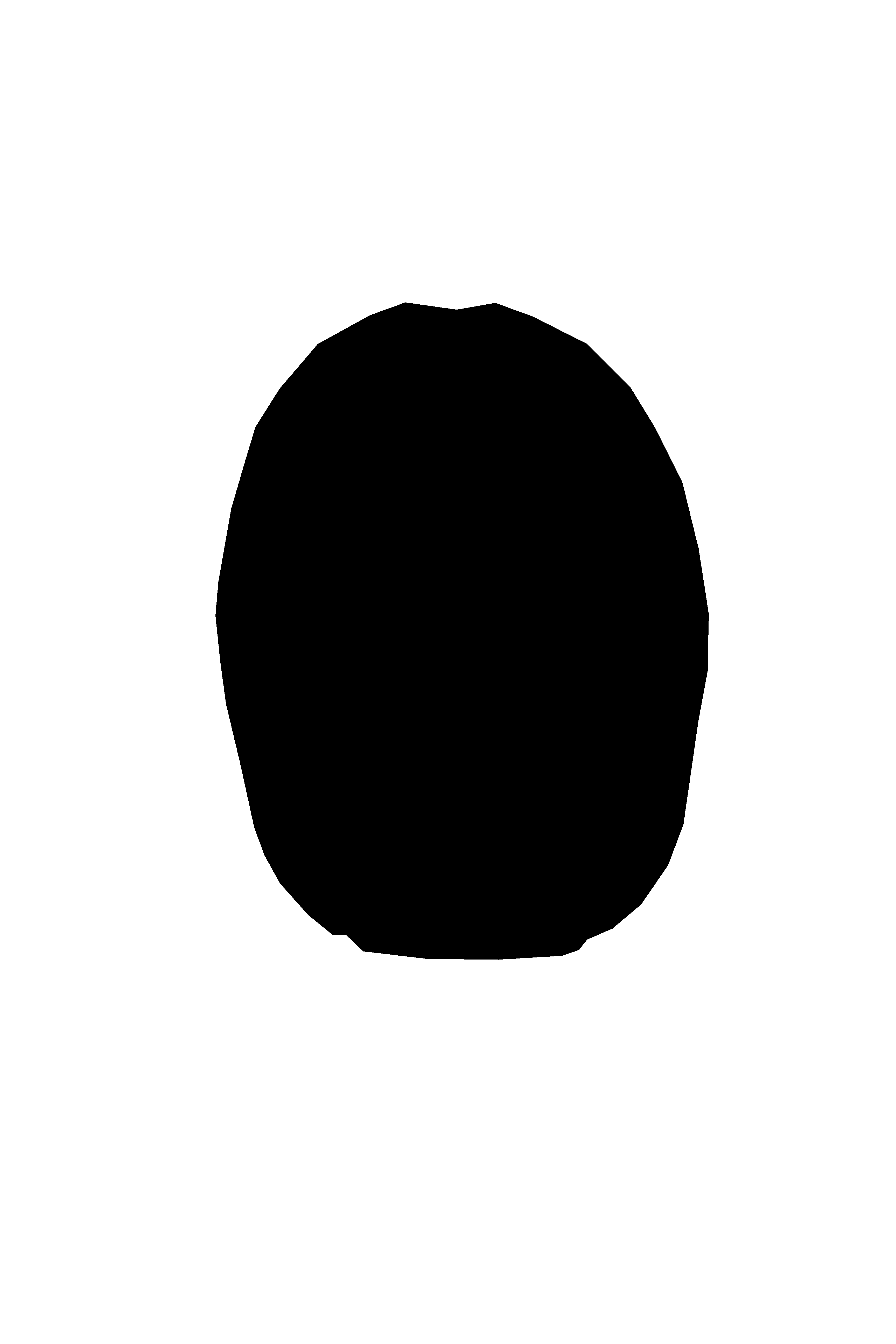

Supplement: Supplementary file 1 [file Data_Sheet_1.zip › face/091_face_mask.png]

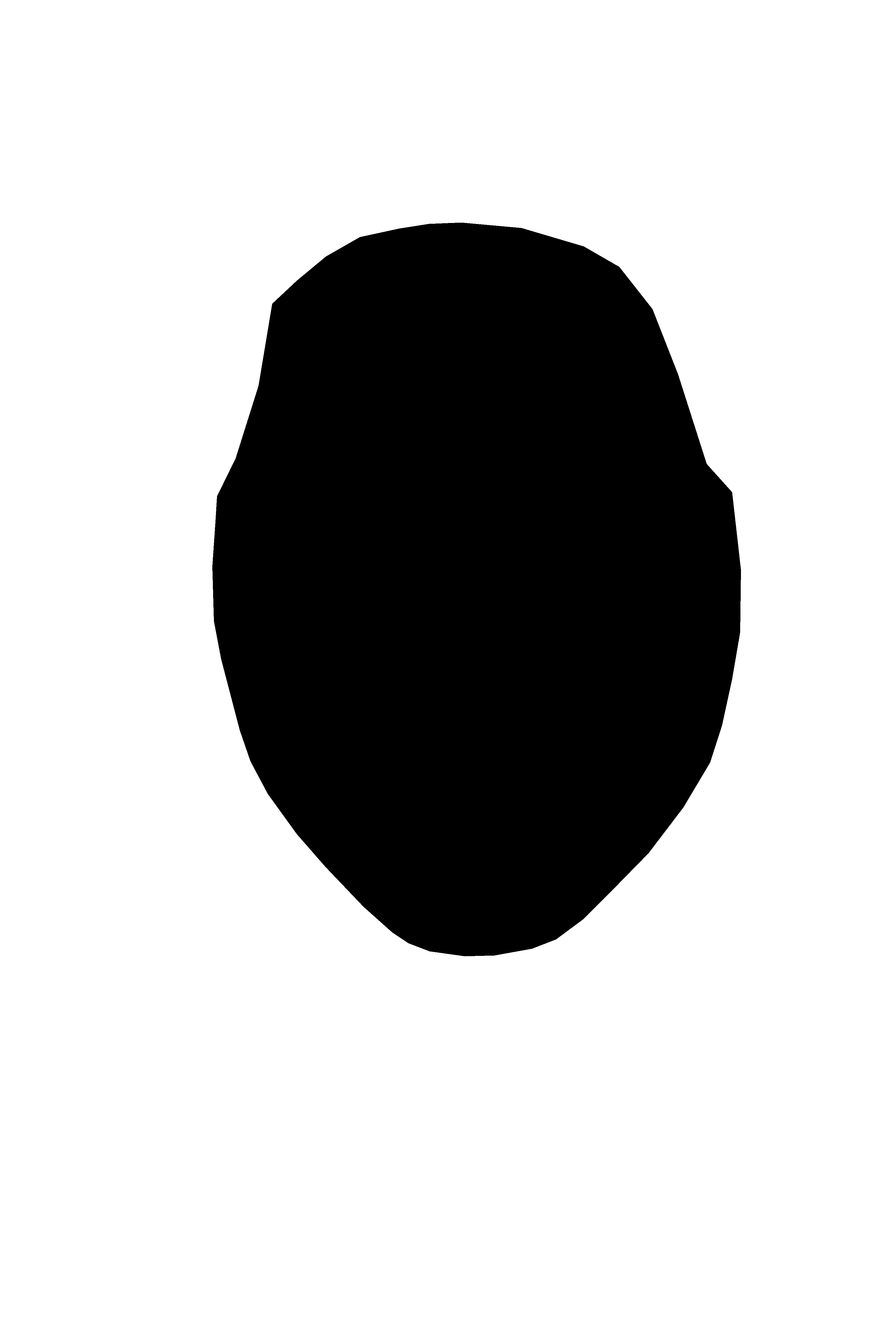

Supplement: Supplementary file 1 [file Data_Sheet_1.zip › face/092_face_mask.png]

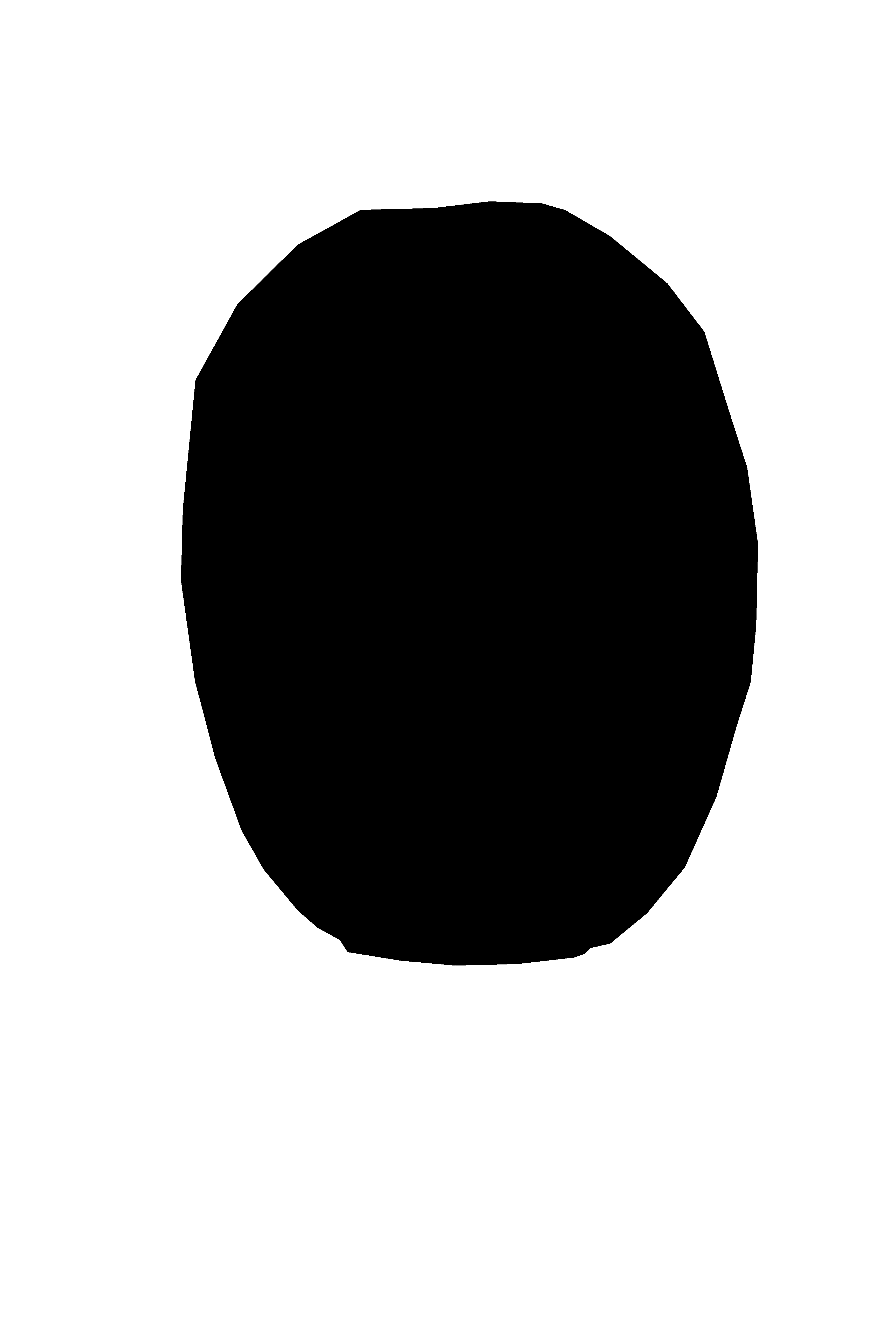

Supplement: Supplementary file 1 [file Data_Sheet_1.zip › face/093_face_mask.png]

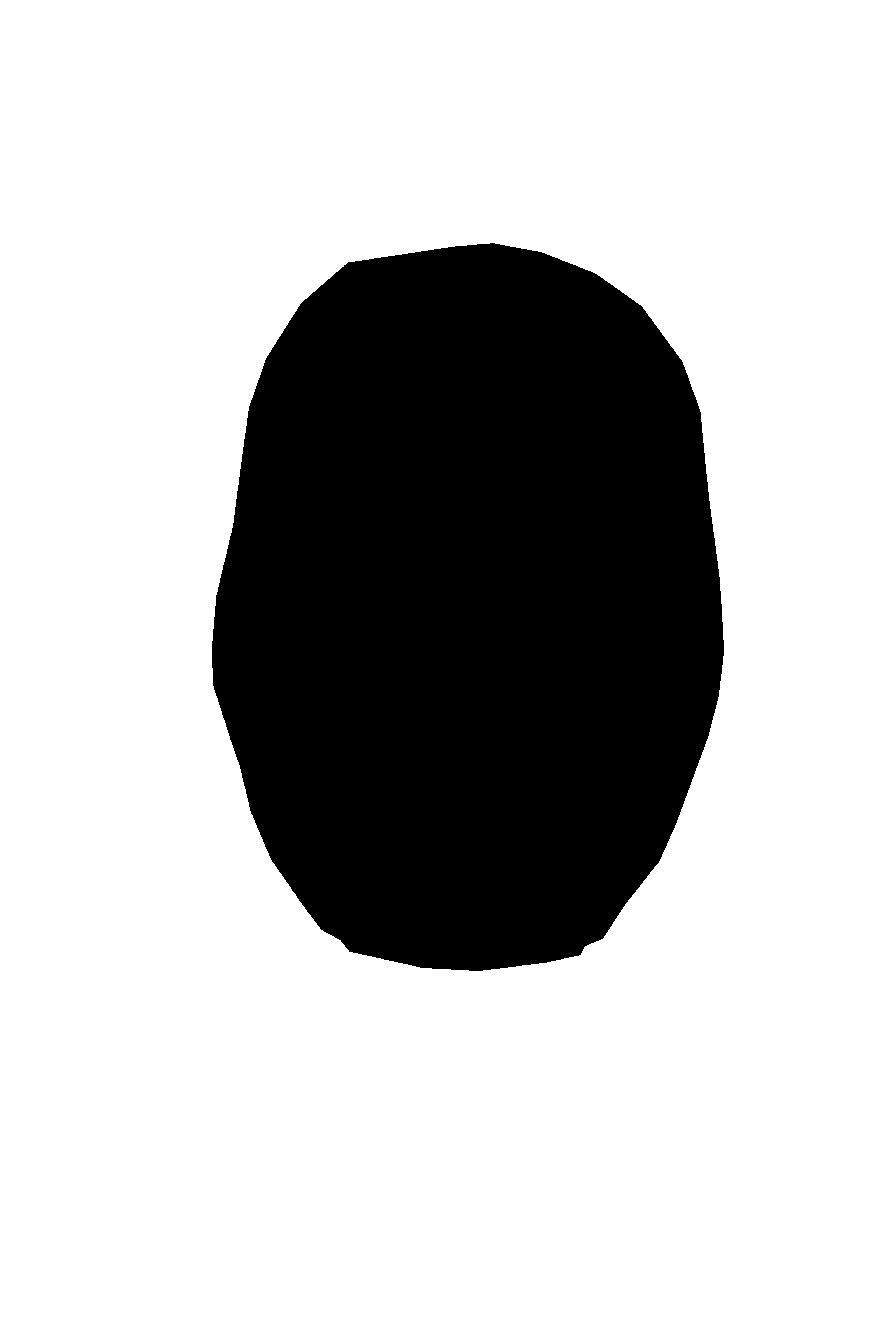

Supplement: Supplementary file 1 [file Data_Sheet_1.zip › face/094_face_mask.png]

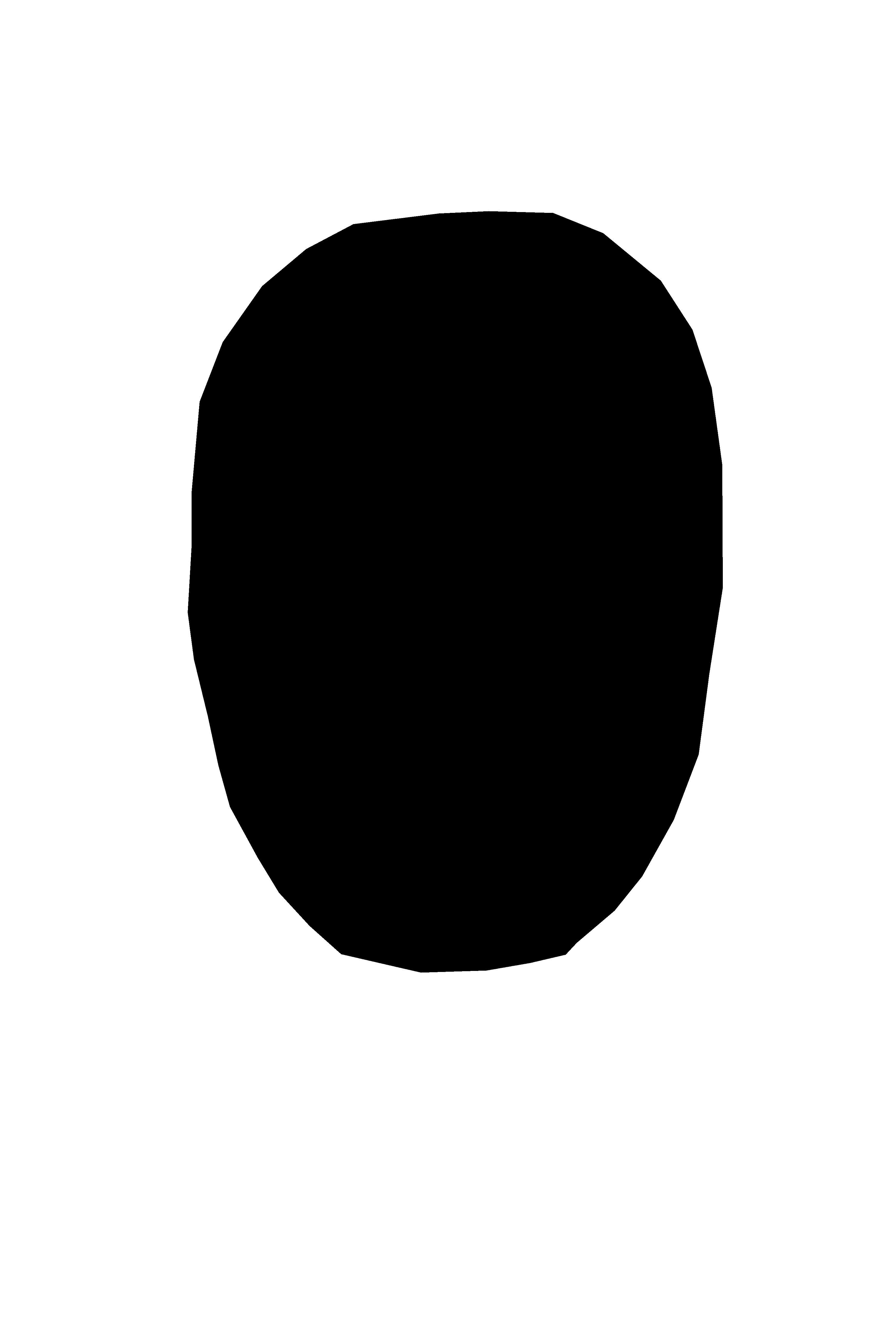

Supplement: Supplementary file 1 [file Data_Sheet_1.zip › face/095_face_mask.png]

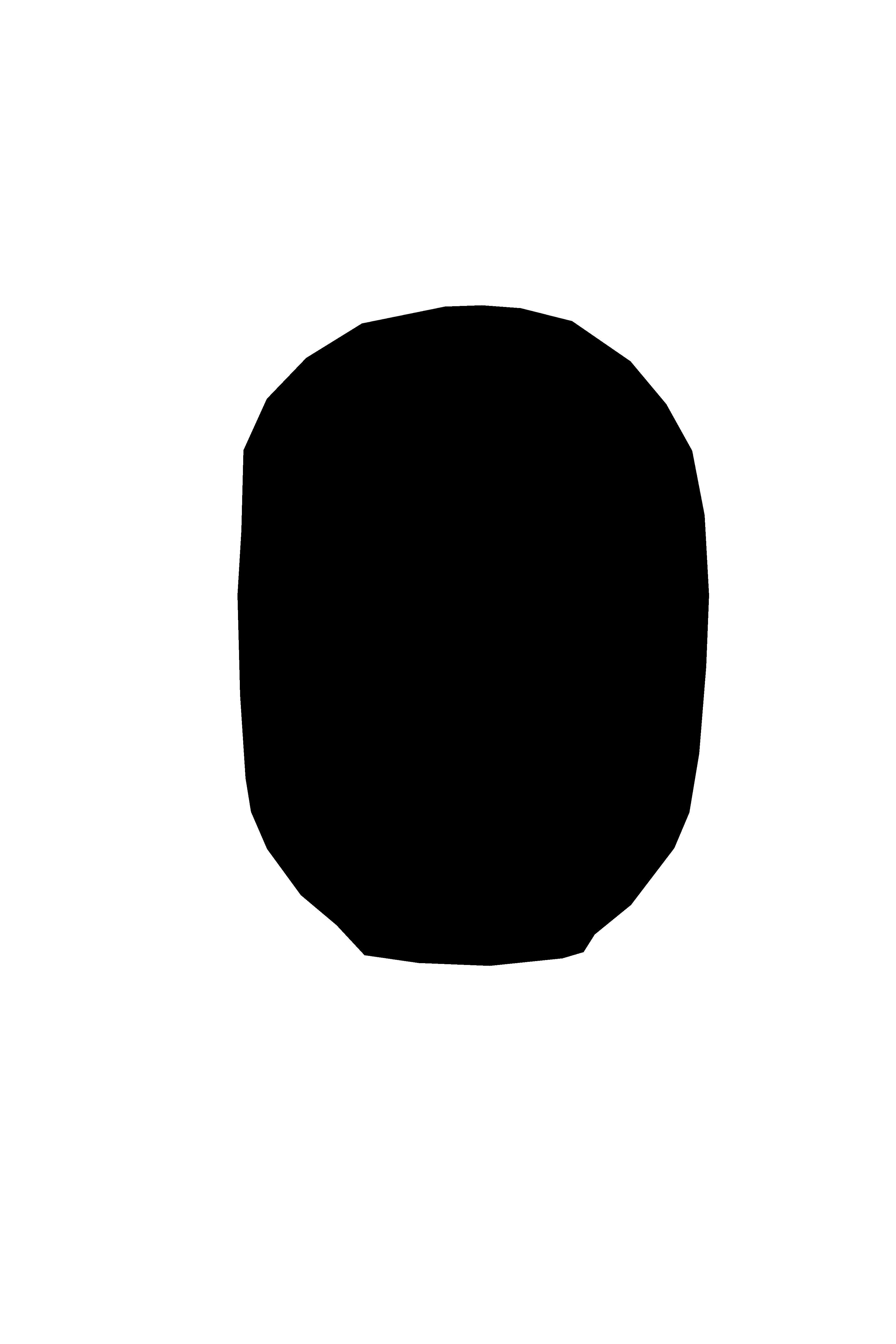

Supplement: Supplementary file 1 [file Data_Sheet_1.zip › face/096_face_mask.png]

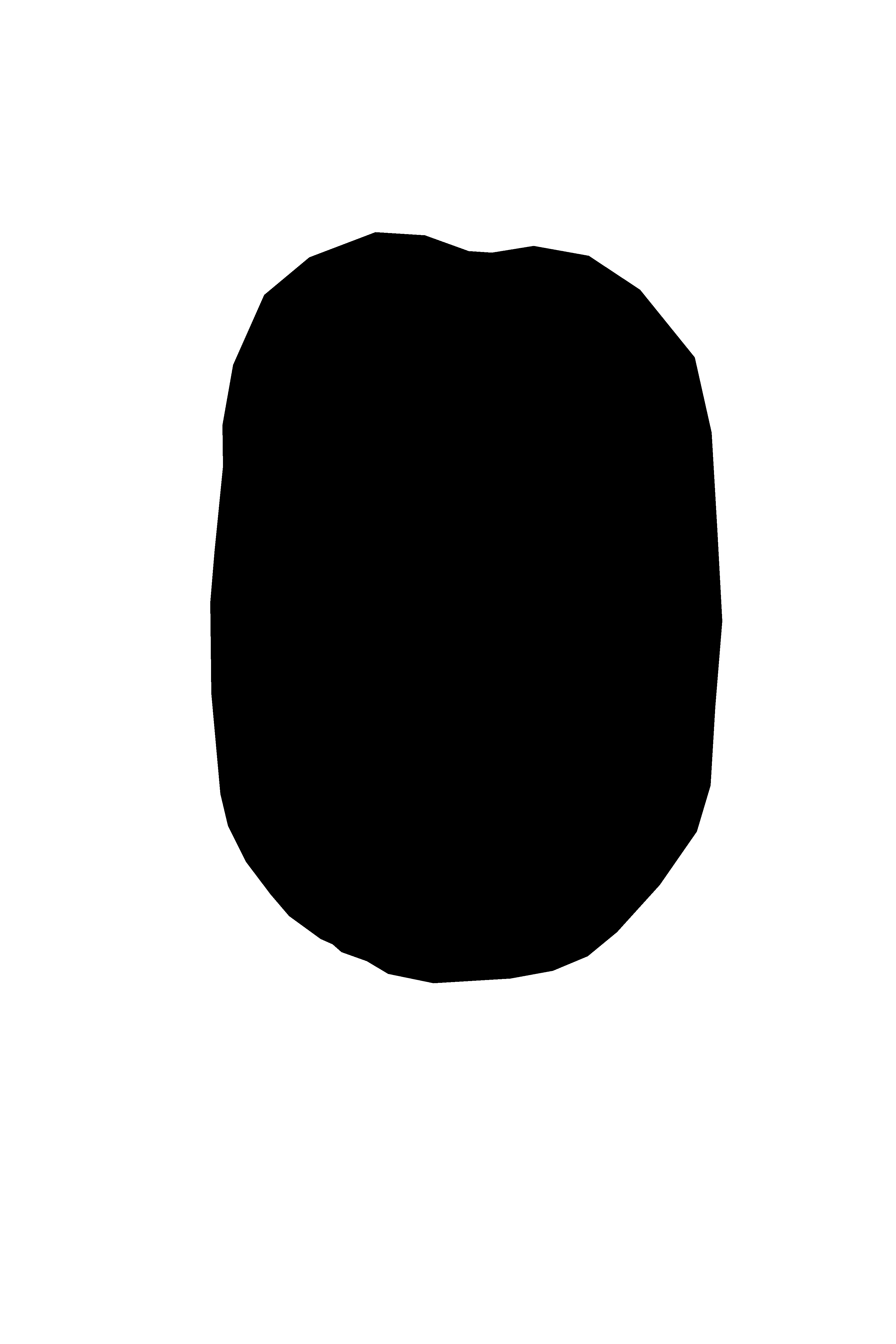

Supplement: Supplementary file 1 [file Data_Sheet_1.zip › face/097_face_mask.png]

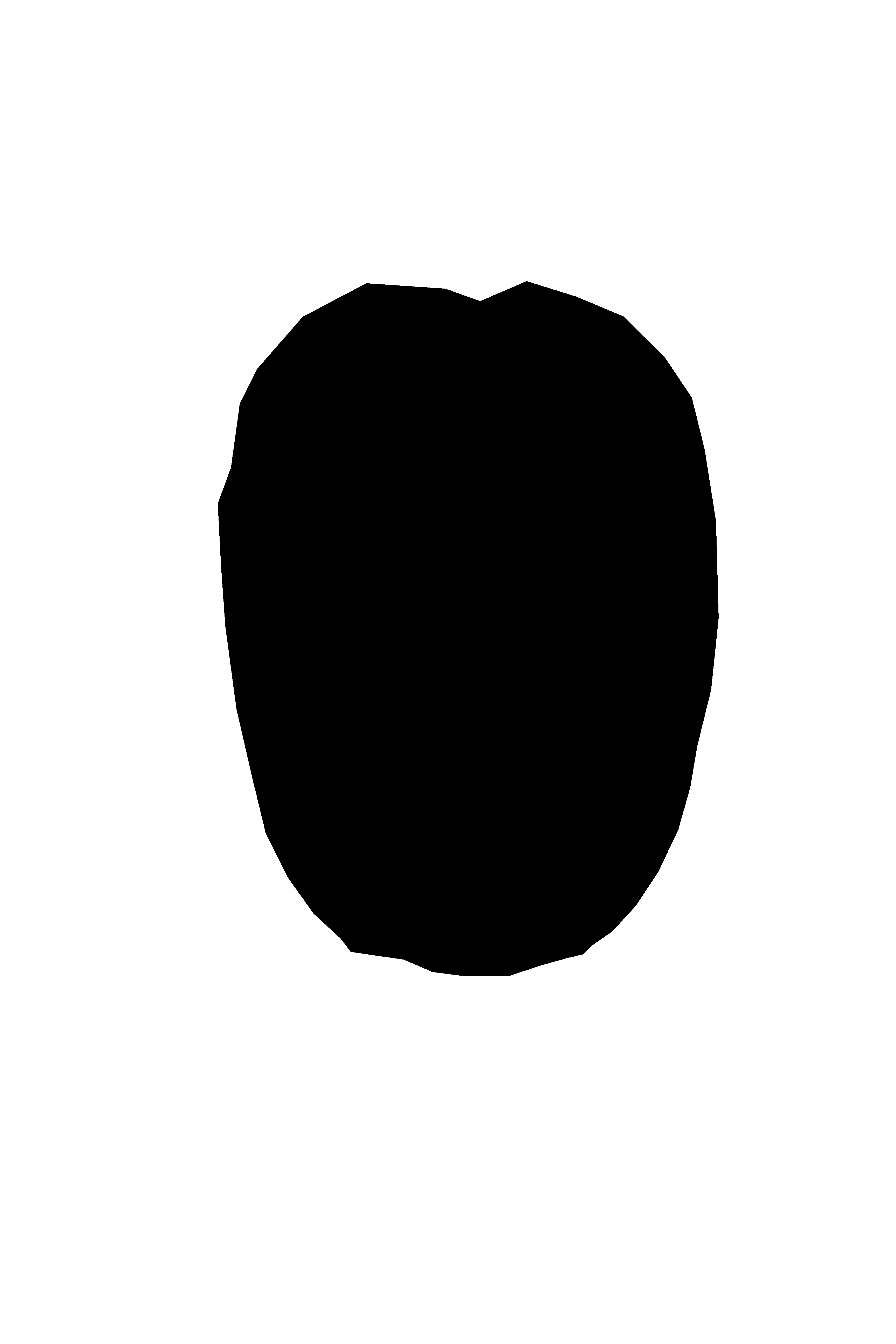

Supplement: Supplementary file 1 [file Data_Sheet_1.zip › face/098_face_mask.png]

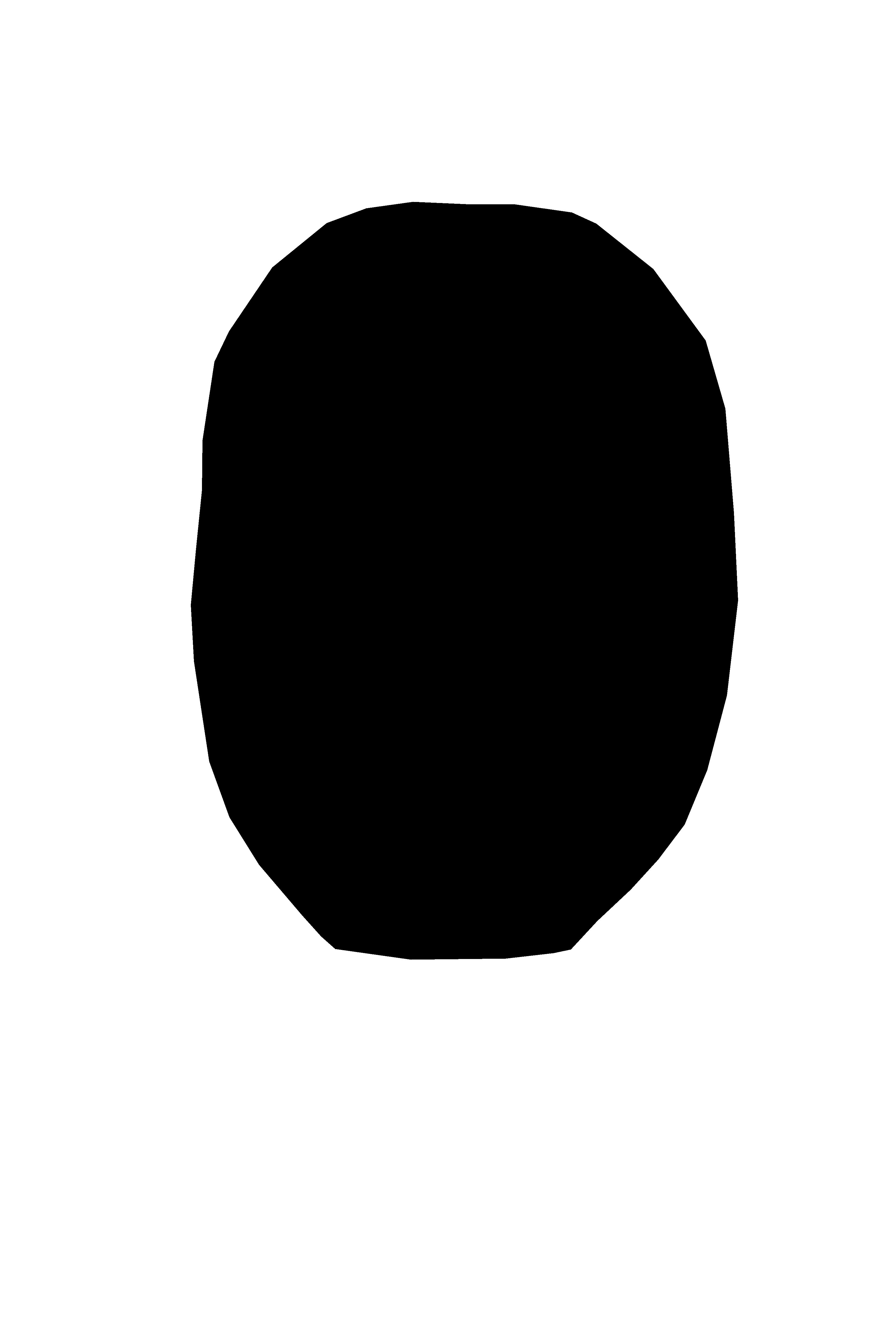

Supplement: Supplementary file 1 [file Data_Sheet_1.zip › face/099_face_mask.png]

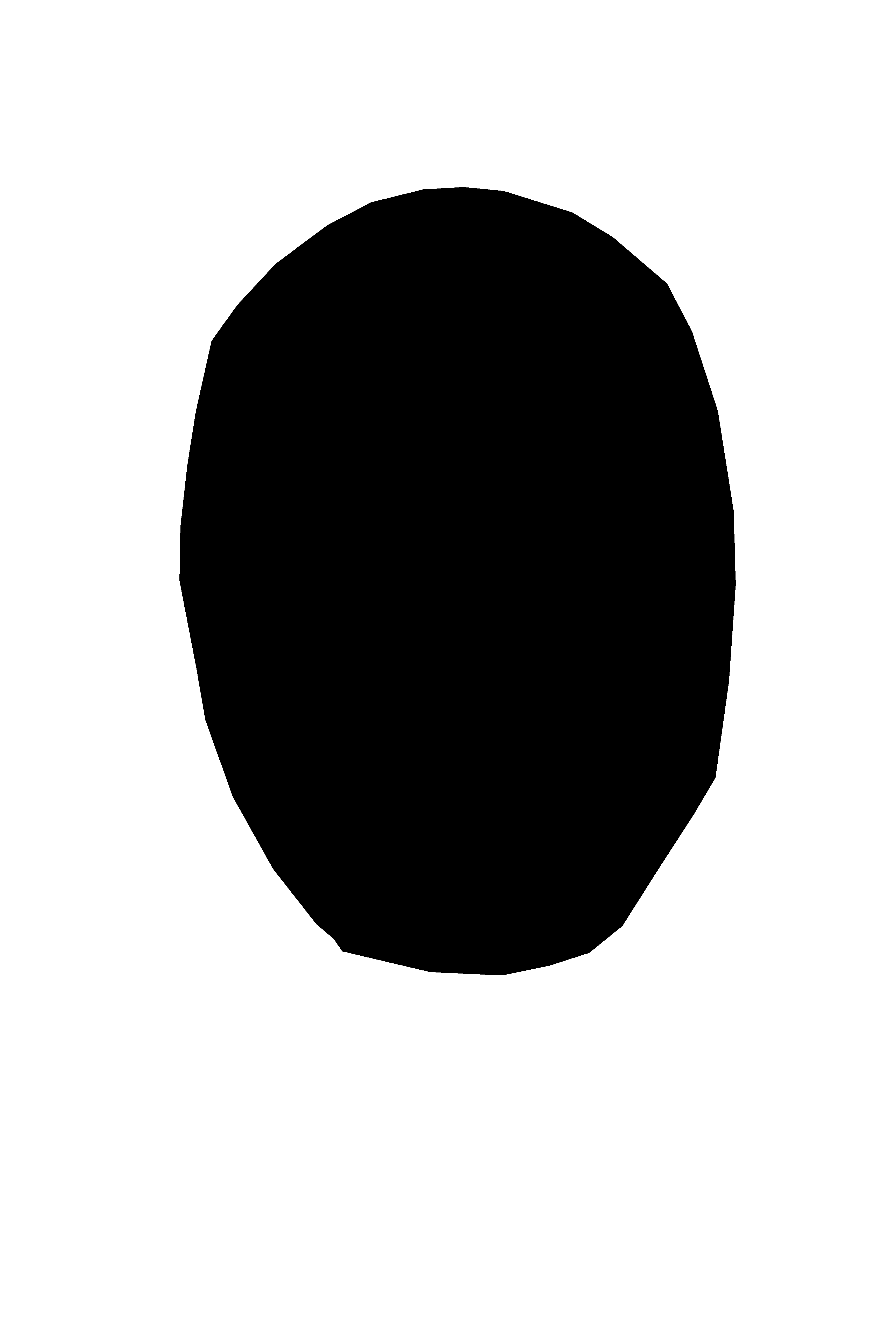

Supplement: Supplementary file 1 [file Data_Sheet_1.zip › face/100_face_mask.png]
