# Supplementary figures and images for: An explainable dual-modal diagnostic model for coronary artery disease: a feature-gated approach using tongue and facial image features (part 3 of 3)
Source: Front Artif Intell. 2025 Nov 17;8:1662577. doi: 10.3389/frai.2025.1662577 (PMC12665729; doi:10.3389/frai.2025.1662577)

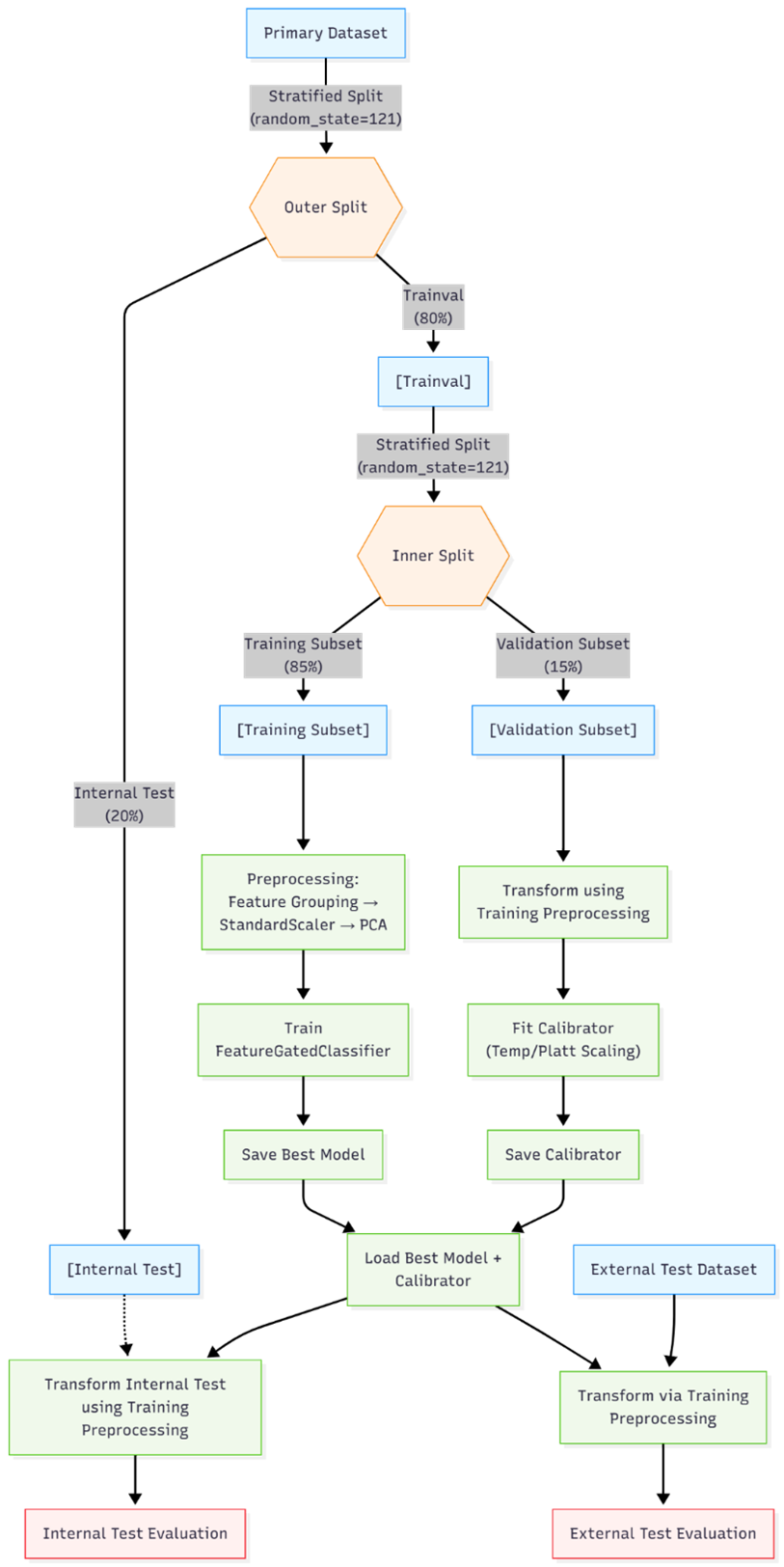

Supplement: Supplementary file 3 [file Image_1.png]

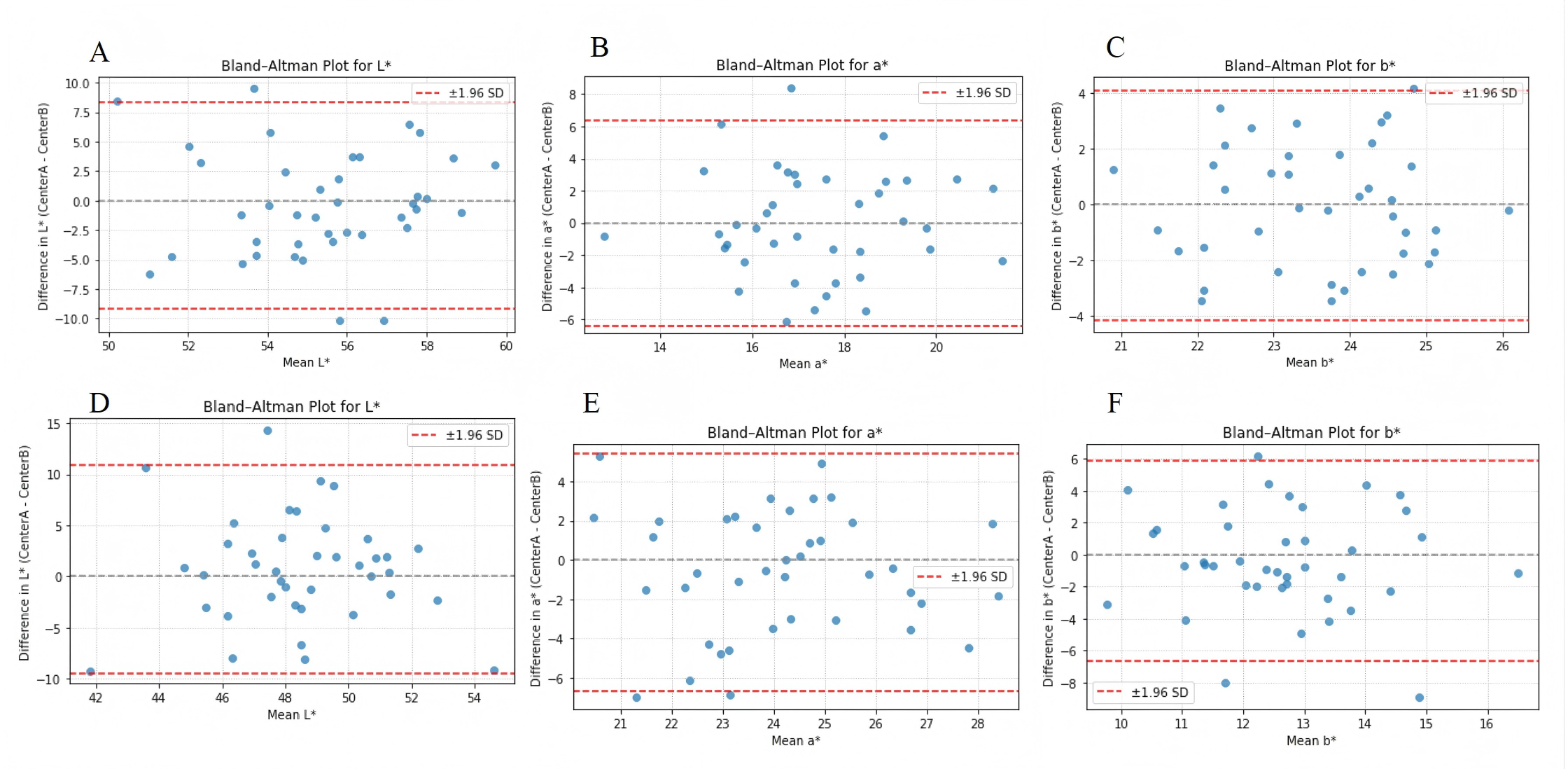

Supplement: Supplementary file 4 [file Image_2.png]
