# Supplementary material for: An explainable dual-modal diagnostic model for coronary artery disease: a feature-gated approach using tongue and facial image features
Source: Front Artif Intell. 2025 Nov 17;8:1662577. doi: 10.3389/frai.2025.1662577 (PMC12665729; doi:10.3389/frai.2025.1662577)
Supplement: Supplementary file 5 [file Table_1.docx]

**Training Pipeline**

1. **Random Seeds**
   - Fixed seeds: np.random.seed(121) and torch.manual_seed(121)
   - Used to ensure reproducibility of the training process and data splits.
2. **Software and Library Versions**
   - Python 3.10
   - PyTorch 2.1.0
   - scikit-learn 1.2.2
   - NumPy 1.26.5
   - pandas 2.1.1
   - Matplotlib 3.7.2
3. **PCA and Feature Standardization**
   - Principal Component Analysis (PCA) was applied separately to facial and tongue features, retaining 85% of the cumulative variance for each.
   - PCA was fitted **only on the training subset**; validation and test sets were transformed using the fitted PCA components.
   - All features were standardized using StandardScaler, which was also fitted **exclusively on the training subset**; validation and test sets underwent only transformation.
4. **Training Hyperparameters**

| **Parameter** | **Value** | **Description** |
| --- | --- | --- |
| Maximum epochs | 200 | n_epochs |
| Batch size | 32 | batch_size |
| Validation set ratio | 15% | val_ratio |
| Early stopping patience | 25 | patience |
| Gating network hidden dim | 256 | hidden_dim |
| Dropout rate | 0.35 | dropout_rate |
| Initial learning rate | 1e-3 | initial_lr |
| Weight decay | 1e-5 | weight_decay |
| Learning rate scheduler | ReduceLROnPlateau(factor=0.5, patience=6) | Learning rate adjusted based on validation AUC |

- **Model Architecture**
  - A gating network is employed to fuse facial and tongue features, learning modality-specific weights via a Softmax layer.
  - The classifier consists of two fully connected hidden layers with Batch Normalization, ReLU activation, and Dropout.
  - The output layer produces binary predictions (CHD vs. non-CHD).
- **Class Imbalance Handling**
  - Class weights were automatically computed using compute_class_weight('balanced') from scikit-learn and incorporated into the CrossEntropyLoss function to mitigate the impact of class imbalance.
