# Supplementary material for: An explainable dual-modal diagnostic model for coronary artery disease: a feature-gated approach using tongue and facial image features
Source: Front Artif Intell. 2025 Nov 17;8:1662577. doi: 10.3389/frai.2025.1662577 (PMC12665729; doi:10.3389/frai.2025.1662577)
Supplement: Supplementary file 6 [file Table_2.docx]

Table S1. Model performance of TF_FGC across internal and external acquisition sites

| **Dataset** | **AUC (95% CI)** | **AUPRC** | **Calibration Slope** | **Intercept** | **Brier Score** |
| --- | --- | --- | --- | --- | --- |
| Site A (Internal) | 0.945 (0.905–0.985) | 0.934 | 0.927 | −0.259 | 0.091 |
| Site B (External) | 0.896 (0.850–0.941) | 0.902 | 1.350 | −0.035 | 0.130 |
