# Supplementary material for: An explainable dual-modal diagnostic model for coronary artery disease: a feature-gated approach using tongue and facial image features
Source: Front Artif Intell. 2025 Nov 17;8:1662577. doi: 10.3389/frai.2025.1662577 (PMC12665729; doi:10.3389/frai.2025.1662577)
Supplement: Supplementary file 7 [file Table_3.docx]

Table S2 Subgroup-Specific Prevalence, PPV, NPV, and Calibration Metrics

| Subgroup | N | Prevalence (%) | PPV (%) | NPV (%) | Slope | Intercept |
| --- | --- | --- | --- | --- | --- | --- |
| age_group = <50 | 14 | 42.86 | 75.00 | 100.00 | 4.40 | -4.22 |
| age_group = 50–69 | 129 | 48.84 | 85.48 | 85.07 | 1.38 | 0.07 |
| age_group = ≥70 | 57 | 54.39 | 79.41 | 82.61 | 1.24 | -0.03 |
| Male | 110 | 50.91 | 81.82 | 80.00 | 1.22 | 0.07 |
| Female | 90 | 48.89 | 83.67 | 92.68 | 1.57 | -0.21 |
| Diabetes | 148 | 47.97 | 78.95 | 84.72 | 1.39 | -0.18 |
| Non-Diabetes | 52 | 55.77 | 92.86 | 87.50 | 1.29 | 0.40 |
| Hypertension | 94 | 35.11 | 73.81 | 96.15 | 1.74 | -1.01 |
| Non-Hypertensive | 106 | 63.21 | 88.71 | 72.73 | 1.19 | 0.60 |
